# The *Legionella* effector RidL binds the large fission GTPase Drp1 to promote mitochondrial fragmentation

**DOI:** 10.1038/s44319-026-00823-3

**Published:** 2026-06-06

**Authors:** Ana Katic, Elizabeth Teresa Vittori, Stefanie Halter, Kevin Steiner, A Leoni Swart, Milad Radiom, Francisco Javier García-Rodríguez, Tobias Jäggi, Xiaodan Li, Jason A Mears, Carmen Buchrieser, Pedro Escoll, Vikram Govind Panse, Hubert Hilbi

**Affiliations:** 1 Institute of Medical Microbiology, University of Zürich, Zürich, Switzerland; 2 Department of Health Sciences and Technology, ETH Zürich, Zürich, Switzerland; 3 Institut Pasteur, Université Paris Cité, CNRS UMR 6047, Paris, France; 4 Paul Scherrer Institute, Villigen, Switzerland; 5 Case Western Reserve University School of Medicine, Cleveland, OH USA; 6 Faculty of Science, University of Zürich, Zürich, Switzerland; 7 Present Address: Institute of Medical Virology, University of Zürich, Zürich, Switzerland; 8 Present Address: Biozentrum, University of Basel, Basel, Switzerland

**Keywords:** Metabolism, Microbiology, Virology & Host Pathogen Interaction, Signal Transduction

## Abstract

Intracellular pathogens such as *Legionella pneumophila* secrete effector proteins that manipulate host cell processes to promote bacterial survival. One such effector, RidL, is known to inhibit retrograde trafficking by interacting with the retromer complex via its N-terminal domain. Here, we identify a second function of RidL mediated by its C-terminal domain, which directly binds to the mitochondrial fission GTPase dynamin-related protein 1 (Drp1) and related large GTPases. In vitro, RidL reduces Drp1 GTPase activity and disrupts its oligomerization. During infection, RidL localizes to mitochondria, enhances the accumulation of Drp1 and the outer membrane protein Tom20, and impairs mitochondrial dynamics and function. Moreover, in *L. pneumophila*-infected cells, RidL promotes phosphorylation of Drp1 at Ser616, leading to Drp1 activation and mitochondrial fragmentation. These findings establish RidL as a bifunctional effector that targets both the retromer complex and Drp1 through distinct domains. By interfering with host mitochondrial dynamics, RidL enables *L. pneumophila* to remodel host organelles and optimize conditions for intracellular replication.

## Synopsis

The bacterial pathogen *Legionella pneumophila* subverts host mitochondria. The secreted effector protein RidL binds the mitochondrial large fission GTPase Drp1, localizes to mitochondria and promotes Drp1 activation, mitochondrial fragmentation and inhibition of respiration.

- The bacterial pathogen *Legionella pneumophila* subverts host mitochondria through secreted effector proteins.
- The *L. pneumophila* effector protein RidL binds the mitochondrial large fission GTPase Drp1 in vitro.
- RidL promotes recruitment of Drp1 to mitochondria, their fragmentation, and respiration inhibition in infected cells.
- RidL-dependent phosphorylation and activation of Drp1 correlates with mitochondrial fragmentation.

## Abstract (web summary)

The bacterial pathogen *Legionella pneumophila* subverts host mitochondria. The secreted effector protein RidL binds the mitochondrial large fission GTPase Drp1, localizes to mitochondria and promotes Drp1 activation, mitochondrial fragmentation and inhibition of respiration.

## Graphical abstract

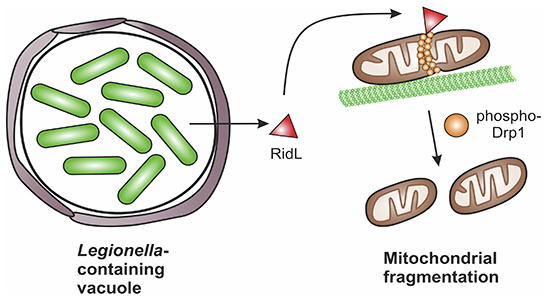

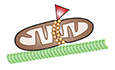

## Introduction

The environmental bacterium *Legionella pneumophila* is a facultative intracellular pathogen, which can cause a life-threatening pneumonia called Legionnaires’ disease (Mondino et al, 2020; Newton et al, 2010). *L. pneumophila* manipulates eukaryotic host cells in a highly sophisticated manner and replicates intracellularly in a unique compartment termed the “*Legionella*-containing vacuole” (LCV) (Mondino et al, 2020; Newton et al, 2010; Steiner et al, 2018). The LCV captures Golgi-derived vesicles (Weber et al, 2018) and forms tight “membrane-contact sites” with the endoplasmic reticulum (ER), which are implicated in lipid flux and pathogen vacuole formation (Steiner et al, 2017; Vormittag et al, 2023). To govern pathogen–host cell interactions, *L. pneumophila* employs a small signaling molecule termed *Legionella* autoinducer-1 (LAI-1) (Michaelis et al, 2024) as well as the intracellular multiplication/defective organelle transport (Icm/Dot) type IV secretion system (T4SS) that translocates more than 300 different “effector proteins” into host cells (Hilbi and Buchrieser, 2022; Personnic et al, 2016; Qiu and Luo, 2017). The effector proteins subvert pivotal processes, including vesicle trafficking, cytoskeletal dynamics, and signal transduction. Some of the secreted effectors target the mitochondria or their dynamics (Garcia-Rodriguez et al, 2023), and act as a nucleotide carrier protein (Dolezal et al, 2012), modify ADP/ATP translocases by reversible ADP-ribosylation (Fu et al, 2022; Kubori et al, 2022), cleave syntaxin 17 (Arasaki et al, 2017), or stabilize microtubules as Ran GTPase activators (Escoll et al, 2017; Rothmeier et al, 2013; Swart et al, 2020).

Mitochondria are highly dynamic organelles, and the fission, fusion, trafficking and interactions of these organelles are essential for their function as cellular “powerhouses” and signaling hubs, controlling metabolism, proliferation, and apoptosis (Chan, 2020; Giacomello et al, 2020). Defects in these processes are linked to ageing (Sharma et al, 2019) and many diseases, including neurodegenerative disorders (Cutillo et al, 2020), cardiovascular ailments (Quiles and Gustafsson, 2022), and cancer (Zong et al, 2016).

A pivotal regulator of mitochondrial dynamics is the 78 kDa large fission GTPase dynamin-related protein 1 (Drp1), which acts in several steps: (i) subcellular translocation from the cytoplasm to the outer mitochondrial membrane, (ii) assembly into multimeric tubular structures called spirals, and (iii) GTP hydrolysis associated with membrane “constriction” followed by disassembly (Giacomello et al, 2020). Drp1 activity is regulated by posttranslational modifications, including activation by phosphorylation at Ser616 (Chang and Blackstone, 2010). Another regulator of mitochondrial dynamics is the retromer coat complex, comprising the cargo recognition subcomplex subunits Vps35, Vps29, and Vps26, as well as membrane-deforming sorting nexins (SNXs) (Bonifacino and Hurley, 2008; Cutillo et al, 2020).

The retromer complex and Drp1 regulate the formation of mitochondria-derived vesicles (MDVs), operating in mitochondrial quality control and protein turnover pathways that transport selected cargo proteins and lipids to lysosomes or peroxisomes for degradation (Braschi et al, 2010; König and McBride, 2024; König et al, 2021; Soubannier et al, 2012; Sugiura et al, 2014). In some MDV pathways, the retromer initiates membrane curvature, followed by the elongation of membrane protrusions pulled along microtubule filaments and scission by Drp1. Specifically, the retromer Vps35 subunit regulates the MDV-dependent turnover of Drp1 itself (Wang et al, 2017; Wang et al, 2016) as well as the mitochondrial outer membrane protein Tom20, a component of the import receptor complex transporting cytoplasmic mitochondrial precursor proteins (König and McBride, 2024; König et al, 2021). The fusion of MDVs with lysosomes is mediated by the SNARE syntaxin 17 (McLelland et al, 2016).

An imbalance in retromer- and Drp1-dependent mitochondrial dynamics underlies neurodegenerative disorders. In Huntington’s disease, mutant forms of the protein huntingtin abnormally interact with Drp1 and stimulate its enzymatic activity (Song et al, 2011). In Parkinson’s disease, mutant forms of Vps35 showed an enhanced interaction with Drp1, which promotes the removal of inactive Drp1 complexes via MDV-dependent trafficking to lysosomes, leading to the polymerization of new active Drp1 and mitochondrial fragmentation (Wang et al, 2017; Wang et al, 2016).

*L. pneumophila* produces a 131 kDa effector protein called RidL (retromer interactor decorating LCVs), which promotes intracellular replication, inhibits retrograde transport, and binds the Vps29 subunit of the retromer cargo recognition subcomplex (Finsel et al, 2013). The interaction of RidL with Vps29 occurs through a small amino acid stretch in the effector’s N-terminal fragment termed β-loop, and leads to the displacement on Vps29 of the Rab7 GTPase activating protein (GAP) TBC1D5 (Bärlocher et al, 2017; Finsel et al, 2013; Romano-Moreno et al, 2017; Yao et al, 2018). The interactor(s) and function of the C-terminal fragment of RidL are unknown. In this study, we identify the large mitochondrial GTPase Drp1 as an interactor of the C-terminal fragment of RidL, and we assess the role of RidL for the phosphorylation-dependent activation of Drp1, and its consequences for mitochondrial dynamics and functions.

## Results

### RidL binds to Drp1 and impairs GTPase activity

**Figure EV1:**
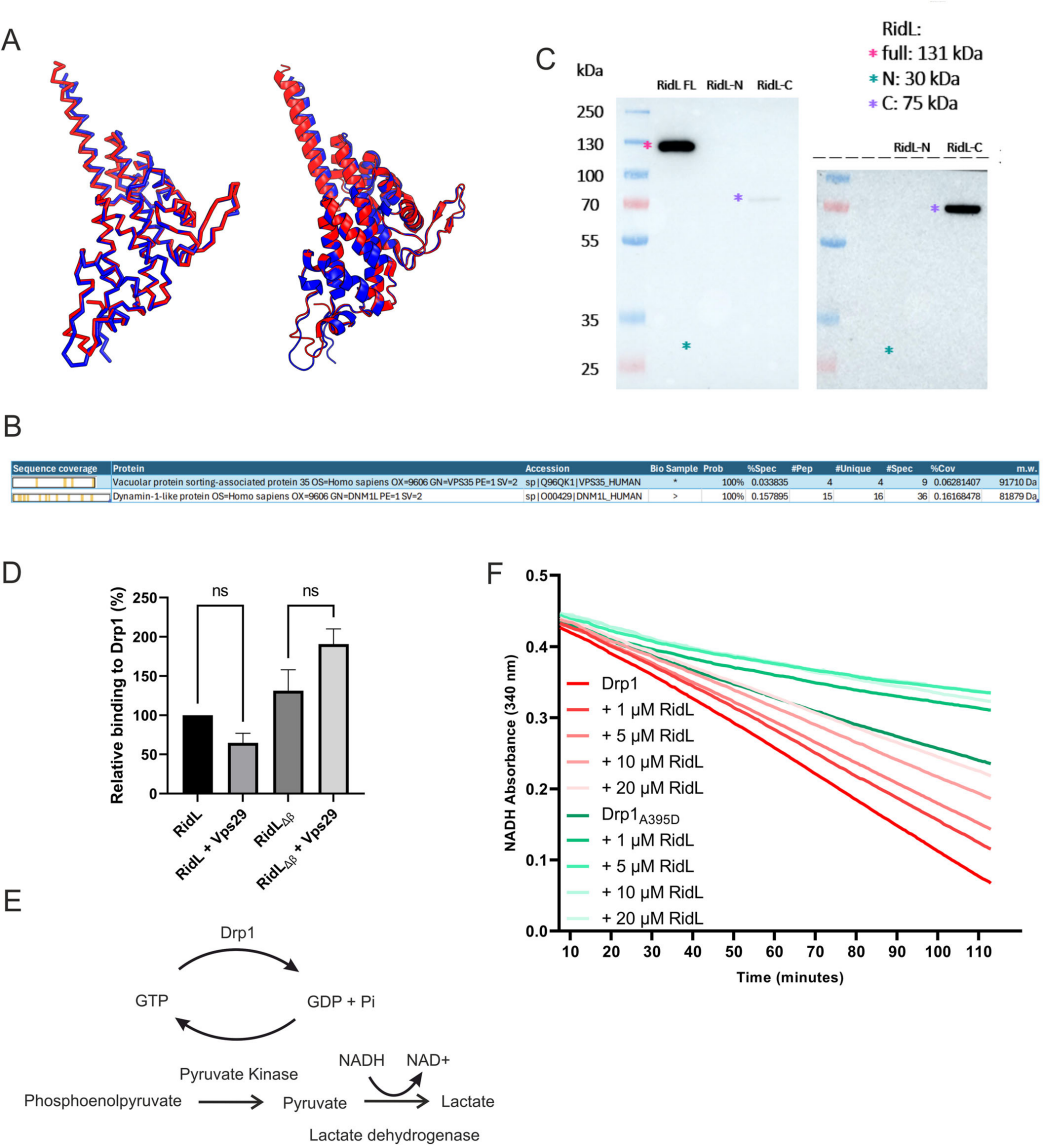
Binding of RidL to Drp1 and effect on Drp1 GTPase activity. (**A**) Comparison of the structure of the N-terminal fragment (213 amino acids) of RidL (PDB code: 5OH5, red) with the corresponding AlphaFold model (blue); ribbon overlay (left image) and cartoon overlay (right image). (**B**) Proteomics data identifying Vps35 and Drp1 as binding partners of RidL or RidL_Δβ_, respectively (screenshot). (**C**) Purified RidL (131 kDa), RidL_N_ (RidL_1–258_, 30 kDa), or RidL_C_ (RidL_437–1100_, 75 kDa) were incubated with purified Drp1 (78 kDa) coupled to UltraLink Biosupport beads. The beads were washed once, and bound proteins were separated by SDS–PAGE and visualized by anti-RidL Western blot. Regular (left panel, Fig. 1G) and prolonged exposure (right panel) is shown. (**D**) Densitometrical quantification of RidL bound to Drp1. Purified Drp1 was immobilized onto polyacrylamide beads and incubated with purified RidL or RidL_Δβ_ preincubated or not with purified Vps29. After washing, bound proteins were eluted, separated by SDS–PAGE, and visualized by silver staining. Band intensities corresponding to RidL were quantified by densitometry and normalized for each replicate to Drp1-bound RidL in the absence of Vps29. Bars indicate mean ± SEM from three biological replicates (*n* = 3). (**E**) Scheme of coupled enzyme assay for Drp1 GTPase activity, adapted from (Ingerman and Nunnari, 2005). (**F**) Graphical depiction of continuous NADH depletion over time reflecting Drp1 GTPase activity (*n* = 3) (means of two technical duplicates each from three biological replicates).

**Figure 1:**
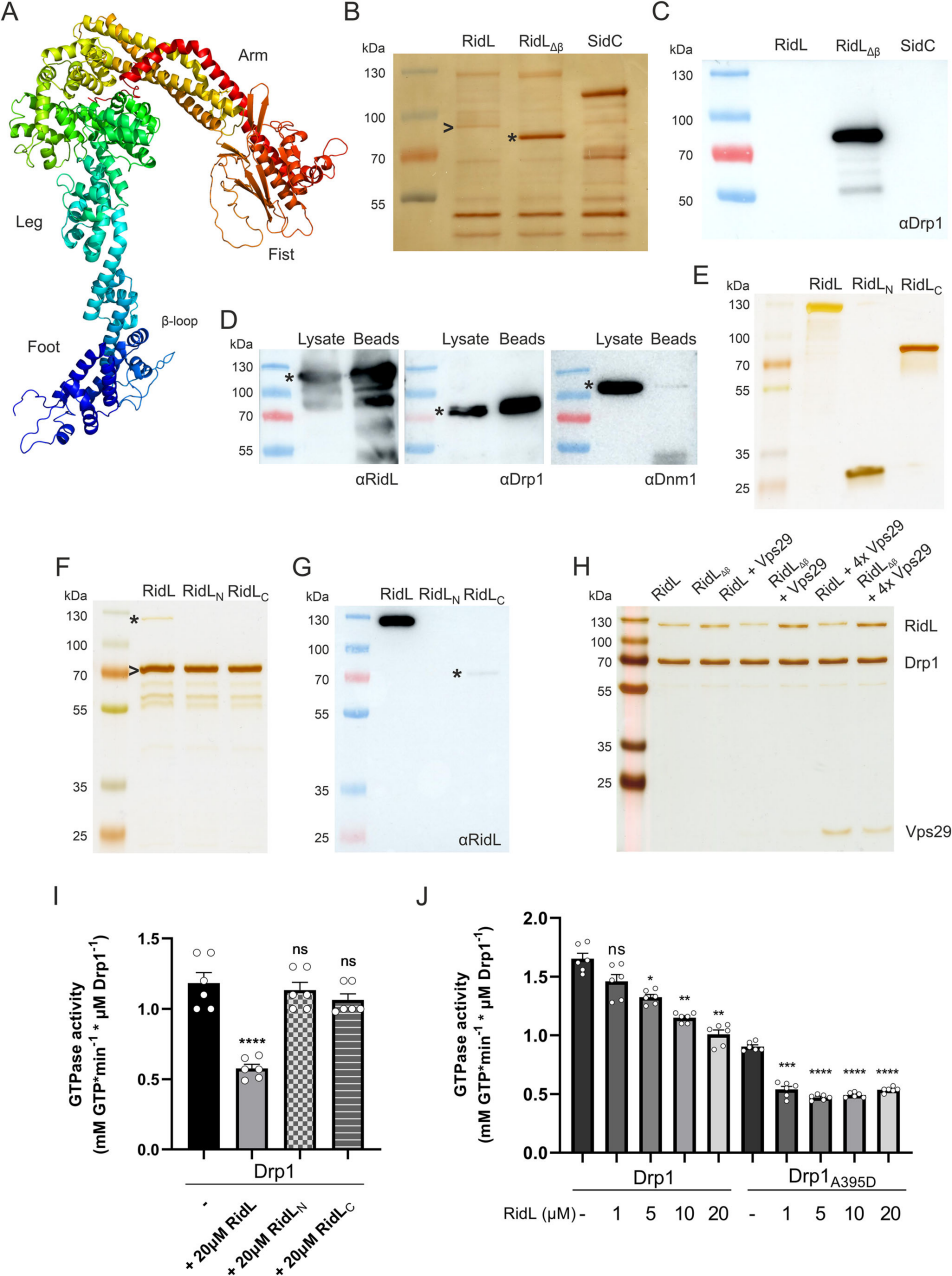
RidL binds to Drp1 and impairs GTPase activity in vitro. (**A**) Alphafold2 model of RidL. The model shows the “foot”, “leg”, “arm”, and “fist” domains. (**B**, **C**) Purified RidL, RidL_Δβ_ (lacking the Vps29-binding β-loop), or SidC was coupled to UltraLink Biosupport beads, incubated with HeLa cell lysate, and washed once. Proteins bound to washed beads were separated by SDS–PAGE and analyzed by (**B**) silver stain and mass spectrometry: protein bands labeled by “<” (Vps35) and “*” (Drp1), or (**C**) anti-Drp1 Western blot. (**D**) Co-immunoprecipitation of HeLa cells producing RidL. HeLa cells ectopically producing RidL were lysed, treated with an anti-RidL antibody, magnetic protein A/G beads, and washed. RidL (131 kDa), Drp1 (78 kDa) and Dnm1 (100 kDa) were analyzed by Western blot in cell lysates and after elution with SDS sample buffer from beads (indicated by “*”). The experiment was performed in two biological replicates (*n* = 2). (**E**–**G**) Purified RidL (131 kDa), RidL_N_ (RidL_1–258_, 30 kDa), or RidL_C_ (RidL_437–1100_, 75 kDa) were (**E**) visualized by silver stain, and equal amounts were incubated with purified Drp1 (78 kDa) coupled to UltraLink Biosupport beads. The beads were washed once, and bound proteins were separated by SDS–PAGE and visualized by (**F**) silver stain (“*” (RidL), “<” (Drp1), or (**G**) anti-RidL Western blot. (**H**) Purified RidL was incubated in the absence or presence of equimolar amounts of purified Vps29, prior to binding to purified Drp1 coupled to UltraLink Biosupport beads. The beads were washed, and RidL (131 kDa), Drp1 (78 kDa), and Vps29 (21 kDa) were visualized by silver stain. (**I**,** J**) Coupled enzymatic assay to assess GTPase activity of 1 µM purified Drp1 or the oligomerization mutant Drp1_A395D_ in presence or absence of purified RidL, RidL_N_ (RidL_1–258_), or RidL_C_ (RidL_437–1100_): Data from continuous NADH depletion assays converted to Drp1 GTPase activity (*n* = 3) (two technical duplicates each from three biological replicates, means + SEM; *p < 0.05; **p < 0.01; ***p < 0.001; ****p < 0.0001, ns not significant). Source data are available online for this figure.

Previous structural studies of an N-terminal fragment of RidL revealed a “foot-like” fold that at its “heel” binds via the “β-loop” to the Vps29 retromer subunit (Bärlocher et al, 2017; Romano-Moreno et al, 2017; Yao et al, 2018). The experimental structure closely matches the Alphafold2 structure prediction with an RMSD value of 0.445 Å for the 213 N-terminal amino acids (Fig. EV1A). The Alphafold2 model of full-length RidL further stipulates a “leg” domain, which is linked by an apparently flexible hinge to domains resembling an “arm” and a “fist” (Fig. 1A). According to the model, the C-terminus of RidL folds back along the “arm” domain as an extended, kinked α-helix and ends in the hinge region. The function and potential interaction partners of the C-terminal fragment of RidL are unknown. Based on these structural elements of RidL, we sought to identify further interaction partners of the effector.

To identify RidL binding partners in lysates of mammalian cells, we performed binding experiments with purified RidL, RidL_Δβ_ (lacking the Vps29-binding β-loop, Fig. 1A) and, as a negative control, the *L. pneumophila* effector SidC. Beads coupled with these baits were incubated with HeLa cell lysate and washed. Bound proteins were eluted, separated by SDS–PAGE, and dominant bands were identified by mass spectrometry (MS) (Figs. 1B and EV1B). Thus, the retromer coat complex subunit Vps35 was identified as an (indirect) binding partner of RidL, as observed previously (Finsel et al, 2013), and intriguingly, the large fission GTPase Drp1 was identified as a potential interaction partner of RidL_Δβ_. The binding of Drp1 in HeLa cell lysates to RidL_Δβ_ was validated by Western blot (Fig. 1C). To further test whether full-length RidL binds to Drp1 in cell lysates, we ectopically produced the effector in HeLa cells and performed pull-down experiments with an anti-RidL antibody and protein A/G magnetic beads (Fig. 1D). RidL was produced by the HeLa cells and retained by the beads. Under the conditions used, Drp1 was pulled down by the anti-RidL antibody, but not the closely related large fission GTPase dynamin-1 (Dnm1), both of which were detected in HeLa cell lysates (Fig. 1D). Taken together, RidL_Δβ_ (no longer interacting with the retromer) as well as wild-type RidL specifically bind to Drp1 in HeLa cell lysates.

To test whether and which portion of RidL directly binds to Drp1 in vitro, purified Drp1 was immobilized on beads and incubated with equal amounts of purified RidL, the N-terminal fragment RidL_N_ (RidL_1–258_), or the C-terminal fragment RidL_C_ (RidL_437–1100_) (Fig. 1E). The washed beads were subjected to SDS–PAGE, and bound proteins were visualized by silver stain (Fig. 1F) or anti-RidL Western blot (Figs. 1G and EV1C). Using these purified proteins, full-length RidL strongly bound to Drp1, RidL_C_ bound to some extent, and RidL_N_ did not bind to Drp1. Upon over-exposure of the Western blot, RidL_C_ showed binding to Drp1 while RidL_N_ did not bind at all (Fig. EV1C). In summary, purified Drp1 binds in vitro to RidL and a C-terminal fragment, RidL_C_ (RidL_437–1100_).

Given that RidL_Δβ_ (lacking the Vps29-binding β-loop) appears to bind Drp1 more strongly than RidL (Fig. 1B,C), we sought to assess whether Vps29 affects the interaction of Drp1 and RidL. To this end, we performed binding experiments with Drp1-coupled beads, incubated with purified RidL or RidL_Δβ_ in the absence or presence of purified Vps29 (Fig. 1H). Under the conditions used, Vps29 slightly but non-significantly reduced the binding of RidL to Drp1, while RidL_Δβ_ bound Drp1 more strongly than RidL in the absence as well as in the presence of up to four times excess Vps29 (Figs. 1H and EV1D). Accordingly, while RidL_Δβ_ binds Drp1 more strongly than RidL, Vps29 does not significantly interfere with the interaction.

To determine whether RidL affects the GTPase activity of Drp1, we used a coupled GTPase enzyme assay (Ingerman and Nunnari, 2005) (Fig. EV1E). Purified full-length RidL, RidL_N_ (RidL_1–258_), or RidL_C_ (RidL_437–1100_) was added to purified Drp1, and the GTPase activity was assessed. At 20× excess, RidL but neither RidL_N_ (RidL_1–258_) nor RidL_C_ (RidL_437–1100_) inhibited the GTPase activity (Fig. 1I). Upon adding increasing concentrations of purified RidL to purified Drp1, RidL inhibited the GTPase activity of wild-type Drp1 in a dose-dependent but inefficient manner (Figs. 1J and EV1F): at 20× excess, the activity was reduced by 39%. At 1–20× excess, RidL also reduced the GTPase activity of the assembly-deficient mutant, Drp1_A395D_ (Chang et al, 2010), by ~40%, indicating that the inhibitory effect was independent of GTPase oligomerization. Taken together, these results indicate that in vitro an excess of full-length RidL but not fragments thereof inhibits the GTPase activity of Drp1 and an oligomerization-deficient mutant protein.

### RidL impairs Drp1 oligomerization and spiralization

**Figure 2:**
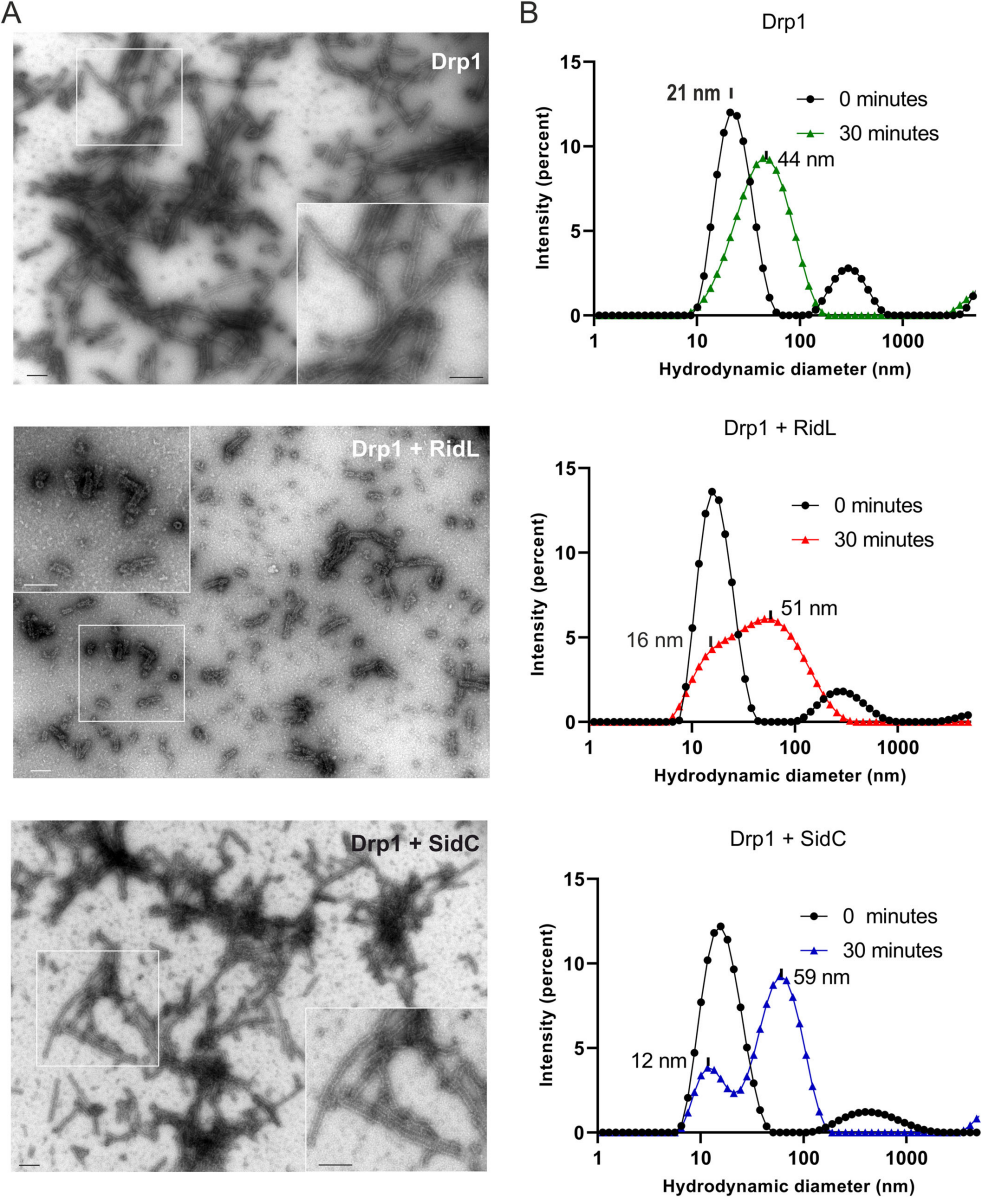
RidL inhibits Drp1 oligomerization and spiralization. (**A**) Purified Drp1 (5 µM) was treated with 0.5 mM GMPPCP, and RidL (15 µM), SidC (15 µM), or left untreated, and incubated for 1 h (room temperature) before staining. Oligomerization/spiralization was assessed by negative stain electron microscopy. Scale bars, 0.2 µm; inset 0.2 µm. (**B**) Purified Drp1 (1 µM) was incubated with 0.5 mM GMPPCP and treated with RidL (3 µM), SidC (3 µM), or left untreated, and Drp1 oligomerization was assessed by dynamic light scattering. Source data are available online for this figure.

Drp1 is a large fission GTPase, which—with complex dynamics—oligomerizes and forms spirals around membranes to constrict and sever membrane bilayers, leading to mitochondrial fragmentation as well as the release of MDVs (König et al, 2021; Smirnova et al, 2001). To assess the effects of RidL on the oligomerization and spiralization of Drp1, we employed negative staining electron microscopy (EM) (Fig. 2A). Using this approach, we first validated that upon treatment of purified Drp1 with the non-hydrolyzable GTP analog guanosine-5’-[(β,γ)-methyleno]triphosphate (GMPPCP), Drp1 oligomerizes and forms spirals (Ganesan et al, 2019; Kalia et al, 2018). Under the conditions used (0.5 mM GMPPCP), the majority of GMPPCP-treated Drp1 indeed oligomerized and formed spirals, which sometimes clustered and aligned in parallel. Intriguingly, the concomitant addition of excess RidL disrupted oligomerization and coiling of GMPPCP-treated Drp1, while the addition of purified SidC, an unrelated *L. pneumophila* effector of similar size, did not (Fig. 2A). Thus, RidL specifically impairs the oligomerization and spiralization of Drp1, which might contribute to the weak inhibition of Drp1 GTPase activity observed (Fig. 1I,J).

**Figure EV2:**
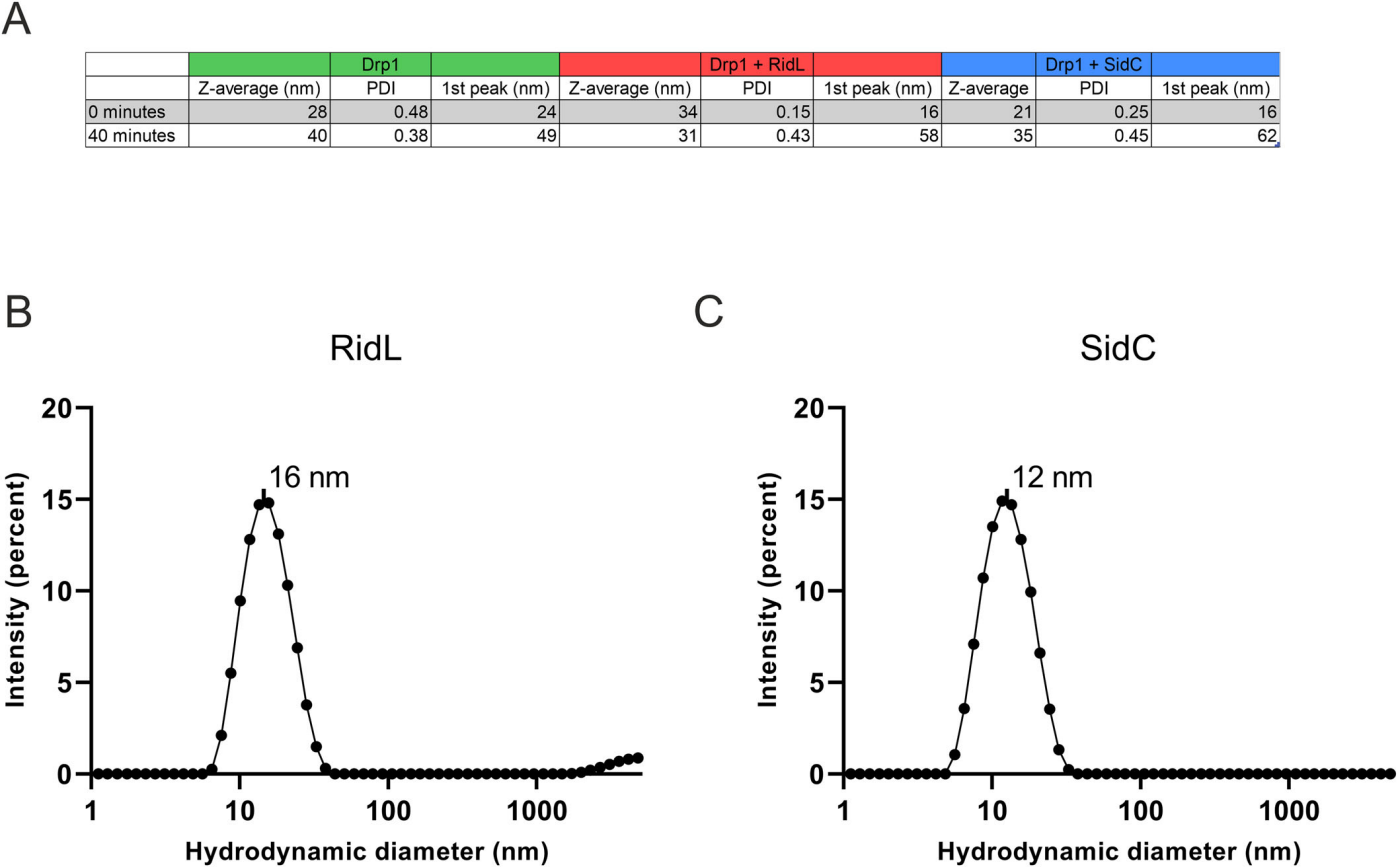
Dynamic light scattering values and profiles. (**A**) Hydrodynamic diameter (in terms of Z-average) and polydispersity index (PDI) from CUMULANT analysis, and mean value of the first distribution peak from CONTIN analysis of 1 µM Drp1 in the presence of 0.5 mM GMPPCP, in the absence (“Drp1”) or presence of 3 µM RidL (“Drp1 + RidL”) or 3 µM SidC (“Drp1 + SidC”). PDI, polydispersity index. Representative dynamic light scattering (DLS) profiles of (**B**) RidL (10 µM) or (**C**) SidC (10 µM) are shown (30 min).

Analogously, we assessed the effect of RidL (131 kDa) on the oligomerization of Drp1 (78 kDa) by dynamic light scattering (DLS) (Fig. EV2). Under the conditions used, RidL and the negative control SidC showed hydrodynamic radii of 16 and 12 nm, respectively (Fig. EV2BC). DLS indicated that upon treatment of purified Drp1 with 0.5 mM GMPPCP, the hydrodynamic diameter of Drp1 complexes increased (Fig. 2B). Prior to the addition of GMPPCP (0 min), the peak around 21 nm likely corresponds to the primarily dimeric form of Drp1 (156 kDa) and multiples thereof (Macdonald et al, 2014). Within 30 min of GMPPCP treatment, a complex oligomeric Drp1 population formed, which showed a symmetrical distribution peaking at a higher hydrodynamic diameter around 44 nm. Upon addition of excess RidL to GMPPCP-treated Drp1, a large population of complexes formed within 30 min, including the region around 16 nm associated with RidL alone (Fig. EV2B), followed by a broad shoulder and a minor peak at 51 nm (Fig. 2B). However, in the presence of Drp1, the discrete, symmetrical peak at 16 nm representing RidL alone was absent, suggesting that RidL interacts with Drp1. Based on the finding that RidL binds Drp1 (Fig. 1B,C), the broad peak around 51 nm likely shows a population of aggregated RidL-Drp1 complexes. Finally, upon addition of excess SidC (103 kDa), a size distribution with two symmetrical peaks was observed within 30 min (Fig. 2B), with the first peak at 12 nm associated with SidC (Fig. EV2C) and a second peak around 59 nm. Based on the finding that SidC does not bind Drp1 (Fig. 1B,C), the symmetrical peak around 59 nm might correspond to oligomerized Drp1, which seems to form even larger aggregates than Drp1 alone in the absence of SidC (44 nm, Fig. 2B). In presence of Drp1, SidC still shows a discrete peak at 12 nm corresponding to the effectors alone (Fig. EV2C), suggesting that SidC does not interact with Drp1. Overall, negative stain EM and DLS revealed that RidL but not SidC interacts with GMPPCP-treated Drp1 and impairs oligomerization and spiralization.

### RidL interacts with evolutionarily conserved large fission GTPases

**Figure 3:**
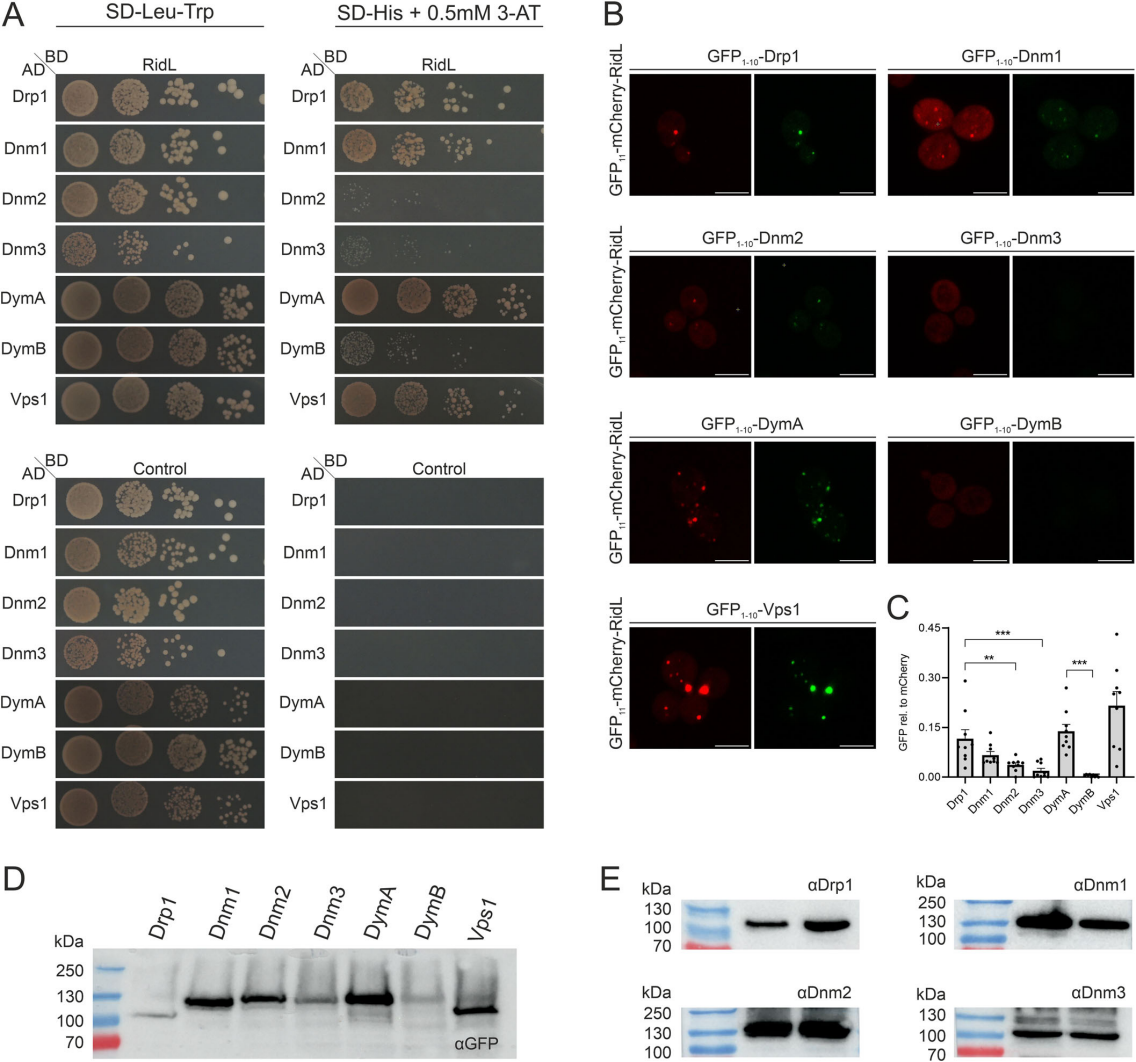
RidL interacts with evolutionarily conserved large fission GTPases. (**A**) Yeast two-hybrid assays using the reporter strain NMY32 producing *L. pneumophila* RidL fused to the LexA DNA-binding domain (BD) and large fission GTPases from human, *Dictyostelium discoideum* and yeast fused to the Gal4 activation domain (AD) of a split transcription factor required for His3 expression (negative controls: MTR4 fragment, lamin C). Yeast transformants were spotted in tenfold serial dilutions on SD plates lacking Leu and Trp (SD-Leu-Trp) or on SD-His plates with 0.5 mM 3-AT (3 d, 30 °C). Images are representative of three biological replicates, with each three tested yeast transformants per condition (*n* = 3). (**B**) Bimolecular fluorescence complementation (BiFC) in yeast strain BY4741 producing RidL fused to GFP_11_-mCherry, and large fission GTPases fused to GFP_1–10_. Aliquots of yeast transformants grown in selective medium were embedded in 0.1% agarose and imaged by confocal laser scanning microscopy. Scale bars, 5 µm. Images are representative of three biological replicates, with each three analyzed yeast transformants (*n* = 3). (**C**) Quantification of (**B**). Integrated densities of GFP and mCherry signals in merged z-stacks were calculated by ImageJ, and GFP signal intensities (representing RidL-GTPases interaction) were normalized to mCherry intensities representing overall protein production levels. Graphs show means + SEM of relative GFP intensities of three biological replicates with each three yeast transformants per condition (*n* = 3). Each dot represents the mean relative GFP intensity of one yeast transformant. All mammalian GTPases (Dnm1, Dnm2, and Dnm3) were compared to Drp1 by one-way ANOVA. *Dictyostelium discoideum* proteins DymA and DymB were compared to each other by Student’s *t*-tests (**p < 0.01; ***p < 0.001). If not indicated otherwise, the data are not significantly different. Production of large fission GTPases (Drp1, Dnm1, Dnm2, Dnm3, DymA, DymB, and Vps1) fused to GFP_1–10_ in yeast strain BY4741 (pKA100, pKA097, pKA098, pKA099, pKA114, pKA115, or pKA072) was validated by Western blot using (**D**) an anti-GFP antibody or (**E**) antibodies against Drp1, Dnm1, Dnm2, or Dnm3 (two different colonies are shown). The data shown is representative of three biological replicates (*n* = 3). Source data are available online for this figure.

RidL and other *L. pneumophila* effectors function in evolutionarily distant host cells and subvert conserved targets (Bärlocher et al, 2017; Finsel et al, 2013; Swart et al, 2018). To validate the binding of RidL to Drp1 and possibly identify additional eukaryotic large fission GTPases as target(s) of RidL, we employed yeast two-hybrid (Y2H) assays using RidL as the bait and large fission GTPases from mammalian cells, *Dictyostelium discoideum* amoeba and the yeast *Saccharomyces cerevisiae* as the prey (Fig. 3A). Using this approach, RidL was found to interact with large fission GTPases from mammalian cells (preferentially with Drp1 and Dnm1, less so with Dnm2 and Dnm3), *D. discoideum* (DymA, but not DymB) and *S. cerevisiae* (Vps1). Interestingly, among the family of dynamin-like large fission GTPases, Drp1, DymA, and Vps1 are the evolutionary most closely related enzymes (Katic et al, 2021).

As an alternative approach, we tested the interaction of RidL with large fission GTPases using confocal laser scanning microscopy and bimolecular fluorescence complementation (BiFC) in yeast. To this end, RidL was fused to GFP_11_-mCherry, and large fission GTPases were fused to GFP_1–10_ (Fig. 3B). RidL interacted and formed GFP-positive, punctate structures in the cells with Drp1 (and to a lesser extent with Dnm1), DymA and Vps1. Quantification of the interactions revealed the following intensity: Vps1 > DymA > Drp1 > Dnm1 > Dnm2 > Dnm3 > DymB, where the latter three fission GTPases did not (or only very weakly) interact with RidL (Fig. 3C).

The production in yeast of the large fission GTPases fused to GFP_1–10_ was validated by Western blot using an anti-GFP antibody (Fig. 3D) or specific antibodies against Drp1, Dnm1, Dnm2, or Dnm3, respectively (Fig. 3E). The Western blots using anti-GFP antibody indicated that all large GTPases are produced (Fig. 3D). The Western blots using specific antibodies revealed that Drp1, Dnm1, Dnm2, and Dnm3 are robustly produced at similar levels (Fig. 3E). In summary, RidL interacts most strongly with the closely related large fission GTPases Drp1, DymA, and Vps1, as well as with Dnm1, and the interactions observed are not due to different production levels of the large GTPases.

### RidL is associated with mitochondria and increases mitochondrial Drp1 and Tom20

**Figure 4:**
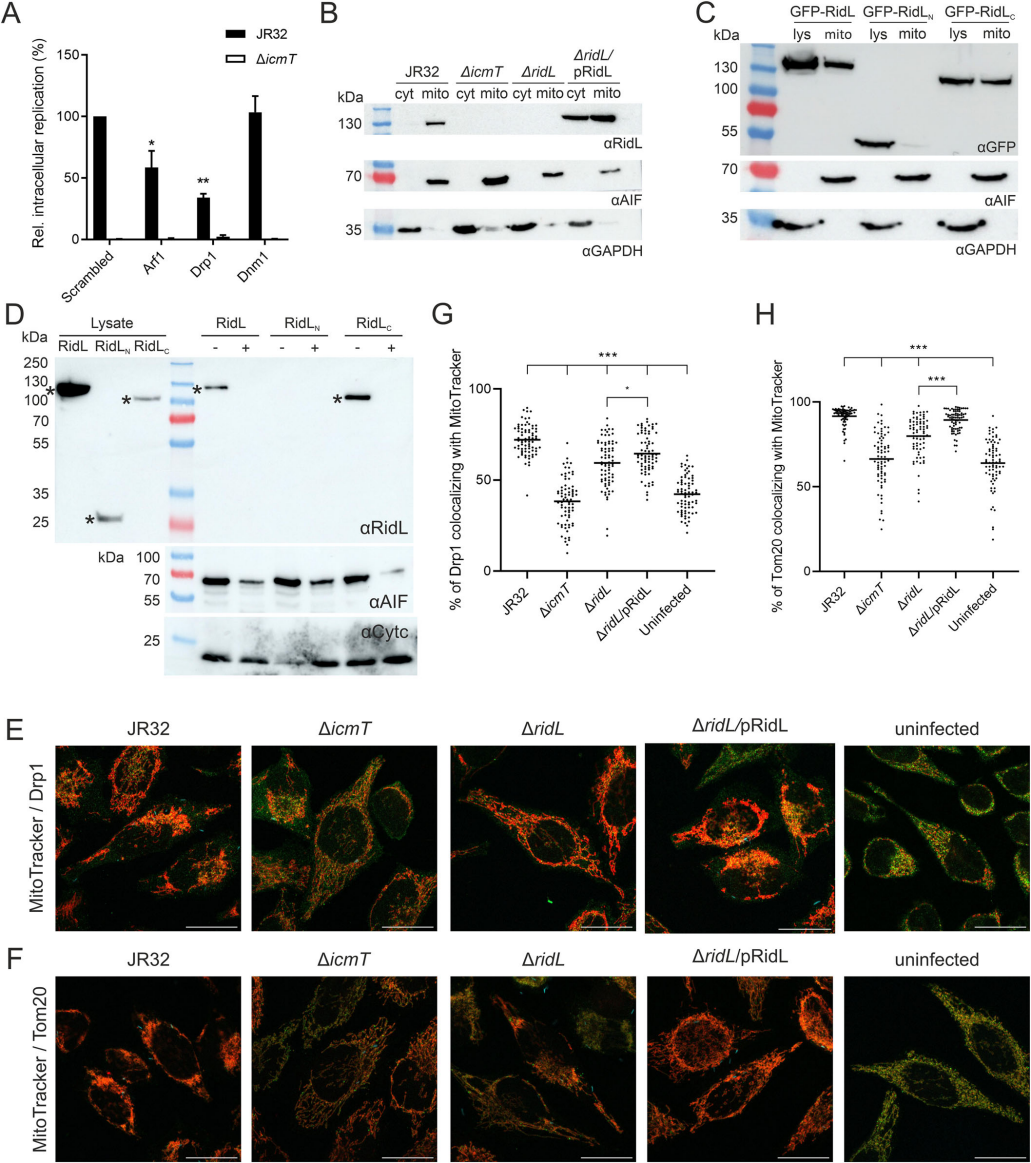
RidL is associated with mitochondria and increases mitochondrial Drp1 and Tom20. (**A**) A549 cells depleted (48 h) by siRNA for Arf, Drp1, or Dnm1 were infected (MOI 10, 24 h) with GFP-producing *L. pneumophila* JR32 or Δ*icmT* (pNT28), and intracellular replication was assessed by GFP fluorescence using a microtiter plate reader. Graphs show the relative intracellular replication (normalized to GFP signal 1 h p.i.) and represent means ± SEM of three biological replicates (*n* = 3) (one-way ANOVA, *p < 0.05; **p < 0.01). (**B**) Mitochondria were isolated by differential centrifugation and sucrose density gradient ultracentrifugation from HeLa cells infected (MOI 100, 6 h) with GFP-producing *L. pneumophila* JR32, *ΔicmT*, *ΔridL*, or *ΔridL*/pRidL (pNT28 or pIF009). Western blot of RidL in cytoplasmic (cyt) and purified mitochondrial (mito) fractions; apoptosis-inducing factor (AIF) and glyceraldehyde 3-phosphate dehydrogenase (GAPDH) served as a mitochondrial or cytoplasmic marker, respectively. (**C**) HEK293 cells were transfected (24 h) for ectopic production of codon-optimized GFP-RidL (RidL; pKB248), GFP-RidL_9–258_ (RidL_N_; pKB249) or GFP-RidL_259–1167_ (RidL_C_; pKB250), mitochondria were isolated, and the GFP fusion proteins were detected by anti-GFP Western blot in the cell lysate (lys) and purified mitochondrial (mito) fractions. (**D**) Mitochondria isolated from HeLa cells producing codon-optimized RidL_1-1167_ (RidL; pKB252), RidL_19–258_ (RidL_N_; pKB253), or RidL_259–1167_ (RidL_C_; pKB254) were incubated with proteinase K (50 µg/mL; “+”) or not (“−“), pelleted, resuspended in SDS sample buffer, and analyzed by Western blot using anti-RidL (“*”; RidL: 131 kDa, RidL_N_: 30 kDa, RidL_C_: 75 kDa), anti-Cytc or anti-AIF antibodies. The figures shown (**B**–**D**) are representative of three biological replicates (*n* = 3). (**E**,** F**) Fluorescence micrographs of HeLa cells infected (MOI 25, 2 h) with mCerulean-producing *L. pneumophila* JR32, Δ*icmT*, Δ*ridL*, or Δ*ridL*/pRidL (pNP99 or pKB208). Mitochondria were visualized with MitoTracker Deep Red, and immuno-labeled with specific antibodies against (**E**) Drp1 or (**F**) Tom20 (green) to quantify the localization of these proteins to mitochondria. Scale bars, 20 µm. (**G**) Quantification of Drp1 signal overlapping with MitoTracker (**E**) and (**H**) quantification of Tom20 signal overlapping with MitoTracker (**F**) show three biological replicates (*n* = 3) with each 25 analyzed cells (each dot is a cell; one-way ANOVA, ***p < 0.001). The brightness of fluorescence signals was linearly increased in ImageJ for enhanced visibility (**E**, **F**); original signal intensities of the images were processed for quantification (**G**, **H**). Source data are available online for this figure.

**Figure EV3:**
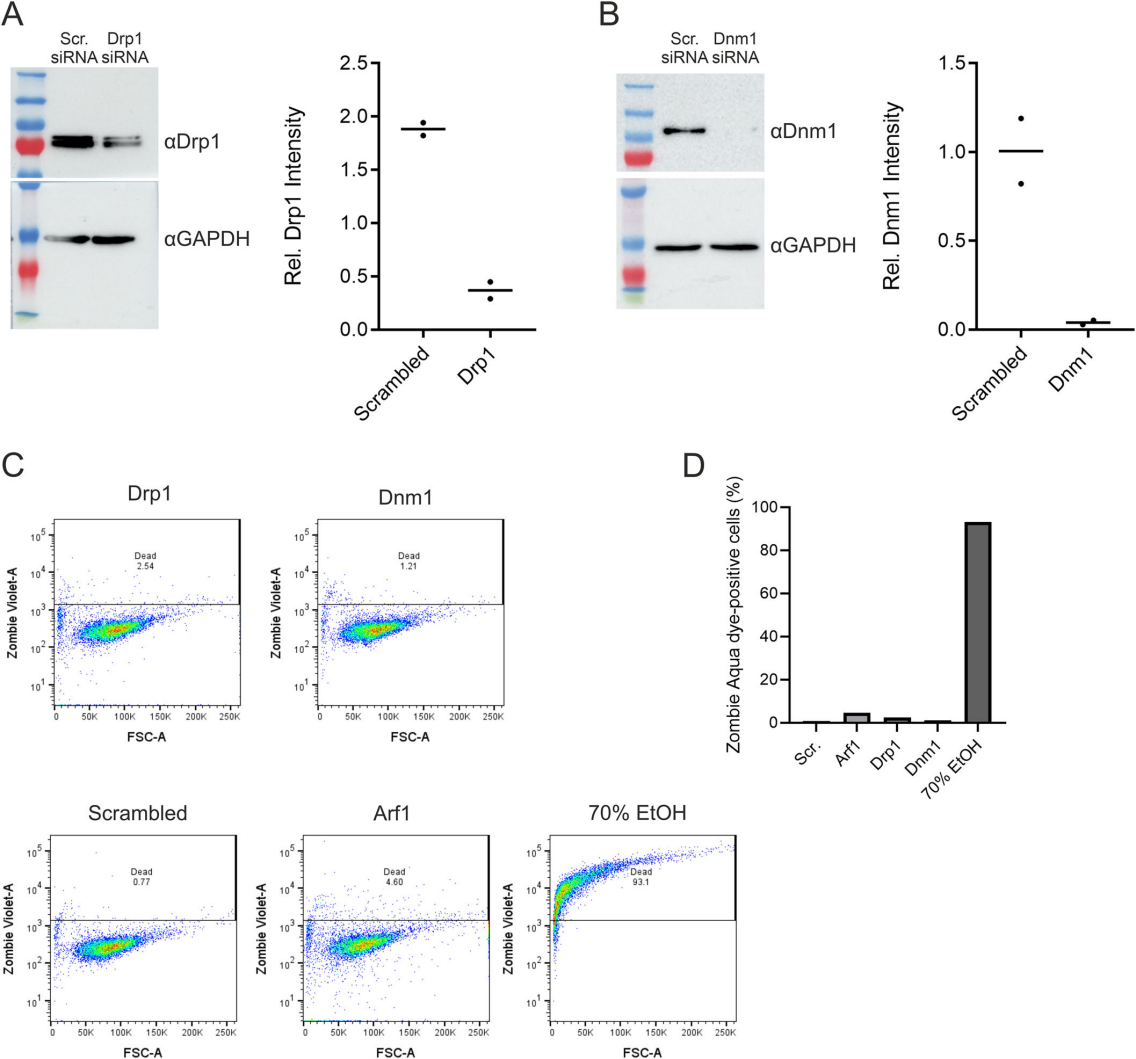
Drp1 but not Dnm1 affects intracellular replication of *L. pneumophila.* Efficiency of siRNA-mediated depletion of (**A**) Drp1 or (**B**) Dnm1 was assessed by Western blot (left panels). Quantification of the Drp1 or Dnm1 and GAPDH signal intensities of cells treated with Drp1- or Dnm1-specific or scrambled siRNA were calculated in ImageJ (right panels). Graphs show the Drp1 or Dnm1 signal intensities normalized to GAPDH from two biological replicates (*n* = 2). (**C**, **D**) Cytotoxicity was assessed in cells transfected with siRNA or treated with 70% EtOH (30 min, 37 °C) by staining with Zombie Aqua dye (1:500 dilution, 30 min). After staining, cells were fixed with 4% PFA and processed by flow cytometry with forward scatter (FSC) voltage 400, sideward scatter (SSC) voltage 250 and laser 525/50 (Vio510) voltage 380 (>10,000 events per sample). Data show one biological replicate (*n* = 1). (**C**) Scatter plots (indicated line shows the set threshold for live/dead cells) of FSC vs. Zombie Aqua dye fluorescence and (**D**) bar graph of Zombie Aqua dye-positive cells are shown.

To functionally validate the Y2H and BiFC interaction experiments, we assessed whether Drp1 or Dnm1 are implicated in intracellular replication of *L. pneumophila*. To this end, Drp1 or Dnm1 were depleted by RNA interference (RNAi) in A549 epithelial cells, and intracellular bacterial replication was assessed (Fig. 4A). Whereas the depletion of Drp1 impaired the intracellular replication of *L. pneumophila* as previously observed (Escoll et al, 2017), the depletion of Dnm1 had no effect. RNAi efficiently depleted the target proteins Drp1 and Dnm1 (Fig. EV3A,B) and was not cytotoxic for the cells (Fig. EV3C,D). Taken together, Drp1 but not Dnm1 promotes the intracellular growth of *L. pneumophila*, consistent with the observation that in mammalian cells RidL interacts with Drp1 and not with Dnm1 (Fig. 1D).

Given that Drp1 localizes to and is implicated in mitochondrial dynamics, we next checked whether RidL is associated with mitochondria in *L. pneumophila*-infected cells. To this end, HeLa cells were infected with the *L. pneumophila* parental strain JR32, the Δ*icmT* mutant strain lacking a functional T4SS, the Δ*ridL* mutant strain lacking RidL, or the complemented Δ*ridL* mutant. Mitochondria were isolated by differential centrifugation and sucrose density gradient ultracentrifugation, and RidL was detected by Western blot in cytoplasmic (cyt) and purified mitochondrial (mito) fractions (Fig. 4B). Upon infection with *L. pneumophila*, RidL was detected in the mitochondrial fractions isolated from the parental strain JR32 and the complemented *ΔridL* mutant strain, but not from the mitochondrial fractions of cells infected with the *ΔicmT* or *ΔridL* mutant strains. While RidL was only detectable in the mitochondrial fraction in cells infected with strain JR32, the effector localized both to the mitochondrial and the cytoplasmic fraction upon overproduction in the complemented Δ*ridL* mutant strain (Fig. 4B). These results indicate that RidL localizes to mitochondria in *L. pneumophila*-infected cells, and a functional Icm/Dot T4SS is required for host cell localization of the effector.

**Figure EV4:**
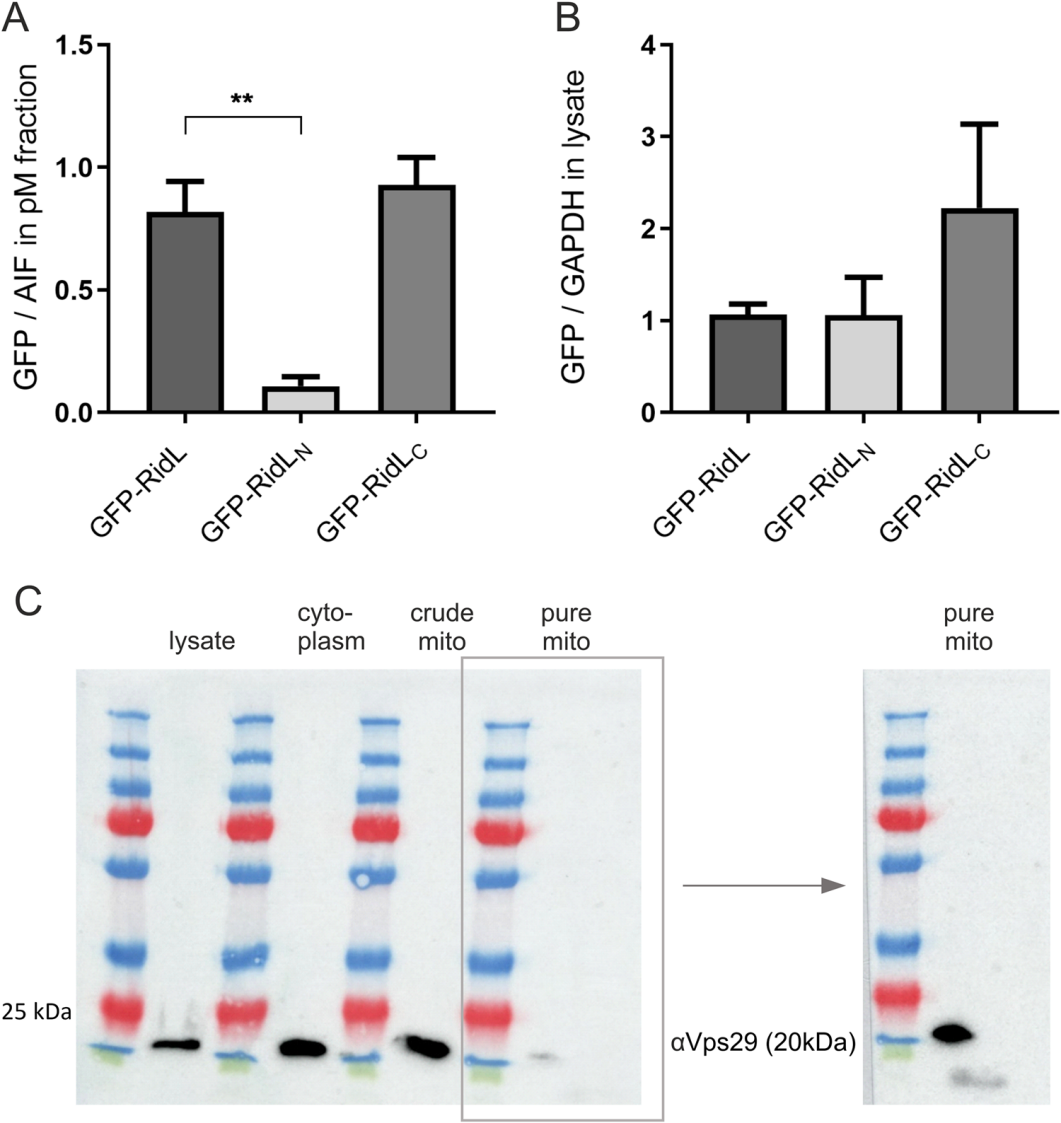
Localization of RidL and retromer. (**A**) Quantification of (Fig. 4C). Signal intensities of GFP and AIF were calculated by ImageJ. Bars show the relative GFP signal in pure mitochondria (pM) fractions. Means + SEM of three biological replicates are shown (one-way ANOVA, **p < 0.01). (**B**) Detection of GFP-RidL, GFP-RidL_N_, and GFP-RidL_C_ in HEK293 cell lysate (additional information for Fig. 4C). Signal intensities of GFP-RidL, GFP-RidL_N_, GFP-RidL_C_ and GAPDH in HeLa cell lysate were assessed by ImageJ. Graphs show the means + SEM of relative GFP intensities (GFP/GAPDH) of three biological replicates. If not indicated otherwise, the data were not significantly different. (**C**) Localization of the Vps29 subunit of the retromer cargo recognition subcomplex in HeLa cell lysates (lysate), cytoplasmic (cytoplasm), and crude/purified mitochondrial (crude/pure mito) fractions. Western blot with anti-VPS29 Antibody (sc-398874, Santa Cruz Biotechnology, 1:100) and secondary antibody goat anti-mouse IgG [H + L] HRP-linked secondary antibody (Invitrogen; 1:5000). Regular (left panel) and prolonged exposure (right panel) is shown.

To determine the portion of RidL that mediates its association to mitochondria, we transfected HEK293 cells with constructs for ectopic production of GFP-RidL, GFP-RidL_9–258_ (RidL_N_), or GFP-RidL_259–1167_ (RidL_C_). Mitochondria were isolated from the transfected cells, and the GFP fusion proteins were detected by anti-GFP Western blots in the cell lysate (lys) and purified mitochondrial (mito) fractions (Fig. 4C). Under these conditions, GFP-RidL and GFP-RidL_C_ but not GFP-RidL_N_ were significantly associated with purified mitochondria (Fig. EV4A), while all fragments were detected in similar amounts in the cell lysates (Fig. EV4B). Accordingly, the C-terminal, Drp1-binding fragment RidL_C_ determines the mitochondrial localization of the effector. In contrast, the N-terminal fragment RidL_N_, which binds the Vps29 subunit of the retromer cargo recognition subcomplex (Bärlocher et al, 2017), does not determine the mitochondrial localization, even though Vps29 is present on mitochondria (Fig. EV4C).

To further assess the mitochondrial compartment where RidL and RidL_C_ localize, mitochondria isolated from HeLa cells producing RidL, RidL_9–258_ (RidL_N_), or RidL_259–1167_ (RidL_C_) were incubated with proteinase K (Fig. 4D). Under the conditions used, RidL and RidL_C_ were completely digested, while the mitochondria-associated protein AIF displayed partial sensitivity to protease treatment, and the inner mitochondrial membrane protein cytochrome c was protease resistant (Fig. 4D). These findings indicate that RidL localizes to the outer mitochondrial membrane. In summary, our findings indicate that the retromer-binding effector RidL localizes through a C-terminal fragment to the outer mitochondrial membrane, likely through its interaction with Drp1.

The retromer complex and the large fission GTPase Drp1 localize to mitochondria and promote MDV formation (König and McBride, 2024). Given that Drp1 and Tom20 are MDV markers and cargo, respectively, we tested whether RidL affects the levels of these mitochondrial factors. To this end, HeLa cells were infected with the *L. pneumophila* parental strain JR32, Δ*icmT*, Δ*ridL*, or complemented Δ*ridL* mutant bacteria, and the mitochondrial levels of Drp1 (Fig. 4E; Appendix Fig. S1A) or Tom20 (Fig. 4F; Appendix Fig. S1B) relative to the total protein signal were assessed by immunofluorescence microscopy. This approach revealed that upon the presence and secretion of RidL, significantly higher portions of Drp1 (Fig. 4G) or Tom20 (Fig. 4H) localized to mitochondria, and the lower abundance of Drp1 and Tom20 upon infection with the Δ*ridL* mutant was significantly increased upon complementation of the mutant strain. Even less Drp1 or Tom20 localized to mitochondria upon infection of the cells with Δ*icmT* mutant bacteria. Taken together, in *L. pneumophila*-infected cells, RidL and a functional Icm/Dot T4SS increase the relative amounts of Drp1 and Tom20 on mitochondria.

### During *L. pneumophila* infection, RidL promotes fragmentation and impairs the function of mitochondria

**Figure 5:**
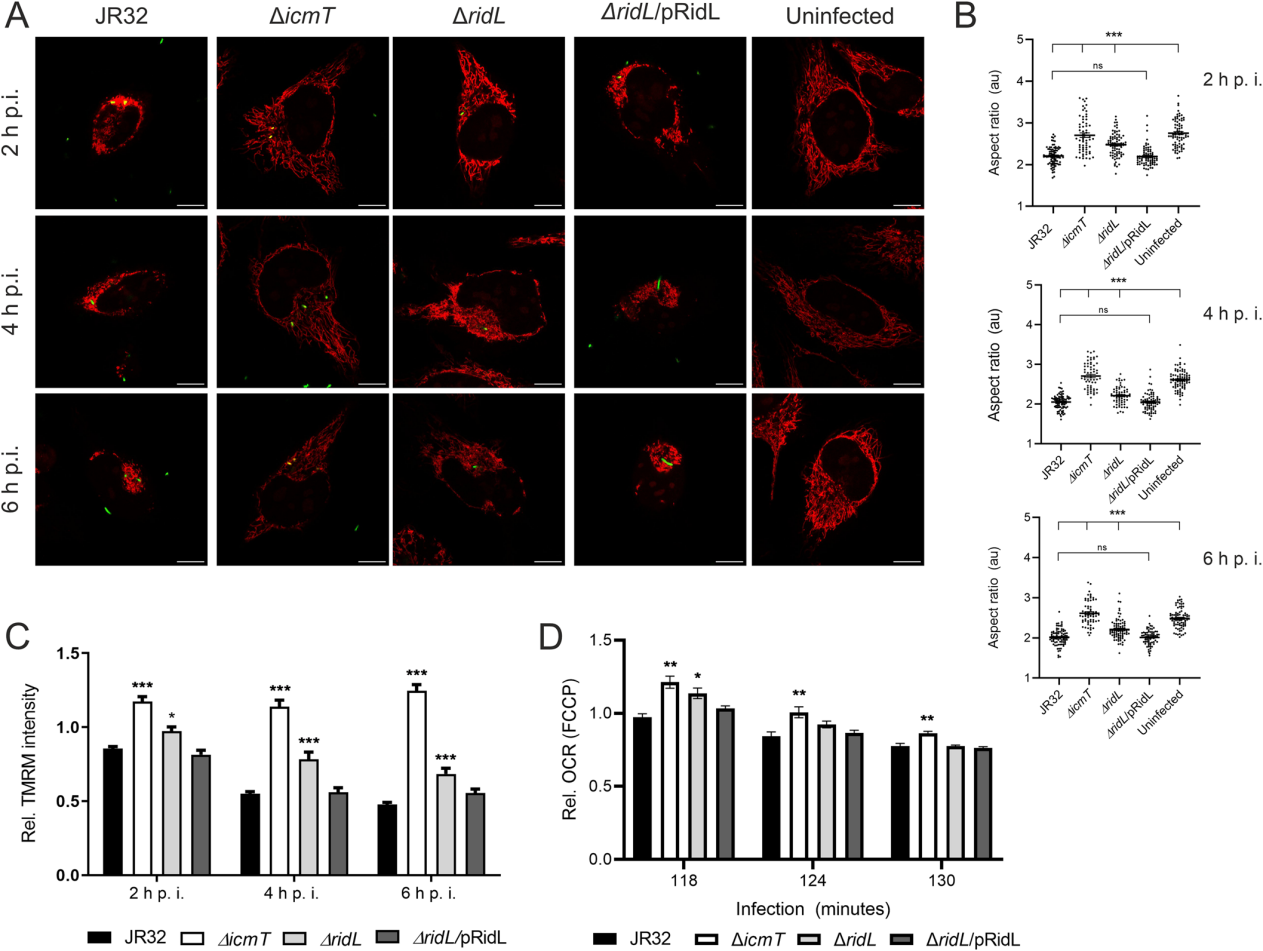
During *L. pneumophila* infection, RidL promotes fragmentation and impairs the function of mitochondria. (**A**) HeLa cells were stained with MitoTracker Orange CMTMRos (100 nM, 30 min, 37 °C) and infected (MOI 100, 2–6 h) with GFP-producing *L. pneumophila* JR32, *ΔicmT*, *ΔridL*, or *ΔridL*/pRidL (pNT28 or pIF009). Mitochondrial morphology was assessed by confocal laser scanning microscopy at the time points post infection (p.i.) indicated. Images are representative of 3 biological replicates (*n* = 3). Scale bars, 10 μm. (**B**) Quantification of (**A**). In infected cells, the mitochondrial aspect ratio (*d*_max_ / *d*_min)_ was assessed using the Mitochondria Analyzer plugin for ImageJ. Dots represent all analyzed cells from three biological replicates (*n* = 3) (20–30 cells each; means + SEM, one-way ANOVA, ***p < 0.001). (**C**) HeLa cells were infected (MOI 100, 2–6 h) with GFP-producing *L. pneumophila* JR32, *ΔicmT*, *ΔridL*, or *ΔridL*/pRidL (pNT28 or pIF009) and stained with the membrane potential probe tetramethyl rhodamine methyl ester (TMRM; 20 nM, 30 min, 37 °C). GFP and TMRM fluorescence were assessed by flow cytometry at the time points p.i. indicated. Graphs show means + SEM of TMRM intensities of infected cells relative to uninfected cells of nine samples from three biological replicates (*n* = 3) (>15.000 cells per sample; samples within each time point were compared to JR32-infected cells by one-way ANOVA, *p < 0.05; ***p < 0.001). (**D**) HeLa cells were infected (MOI 10) with *L. pneumophila* strains JR32, Δ*icmT*, Δ*ridL*, or Δ*ridL*/pRidL (pNT28 or pIF009), subjected to a mitochondrial stress test, and the oxygen consumption rate (OCR) was measured by a Seahorse XF Pro analyzer. Oligomycin (final concentration 1 μM), FCCP (0.5 μM) and rotenone/antimycin A (0.5 μM) were injected at the indicated time points (see Fig. EV5C). Graphs show relative OCR values upon injection of oligomycin and FCCP (normalized to OCR before mitochondrial stress test). Data taken from Fig. EV5C; means + SEM of three biological replicates (*n* = 3) (one-way ANOVA, *p < 0.05, **p < 0.01). If not indicated otherwise, the data are not significantly different. Source data are available online for this figure.

**Figure EV5:**
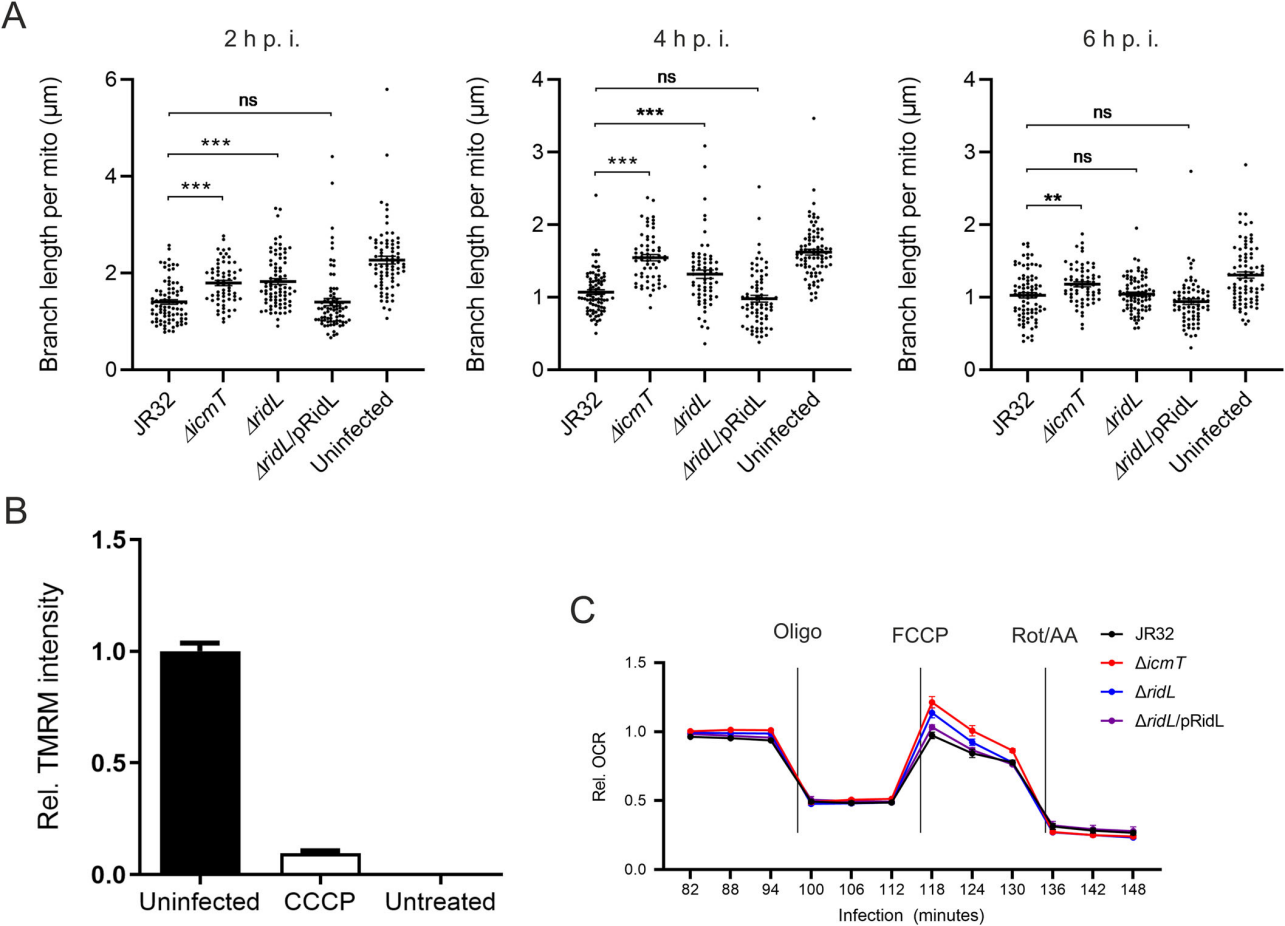
RidL modulates mitochondrial fragmentation and function in *L. pneumophila*-infected cells. (**A**) Mitochondrial branch lengths are quantified in infected cells (Fig. 5A). In infected cells, the average branch lengths per mitochondrion were assessed using the Mitochondria Analyzer plugin for ImageJ. Dots represent all analyzed cells from three biological replicates (*n* = 3) (20–30 cells each; means + SEM; one-way ANOVA, **p < 0.01; **p < 0.001; ****p < 0.0001). (**B**) Controls for TMRM assays shown in Fig. 5C. HeLa cells were treated with 50 μM CCCP (5 min, 37 °C). Uninfected and CCCP-treated cells were stained with tetramethyl rhodamine methyl ester (TMRM; 20 nM, 30 min, 37 °C), untreated cells were neither infected nor stained. TMRM intensities, reflecting the membrane potential, were assessed by flow cytometry with FSC voltage 420, SSC voltage 280 and YG610/20 voltage 400 (TMRM) (>10.000 events per sample). Data was processed in FlowJo and normalized to the average TMRM signal intensity of uninfected cells. Graphs show means + SEM of three biological replicates (*n* = 3; untreated mean: 0.000493). (**C**) Oxygen consumption of infected HeLa cells, as shown in Fig. 5D. Oxygen consumption rates (OCR) of uninfected HeLa cells were measured 2×. HeLa cells were infected (MOI 10) with *L. pneumophila* strains JR32, Δ*icmT*, Δ*ridL,* or Δ*ridL*/pRidL (pNT28 or pIF009), subjected to a mitochondrial stress test, and the OCR was measured every 6 min in 15 cycles (3:00 mix, 0:00 wait, 3:00 measure). Oligomycin (final concentration 1 μM), FCCP (0.5 μM) and rotenone/antimycin A (0.5 μM) were injected at the indicated time points. OCR values were normalized to the average OCR of the last two time points measured before the stress test. Graphs show the OCR during infection, normalized to OCR values before infection. Means + SEM of three biological replicates (*n* = 3).

The large fission GTPase Drp1 is a master regulator of mitochondrial dynamics (Chang and Blackstone, 2010; Giacomello et al, 2020). Given the localization of RidL to mitochondria and its role in mitochondrial Drp1 levels, we sought to assess whether the effector modulates mitochondrial dynamics and function. To this end, we stained HeLa cells with MitoTracker Orange, infected the cells with the parental strain JR32, Δ*icmT*, Δ*ridL*, or the complemented Δ*ridL* mutant strain, and visualized mitochondrial morphology by confocal microscopy (Fig. 5A). Compared to the *L. pneumophila* parental strain JR32, the mitochondrial aspect ratio (Fig. 5B) and branch length per mitochondrium (Fig. EV5A) were significantly increased upon infection with the Δ*ridL* strain, in particular at early time points post infection, and the effect was reversed upon infection with the complemented Δ*ridL* strain. RidL does not seem to account for the entire effect of mitochondrial fragmentation, since the mitochondria in Δ*icmT*-infected cells were still more elongated, as observed previously (Escoll et al, 2017). Taken together, these results indicate that the *L. pneumophila* effector protein RidL promotes mitochondrial fragmentation.

To assess the functional implications of RidL-dependent mitochondrial fragmentation, we tested the mitochondrial membrane potential and mitochondrial respiration (oxygen consumption rate) of *L. pneumophila*-infected HeLa cells. Along with mitochondrial fragmentation, RidL was also found to promote the dissipation of the mitochondrial membrane potential (Figs. 5C and EV5B). Compared to the wild-type strain, the Δ*ridL* mutant strain was significantly less effective in reducing the mitochondrial membrane potential of infected cells, and the complemented strain reached wild-type levels of membrane potential dissipation.

Mitochondrial respiration was impaired upon infection of HeLa cells with *L. pneumophila* wild-type, but less so with the Δ*ridL* mutant strain, and the complemented Δ*ridL* mutant strain reached wild-type levels of respiration inhibition (Figs. 5D and EV5C). This phenotype was particularly pronounced upon addition of the uncoupler carbonyl cyanide-p-trifluoromethoxyphenyl-hydrazone (FCCP) to assess maximal respiratory capacity. In contrast, upon infection of HeLa cells with the *L. pneumophila* Δ*icmT* mutant strain, the mitochondria were not fragmented, and neither the mitochondrial membrane potential nor respiration were affected. In summary, these results indicate that in *L*. *pneumophila*-infected cells, RidL contributes to mitochondrial fragmentation and impairs the mitochondrial membrane potential and respiration.

### RidL promotes phosphorylation-dependent activation of Drp1 in *L. pneumophila*-infected cells

**Figure 6:**
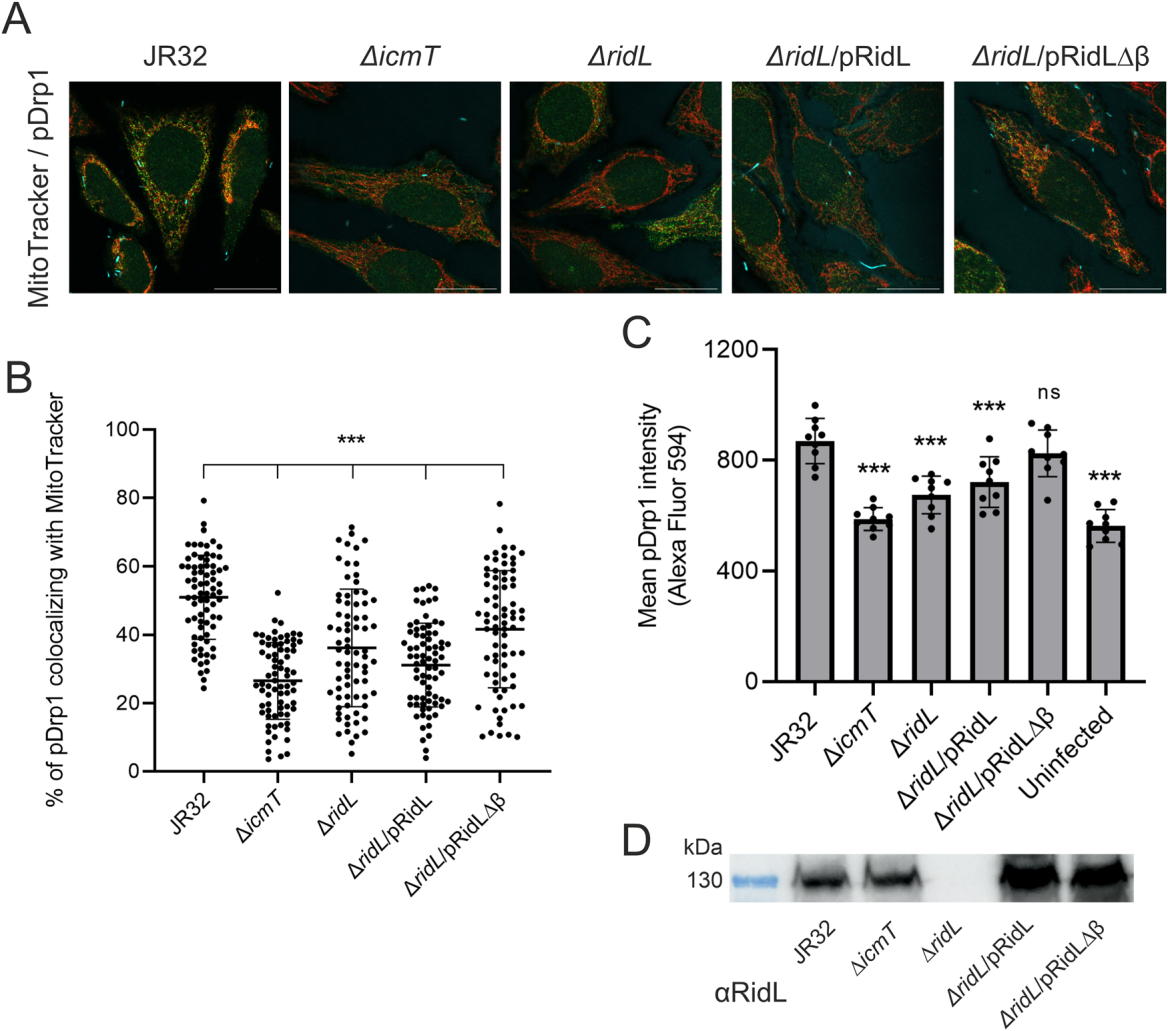
RidL promotes phosphorylation-dependent activation of Drp1 in *L. pneumophila*-infected cells. (**A**) Fluorescence microscopy of HeLa cells treated with MitoTracker Deep Red (30 min), infected (MOI 50, 2 h) with mCerulean-producing *L. pneumophila* strains JR32, Δ*icmT*, Δ*ridL*, Δ*ridL*/pRidL, or Δ*ridL*/pRidL_Δβ_ (pNP99, pKB208, or pKB209), and immuno-stained with an anti-phospho-Drp1 antibody (Ser616) followed by an Alexa Fluor 488-coupled secondary antibody. Signal intensities were linearly increased in ImageJ/ Fiji for enhanced visibility. Images are representative of three biological replicates (*n* = 3). Scale bars, 20 μm. (**B**) Quantification of colocalization of MitoTracker and pDrp1 (**A**) is shown for three biological replicates (*n* = 3) with each 25 analyzed cells (means and SD, one-way ANOVA, ***p < 0.001). (**C**) Flow cytometry of HeLa cells infected (MOI 50, 2 h) with GFP-producing *L. pneumophila* strains JR32, Δ*icmT*, Δ*ridL*, Δ*ridL*/pRidL, or Δ*ridL*/pRidL_Δβ_ (pNT28, pIF009, or pKB198), immuno-stained with an anti-phospho-Drp1 antibody (Ser616) followed by an Alexa Fluor 594-coupled secondary antibody. Means and standard deviation of three biological replicates with each three technical replicates (*n* = 3) are shown; each dot represents the mean Alexa Fluor 594 intensity of one technical replicate. All conditions compared to JR32 by one-way ANOVA (***p < 0.001). (**D**) Production of RidL was assessed by Western blot in equal numbers of *L. pneumophila* JR32, ∆*icmT*, ∆*ridL*, ∆*ridL*/pRidL, or ∆*ridL*/pRidL_∆β_ (pNT28, pIF009, or pKB198) grown to the stationary growth phase. Source data are available online for this figure.

Drp1 activity is regulated in a complex manner by posttranslational modifications, including activation by phosphorylation at Ser616 (Chang and Blackstone, 2010). Since RidL promotes mitochondrial fission and impairs mitochondrial functions, we hypothesized that RidL might activate Drp1, perhaps by promoting phosphorylation at Ser616. To test this hypothesis, we infected HeLa cells with mCerulean-producing *L. pneumophila* strain JR32, Δ*icmT*, Δ*ridL*, complemented Δ*ridL* mutant, or Δ*ridL* mutant producing RidL_Δβ_ (lacking the retromer-interacting β-loop) and performed confocal microscopy using a phospho-Ser616-specific anti-Drp1 antibody (Fig. 6A; Appendix Fig. S2). This approach revealed that upon infection with *L. pneumophila* wild-type, ~50% of the detected phospho-Drp1 localized with the mitochondrial stain MitoTracker, while this was the case for only 20–30% of the detected phospho-Drp1 upon infection with *L. pneumophila* Δ*icmT* or Δ*ridL*, respectively (Fig. 6B). Plasmid-produced RidL or RidL_Δβ_ did not restore the colocalization of phospho-Drp1 and MitoTracker.

To validate these results, we performed analogous experiments using flow cytometry and staining for phospho-Drp1 (Fig. 6C). To this end, HeLa cells were infected with GFP-producing *L. pneumophila* strain JR32, Δ*icmT*, Δ*ridL*, a complemented Δ*ridL* mutant, or Δ*ridL* mutant producing RidL_Δβ_ (lacking the retromer-interacting β-loop), fixed and analyzed by flow cytometry. This approach indicated that compared to cells infected with *L. pneumophila* wild-type, the mean fluorescence intensity of cells infected with *L. pneumophila* Δ*icmT* or Δ*ridL* was significantly lower (Fig. 6C). The phenotype was complemented by supplying plasmid-produced RidL_Δβ_ and—less efficiently—by wild-type RidL. As a control, we assessed the production of RidL by different *L. pneumophila* strains and found that the complemented ∆*ridL* mutant strains produced the effector protein at even higher levels than the wild-type strain JR32 (Fig. 6D). Taken together, these results revealed that RidL promotes the activating phosphorylation of Drp1 at the amino acid residue Ser616. Intriguingly, this activity was observed for RidL as well as for RidL_Δβ_, indicating that binding to the retromer is not required for Drp1 activation.

## Discussion

In the present study we show that the *L. pneumophila* effector protein RidL binds through a C-terminal fragment to the large dynamin-like mitochondrial fission GTPase Drp1 (Fig. 1). In vitro, RidL impairs GTPase activity (Fig. 1) and inhibits Drp1 oligomerization and spiralization (Fig. 2). Upon ectopic production in yeast, RidL interacts with several closely related and evolutionary conserved large fission GTPases (Fig. 3). Finally, in *L. pneumophila*-infected host cells, RidL associates with mitochondria and increases the relative levels of mitochondrial Drp1 as well as Tom20 (Fig. 4), promotes mitochondrial fragmentation and impairs mitochondrial function (Fig. 5), and upregulates phosphorylation of Drp1 (Fig. 6). While the N-terminal fragment of RidL has been characterized to interact with the Vps29 subunit of the cargo recognition subunit of the retromer coat complex, thereby displacing the Rab7 GAP TBC1D5 (Bärlocher et al, 2017; Finsel et al, 2013; Romano-Moreno et al, 2017; Yao et al, 2018), the interactor(s) and function of the C-terminal fragment of RidL were unknown. Here we reveal that the C-terminal fragment of RidL binds to Drp1 in vitro (Fig. 1F,G) and localizes to mitochondria upon ectopic production (Figs. 4C,D and EV4A).

Drp1 is the major fission GTPase promoting mitochondrial fragmentation (Giacomello et al, 2020). Upon secretion of RidL in *L. pneumophila*-infected cells, significantly higher portions of Drp1 (Fig. 4E,G) or Tom20 (Fig. 4F,H) localized to mitochondria. The mechanism underlying the RidL-dependent mitochondrial fragmentation likely involves the activation of Drp1 by Ser616 phosphorylation (Fig. 6). Activation phosphorylation might either occur in the cytoplasm, followed by recruitment of activated Drp1 to mitochondria, or by phosphorylation of mitochondria-bound Drp1. Several eukaryotic kinases modulate the activity of Drp1 (Chang and Blackstone, 2010; Giacomello et al, 2020), but the kinase activating Drp1 in the context of *L. pneumophila* infection has not been identified. Moreover, neither bioinformatics nor experimental evidence was obtained that RidL itself is a kinase. Our results are in agreement with the notion that RidL might either recruit Drp1 to mitochondria and/or prevent the removal/turnover of Drp1 from mitochondria.

Alternatively, or additionally to the direct mitochondrial recruitment or stabilization of Drp1 or phospho-Drp1, the mechanism underlying RidL-dependent mitochondrial fragmentation might involve the modulation of Drp1 removal/turnover via MDV formation. Mitochondrial protein turnover occurs—at least partly—through the formation of MDVs (König and McBride, 2024). While the mitochondrial recruitment of Drp1, oligomerization, and membrane constriction do not involve the retromer coat complex, Drp1 is indeed an MDV marker in a pathway implicating the retromer (Wang et al, 2017; Wang et al, 2016). Furthermore, Drp1 is also recruited to the sites of nascent MDVs and promotes MDV fission (König et al, 2021). RidL inhibits the retromer in endosome-derived retrograde trafficking (Bärlocher et al, 2017; Finsel et al, 2013), and analogously, RidL might inhibit the (initial step of) MDV formation and removal of (inactive) Drp1 via this route. However, RidL promotes the accumulation of Drp1 and phospho-Drp1 on mitochondria (Figs. 5 and 6), and therefore, the (final steps of) MDV formation, i.e., the fission of MDVs from mitochondria, might be facilitated. Future studies will shed light on whether and how MDV formation is implicated in RidL-mediated mitochondrial fragmentation. Our study addresses the complex interplay between the retromer and Drp1, mitochondrial dynamics and MDV trafficking. In *L. pneumophila*-infected cells, RidL promotes the mitochondrial accumulation of not only Drp1 (Fig. 4E,G) but also Tom20 (Fig. 4F,H). Accordingly, RidL might interfere with the general protein turnover and quality control of mitochondria. Further studies will explore the role of RidL for MDV trafficking in infection biology and will also use RidL as a tool to dissect the role of MDV trafficking in cell biology, neurodegeneration and cancer.

Intriguingly, RidL is not the only *L. pneumophila* effector protein possibly targeting the MDV pathway. The Ran GTPase activator LegG1/MitF stabilizes microtubules (Rothmeier et al, 2013; Swart et al, 2020), likely accounting for its effect on promoting mitochondrial fragmentation (Escoll et al, 2017) and the microtubule-dependent formation of mitochondria-derived tubules (König and McBride, 2024). Moreover, the effector Lpg1137 cleaves the SNARE syntaxin 17 (Arasaki et al, 2017), thereby possibly inhibiting the final step of MDV fusion with lysosomes (McLelland et al, 2016). Several other *L. pneumophila* effectors have been shown to target mitochondria, such as the nucleotide carrier protein LncP (Dolezal et al, 2012), or the ADP/ATP translocase ADP-ribosyltransferase Lpg0080/Ceg3 and the antagonist hydrolase Lpg0081 (Fu et al, 2022; Kubori et al, 2022). In summary, *L. pneumophila* seems to adopt a “double-hit” strategy to impair mitochondrial dynamics and function. The pathogen not only directly targets mitochondrial components but might also interfere with their turnover.

*L. pneumophila* promotes the accumulation of activated phospho-Drp1 to mitochondria in a RidL-dependent manner in infected cells (Fig. 6A,B). Intriguingly, the corresponding phenotype of the Δ*ridL* mutant strain was complemented by supplying plasmid-produced RidL_Δβ_ or—less efficiently—wild-type RidL (Fig. 6C). This finding suggests that the RidL-dependent recruitment of pDrp1 to mitochondria does not require the retromer. Moreover, the localization of RidL to mitochondria occurs through the Drp1-binding C-terminal fragment rather than through the retromer-binding N-terminal fragment (Fig. 4C).

While RidL promotes the phosphorylation (and presumably) activation of Drp1 in infected cells, an excess of full-length RidL but not the C- or N-terminal fragment impairs GTPase activity in vitro (Fig. 1J). This apparent discrepancy likely reflects the absence of any kinase in the in vitro assay. Moreover, RidL inhibits Drp1 oligomerization and spiralization in vitro (Fig. 2). The DLS experiments support the notion that RidL interacts with Drp1 and interferes with its oligomerization, since RidL but not SidC, suppresses the emergence of a symmetrical peak at a high hydrodynamic radius of 44 or 59 nm, respectively, and instead, the addition of RidL but not SidC to Drp1 results in a broad distribution of hydrodynamic radii between 16–51 nm (Fig. 2B). Moreover, in presence of Drp1, RidL no longer shows a discrete symmetrical peak at 16 nm (Fig. EV2B), while SidC still shows the symmetrical peak at 12 nm of SidC alone (Fig. EV2C), indicating binding to Drp1 of the former but not the latter. As a caveat, the EM and DLS experiments were performed in the absence of the (unknown) kinase promoting the phosphorylation of Drp1, and therefore, must be interpreted cautiously.

RidL_Δβ_, lacking the Vps29-binding β-loop, as well as wild-type RidL, bound Drp1 in lysates of HeLa cells (Fig. 1B–D). However, RidL_Δβ_ interacted with Drp1 more strongly than RidL (Figs. 1H, and EV1D), and full-length RidL bound Drp1 more strongly than the C-terminal fragment RidL_C_ (RidL_437–1100_) (Fig. 1F,G). Accordingly, the Vps29-binding β-loop and the N-terminal fragment of RidL seem to play a role in the interaction of the effector with the large GTPase. Perhaps full-length RidL interacts with oligomeric Drp1, while the C-terminal fragment of RidL does not. Moreover, it is unclear whether and how the interaction of RidL with Vps29 and/or the entire retromer cargo recognition complex affects its binding to Drp1. In agreement with a mutual dependency of different target protein interactions, the predicted structure of RidL comprises the Vps29-binding “foot” and “leg” domains, linked by an apparently flexible hinge to the Drp1-binding “arm” and “fist” domains (Fig. 1A). Intriguingly, RidL_Δβ_ promotes the accumulation of phospho-Drp1 on mitochondria, and therefore, binding to the retromer seems dispensable for this activity (Fig. 6). Finally, the presence of a lipid bilayer membrane likely also plays an important role for the Vps29-RidL-Drp1 interactions. Of note, RidL binds to the phosphoinositide lipid PtdIns(3)*P* (Finsel et al, 2013), which might also affect the interactions among the effector, Vps29 and Drp1. Further mechanistic studies will address in detail these protein–protein and protein–lipid interactions.

*L. pneumophila* invests considerable resources to subvert mitochondria. An efficient inhibition of mitochondrial function likely provides an advantage for the strictly aerobic pathogen regarding the competition between mitochondria and the bacteria for intracellularly available nutrients. In agreement with this notion, cellular lipid droplets associate with LCVs and are used by *L. pneumophila* as a source of catabolic fatty acids (Hüsler et al, 2023a; Hüsler et al, 2023b; Hüsler et al, 2021). Further studies will address the intricate (competitive) relationship between *L. pneumophila* and mitochondria.

## Methods

**Table:** Reagents and tools table

Reagent/resource	Reference or source	Identifier or catalog number
**Experimental models**		
*E. coli* BL21 (DE3)	Novagen	
*E. coli* MC1061	Casadaban and Cohen, 1980	
*E. coli* TOP10	Invitrogen	
*L. pneumophila* (Δ*ridL*)	Finsel et al, 2013	CR06
*L. pneumophilla (*Δ*icmT)*	Segal and Shuman, 1998	GS3011
*L. pneumophila* (wild-type)	Sadosky et al, 1993	JR32
Yeast BY4741	Panse lab collection	
Yeast NMY32	Dualsystems Biotech	
HeLa cells	ATCC	
HEK293 cells	ATCC	
A549 cells	ATCC	
**Recombinant DNA**		
Yeast two-hybrid control	Panse lab collection	pAct2.2-large T
N-terminal 3C protease cleavable His_10_-tag, pBAD, Amp^R^	Geertsma and Dutzler, 2011	pBXNH3
pET28(+)-*sidC* (His_6_-SidC)	Weber et al, 2006	pCR001
Drp1 (human)	Addgene #49152, Friedman et al, 2011	mCh-Drp1
Drp1 (murine)	Addgene #72929, Liu and Chan, 2015	pET21-Drp1
pET21-Drp1_A395D_ (human)	This work	pEV001
Mammalian expression vector, P_*CMV*_, Neo^R^, Kan^R^	Clontech Laboratories	pEGFP-C1
Mammalian expression vector, P_*CMV*_, Neo^R^, Kan^R^	Clontech Laboratories	pEGFP-N1
Dynamin-eGFP	Addgene #120313, Kong et al, 2018	pEGFP-N1-dynamin
pMMB207C-RBS-*gfp*-P_*ridL*_-RidL (constitutive GFP)	Finsel et al, 2013	pIF009
pLexA-RidL	This work	pKA001
pAct2.2-Vps1 (yeast)	This work	pKA004
pAct2.2-Drp1 (human)	This work	pKA009
pAct2.2-Dnm1 (human)	This work	pKA103
pAct2.2-Dnm2 (human)	This work	pKA011
pAct2.2-Dnm3 (human)	This work	pKA104
pAct2.2-DymA (*D. discoideum*)	This work	pKA014
pAct2.2-DymB (*D. discoideum*)	This work	pKA015
pRS315_Nop1pr_GFP_11_-mCherry-RidL	This work	pKA067
pRS316_Nop1pr_GFP_1–10_-Vps1	This work	pKA072
pRS316_Nop1pr_GFP_1–10_-Dnm1	This work	pKA097
pRS316_Nop1pr_GFP_1–10_-Dnm2	This work	pKA098
pRS316_Nop1pr_GFP_1–10_-Dnm3	This work	pKA099
pRS316_Nop1pr_GFP_1–10_-Drp1	This work	pKA100
pGEM-T Easy_NdeI-DymB-BamHI	This work	pKA105
pRS316_Nop1pr_GFP_1–10_-DymA	This work	pKA114
pRS316_Nop1pr_GFP_1–10_-DymB	This work	pKA115
pGEM-T-Easy_DymB	This work	pKA117
pRS316_Nop1pr_GFP_1–10_-AgeI	This work	pKA118
YEp351tet-Drp1	This work	pKA080
pET21-Drp1 (human)	This work	pKA173
pINIT-RidL	Bärlocher et al, 2017	pKB002
pBXC3GH_RidL	Bärlocher et al, 2017	pKB016
pBXC3GH_hVps29	Bärlocher et al, 2017	pKB024
pBXNH3_RidL_1–258_	This work	pKB139
pInit_RidL_∆β_	Bärlocher et al, 2017	pKB151
pMMB207C-GFP-P_*ridL*_-RidL_∆β_	This work	pKB198
pMMB207C-mCerulean-MCS (constitutive mCerulean)	This work	pKB207
pMMB207C-mCerulean-P_*ridL*_-RidL (constitutive mCerulean)	This work	pKB208
pMMB207C-mCerulean-P_*ridL*_-RidL_∆β_ (constitutive mCerulean)	This work	pKB209
pINIT-Dnm2 (PCR template)	This work	pKB241
pINIT-Vps1 (PCR template)	This work	pKB242
pEGFP-N1_RidL^opt^ (PCR template)	This work	pKB245
pEGFP-C1_RidL^opt^-STOP (eGFP-RidL)	This work	pKB248
pEGFP-C1_RidL^opt^_9–258_-STOP	This work	pKB249
pEGFP-C1_RidL^opt^_259–1167_-STOP	This work	pKB250
pEGFP-N1_RidL^opt^_1–1167_-STOP (RidL)	This work	pKB252
pEGFP-N1_RidL^opt^_9–258_-STOP	This work	pKB253
pEGFP-N1_RidL^opt^_259–1167_-STOP	This work	pKB254
pBXC3GH-RidL_437–1100_	This work	pKB306
Yeas two-hybrid control	Panse lab collection	pLexA_Lamin C
pMMB207C, Δ*lacI*^q^ (constitutive *gfp*), Cam^R^	Tiaden et al, 2007	pNT28
pNT28-MCS	Tiaden et al, 2007	pNT29
pMMB207C, Δ*lacI*^q^ (constitutive mCerulean), Cam^R^	Steiner et al, 2017	pNP99
pRS315-NOP1pr-GFP_11_-mCherry-PUS1	Addgene #86413, Smoyer et al, 2016	pSJ1321
pRS316-NOP1pr-GFP_1–10_-SCS2TM	Addgene #86418, Smoyer et al, 2016	pSJ2039
**Antibodies**		
RidL (rabbit) polyclonal, 1:5000	Finsel et al, 2013	
Drp1 (rabbit) monoclonal, 1:1000	Cell Signaling Technology	#5391
Drp1 (mouse) monoclonal, 1:1000	Abcam	ab56788
AIF (rabbit) polyclonal, 1:5000	Proteintech	17984-1-AP
Cytochrome C polyclonal, (rabbit) 1:1000	Proteintech	10993-1-AP
GAPDH (rabbit) monoclonal, 1:1000	Cell Signaling Technology	#2118
Vps29 (mouse) monoclonal, 1:100	Santa Cruz Biotechnology	#sc-398874
Dnm1 (rabbit) monoclonal, 1:1000	Proteintech	#68009-1-Ig
Dnm2 (rabbit) polyclonal, 1:1000	Abcam	ab3457
Dnm3 (rabbit) polyclonal, 1:1000	Thermo Fisher	PA1-662
GFP Living Colors JL-8 (mouse) monoclonal, 1:5000	Clontech	#632380
GFP polyclonal, 1:2000	Thermo Fisher	A-11122
Tom20 (rabbit) polyclonal, 1:1000	Proteintech	11802-1-AP
Donkey anti-rabbit, 1:5000	Cytiva/Amersham	#NA934-1ML
Goat anti-mouse, 1:5000	Invitrogen	#31430
Sheep anti-mouse, 1:5000	Cytiva/Amersham	#NA931-1ML
Drp1 (C-terminal) (rabbit) polyclonal, 1:50	Proteintech	#12957-1-AP
Phospho-Drp1 (Ser616) (rabbit) monoclonal, 1:1000	Cell Signaling Technology	#4494
Chicken anti-rabbit, 1:5000	Aves Lab Inc.	H-1004
Goat anti-Rabbit IgG (H + L) Cross-Adsorbed ReadyProbes Secondary Antibody, Alexa Fluor 594	Thermo Fisher	R37117
Chicken anti-Rabbit IgG (H + L) Cross-Adsorbed Secondary Antibody, Alexa Fluor 488, 1:250	Thermo Fisher	#A-21441
F(ab’)2-Goat anti-Rabbit IgG (H + L) Cross-Adsorbed Secondary Antibody, Alexa Fluor 488	Thermo Fisher	#A-11070
F(ab’)2-goat anti-rabbit, 1:5000	Thermo Fisher	A24531
**Oligonucleotides and other sequence-based reagents**		
TAAGCAGGATCCGGATGATTCTCGAGGAGTAC	This work	oKA001
TAAGCAGAATTCATGGATGAGCATTTAATT	This work	oKA004
TGCTTAGTCGACCTAAACAGAGGAGACGAT	This work	oKA005
TAAGCACATATGATGGAGGCGCTAATTCC	This work	oKA022
TGCTTAGGATCCCTCACCAAAGATGAGTCTC	This work	oKA023
TAAGCACATATGATGGGCAACCGCGGG	This work	oKA025
TGCTTAGAATTCGCTAGTCGAGCAGGGATGG	This work	oKA026
TAAGCACATATGATGGATCAATTAATC	This work	oKA029
TGCTTAGAATTCCTTAATTTCTAAAATCTCT	This work	oKA030
TGCTTAGTCGACTTACTTACGCATCCCTGT	This work	oKA051
TAAGCAGCTAGCATGATTCTCGAGGAGTACA	This work	oKA183
TTAATTAAGCGAATTTCTTATGATTTATGA	This work	oKA207
TTCATTTTGGGCCCCAGA	This work	oKA208
**TGGGGCCCAAAATGAA**ATGGATGAGCATTTAATTTC	This work	oKA209
**TCATAAGAAATTCGCTTAATTAA**CTAAACAGAGGAGACGAT	This work	oKA210
TAAGCAAGATCTTTAATTAAGCGAATTTCTTATGATTTATGA	This work	oKA224
TGCTTAAGATCTCTATTCATTTTGGGCCCCAGA	This work	oKA225
**TGGGGCCCAAAATGAA**ATGGGCAACCGCGGCATG	This work	oKA239
**TCATAAGAAATTCGCTTAATTAA**TTAGAGGTCGAAGGGGGGCCTGGG	This work	oKA240
**TGGGGCCCAAAATGAA**ATGGGCAACCGCGGG	This work	oKA241
**TCATAAGAAATTCGCTTAATTAA**CTAGTCGAGCAGGGATGG	This work	oKA242
**TGGGGCCCAAAATGAA**ATGGGGAACCGGGAG	This work	oKA243
**TCATAAGAAATTCGCTTAATTAA**TTAGTCTAACAGGGAGGATTCTAG	This work	oKA244
**TGGGGCCCAAAATGAA**ATGGAGGCGCTAATTCC	This work	oKA245
**TCATAAGAAATTCGCTTAATTA**TCACCAAAGATGAGTCTC	This work	oKA246
ATATATCATATGAAAATGTTGAGTTCAACAGCAATATTAAAG	This work	oKA263
ATATATGGATCCTTAATACAAATGAAGTAAATCACTATTTTCAGATTG	This work	oKA264
**CTGGGGCCCAAAATGAA**ATGGATCAATTAATCCC	This work	oKA280
**TCATAAATCATAAGAAATTCGCTTAATTAA**TTAATTTCTAAAATCTCTAAT	This work	oKA281
AGCTTGGGTGGTCATATGATGGGCAACCGCGGCATG	This work	oKA288
**CGGGGCCTCCATGGC**TTAGAGGTCGAAGGGGGGCCTGGG	This work	oKA289
GCCATGGAGGCCCCG	This work	oKA290
CATATGACCACCCAAGCTAGC	This work	oKA291
ATATATACCGGTATGTTGAGTTCAACAGCAATATTAAAG	This work	oKA298
ATATATACCGGTTTAATACAAATGAAGTAAATCACTATTTTCAGATTG	This work	oKA299
TGGTGGTTCTGGGGCCCAAAATGAAACCGGTTTAATTAAGCGAATTTCTTATGATTTATG	This work	oKA300
**CTTTAAGAAGGAGATATACAT**ATGGAGGCGCTAATTC	This work	oKA355
**GAACAGAACTTCCAGCGGATCCGC**CCAAAGATGAGTCTCCC	This work	oKA356
AGCTGCTCTTCTAGTATTCTCGAGGAGTACATCC	This work	oKB001
TATATAGCTCTTCATGCTAGTTTTTCAACCCTTTCTTCCAGAGC	This work	oKB082
ATTTAGTCGACATGGAGCAGGAGGCAGCCAAG	This work	oKB153
ATTTAGGATCCTTACTTCCTCATGCCTGTAGATGGG	This work	oKB160
ATTTAGTCGACATGATCCTGGAGGAGTACATC	This work	oKB161
ATTTAGTCGACATGGCCAAGAACAAGGAGTTCTTTG	This work	oKB162
ATTTAGGATCCTTACAGCTTCTCCACTCTTTCCTCCAG	This work	oKB163
TATATAGCTCTTCATGCAGACTGTATGATATTCTGGGTCTCTGT	This work	oKB179
ATATATGCTCTTCTAGTGCAAGAGCTCGCCAGTTTGAACTTGAA	This work	oKB210
AAAAACGCGTCGACTTACTTACGCATCCC	This work	pLS163
**Chemicals, enzymes and other reagents**		
Ampicillin	Roth	K029.2
Kanamycin	Roth	T832.2
Chloramphenicol	Roth	3886.1
Tris	Roth	4855.2
NaCl	Roth	3957.2
L(+) Arabinose	Roth	5118.2
D-Sorbitol	Roth	6213.1
PMSF	Thermo Fisher	36978
Proteinase K	Machery Nagel	740506
Glycerol	Sigma	G7757-1L
Imidazole	Roth	X998.4
D(+) Saccharose	Roth	4621.1
EDTA	Huberlab	A1104.0500
MOPS	Roth	6979.2
SDS	Roth	CN30.3
Silver nitrate	Sigma	209139-25 G
Polyethylenimine (PEI)	Chemie Brunschwig AG	POL24765-100
RPMI media	Gibco/Fischer scientific	11530586
L glutamate	Gibco/Fisher scientific	31905430
Fetal bovine serum	Gibco/Thermo Fisher	A5256701
Trypsin	Gibco/Thermo Fisher	15400054
OptiMEM	Gibco/Thermo Fisher	31985070
DPBS	Gibco/Thermo Fisher	10010023
HEPES	Roth	6763.3
NADH	Sigma	10128023001
Pyruvate kinase and lactate dehydrogenase	Sigma	P0294-5ML
β-mercaptoethanol	Roth	4227.1
Phosphoenolpyruvate	Sigma	P7002-250MG
MgCl_2_	Sigma	63068-250G
Protein A/G magnetic beads	Thermo Fisher	88803
Ultralink biosupport beads	Thermo Fisher	53110
Bromphenol Blue	Sigma	1125530025
Glacial acetic acid	Sigma	695092-500 ML
Glycine	Fluka	3908.2
Tween 20	Roth	9127.1
Protease inhibitor cocktail tablet (without EDTA)	Merck	11836170001
Bovine serum albumin (BSA)	Roth	8076.4
Milk powder	Roth	T145.3
Acrylamide	Roth	3029.1
TEMED	Roth	2367.1
APS	Roth	9502.3
GMPPCP	Jena Bioscience	NU-402-5
GTP	Sigma	G8877-100MG
DTT	Sigma	10197777001
Ethanol	Fluka	02860- 1 L
Methanol	Sigma	34885
Formaldehyde	Fisher Scientific	F/1501/PB15
Na_2_CO_3_	Sigma	13418-1KG-R
Deoxyribonuclease	Sigma	DN25-1G
Polyethylenimine hydrochloride (PEI)	Polysciences	24765-100
T4 DNA Ligase	NEB	M0202
Isopropyl-β-d-thiogalactoside	Roth	CN08.2
MitoTracker Deep Red FM	Thermo Fisher	M22426
MitoTracker Orange CMTMRos	Thermo Fisher	M7510
Triton X-100	Roth	3051.4
DMEM, high glucose, pyruvate	Gibco	41966052
L-glutamine (200 mM)	Gibco/Thermo Fisher	25030081
Thermo Scientific^TM^ Phusion^TM^ High-Fidelity DNA Polymerase (2U/μl)	Thermo Fisher	16524551
NEBuilder HiFi DNA Assembly Master Mix	New England Biolabs	E2621L
Q5^®^ Hot Start High-Fidelity DNA Polymerase	New England Biolabs	M0493S
CIAP (Calf Intestinal Alkaline Phosphatase), 20U/μl	Invitrogen/Thermo Fisher Scientific	18009019
Yeast extract, powder	Formedium	YEA03
Peptone	Formedium	PEP03
D(+)-glucose anhydrous	Formedium	GLU04
Complete Supplement Mixuture (CSM), Single Drop-Out -His	Formedium	DCS0079
CSM, Double Drop-Out -Leu, -Trp	Formedium	DCS0569
CSM, Single Drop-Out -Leu	Formedium	DCS0099
CSM, Single Drop-Out -Ura	Formedium	
CSM, Double Drop-Out -Leu, -Ura	Formedium	DCS0589
Base Trizma	Sigma-Aldrich/Merck	T1503
Lithium acetate dihydrate	Sigma-Aldrich/Merck	L6883
Salmon Sperm DNA, sheared (10 mg/mL)	Invitrogen/Thermo Fisher Scientific	AM9680
UltraPure Agarose	Invitrogen/Thermo Fisher Scientific	16500100
Sodium hydroxide	Carl Roth	6771.1
Trichloroacetic acid	Sigma-Aldrich/Merck	T9159
Tetramethyl rhodamine, methyl ester (TMRM)	Abcam	ab275547
ProLong Diamond Antifade Mountant	Invitrogen/Thermo Fisher Scientific	P36961
AgeI-HF	New England Biolabs	R3552S
BamHI-HF	New England Biolabs	R3136S
FastDigest BamHI	Thermo Scientific	FD0054
BspQI	New England Biolabs	R3136S
DpnI (10U/μL)	Thermo Scientific	ER1701
FastDigest EcoRI	Thermo Scientific	FD0274
FastDigest HindIII	Thermo Scientific	FD0504
FastDigest NdeI	Thermo Scientific	FD0583
FastDigest NheI	Thermo Scientific	FD0973
SalI-HF	New England Biolabs	R3138S
FastDigest SalI	Thermo Scientific	FD0644
SmaI	New England Biolabs	R0141S
XhoI	New England Biolabs	R0146S
**Software**		
GraphPad v7.01	https://www.graphpad.com	
CorelDraw 2019	https://www.coreldraw.com	
Gen5 v3.16	https://www.agilent.com	
Cytiva UNICORN 7 v6.3	https://www.cytivalifesciences.com	
Zetasizer Software from Malvern Panalytical v8.02	https://www.malvernpanalytical.com	
ImageQuant 800 v2.0.0	https://www.cytivalifesciences.com	
FlowJo v10.1.0.0	https://www.flowjo.com/	
AlphaFold2	Jumper et al, 2021	
ImageJ v1.54p	https://imagej.net/	
Seahorse control software v10.1.0.11	https://www.agilent.com	
Leica Application Suite X v3.5.7.23225	https://www.leica-microsystems.com	
**Other**		
Microfluidizer	Microfluidics	LM20
BioTek 5 plate reader	Agilent	Cytation5
BCA assay reagent	Thermo Fisher	23225
NucleoSpin Plasmid	Machery Nagel	740588.50
Superdex 200 column Cytiva	GE Healthcare/Cytiva	GE28-9909-44
ÄKTA pure	Cytiva	29046694
Fraction collector	Cytiva	29027743
Auto injector	Cytiva	29347718
Zetasizer Nano ZS	Malvern Panalytical	
Cell homogenizer	isobiotec	
NanoDrop 2000 photospectrometer	Thermo Fisher	1.6.198
WESTAR SUN ECL susbtrate	Cyanagen	XLS063,0250
SuperSignal PLUS Chemiluminescent Substrate	Thermo Fisher	34580
EndoFree Plasmid Maxi Kit	Qiagen	12362
PCR Clean-up Kit	Machery Nagel	740609.50
LSR II Fortessa Cell Analyzer	BD Biosciences	
MitoProbe TMRM Assay Kit for Flow Cytometry	Thermo Fisher	M20036
Seahorse XF Cell Mito Stress Test Kit	Agilent	103015-100

### Cells, bacteria, and infection

Human HeLa and A549 lung epithelial carcinoma cells were cultivated in RPMI 1640 medium, supplemented with 10% fetal bovine serum (FBS) and 2 mM L-glutamine (all from Gibco; Thermo Fisher Scientific). HEK293 cells were cultivated in Dulbecco’s Modified Eagle Medium (DMEM; Gibco), supplemented with 10% FBS and 2 mM L-glutamine. The cells were maintained at 37 °C and 5% CO_2_ in a humid atmosphere.

*L. pneumophila* strains harboring derivatives of pMMB207C (pIF009, pKB198, pKB208, pKB209, pNP99, pNT28) were grown on charcoal yeast extract (CYE) agar plates, buffered with *N*-(2-acetamido)-2-aminoethane sulfonic acid (ACES) containing 5-10 μg/ml chloramphenicol (Cam) at 37 °C for 3 days. Bacterial overnight cultures were prepared by resuspending the strains in AYE containing 5 μg/ml Cam (OD_600_ of 0.1) and growing the liquid cultures at 37 °C for 20.5–21.5 h until an OD_600_ of ca. 5 was reached (stationary phase).

For infection of mammalian cells, bacterial suspensions were prepared in the appropriate mammalian cell growth medium and added to the cells at the multiplicity of infection (MOI) indicated. The infections were synchronized by centrifugation (450–490 × *g*, 10 min). The infected cells were washed 1 h post infection with DPBS (Gibco) and further incubated in fresh growth medium for the time indicated.

### Biosafety

All handling of virulent *L. pneumophila* strains was conducted in contained systems at Biosafety Level 2, as required by Swiss biosafety regulations and approved by the Federal Office of Public Health notification A141332/2.

### Molecular cloning

Plasmids and oligonucleotides used in this study are listed in the Reagents and tools table. If not indicated otherwise, PCRs were performed using Phusion High-Fidelity DNA polymerase (Thermo Scientific) according to the manufacturer’s protocol and with the addition of DMSO. Backbones and inserts were digested with restriction enzymes from Thermo Fisher Scientific or New England Biolabs (NEB) at 37 °C for 20–60 min. Ligations were performed at a vector:insert ratio of 1:3 with T4 DNA Ligase (NEB) at either 4 °C overnight or at room temperature (RT) for 1 h. Competent *E. coli* TOP10 were transformed with the ligated constructs through heat shock (42 °C, 30 sec). Transformants were selected on LB plates containing ampicillin (Amp) or kanamycin (Kan). Colony PCRs were performed with in-house Phusion or Taq polymerase. All PCR-amplified constructs were sequenced (Microsynth). Plasmids were isolated using the NucleoSpin Plasmid Mini kit for plasmid DNA (Macherey-Nagel), the NucleoBond Xtra Midi Plus EF Kit (Macherey-Nagel), or with the EndoFree Plasmid Maxi Kit (Qiagen). PCR products were extracted from gels with the NucleoSpin Gel and PCR Clean-up Kit (Macherey-Nagel).

For the generation of yeast two-hybrid (Y2H) vectors, RidL was amplified from template pKB002 using the primers oKA001/oLS163 and fused to the DNA-binding domain of pLexA (BamHI/SalI), resulting in pKA001. Large GTPases were fused to the activating domain of pAct2.2. Vps1 was amplified using oKA004/oKA005 from template pKB242 and inserted into pAct2.2 through NdeI/EcoRI, resulting in pKA004. Drp1 was amplified with oKA020/oKA021 from template mCh-Drp1 (Addgene #49152 (Friedman et al, 2011)) and inserted via NdeI/BamHI, yielding pKA009. Dnm2 was amplified with oKA023/oKA024 from template pKB241 and inserted into pAct2.2 through EcoRI/NdeI, resulting in pKA011. Dnm3 was amplified with oKA261/oKA262 from template pDONR201-DNM3 (HsCD00080526; DNASU Plasmid Repository) and inserted into pAct2.2 via SmaI/BamHI, yielding pKA104. The construct pAct2.2-Dnm1 (pKA103) was assembled through NEBuilder HiFi DNA Assembly (NEB). The insert (*Dnm1*) was amplified from pEGFP-N1-dynamin (Addgene #120313 (Kong et al, 2018)) using primers oKA288/oKA289 and Q5 Hot Start High-Fidelity DNA Polymerase (NEB), including Q5 High GC Enhancer. Due to the lack of matching restriction sites, backbone pAct2.2 was fully amplified using oKA290/oKA291 and Q5 Hot Start High-Fidelity DNA Polymerase and DpnI-digested for clearance of the DNA template. DNA assembly was performed as indicated in the NEBuilder HiFi DNA Assembly manufacturer’s manual. Both the DNA-binding domain of pAct2.2 and the insert *Dnm1* were sequenced. For pACT2.2-DymA (pKA014), *DymA* was amplified with oKA029/oKA030 from pDXA-GFP-DymA (gift from F. Letourneur), the backbone and insert were digested with NdeI/EcoRI, and the backbone was treated with CIAP (Calf Intestinal Alkaline Phosphatase; Invitrogen) before ligation. For pAct2.2-DymB (pKA015), *DymB* was initially amplified with oKA263/oKA264 from pME18SFL3-DymB (#G01672; National BioResource Project (NBRP), Nenkin, Japan). The PCR product was cleaned up and conducted to A-tailing (0.2 mM ATP, NEB One Taq Hot Start Polymerase, 94 °C for 30 sec, 65 °C for 10 min), followed by DpnI digest (37 °C for 1 h, heat-inactivation at 80 °C for 20 min) and ligated with the pGEM-T Easy vector (pGEM-T Easy Vector Systems; Promega) overnight at 4 °C, resulting in the shuttle vector pKA105. *DymB* was cut out from pKA105 with NdeI/BamHI and ligated with pAct2.2, yielding pKA015.

To generate split-GFP vectors for bimolecular fluorescence complementation (BiFC) of RidL and large fission GTPases, RidL was amplified with the primers oKA183/oKA051 from template pKA001 and fused to GFP_11_-mCherry in pRS315-NOP1pr-GFP_11_-mCherry-PUS1 (pSJ1321, Addgene #86413 (Smoyer et al, 2016)) by replacing PUS1 (NheI/SalI), resulting in pKA067. The large fission GTPases Drp1, Dnm1, Dnm2, Dnm3, DymA, DymB and Vps1 were fused to GFP_1–10_ in pRS316-NOP1pr-GFP1-10-SCS2TM (pSJ2039, Addgene #86418 (Smoyer et al, 2016)) by replacing SCS2TM. For cloning of pKA100 (pRS316-GFP_1–10_-Drp1), pKA097 (pRS316-GFP_1–10_-Dnm1), pKA098 (pRS316-GFP_1–10_-Dnm2), pKA099 (pRS316-GFP_1–10_-Dnm3), pKA114 (pRS316-GFP_1–10_-DymA) and pKA072 (pRS316-GFP_1–10_-Vps1) the backbone was amplified (excluding the *scs2tm* sequence) with the primers oKA207/oKA208 and Q5 Hot Start High-Fidelity DNA Polymerase. *Drp1* was amplified with oKA245/oKA246, *Dnm1* with oKA239/oKA240, *Dnm2* with oKA241/oKA242, *Dnm3* with oKA243/oKA244, *DymA* with oKA280/oKA281 and *Vps1* with oKA209/oKA210. Inserts and fragments were assembled with NEBuilder HiFi DNA Assembly (NEB). For the generation of pRS315-GFP_1–10_-DymB (pKA115), an AgeI restriction site was initially introduced into the backbone pSJ2039, replacing *scs2tm*, by amplifying the full backbone excluding *scs2tm* with oKA224/oKA225. The PCR product was annealed with an oligonucleotide bridge containing the AgeI restriction site (oKA300) through NEBuilder assembly, resulting in plasmid pKA118. An AgeI-flanked *DymB* construct was amplified with oKA298/oKA299 and inserted into a pGEM-T Easy vector as described for pKA105, resulting in pKA117. Finally, *DymB* was cut out of pKA117 and inserted into pKA118 (AgeI), resulting in pKA115.

pKB248 and pKB252 for the production of GFP-RidL^opt^ and untagged RidL^opt^, respectively, were cloned by amplification of codon-optimized RidL from template pKB245 (pEGFP-N1-RidL^opt^; Genscript) with oKB160/oKB161 and insertion into pEGFP-C1 (resulting in pKB248) or into pEGFP-N1 (resulting in pKB252) through Sal/BamHI. For construction of pKB249 (pEGFP-C1-RidL^opt^_9–258_-Stop) or pKB250 (pEGFP-C1-RidL^opt^_259–1167_-Stop), RidL fragments were amplified using pKB245 as the template and oKB162/oKB163 (pKB249) or oKB153/oKB160 (pKB250), respectively, and inserted into pEGFP-C1 through SalI/BamHI. Analogously, for the production of untagged RidL^opt^_9–258_ or RidL^opt^_259–1167_, the amplified fragments were inserted into pEGFP-N1, resulting in pKB253 (pEGFP-N1-RidL^opt^_9–258_-Stop) and pKB254 (pEGFP-N1-RidL^opt^_259–1167_-Stop).

For the production and purification of RidL fragments, RidL_1–258_ (oKB001/oKB082) and RidL_437–1100_ (oKB210/oKB179) fragments were amplified using pKB002 as a template and inserted into pINIT through an FX cloning system. RidL_1–258_ was cut (BspQI) and inserted into pBXNH3, resulting in pKB139. RidL_437–1100_ was inserted into pBXC3GH, resulting in pKB306.

For Drp1 production, human Drp1 was amplified with oKA355/oKA356 using mCh-Drp1 or pKA080 as templates and inserted into pET21-Drp1 (Addgene #72927 (Liu and Chan, 2015)) through NdeI/BamHI, replacing the murine Drp1, and yielding pKA173 (pET21-Drp1). The Drp1_A395D_ point mutation was created using QuikChange, with pKA173 as a template, yielding pEV001.

Plasmid pKB198 (pMMB207C-GFP-P_*ridL*_-RidL_∆β_) was constructed by cloning the insert of pKB151 (XhoI) into pIF009, pKB207 (pMMB207C-mCerulean-MCS) was constructed by cloning the insert of pNP99 (HindIII, EcoRI) into pNT29, pKB208 (pMMB207C-mCerulean-P_*ridL*_-RidL) was constructed by cloning the insert of pIF009 (BamHI/SalI) into pKB207, and pKB209 (pMMB207C-mCerulean-P_*ridL*_-RidL_∆β_) was constructed by cloning the insert of pKB198 (BamHI/SalI) into pKB207.

### Protein production and purification

To produce human Drp1, *E. coli* BL21(DE3) harboring plasmid pKA173 was grown in LB broth containing 100 μg/ml Amp, induced with 1 mM isopropyl-β-d-thiogalactoside (IPTG; Roth) at OD_600_ 0.8 and left to grow overnight at 21 °C. To produce RidL and its N- and C-terminal fragments, *E. coli* MC1061 containing a pBXNH3-derivative (N-terminal cleavable His_10_-tag) were grown in LB/100 μg/ml Amp and induced at OD_600_ 0.7 with 0.02% (w/v) L(+)-arabinose overnight at 16 °C.

Bacterial pellets were resuspended on ice in lysis buffer (50 mM Tris/HCl, pH 7.5, 200 mM NaCl) supplemented with 3 mM MgSO_4_, 1 mM PMSF, and DNAse (Sigma) and lysed by using high-pressure homogenization (Microfluidizer; Microfluidics) with a pressure of 21,000 PSI. The resulting lysate was centrifuged (16,000 × *g*, 4 °C, 30 min). Lysates were then passed through a Ni^2+^-NTA affinity chromatography column, washed with wash buffer (50 mM imidazole, pH 7.5, 200 mM NaCl, 10% glycerol), and His-tagged protein was eluted with elution buffer (washing buffer with 300 mM imidazole). To remove imidazole, the buffer was exchanged with the size-exclusion chromatography (SEC) buffer (50 mM Tris/HCl, pH 7.5, 200 mM NaCl) using PD10 desalting columns (GE Healthcare). The protein was incubated overnight with 3C protease to remove the His-tag (produced in-house, 10 ug/ml). As a final purification step, the protein was loaded onto a Superdex 200 10/300 GL (GE Healthcare) equilibrated with SEC buffer. Fractions where the monodispersed peak eluted were combined and concentrated with Amicon Ultra-4 centrifugal filter units, using a molecular weight cutoff of 10 (RidL_N_), 50 (RidL_C_, Drp1) or 100 kDa (full-length RidL). Protein concentration was determined by OD_280_ using a NanoDrop 2000 photospectrometer and calculated based on theoretical extinction coefficients (www.expasy.ch/tools/protparam.html). For storage, the protein aliquots were snap-frozen and stored at −80 °C.

### Protein pull-down in cell lysates and interactions of purified proteins

For pull-down experiments, purified RidL (full-length, N-, and C-terminal fragments, RidL_∆β_) or SidC were covalently linked to polyacrylamide beads (UltraLink Biosupport; Thermo Fisher Scientific) according to the manufacturer’s protocol. The efficiency of the coupling was determined by using a BCA protein determination assay to check equimolar amounts of RidL being linked to the beads. To produce lysates, HeLa cells were seeded at a density of 3 × 10^6^ cells in T75 flasks and were left to grow for 24 h as described above. The cells were collected and lysed with a ball homogenizer (Isobiotec) using SEM buffer (250 mM sucrose, 1 mM EDTA, 10 mM MOPS-KOH, pH 7.2) containing protease inhibitors (Pierce Protease Inhibitor Mini Tablets, EDTA-free; Thermo Fisher Scientific). Lysed cells were collected and spun down at 18,000 × *g* at 4 °C to separate proteins from cell debris. The supernatant was collected and incubated with the RidL or SidC-coupled beads for 1 h at 4 °C with gentle rotation. After incubation, the liquid was removed using a syringe, the beads were washed with SEC buffer, collected, resuspended in SDS buffer, and boiled at 95 °C for 5 min, prior to analysis by SDS–PAGE.

For pull-down experiments with proteins, purified Drp1 was coupled to UltraLink Biosupport beads according to the manufacturer’s instructions and incubated with purified RidL (131 kDa), RidL_N_ (RidL_1–258_, 30 kDa), or RidL_C_ (RidL_437–1100_, 75 kDa) for 1 h at 4 °C with gentle rotation. Following incubation, beads were collected by centrifugation or magnetic separation and washed two times with cold binding buffer (50 mM Tris-HCl, pH 7.5, 150 mM NaCl) to remove unbound proteins. Bound proteins were eluted by the addition of SDS sample buffer and incubation at 95 °C for 5 min, followed by SDS–PAGE and visualization by silver stain, or anti-RidL Western blot. Purified RidL and RidL_∆β_ were also preincubated with equimolar amounts of purified Vps29 for 30 min on ice in binding buffer, prior to binding to beads-coupled Drp1 and visualization by silver stain.

### GTPase activity assay

A continuous GTPase activity assay was used to analyze the activity of Drp1 in the presence and absence of RidL. Prior to running the assay, Drp1 samples with and without RidL were incubated for 1 h at RT, with shaking at 350 rpm. Drp1 was diluted to a final concentration of 1 μM in a master mix solution (50 mM HEPES/KOH, pH 8, 150 mM NaCl, 0.5 mM MgCl_2_, 10 mM β-mercaptoethanol, 4 mM phosphoenolpyruvate, 0.3 mM NADH, and 10 units of pyruvate kinase and lactate dehydrogenase). Once the samples were pipetted into a 96-well UV-transparent plate, GTP was added to all samples to a final concentration of 1 mM. The depletion of NADH was measured at 340 nm (BioTek Cytation 5; Agilent) over the course of 2 h. As controls, wells were filled with master mix with and without NADH and used to correct for background. The rate of NADH depletion was used to measure the GTPase activity of Drp1.

### Co-immunoprecipitation of RidL and Drp1

To ectopically produce codon-optimized RidL, HeLa cells were transfected with plasmid pKB252 using 4 μg polyethylenimine (PEI) per μg plasmid. After 24 h, the HeLa cells (4 T75 flasks) producing RidL were lysed in ice-cold lysis buffer (50 mM Tris-HCl, pH, 50 mM NaCl) supplemented with a protease inhibitor cocktail tablet (Thermo Fisher Scientific). Lysates were cleared by centrifugation (18,000 ×* g*, 30 min, 4 °C) and incubated with a polyclonal anti-RidL antibody (Finsel et al, 2013) overnight at 4 °C with gentle rotation. Subsequently, protein A/G magnetic beads were added and incubated for an additional 1 h at RT. Beads were collected using a magnetic rack and washed with lysis buffer to remove nonspecific interactions. Bound proteins were eluted by incubation with SDS sample buffer and heating at 72 °C for 10 min. Eluted proteins were then separated by SDS–PAGE and analyzed by Western blot with anti-RidL, anti-Drp1, or anti-Dnm1 antibodies.

### Western blot analysis

For assessment of RidL, Drp1, Dnm1, Dnm2, Dnm2, Vps29, AIF, GAPDH, or Cytc in cell lysates, isolated mitochondria and other cellular fractions, the samples were separated by SDS–PAGE (10% acrylamide gels) and blotted onto nitrocellulose membranes at 300 mA, 4 °C for 90 min. In general, membranes were blocked in 5% milk, 5% BSA (Drp1), or 3% BSA (RidL) for 60 min at RT and incubated overnight at 4 °C with primary antibodies: rabbit polyclonal anti-AIF (17984-1-AP, 1:5000; Proteintech), rabbit monoclonal anti-GAPDH (14C10) (#2118, 1:1000; Cell Signaling Technology), rabbit polyclonal anti-Cytc (10993-1-AP, 1:1000; Proteintech), mouse anti-Vps29 (#sc-398874, 1:100; Santa Cruz Biotechnology), rabbit monoclonal anti-DRP1 (D8H5) (#5391, 1:1000; Cell Signaling Technology), mouse monoclonal anti-Drp1 (ab56788, 1:1000; Abcam), monoclonal anti-Dnm1 (#68009-1-Ig, 1:1000; Proteintech), rabbit polyclonal anti-Dnm2 (ab3457, 1:1000; Abcam), rabbit polyclonal anti-Dnm3 (PA1-662, 1:1000; Thermo Fisher Scientific), mouse monoclonal anti-GFP Living Colors JL-8 (#632380, 1:5000; Clontech), or polyclonal rabbit anti-RidL (1:5000, (Finsel et al, 2013)). For the detection of fusion proteins of large GTPases and GFP_1–10_ in *S. cerevisiae*, GFP polyclonal antibody (A-11122, 1:2000 in 5% milk; Thermo Fisher Scientific) was used. To assess Tom20 in mitochondrial fractions, samples were separated on SDS gels containing 12.5% acrylamide and blotted onto 0.2-μm nitrocellulose membranes (100 V, 4 °C, 1 h). Tom20 polyclonal antibody (11802-1-AP; Proteintech) was used at a dilution of 1:1000 in 5% milk (in TBST) and incubated for 60 min. at 37 °C or for 90 min. at RT. Secondary antibodies (#NA934-1ML, ECL donkey anti-rabbit IgG HRP-linked whole Ab; Cytiva/Amersham; #31430, goat anti-mouse IgG [H + L] HRP-linked secondary antibody; Invitrogen; #NA931-1ML, ECL HRP-linked sheep anti-mouse IgG; Cytiva/Amersham) were diluted 1:5000 in 4% milk and incubated for 1 h at RT. Protein detection was conducted with Westar Sun ECL Substrate (Cyanagen) or with SuperSignal^TM^ West Pico PLUS Chemiluminescent Substrate (Thermo Fisher Scientific) and the ImageQuant 800 (Amersham).

### Negative stain electron microscopy

Drp1 was visualized by negative stain electron microscopy as described (Mears and Hinshaw, 2008). To induce filaments, Drp1 was diluted into negative staining buffer (50 mM Tris/HCl, pH 7.5, 200 mM NaCl, and 1 mM DTT) and incubated with 0.5 mM GMPPCP for one hour. Samples incubated with RidL and SidC were added simultaneously upon the addition of GMPPCP. To visualize the filaments, samples were placed onto carbon film-coated, 300 mesh, copper grids (Science Services). After 1 min of incubation, the sample was blotted with filter paper. The grids were immediately washed with uranyl acetate, blotted again, and then incubated with the uranyl acetate once again for 1 min After staining, the grids were dried for about 10 min before imaging on the FEI Tecnai Spirit, a 120 kV electron microscope from the University of Zürich Center for Microscopy (ZMB).

### Dynamic light scattering

To quantify the presence and absence of Drp1 filaments in solution, we measured the amount of scattered light among various samples using dynamic light scattering (Zetasizer Nano ZS; Malvern Panalytical). The scattered intensity was measured at 173 degrees. The oligomerization of Drp1 (1 µM) induced by 0.5 mM GMPPCP, in the absence or presence of RidL (3 µM) or SidC (3 µM), was assessed in a volume of 100 µl. Immediately after the components were mixed, the sample was pipetted into a cuvette, and scattered intensity was read by the machine in 1-min intervals. The hydrodynamic size distributions were calculated using the cumulant and CONTIN methods.

### Yeast two-hybrid assays

For yeast two-hybrid (Y2H) assays, RidL was fused to a LexA DNA-binding domain (pLexA), and large fission GTPases (Drp1, Dnm1, Dnm2, Dnm3, DymA, DymB, or Vps1) were fused to the Gal4 activation domain (pAct2.2). Upon transformation of the Y2H reporter strain NMY32, an interaction between bait and prey proteins leads to the reversion of histidine auxotrophy.

Strain NMY32 was grown on yeast-extract peptone dextrose (YPD) agar plates at 30 °C for 3 days. For the generation of competent cells, NMY32 was grown in liquid YPD at 30 °C overnight, diluted to an OD_600_ of 0.2 and further incubated until an OD_600_ of 0.6–0.8 was reached. Yeast cells were harvested (600 × *g*, 3 min, 4 °C), washed with bi-distilled H_2_O (ddH_2_O) and LiSorb (10 mM Trizma base, pH 8.0, 100 mM C_2_H_3_LiO_2_, 1 mM EDTA, and 1 M sorbitol) and finally resuspended in LiSorb.

For transformation, 500 ng plasmid DNA, 50 μg sheared salmon sperm DNA (Invitrogen), 50 μl of competent yeast cells, and 300 μl LiPEG (10 mM Trizma base, pH 8.0, 100 mM C_2_H_3_LiO_2_, 1 mM EDTA, and 45% PEG4000) were incubated at 30 °C for 30 min while being mixed. Then, 35 μl DMSO were added to the mixture before exposure to a heat shock at 42 °C for 15 min. Yeast transformants were washed with and resuspended in ddH_2_O, plated for selection onto SD-Leu-Trp agar plates and incubated for 3 days at 30 °C.

For Y2H assays, single yeast colonies were re-streaked onto SD-Leu-Trp agar plates, grown for another 2–3 days at 30 °C and resuspended in ddH_2_O. Yeast suspensions were adjusted to an OD_600_ of 0.5 and spotted in a tenfold dilution series onto SD-His agar plates containing 0.5 mM 3-amino-1,2,4-triazole (3-AT) and grown for 3 days at 30 °C to select for protein–protein interactions. As a control, the serial dilutions were additionally spotted onto SD-Leu-Trp agar plates.

### Bimolecular fluorescence complementation

For bimolecular fluorescence complementation (BiFC) using split GFP, RidL was fused to one strand of the GFP β-barrel (GFP_11_) and mCherry using the plasmid pRS315-NOP1pr-GFP_11_-mCherry (derived from pSJ1321) (Smoyer et al, 2016), resulting in the fusion protein GFP_11_-mCherry-RidL (pKA067). Large GTPases (Drp1, Dnm1, Dnm2, Dnm3, DymA, DymB, or Vps1) were fused to the GFP_1–10_ fragment by modification of pSJ2039 (Smoyer et al, 2016). The yeast strain BY4741 was first transformed with pKA067 (GFP_11_-mCherry-RidL), selected on SD-Leu, and competent BY4741/pKA067 was then transformed with plasmids encoding GFP_1–10_ fused to a large GTPase (selection on SD-Leu-Ura).

For fluorescence imaging of GFP- and mCherry signals, overnight cultures were prepared by inoculating selective media with the transformants grown at 30 °C. On the day of the experiment, overnight cultures were diluted to an OD_600_ of 0.2 and further grown until an OD_600_ of 0.6–0.8 was reached. At this stage, aliquots of the yeast cultures were transferred to eight-well μ-slides, ibiTreat (#80826; ibidi), embedded with 0.1% agarose and imaged by confocal laser scanning microscopy (Leica SP8 DMi8 CS with AFC, objective HC PL APO CS2 63× oil) with 4× zoom, bi-directional laser scan and a scanning speed of 400 Hz. For each condition, three individual transformed yeast colonies were imaged per experiment. For each colony/sample, z-stacks (ten images) of tile scans were recorded, in order to assess the total fluorescence signal within each cell. For image analysis, the z-stacks of each tile image were merged in ImageJ/Fiji. Individual thresholds for GFP and mCherry signals were defined, and the integrated densities of both channels were measured.

To assess the production of GFP_1–10_ fusion proteins, the *S. cerevisiae* strain was transformed with plasmids pKA072, pKA097, pKA098, pKA099, pKA114, and pKA115 and selected on SD-Ura drop-out medium. Transformants were grown in selection medium overnight, diluted to OD_600_ = 0.2 and further grown as a day culture to the exponential phase (OD_600_ = 0.6–1). Each 10 mL of the day cultures were collected (4300 ×* g*, 5 min, RT), yeast pellets were resuspended in 2 mL dH_2_O and sample volumes corresponding to OD_600_ = 4 were taken for further analysis. The transformants were collected again, yeast pellets were incubated on ice for 5 min., resuspended in each 150 μl lysis solution (1.85 M NaOH, 1 M β-mercaptoethanol), incubated on ice for 10 min. before the addition of 150 μl 50% trichloroacetic acid. The lysates were incubated on ice for another 10 min. Next, protein precipitates were collected at 2500 ×* g* (5 min, 4 °C), pellets were washed with each 1 mL acetone, centrifuged again and eventually resuspended in SDS sample buffer and boiled at 70 °C for 10 min.

### Mitochondria isolation

Mitochondria were isolated from infected HeLa or transfected HEK293 cells as described (Morgenstern et al, 2021). In brief, previously infected or transfected cells from 12 confluent T75 flasks were trypsinized, collected (450 ×* g*, 5 min), resuspended in SEM buffer, supplemented with protease inhibitors (Thermo Fisher Scientific), lysed using a ball homogenizer, and kept on ice. This cell lysate was centrifuged (800 × *g*, 4 °C, 5 min), resulting in a nuclei-enriched pellet and an organelle-containing supernatant. The supernatant was centrifuged again (800 ×* g*, 4 °C, 5 min), followed by a final centrifugation step (8000 × *g*, 10 min, 4 °C), resulting in a mitochondria-enriched pellet (crude mitochondria, “cM”) and a supernatant referred to as a cytoplasmic fraction (“cyt”). To receive a pure mitochondria fraction (“pM”), the crude mitochondria pellet was resuspended in SEM buffer containing protease inhibitor, subjected to sucrose density gradient ultracentrifugation (60, 32, 23, and 15% sucrose in 10 mM MOPS/1 mM EDTA, pH 8.0), and centrifuged (ca. 133,500 × *g*, 1 h, 4 °C). The pure mitochondria were retrieved from the interphase between 60 and 32% sucrose, diluted with the twofold volume of SEM buffer, collected (8000 × *g*, 10 min, 4 °C) and finally resuspended in 200 μl SEM buffer.

For the expression of GFP-tagged full-length RidL and fragments, HEK293 cells were transfected in 90% confluent T75 flasks with each 23.8 μg plasmid DNA per flask by polyethylenimine (PEI) treatment (PEI MAX; Polysciences). A mix of plasmid DNA, PEI (95 μg per flask, dissolved in H_2_O) and OptiMEM I (Gibco) was added to HEK293 cells for 1 day before being replaced by growth medium. Transfected HEK293 cells were further processed 2 days after transfection.

For the production of untagged full-length or fragments of RidL in HeLa cells, 80–90% confluent cells were transfected in T75 flasks with each 20 μg plasmid DNA (pKB252, pKB253, and pKB254) and 80 μg PEI (4 μg PEI per μg DNA). The mix of PEI and DNA was added directly to the growth medium and was replaced by fresh medium 1 day post transfection. Transfected cells were harvested after 24–48 h post transfection by scraping, collected (400 × *g*, 5 min, 4 °C), and washed in cold DPBS before mitochondria isolation. Isolated mitochondria were used to assess the localization and protease sensitivity of RidL.

Protease treatment was performed as described (Yek et al, 2025). Briefly, equal amounts of mitochondrial suspension (50 µL per condition) were either left untreated or incubated with proteinase K at a final concentration of 50 µg/mL (15 min on ice), followed by the addition of PMSF (final concentration 1 mM). Mitochondria were pelleted by centrifugation (16,000 × *g*, 10 min., 4 °C) and resuspended in SEM and SDS sample buffer. Samples were incubated at 95 °C for 5 min prior to SDS–PAGE and Western blot against RidL, Cytc, or AIF.

For the analysis of Tom20 and Drp1 on isolated mitochondria, 1.8 × 10^6^ HeLa cells were seeded into each T75 flask 2 days before infection. For each sample, cells from four flasks were collected by gently scraping the cells off in growth medium. The cells were collected at 400 × *g*, 5 min, 4 °C, resuspended in ice-cold DPBS buffer, collected again and resuspended in cold SEM buffer (supplemented with protease inhibitors). Crude mitochondria fractions were isolated as described above. Further purification by sucrose gradient centrifugation was omitted to avoid loss of surface-bound proteins. For the preparation of Western blot samples, protein concentrations were measured by NanoDrop and adjusted with SEM buffer and 5× SDS sample buffer (final concentration: ca. 1 μg/μl). Alternatively, equal volumes of each fraction were mixed with 5× SDS sample buffer, and the highest possible protein concentration was used for Western blot. For each sample, 30 μl were loaded onto SDS gels.

### Imaging of mitochondrial fragmentation

Mitochondrial networks were imaged and analyzed within individual infected HeLa cells. To this end, the cells were seeded at low densities (25,000 cells per well) into 24-well μ-plates (#82426; ibidi) and incubated for ca. 24 h. Before infection, cells were stained with 100 nM MitoTracker Orange CMTMRos (Thermo Fisher Scientific) for 30 min at 37 °C, washed with DPBS and infected (MOI 100) with *L. pneumophila* strains JR32, *ΔicmT*, *ΔridL* harboring pNT28 (GFP) or *ΔridL* harboring pIF009 (GFP, RidL) as described above. Two to six hours post infection, the mitochondrial morphology was assessed by live confocal laser scanning microscopy (Leica SP8 DMi8 CS with AFC, objective HC PL APO CS2 63× oil) with 2× zoom, bi-directional laser scan and a scanning speed of 200 Hz. For quantification, mitochondria within individual cells were identified, and their mean branch lengths and aspect ratios (d_max_/d_min_, d = diameter) were assessed with the Mitochondria Analyzer plugin in ImageJ/Fiji (Chaudhry et al, 2020; Legland et al, 2016).

### Membrane potential of infected cells

HeLa cells were seeded into 12-well plates the day before the experiment and grown at 37 °C, 5% CO_2_ for ca. 24 h. Cells were infected (MOI 100) with *L. pneumophila* strains JR32, *ΔicmT*, *ΔridL* harboring pNT28 (GFP) or *ΔridL* harboring pIF009 (GFP, RidL) as described above. About 1, 3, and 5 h post infection, cells were detached by trypsinization and incubated with 20 nM tetramethyl rhodamine methyl ester (TMRM, ab275547; Abcam) diluted in growth medium (37 °C, 5% CO_2_, 30 min). After incubation, cells were collected (450 × *g*, 5 min), resuspended in DPBS and processed by flow cytometry with an LSR II Fortessa Cell Analyzer (BD Biosciences), corresponding to a total of 2, 4, and 6h post infection. GFP-producing intracellular bacteria were assessed using the blue laser (488 nm), mirror 505LP and filter 530/30 at 450 V, and the TMRM signal was assessed through the yellow/green laser (561 nm), mirror 600LP, and filter 610/20 at 400 V (FSC at 420 V, SSC at 280 V). Greater than 10^4^ infected (GFP-positive) HeLa cells or the total sample volume was processed and measured. The mean TMRM intensities of all infected HeLa cells (based on the GFP signal) were assessed with the FlowJo software. As controls, cells were treated with 50 μM carbonyl cyanide 3-chlorophenylhydrazone (CCCP, 98%; Thermo Fisher Scientific or M20036, MitoProbe TMRM kit for flow cytometry; Invitrogen) for 5 min at 37 °C, 5% CO_2_. TMRM was added to a final concentration of 20 nM directly to the CCCP-containing sample. The sample was further incubated (37 °C, 5% CO_2_, 30 min) and processed as described above.

### Mitochondrial respiration and stress test

Mitochondrial basal and stressed respiration of *L. pneumophila*-infected HeLa cells were tested using a Seahorse XF Pro Analyzer (Agilent) and the Cell Mito Stress Test Kit (Agilent). To this end, 1.5 ×  10^4^ HeLa cells per well were seeded into 96-well plates (Agilent), let rest for 1 h at RT, and incubated for one day at 37 °C, 5% CO_2_. The basal respiration of uninfected HeLa cells was measured at 12 min and 6 min before infection (1 measurement cycle = 6 min). The HeLa cells were then infected (MOI 10, 450 × *g*, 10 min) with *L. pneumophila* and subjected to respiration measurements for 15 cycles (6 min per cycle). The data were normalized to the basal respiration rate. 100 min post infection, a mitochondrial stress test was performed according to the manufacturer’s protocol, with final drug concentrations of 1 μM oligomycin, 0.5 μM FCCP, and 0.5 μM rotenone/antimycin A. The data were normalized to the respiration rate of infected cells immediately before the stress test.

### Protein depletion by RNA interference

The siRNA-mediated depletion of Dnm1 and Drp1 was performed as described (Steiner et al, 2019). In brief, A549 cells were seeded and grown to ca. 80–90% confluency overnight before being transfected with siRNA oligonucleotides targeting Drp1 (Hs_DNM1L_4, Hs_DNM1L_8, Hs_DNM1L_9, and Hs_DNM1L_10 FlexiTube siRNA; Quiagen), Dnm1 (FlexiTube GeneSolution GS1759 for DNM1; Qiagen) or Arf1 (Hs_ARF1_10 FlexiTube siRNA; Qiagen). The siRNA oligonucleotides were mixed with HiPerFect Transfection Reagent (Qiagen) and transferred into 96-well plates before adding each 2 × 10^4^ A549 cells. Transfected cells were incubated at 37 °C, 5% CO_2_ for 48 h before infection with *L. pneumophila* JR32 or Δ*icmT* (MOI 10). Bacterial GFP signal was measured with a plate reader 1 and 24 h post infection. For analysis, the background signal measured for untreated A549 cells was subtracted from the measured values of infected cells. The means of each three technical replicates measured 24 h post infection were divided by the means measured 1 h p.i to show relative intracellular replication. For normalization to treatment with Scrambled siRNA, relative means were normalized to the relative mean of the Scrambled sample of each biological replicate. To compensate for day-to-day variations, the data for Scrambled was set to 100 for each of the different biological replicates. To assess the cytotoxicity of protein depletion, the samples were stained with Zombie Aqua dye (1 h, 37 °C) and processed by flow cytometry. The efficiency of protein depletion was checked by Western blot.

### Immunofluorescence microscopy

For immunostaining of Tom20, Drp1, and pDrp1 (Ser616), 10^5^ HeLa cells each were seeded on coverslips in 24-well plates overnight. The cells were stained with 100 nM MitoTracker Deep Red FM (Thermo Fisher Scientific) for 30 min at 37 °C, washed and infected (MOI 25, 2 h for Tom20 and Drp1, MOI 50 for 1 h for pDrp1 (Ser616)) with mCerulean-producing *L. pneumophila* strains JR32, Δ*icmT*, or Δ*ridL* harboring pNP99 or Δ*ridL* harboring pKB208 or pKB209. Infected cells were fixed with 4% PFA (30 min, RT), permeabilized with 0.25% Triton X-100 (5 min, RT; Tom20, Drp1) or 0.1% Triton X-100 (30 min, RT; pDrp1) and blocked with 10% FBS (Tom20, Drp1) or 1% BSA (pDrp1), diluted in DPBS or PBS (1 h, RT). Samples were incubated with either anti-Drp1 (C-terminal) polyclonal antibody (#12957-1-AP, 1:50 in 1% BSA; Proteintech), anti-Tom20 polyclonal antibody (#11802-1-AP, 1:200 in 1% BSA; Proteintech), or anti-phospho-Drp1 (Ser616) monoclonal antibody (#4494, 1:1000 in 1% BSA; Cell Signaling Technology) for 1.5 h at RT in the dark. After washing, samples were incubated with chicken anti-rabbit IgG (H + L) cross-adsorbed secondary antibody, Alexa Fluor 488 (#A-21441, 1:250 in 1% BSA; Thermo Fisher Scientific) or F(ab’)2-goat anti-rabbit IgG (H + L) cross-adsorbed secondary antibody, Alexa Fluor 488 (A-11070, 1:250 in 1% BSA; Invitrogen) for 45 min. Coverslips were mounted with ProLong Diamond antifade mountant (Thermo Fisher Scientific) and dried overnight at RT. Imaging was performed by confocal laser scanning microscopy (Leica SP8 DMi8 CS with AFC, objective HC PL APO CS2 63× oil) with 2× or 4× zoom, bi-directional laser scan and a scanning speed of 200 Hz.

For analysis, cells were randomly picked based on MitoTracker signal and intracellular bacteria, if infected, without the Alexa Fluor 488 channel being considered. Regions of interest (ROI), surrounding the selected cells (ROI_cell_), were defined in ImageJ/Fiji and individual thresholds for MitoTracker Deep Red and Alexa Fluor 488 channels were set. Subsequently, ROIs comprising the entire MitoTracker Deep Red (ROI_MT_) or Alexa Fluor 488 (ROI_AF_) signal were defined in ImageJ/Fiji. To assess the total Drp1/Tom20/pDrp1 (Ser616) signal within a cell, an intersection of ROI_cell_ and ROI_AF_ was defined, and the respective Integrated Density of the Alexa Fluor 488 signal was measured. To assess the portion of Drp1/Tom20/pDrp1 (Ser616) that is localized on mitochondria, an intersection of ROI_cell_, ROI_MT_ and ROI_AF_ was defined, the respective Integrated Density of Alexa Fluor 488 was measured and divided by the total Drp1/Tom20/pDrp1 (Ser616) Integrated Density assessed in the first step. For improved illustration, the brightness of fluorescence microscopy images was increased in ImageJ.

### Flow cytometry of immuno-stained cells

For flow cytometry of immuno-stained cells, 4 × 10^5^ HeLa cells were seeded per well of a six-well plate and grown overnight. The cells were infected (MOI 50) with GFP-producing *L. pneumophila* strains JR32, Δ*icmT*, or Δ*ridL* harboring pNT28, or Δ*ridL* harboring pIF009 or pKB198. One hour post infection, cells were washed two times with DPBS, detached by trypsinization for 3 min at 37 °C, collected (500 ×* g*, 5 min) and fixed with 4% PFA (15 min, RT). After one wash with PBS, cells were permeabilized with 90% methanol for 10 min on ice, followed by a wash with PBS. For immunostaining, cells were incubated (1 h, RT) with an anti-pDrp1 (Ser616) rabbit monoclonal antibody (#4494, 1:1000 in 0.5% BSA; Cell Signaling Technology), washed with PBS and incubated (30 min, RT) with an Alexa Fluor 594-conjugated secondary antibody (2 drops per mL of 0.5% BSA (according to the manufacturer’s protocol); R37117, Invitrogen). Controls were incubated in 0.5% BSA without the primary antibody. After a final wash step with PBS, cells were resuspended in PBS and processed by flow cytometry (BD LSRFortessa Cell Analyzer; BD Biosciences). Intracellular GFP-producing bacteria were assessed with the 488 nm laser (Blue) with mirror 505LP and filter 530/30 (channel Blue 530/30) at 380 V, pDrp1-bound Alexa Fluor 594-conjugated antibody signal was assessed with the 561 nm laser (Yellow/Green) with mirror 600LP and filter 610/20 (channel YG610/20) at 470 V. Analysis was done with the FlowJo software.

### Bioinformatics and statistics

Protein structure predictions were performed with the Alphafold2 algorithm (Jumper et al, 2021). Each experiment was independently replicated at least three times, and representative images are shown. All statistical analyses were performed using GraphPad Prism version 7.01 for Windows, GraphPad Software, La Jolla, California, USA (www.graphpad.com). Two-tailed Student’s *t*-test or one-way ANOVA was used, and significance levels are defined as follows: *, **, ***, or **** to indicate probability values of less than 0.05, 0.01, 0.001, or 0.0001, respectively.

## Supplementary information

Appendix

Peer Review File

Source data Fig. 1

Source data Fig. 2

Source data Fig. 3

Source data Fig. 4

Source data Fig. 5

Source data Fig. 6

Expanded View Figures

## Data Availability

No datasets have been deposited at external repositories. The source data of this paper are collected in the following database record: biostudies:S-SCDT-10_1038-S44319-026-00823-3.

## References

[CR1] Arasaki K, Mikami Y, Shames SR, Inoue H, Wakana Y, Tagaya M (2017) *Legionella* effector Lpg1137 shuts down ER-mitochondria communication through cleavage of syntaxin 17. Nat Commun 8:15406 28504273 10.1038/ncomms15406 PMC5440676

[CR2] Bärlocher K, Hutter CAJ, Swart AL, Steiner B, Welin A, Hohl M, Letourneur F, Seeger MA, Hilbi H (2017) Structural insights into *Legionella* RidL-Vps29 retromer subunit interaction reveal displacement of the regulator TBC1D5. Nat Commun 8:1543 29146912 10.1038/s41467-017-01512-5 PMC5691146

[CR3] Bonifacino JS, Hurley JH (2008) Retromer. Curr Opin Cell Biol 20:427–436 18472259 10.1016/j.ceb.2008.03.009 PMC2833274

[CR4] Braschi E, Goyon V, Zunino R, Mohanty A, Xu L, McBride HM (2010) Vps35 mediates vesicle transport between the mitochondria and peroxisomes. Curr Biol 20:1310–1315 20619655 10.1016/j.cub.2010.05.066

[CR5] Casadaban MJ, Cohen SN (1980) Analysis of gene control signals by DNA fusion and cloning in *Escherichia coli*. J Mol Biol 138:179–207 6997493 10.1016/0022-2836(80)90283-1

[CR6] Chan DC (2020) Mitochondrial dynamics and its involvement in disease. Ann Rev Pathol 15:235–259 31585519 10.1146/annurev-pathmechdis-012419-032711

[CR7] Chang CR, Blackstone C (2010) Dynamic regulation of mitochondrial fission through modification of the dynamin-related protein Drp1. Ann N Y Acad Sci 1201:34–39 20649536 10.1111/j.1749-6632.2010.05629.x PMC5585781

[CR8] Chang CR, Manlandro CM, Arnoult D, Stadler J, Posey AE, Hill RB, Blackstone C (2010) A lethal de novo mutation in the middle domain of the dynamin-related GTPase Drp1 impairs higher order assembly and mitochondrial division. J Biol Chem 285:32494–32503 20696759 10.1074/jbc.M110.142430 PMC2952251

[CR9] Chaudhry A, Shi R, Luciani DS (2020) A pipeline for multidimensional confocal analysis of mitochondrial morphology, function, and dynamics in pancreatic beta-cells. Am J Physiol Endocrinol Metab 318:E87–E101 31846372 10.1152/ajpendo.00457.2019 PMC7052579

[CR10] Cutillo G, Simon DK, Eleuteri S (2020) VPS35 and the mitochondria: Connecting the dots in Parkinson’s disease pathophysiology. Neurobiol Dis 145:105056 32853677 10.1016/j.nbd.2020.105056

[CR11] Dolezal P, Aili M, Tong J, Jiang JH, Marobbio CM, Lee SF, Schuelein R, Belluzzo S, Binova E, Mousnier A et al (2012) *Legionella pneumophila* secretes a mitochondrial carrier protein during infection. PLoS Pathog 8:e1002459 22241989 10.1371/journal.ppat.1002459 PMC3252375

[CR12] Escoll P, Song OR, Viana F, Steiner B, Lagache T, Olivo-Marin JC, Impens F, Brodin P, Hilbi H, Buchrieser C (2017) *Legionella pneumophila* modulates mitochondrial dynamics to trigger metabolic repurposing of infected macrophages. Cell Host Microbe 22:302–316.e307 28867389 10.1016/j.chom.2017.07.020

[CR13] Finsel I, Ragaz C, Hoffmann C, Harrison CF, Weber S, van Rahden VA, Johannes L, Hilbi H (2013) The *Legionella* effector RidL inhibits retrograde trafficking to promote intracellular replication. Cell Host Microbe 14:38–50 23870312 10.1016/j.chom.2013.06.001

[CR14] Friedman JR, Lackner LL, West M, DiBenedetto JR, Nunnari J, Voeltz GK (2011) ER tubules mark sites of mitochondrial division. Science 334:358–362 21885730 10.1126/science.1207385 PMC3366560

[CR15] Fu J, Zhou M, Gritsenko MA, Nakayasu ES, Song L, Luo ZQ (2022) *Legionella pneumophila* modulates host energy metabolism by ADP-ribosylation of ADP/ATP translocases. eLife 11:e73611 35084332 10.7554/eLife.73611 PMC8820735

[CR16] Ganesan V, Willis SD, Chang KT, Beluch S, Cooper KF, Strich R (2019) Cyclin C directly stimulates Drp1 GTP affinity to mediate stress-induced mitochondrial hyperfission. Mol Biol Cell 30:302–311 30516433 10.1091/mbc.E18-07-0463 PMC6589575

[CR17] Garcia-Rodriguez FJ, Buchrieser C, Escoll P (2023) *Legionella* and mitochondria, an intriguing relationship. Int Rev Cell Mol Biol 374:37–81 36858656 10.1016/bs.ircmb.2022.10.001

[CR18] Geertsma ER, Dutzler R (2011) A versatile and efficient high-throughput cloning tool for structural biology. Biochemistry 50:3272–3278 21410291 10.1021/bi200178z

[CR19] Giacomello M, Pyakurel A, Glytsou C, Scorrano L (2020) The cell biology of mitochondrial membrane dynamics. Nat Rev Mol Cell Biol 21:204–224 32071438 10.1038/s41580-020-0210-7

[CR20] Hilbi H, Buchrieser C (2022) Microbe profile: *Legionella pneumophila*—a copycat eukaryote. Microbiology 10.1099/mic.1090.001142 10.1099/mic.0.001142 35230931

[CR21] Hüsler D, Stauffer P, Hilbi H (2023a) Tapping lipid droplets: A rich fat diet of intracellular bacterial pathogens. Mol Microbiol 120:194–209 37429596 10.1111/mmi.15120

[CR22] Hüsler D, Stauffer P, Keller B, Bock D, Steiner T, Ostrzinski A, Vormittag S, Striednig B, Swart AL, Letourneur F et al (2023b) The large GTPase Sey1/atlastin mediates lipid droplet- and FadL-dependent intracellular fatty acid metabolism of *Legionella pneumophila*. eLife 12:e85142 37158597 10.7554/eLife.85142 PMC10259473

[CR23] Hüsler D, Steiner B, Welin A, Striednig B, Swart AL, Molle V, Hilbi H, Letourneur F (2021) *Dictyostelium* lacking the single atlastin homolog Sey1 shows aberrant ER architecture, proteolytic processes and expansion of the *Legionella*-containing vacuole. Cell Microbiol 23:e13318 33583106 10.1111/cmi.13318

[CR24] Ingerman E, Nunnari J (2005) A continuous, regenerative coupled GTPase assay for dynamin-related proteins. Methods Enzymol 404:611–619 16413304 10.1016/S0076-6879(05)04053-X

[CR25] Jumper J, Evans R, Pritzel A, Green T, Figurnov M, Ronneberger O, Tunyasuvunakool K, Bates R, Žídek A, Potapenko A et al (2021) Highly accurate protein structure prediction with AlphaFold. Nature 596:583–589 34265844 10.1038/s41586-021-03819-2 PMC8371605

[CR26] Kalia R, Wang RY, Yusuf A, Thomas PV, Agard DA, Shaw JM, Frost A (2018) Structural basis of mitochondrial receptor binding and constriction by DRP1. Nature 558:401–405 29899447 10.1038/s41586-018-0211-2 PMC6120343

[CR27] Katic A, Hüsler D, Letourneur F, Hilbi H (2021) *Dictyostelium* dynamin superfamily GTPases implicated in vesicle trafficking and host-pathogen interactions. Front Cell Dev Biol 9:731964 34746129 10.3389/fcell.2021.731964 PMC8565484

[CR28] Kong L, Sochacki KA, Wang H, Fang S, Canagarajah B, Kehr AD, Rice WJ, Strub MP, Taraska JW, Hinshaw JE (2018) Cryo-EM of the dynamin polymer assembled on lipid membrane. Nature 560:258–262 30069048 10.1038/s41586-018-0378-6 PMC6121775

[CR29] König T, McBride HM (2024) Mitochondrial-derived vesicles in metabolism, disease, and aging. Cell Metab 36:21–35 38171335 10.1016/j.cmet.2023.11.014

[CR30] König T, Nolte H, Aaltonen MJ, Tatsuta T, Krols M, Stroh T, Langer T, McBride HM (2021) MIROs and DRP1 drive mitochondrial-derived vesicle biogenesis and promote quality control. Nat Cell Biol 23:1271–1286 34873283 10.1038/s41556-021-00798-4

[CR31] Kubori T, Lee J, Kim H, Yamazaki K, Nishikawa M, Kitao T, Oh BH, Nagai H (2022) Reversible modification of mitochondrial ADP/ATP translocases by paired *Legionella* effector proteins. Proc Natl Acad Sci USA 119:e2122872119 35653564 10.1073/pnas.2122872119 PMC9191684

[CR32] Legland D, Arganda-Carreras I, Andrey P (2016) MorphoLibJ: integrated library and plugins for mathematical morphology with ImageJ. Bioinformatics 32:3532–3534 27412086 10.1093/bioinformatics/btw413

[CR33] Liu R, Chan DC (2015) The mitochondrial fission receptor Mff selectively recruits oligomerized Drp1. Mol Biol Cell 26:4466–4477 26446846 10.1091/mbc.E15-08-0591 PMC4666140

[CR34] Macdonald PJ, Stepanyants N, Mehrotra N, Mears JA, Qi X, Sesaki H, Ramachandran R (2014) A dimeric equilibrium intermediate nucleates Drp1 reassembly on mitochondrial membranes for fission. Mol Biol Cell 25:1905–1915 24790094 10.1091/mbc.E14-02-0728 PMC4055269

[CR35] McLelland GL, Lee SA, McBride HM, Fon EA (2016) Syntaxin-17 delivers PINK1/parkin-dependent mitochondrial vesicles to the endolysosomal system. J Cell Biol 214:275–291 27458136 10.1083/jcb.201603105 PMC4970327

[CR36] Mears JA, Hinshaw JE (2008) Visualization of dynamins. Methods Cell Biol 88:237–256 18617037 10.1016/S0091-679X(08)00413-5 PMC2692555

[CR37] Michaelis S, Gomez-Valero L, Chen T, Schmid C, Buchrieser C, Hilbi H (2024) Small molecule communication of *Legionella*: the ins and outs of autoinducer and nitric oxide signaling. Microbiol Mol Biol Rev 88:e0009723 39162424 10.1128/mmbr.00097-23 PMC11426016

[CR38] Mondino S, Schmidt S, Rolando M, Escoll P, Gomez-Valero L, Buchrieser C (2020) Legionnaires’ disease: state of the art knowledge of pathogenesis mechanisms of *Legionella*. Ann Rev Pathol 15:439–466 31657966 10.1146/annurev-pathmechdis-012419-032742

[CR39] Morgenstern M, Peikert CD, Lubbert P, Suppanz I, Klemm C, Alka O, Steiert C, Naumenko N, Schendzielorz A, Melchionda L et al (2021) Quantitative high-confidence human mitochondrial proteome and its dynamics in cellular context. Cell Metab 33:2464–2483.e2418 34800366 10.1016/j.cmet.2021.11.001 PMC8664129

[CR40] Newton HJ, Ang DK, van Driel IR, Hartland EL (2010) Molecular pathogenesis of infections caused by *Legionella pneumophila*. Clin Microbiol Rev 23:274–298 20375353 10.1128/CMR.00052-09 PMC2863363

[CR41] Personnic N, Bärlocher K, Finsel I, Hilbi H (2016) Subversion of retrograde trafficking by translocated pathogen effectors. Trends Microbiol 24:450–462 26924068 10.1016/j.tim.2016.02.003

[CR42] Qiu J, Luo ZQ (2017) *Legionella* and *Coxiella* effectors: strength in diversity and activity. Nat Rev Microbiol 15:591–605 28713154 10.1038/nrmicro.2017.67

[CR43] Quiles JM, Gustafsson AB (2022) The role of mitochondrial fission in cardiovascular health and disease. Nat Rev Cardiol 19:723–736 35523864 10.1038/s41569-022-00703-y PMC10584015

[CR44] Romano-Moreno M, Rojas AL, Williamson CD, Gershlick DC, Lucas M, Isupov MN, Bonifacino JS, Machner MP, Hierro A (2017) Molecular mechanism for the subversion of the retromer coat by the *Legionella* effector RidL. Proc Natl Acad Sci USA 114:E11151–E11160 29229824 10.1073/pnas.1715361115 PMC5748213

[CR45] Rothmeier E, Pfaffinger G, Hoffmann C, Harrison CF, Grabmayr H, Repnik U, Hannemann M, Wölke S, Bausch A, Griffiths G et al (2013) Activation of Ran GTPase by a *Legionella* effector promotes microtubule polymerization, pathogen vacuole motility and infection. PLoS Pathog 9:e1003598 24068924 10.1371/journal.ppat.1003598 PMC3777869

[CR46] Sadosky AB, Wiater LA, Shuman HA (1993) Identification of *Legionella pneumophila* genes required for growth within and killing of human macrophages. Infect Immun 61:5361–5373 8225610 10.1128/iai.61.12.5361-5373.1993 PMC281323

[CR47] Segal G, Shuman HA (1998) Intracellular multiplication and human macrophage killing by *Legionella pneumophila* are inhibited by conjugal components of IncQ plasmid RSF1010. Mol Microbiol 30:197–208 9786196 10.1046/j.1365-2958.1998.01054.x

[CR48] Sharma A, Smith HJ, Yao P, Mair WB (2019) Causal roles of mitochondrial dynamics in longevity and healthy aging. EMBO Rep 20:e48395 31667999 10.15252/embr.201948395 PMC6893295

[CR49] Smirnova E, Griparic L, Shurland DL, van der Bliek AM (2001) Dynamin-related protein Drp1 is required for mitochondrial division in mammalian cells. Mol Biol Cell 12:2245–2256 11514614 10.1091/mbc.12.8.2245 PMC58592

[CR50] Smoyer CJ, Katta SS, Gardner JM, Stoltz L, McCroskey S, Bradford WD, McClain M, Smith SE, Slaughter BD, Unruh JR et al (2016) Analysis of membrane proteins localizing to the inner nuclear envelope in living cells. J Cell Biol 215:575–590 27831485 10.1083/jcb.201607043 PMC5119940

[CR51] Song W, Chen J, Petrilli A, Liot G, Klinglmayr E, Zhou Y, Poquiz P, Tjong J, Pouladi MA, Hayden MR et al (2011) Mutant huntingtin binds the mitochondrial fission GTPase dynamin-related protein-1 and increases its enzymatic activity. Nat Med 17:377–382 21336284 10.1038/nm.2313 PMC3051025

[CR52] Soubannier V, Rippstein P, Kaufman BA, Shoubridge EA, McBride HM (2012) Reconstitution of mitochondria derived vesicle formation demonstrates selective enrichment of oxidized cargo. PLoS ONE 7:e52830 23300790 10.1371/journal.pone.0052830 PMC3530470

[CR53] Steiner B, Swart AL, Hilbi H (2019) Perturbation of *Legionella* cell infection by RNA interference. Methods Mol Biol 1921:221–238 30694495 10.1007/978-1-4939-9048-1_14

[CR54] Steiner B, Swart AL, Welin A, Weber S, Personnic N, Kaech A, Freyre C, Ziegler U, Klemm RW, Hilbi H (2017) ER remodeling by the large GTPase atlastin promotes vacuolar growth of *Legionella pneumophila*. EMBO Rep 18:1817–1836 28835546 10.15252/embr.201743903 PMC5623866

[CR55] Steiner B, Weber S, Hilbi H (2018) Formation of the *Legionella*-containing vacuole: phosphoinositide conversion, GTPase modulation and ER dynamics. Int J Med Microbiol 308:49–57 28865995 10.1016/j.ijmm.2017.08.004

[CR56] Sugiura A, McLelland GL, Fon EA, McBride HM (2014) A new pathway for mitochondrial quality control: mitochondrial-derived vesicles. EMBO J 33:2142–2156 25107473 10.15252/embj.201488104 PMC4282503

[CR57] Swart AL, Harrison CF, Eichinger L, Steinert M, Hilbi H (2018) *Acanthamoeba* and *Dictyostelium* as cellular models for *Legionella* infection. Front Cell Infect Microbiol 8:61 29552544 10.3389/fcimb.2018.00061 PMC5840211

[CR58] Swart AL, Steiner B, Gomez-Valero L, Schütz S, Hannemann M, Janning P, Irminger M, Rothmeier E, Buchrieser C, Itzen A et al (2020) Divergent evolution of *Legionella* RCC1 repeat effectors defines the range of Ran GTPase cycle targets. mBio 11:e00405–20 32209684 10.1128/mBio.00405-20 PMC7157520

[CR59] Tiaden A, Spirig T, Weber SS, Brüggemann H, Bosshard R, Buchrieser C, Hilbi H (2007) The *L. pneumophila* response regulator LqsR promotes host cell interactions as an element of the virulence regulatory network controlled by RpoS and LetA. Cell Microbiol 9:2903–2920 17614967 10.1111/j.1462-5822.2007.01005.x

[CR60] Vormittag S, Hüsler D, Haneburger I, Kröniger T, Anand A, Prantl M, Barisch C, Maass S, Becher D, Letourneur F et al (2023) *Legionella*- and host-driven lipid flux at LCV-ER membrane contact sites promotes vacuole remodeling. EMBO Rep 24:e56007 36588479 10.15252/embr.202256007 PMC9986823

[CR61] Wang W, Ma X, Zhou L, Liu J, Zhu X (2017) A conserved retromer sorting motif is essential for mitochondrial DLP1 recycling by VPS35 in Parkinson’s disease model. Hum Mol Genet 26:781–789 28040727 10.1093/hmg/ddw430 PMC5903416

[CR62] Wang W, Wang X, Fujioka H, Hoppel C, Whone AL, Caldwell MA, Cullen PJ, Liu J, Zhu X (2016) Parkinson’s disease-associated mutant VPS35 causes mitochondrial dysfunction by recycling DLP1 complexes. Nat Med 22:54–63 26618722 10.1038/nm.3983 PMC4826611

[CR63] Weber S, Steiner B, Welin A, Hilbi H (2018) *Legionella*-containing vacuoles capture PtdIns(4)*P*-rich vesicles derived from the Golgi apparatus. mBio 9:e0242018 10.1128/mBio.02420-18 PMC6299486 30538188

[CR64] Weber SS, Ragaz C, Reus K, Nyfeler Y, Hilbi H (2006) *Legionella pneumophila* exploits PI(4)*P* to anchor secreted effector proteins to the replicative vacuole. PLoS Pathog 2:e46 16710455 10.1371/journal.ppat.0020046 PMC1463015

[CR65] Yao J, Yang F, Sun X, Wang S, Gan N, Liu Q, Liu D, Zhang X, Niu D, Wei Y et al (2018) Mechanism of inhibition of retromer transport by the bacterial effector RidL. Proc Natl Acad Sci USA 115:E1446–E1454 29386389 10.1073/pnas.1717383115 PMC5816186

[CR66] Yek KQ, Hodgson ER, Ang CS, Palmer CS, Frazier AE, Newton HJ, Stojanovski D (2025) *Legionella* effector LpPIP recruits protein phosphatase 1 to the mitochondria to induce dephosphorylation of outer membrane proteins. PLoS Biol 23:e3003261 40700448 10.1371/journal.pbio.3003261 PMC12313075

[CR67] Zong WX, Rabinowitz JD, White E (2016) Mitochondria and cancer. Mol Cell 61:667–676 26942671 10.1016/j.molcel.2016.02.011 PMC4779192

