# Supplementary material for: The Legionella effector RidL binds the large fission GTPase Drp1 to promote mitochondrial fragmentation
Source: EMBO Rep. 2026 Jun 6;27(13):3759–87. doi: 10.1038/s44319-026-00823-3 (PMC13354802; doi:10.1038/s44319-026-00823-3)
Supplement: Supplementary file 1 — Appendix [file 44319_2026_823_MOESM1_ESM.pdf]

Appendix for:

The *Legionella* effector RidL binds the large fission GTPase Drp1 to promote mitochondrial fragmentation

Table of Contents

Appendix Figure S1. Raw microscopy images of Drp1 and Tom20 immunostaining in *L. pneumophila*-infected cells. p. 2

Appendix Figure S2. Raw microscopy images of phospho-Drp1 immunostaining in *L. pneumophila*-infected cells. p. 3

Appendix Figure S1

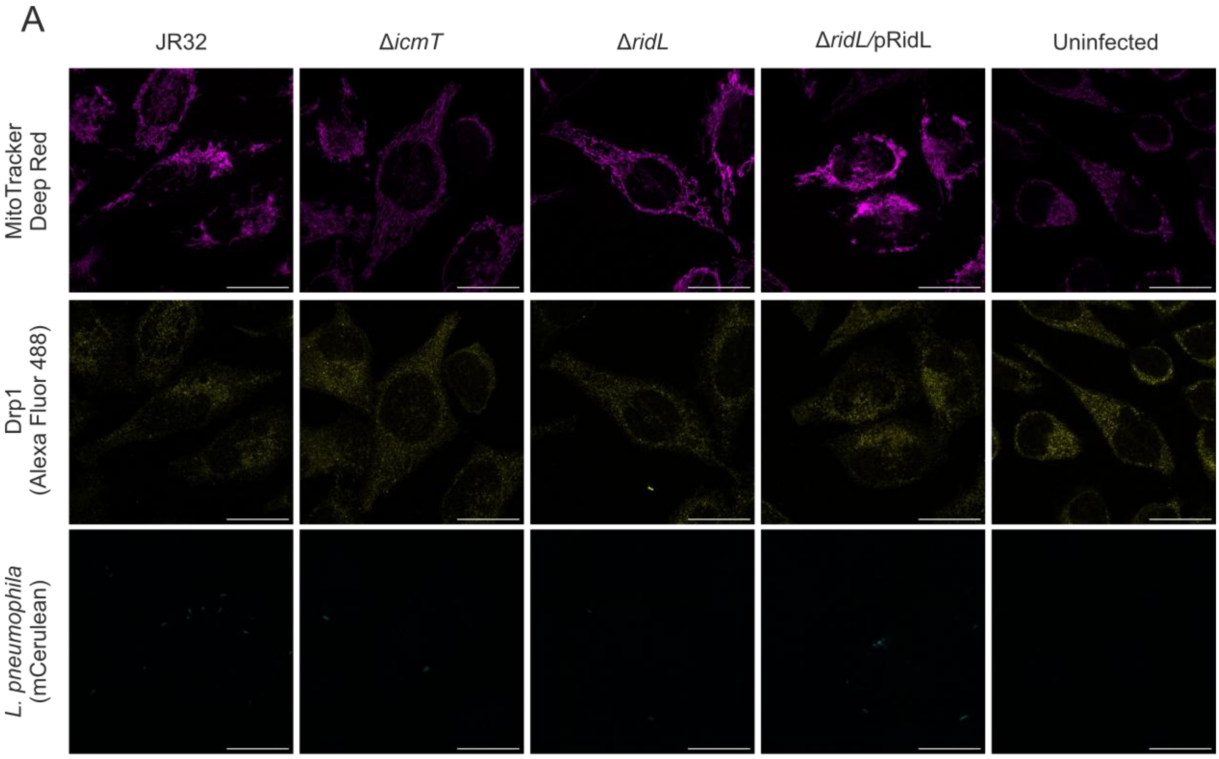

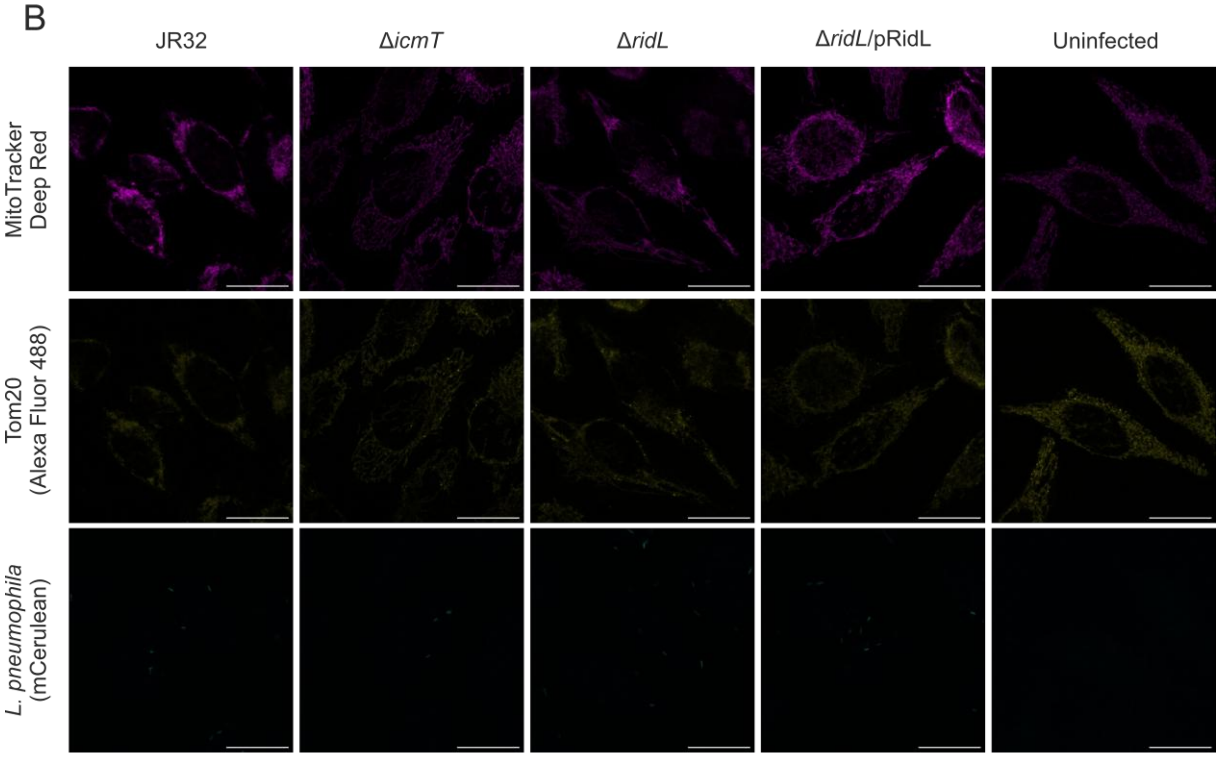

Appendix Figure S1. Raw microscopy images of Drp1 and Tom20 immunostaining in *L. pneumophila*-infected cells. Images show the individual channels for MitoTracker Deep Red, Alexa Fluor 488 of (**A**) Drp1 or (**B**) Tom20, and mCerulean (*L. pneumophila*, pNP99), corresponding to **Fig. 4E** or **Fig. 4F**, respectively. Original signal intensities are shown without adjustment of brightness or contrast. Scale bars, 20 µm.

Appendix Figure S2

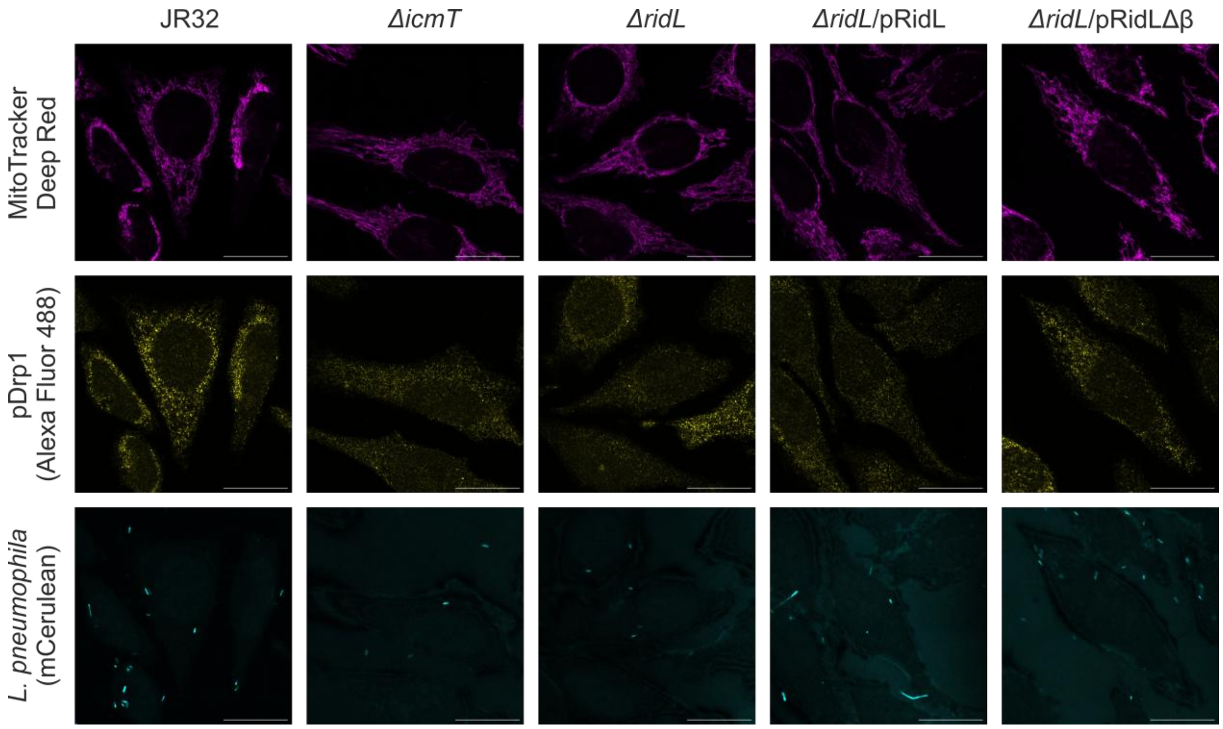

Appendix Figure S2. Raw microscopy images of phospho-Drp1 immunostaining in *L. pneumophila*-infected cells. Images show the individual channels for MitoTracker Deep Red, Alexa Fluor 488 of phospho-Drp1 (Ser616), and mCerulean (*L. pneumophila*; pNP99, pKB208, or pKB209), corresponding to **Fig. 6A**. Signal intensities with adjustment of brightness and contrast are shown. Scale bars, 20 µm.
