# Supplementary figures and images for: The Legionella effector RidL binds the large fission GTPase Drp1 to promote mitochondrial fragmentation
Source: EMBO Rep. 2026 Jun 6;27(13):3759–87. doi: 10.1038/s44319-026-00823-3 (PMC13354802; doi:10.1038/s44319-026-00823-3)

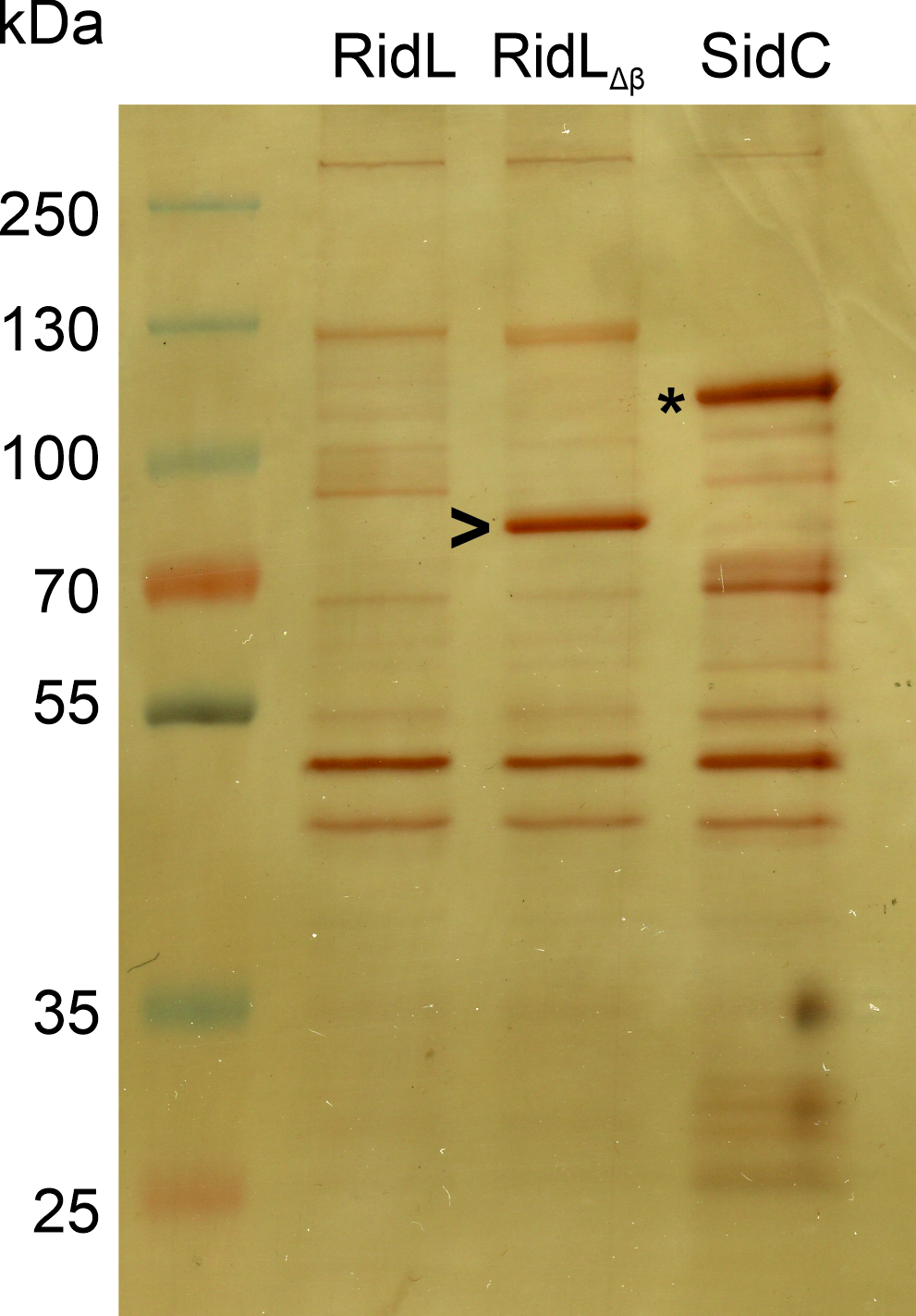

Supplement: Supplementary file 3 — Source data Fig. 1 [file 44319_2026_823_MOESM3_ESM.zip › Fig. 1/1B/silver stain pulldown.tif]

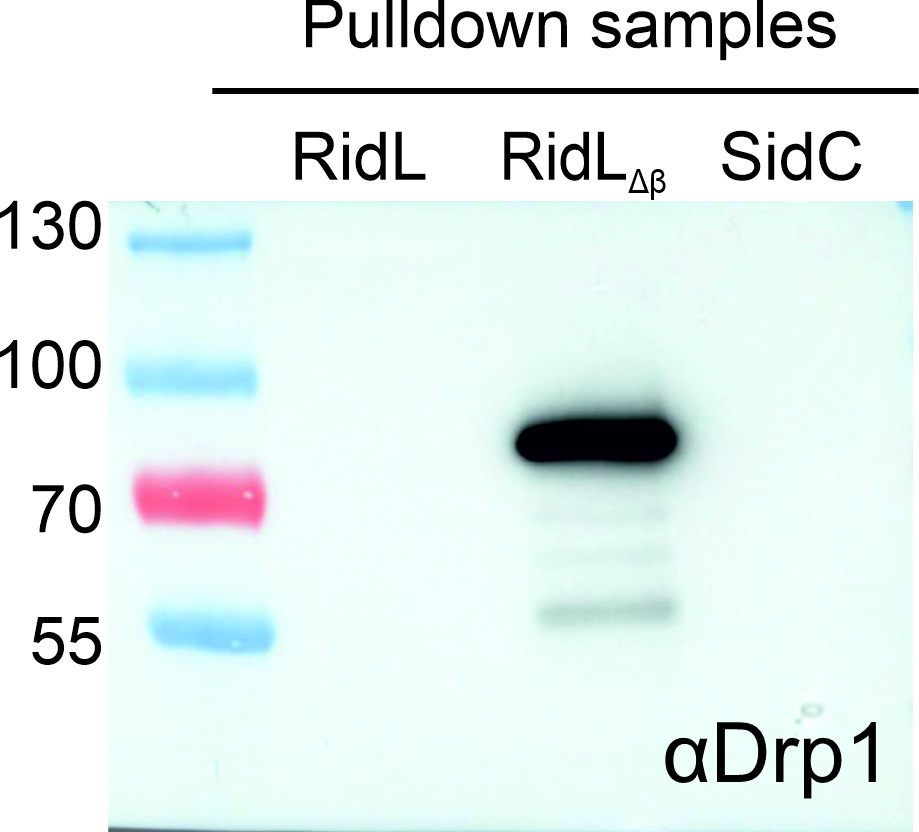

Supplement: Supplementary file 3 — Source data Fig. 1 [file 44319_2026_823_MOESM3_ESM.zip › Fig. 1/1C/western blot Drp1.tif]

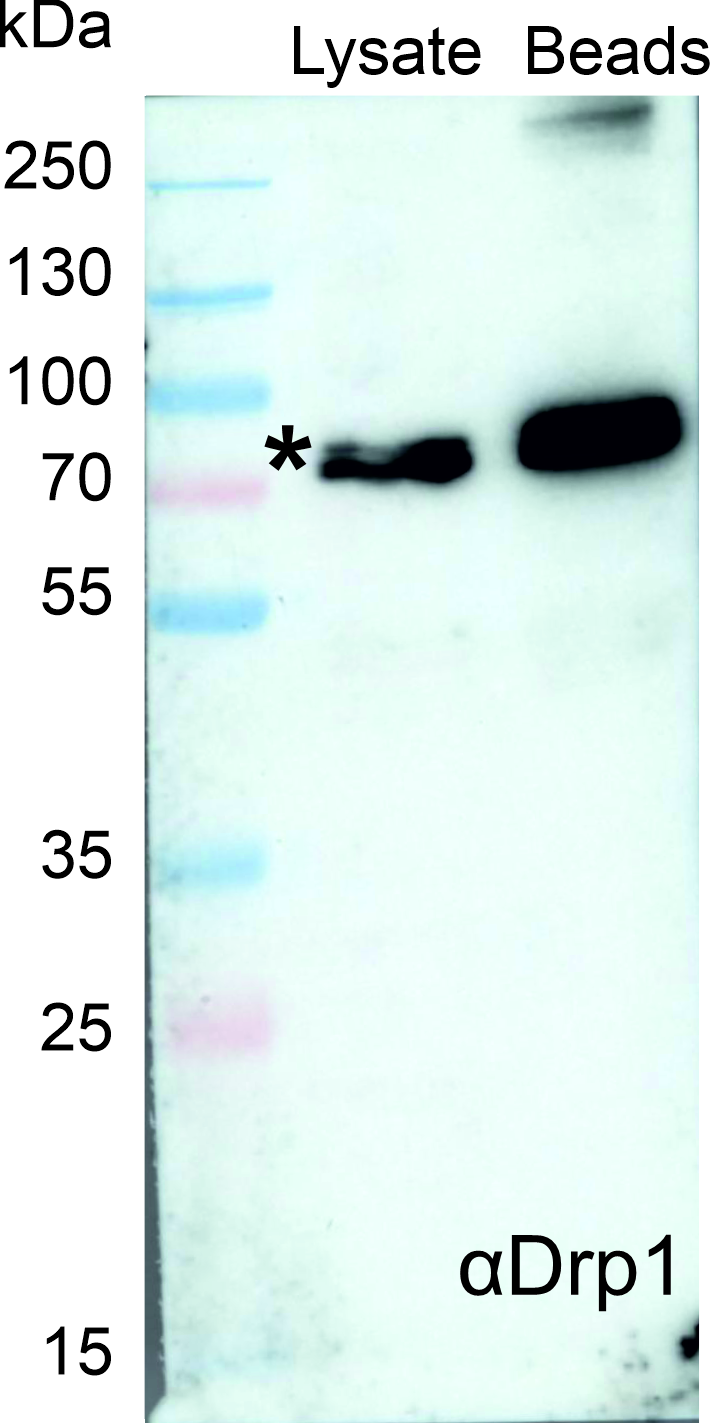

Supplement: Supplementary file 3 — Source data Fig. 1 [file 44319_2026_823_MOESM3_ESM.zip › Fig. 1/1D/western blot Drp1.tif]

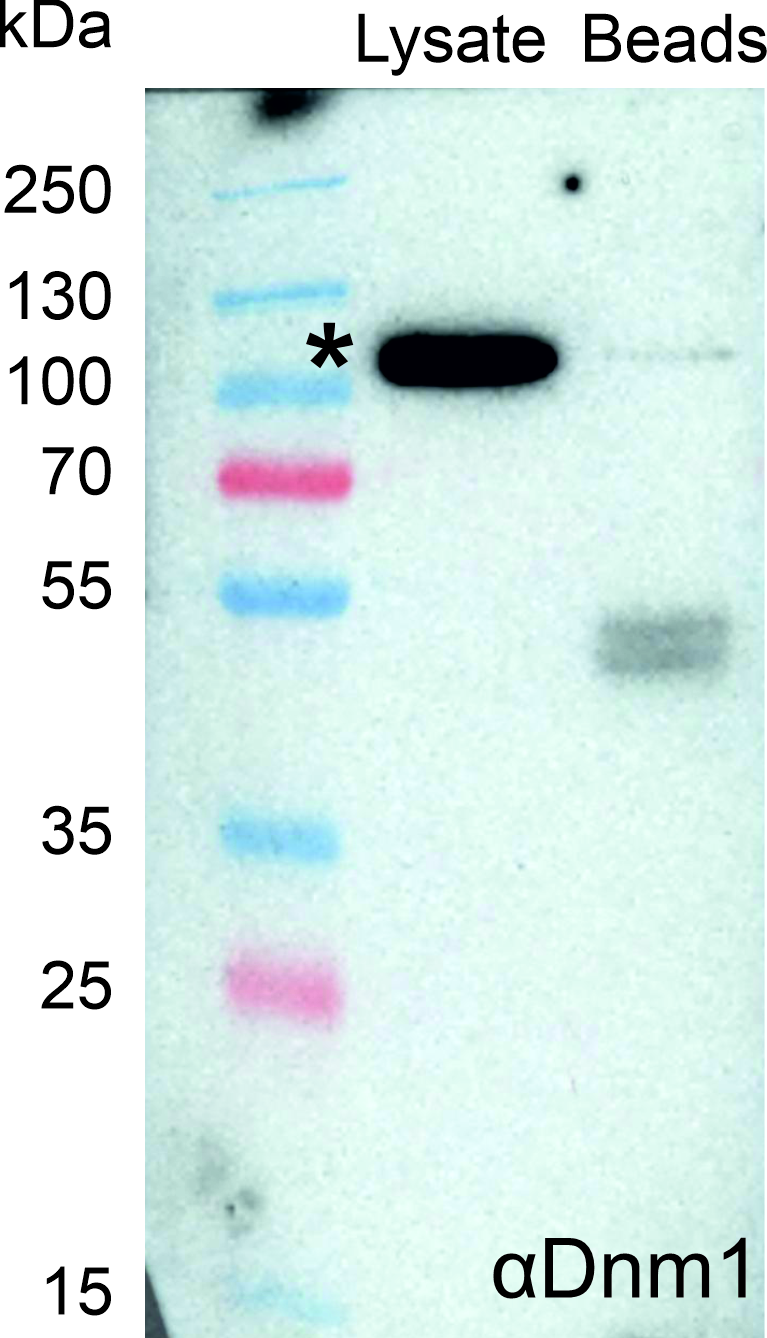

Supplement: Supplementary file 3 — Source data Fig. 1 [file 44319_2026_823_MOESM3_ESM.zip › Fig. 1/1D/western blot Dynamin 1.tif]

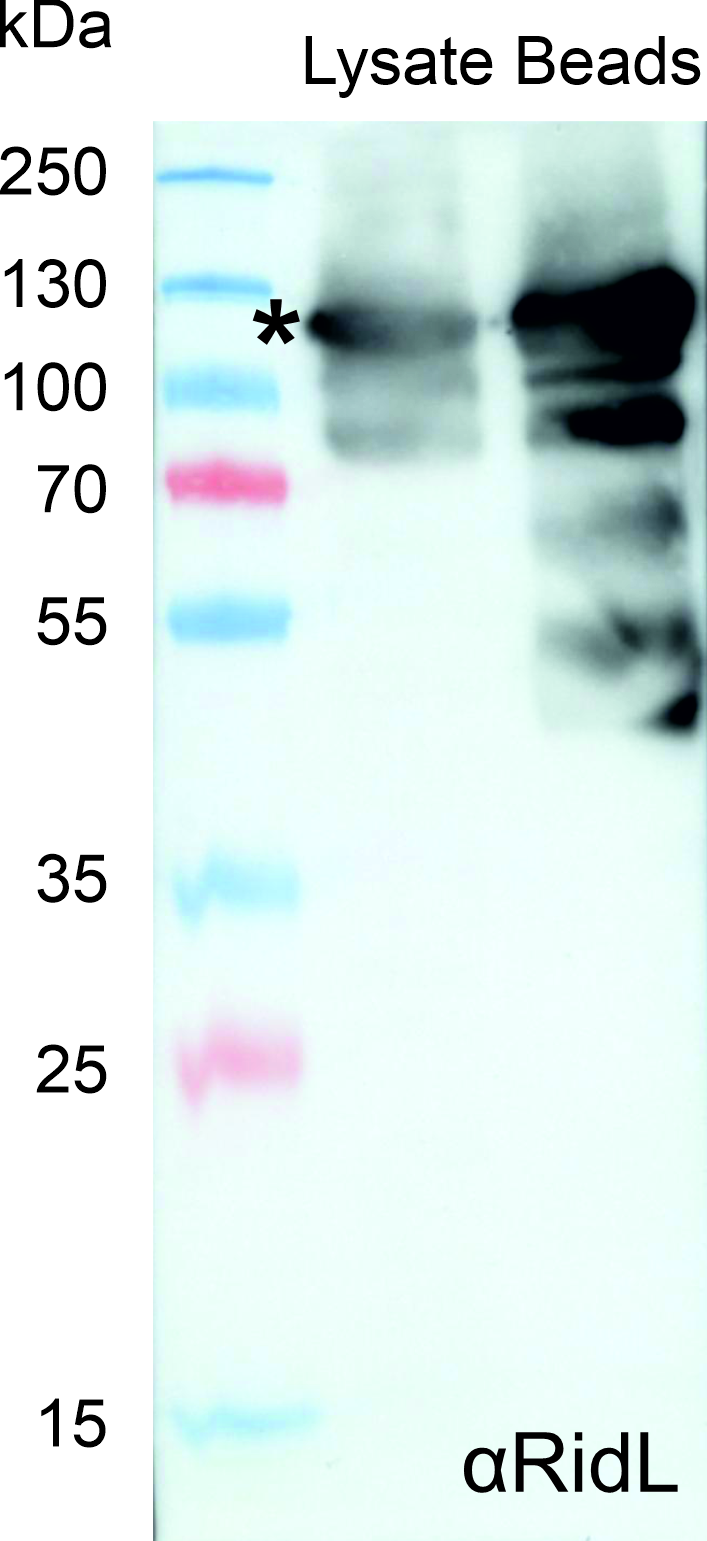

Supplement: Supplementary file 3 — Source data Fig. 1 [file 44319_2026_823_MOESM3_ESM.zip › Fig. 1/1D/western blot RidL.tif]

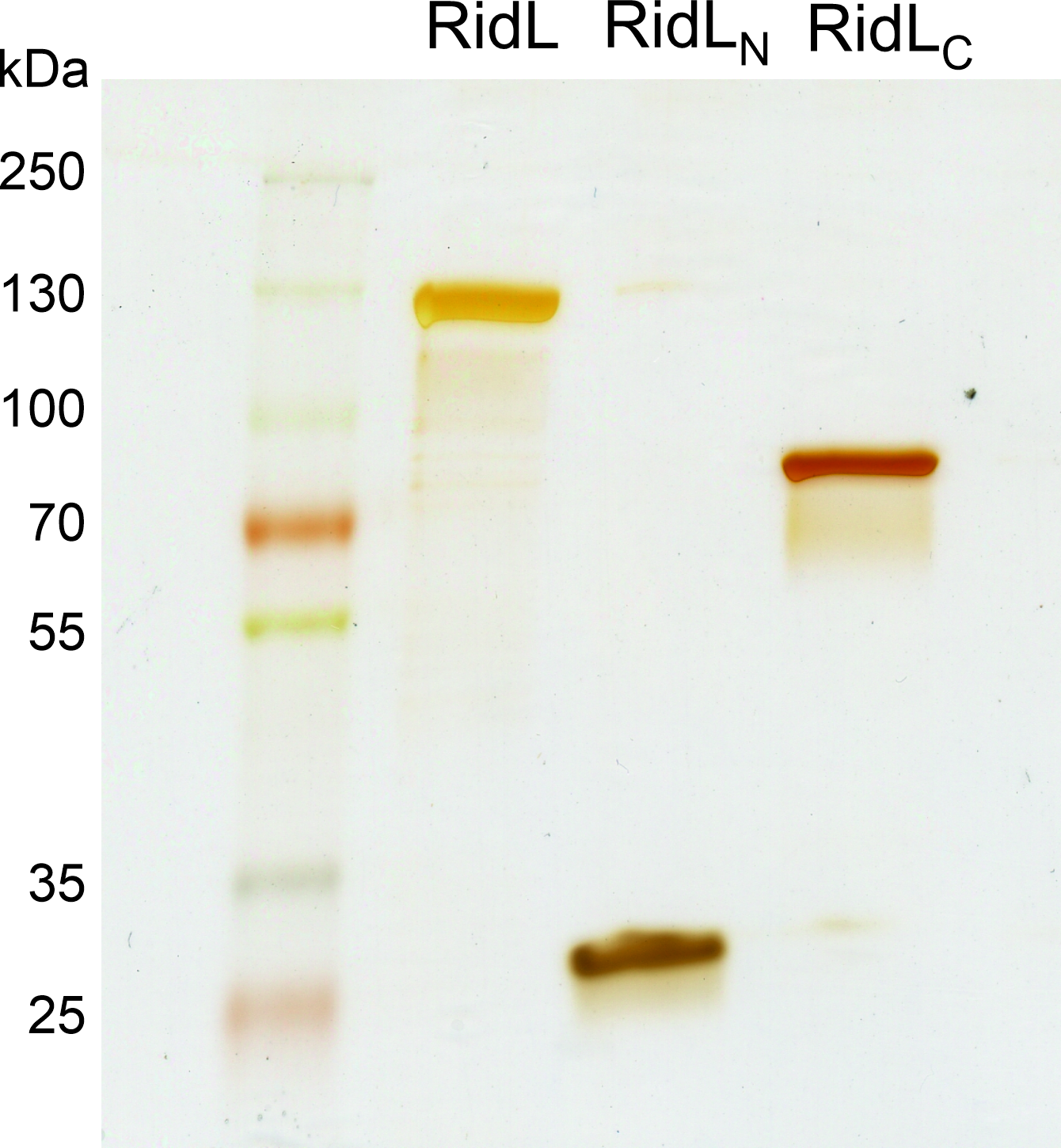

Supplement: Supplementary file 3 — Source data Fig. 1 [file 44319_2026_823_MOESM3_ESM.zip › Fig. 1/1E/silver stain input.tif]

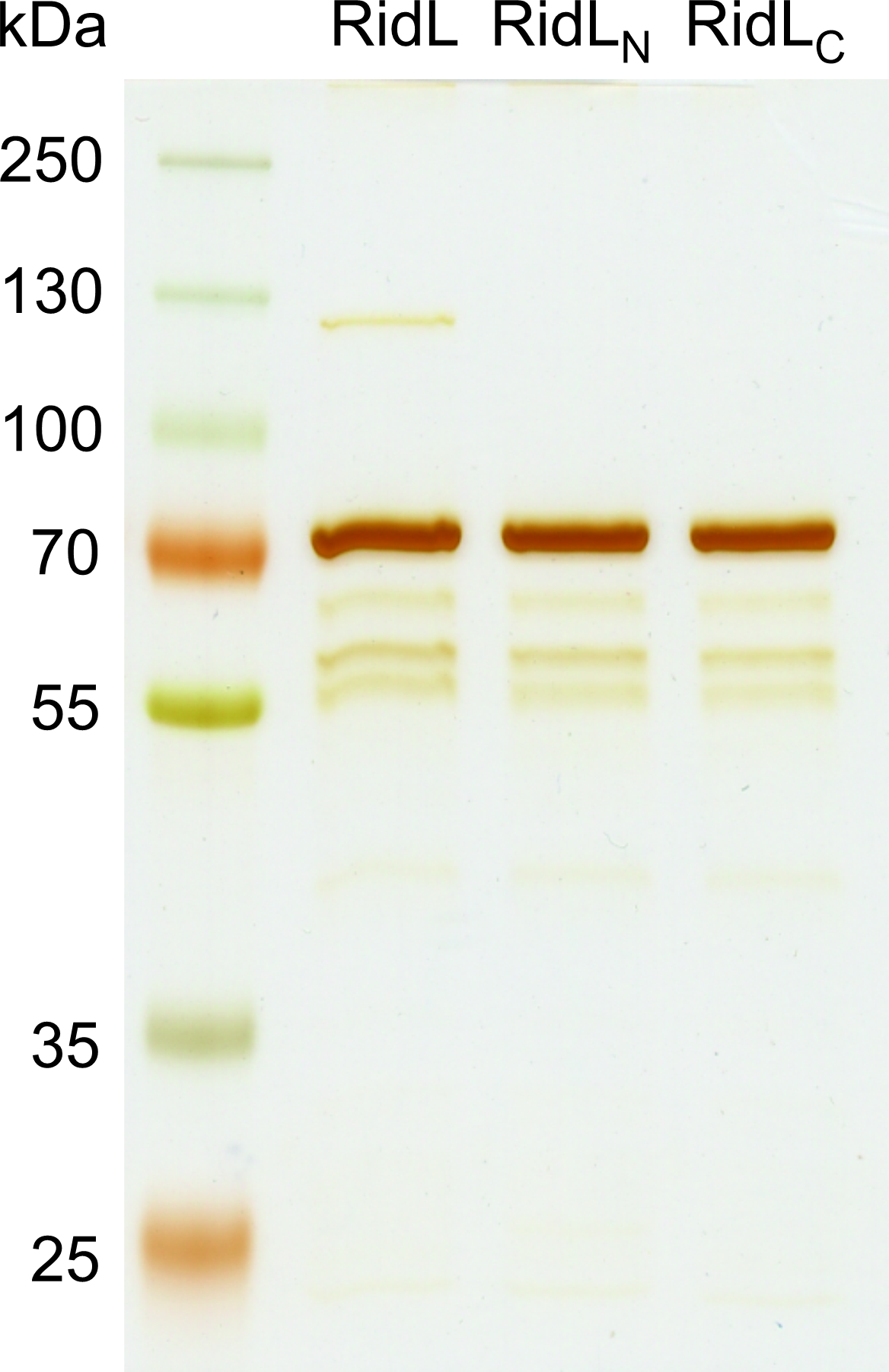

Supplement: Supplementary file 3 — Source data Fig. 1 [file 44319_2026_823_MOESM3_ESM.zip › Fig. 1/1F/silver stain pulldown.tif]

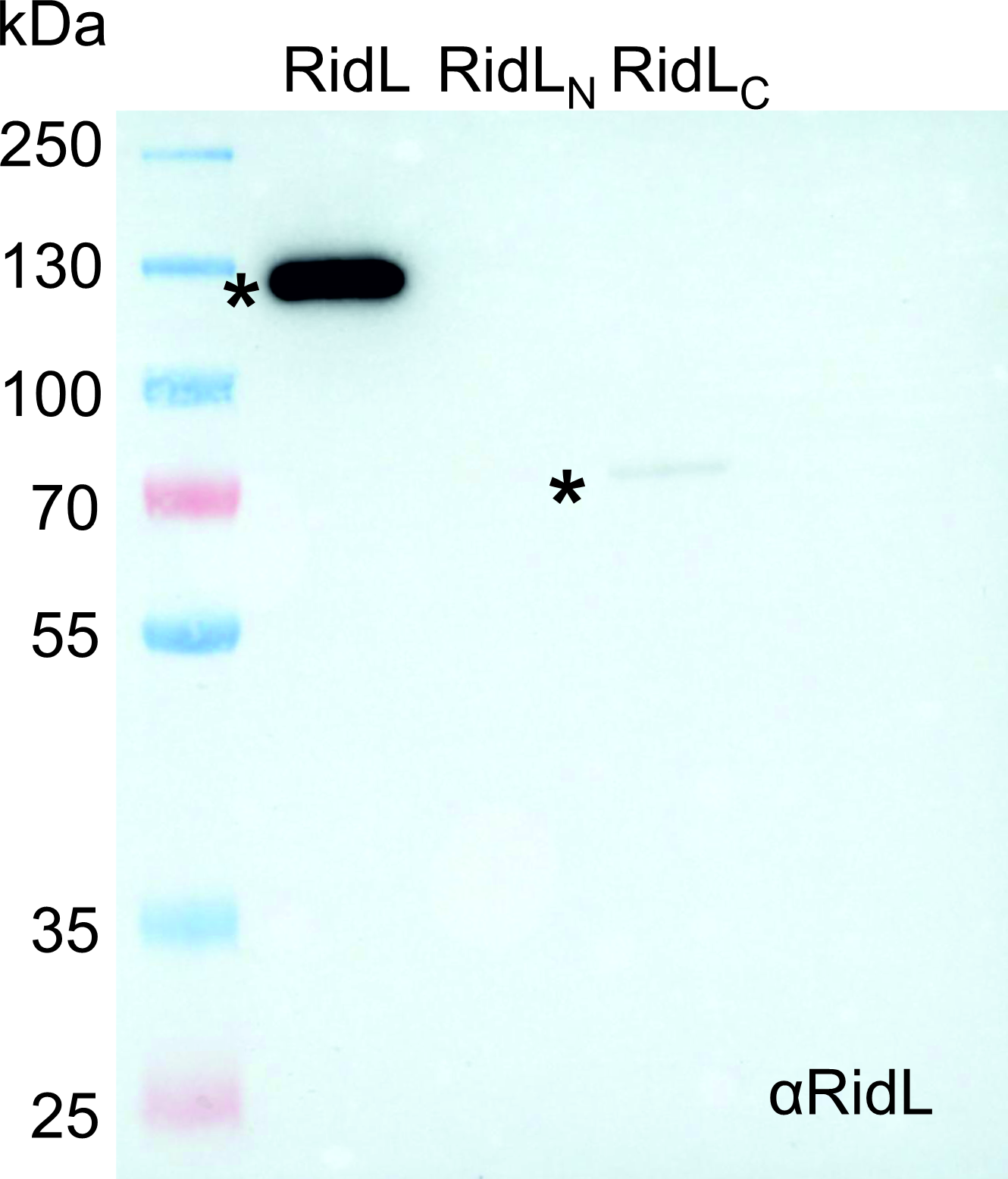

Supplement: Supplementary file 3 — Source data Fig. 1 [file 44319_2026_823_MOESM3_ESM.zip › Fig. 1/1G/western blot RidL.tif]

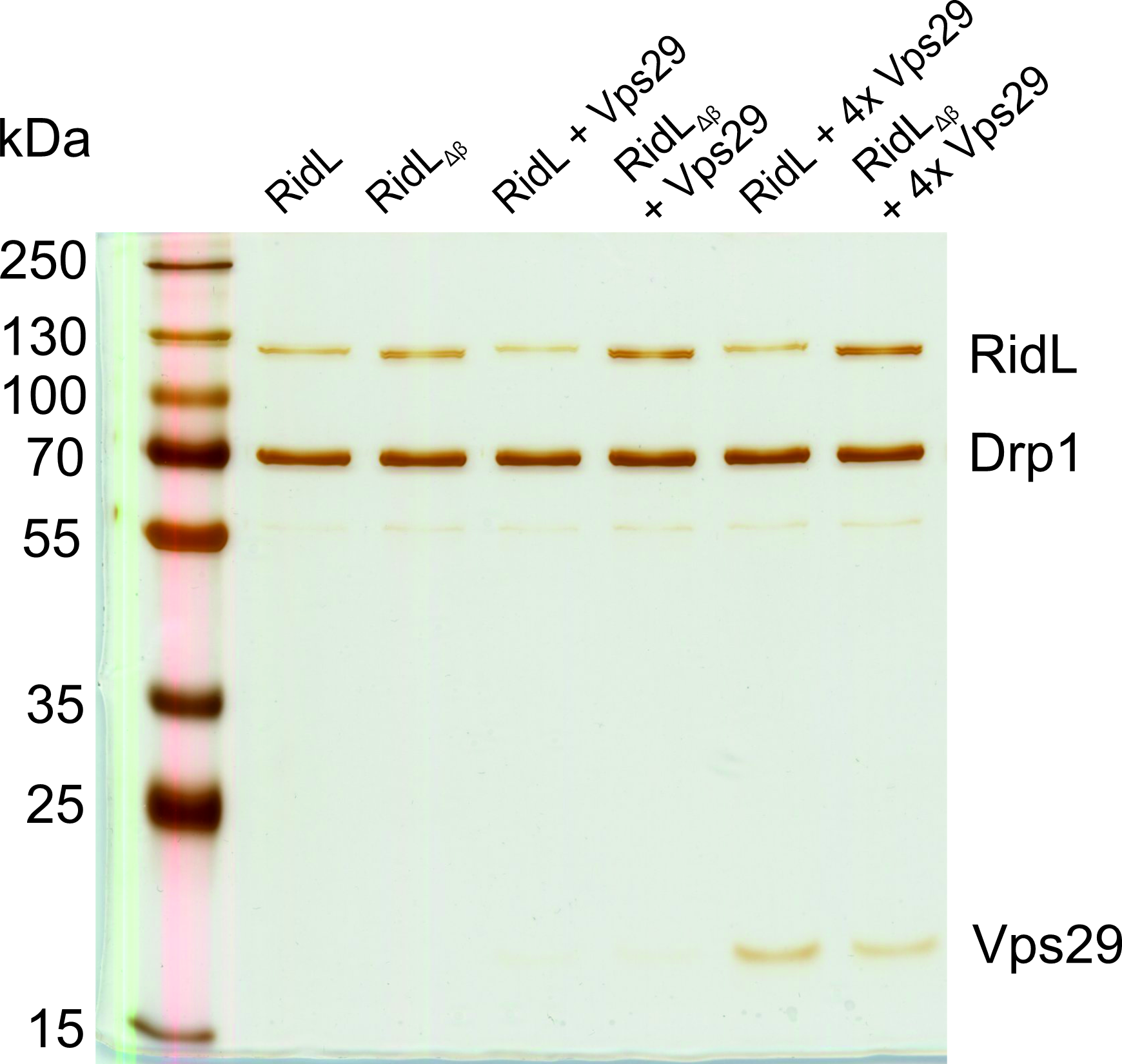

Supplement: Supplementary file 3 — Source data Fig. 1 [file 44319_2026_823_MOESM3_ESM.zip › Fig. 1/1H/silver stain pulldown.tif]

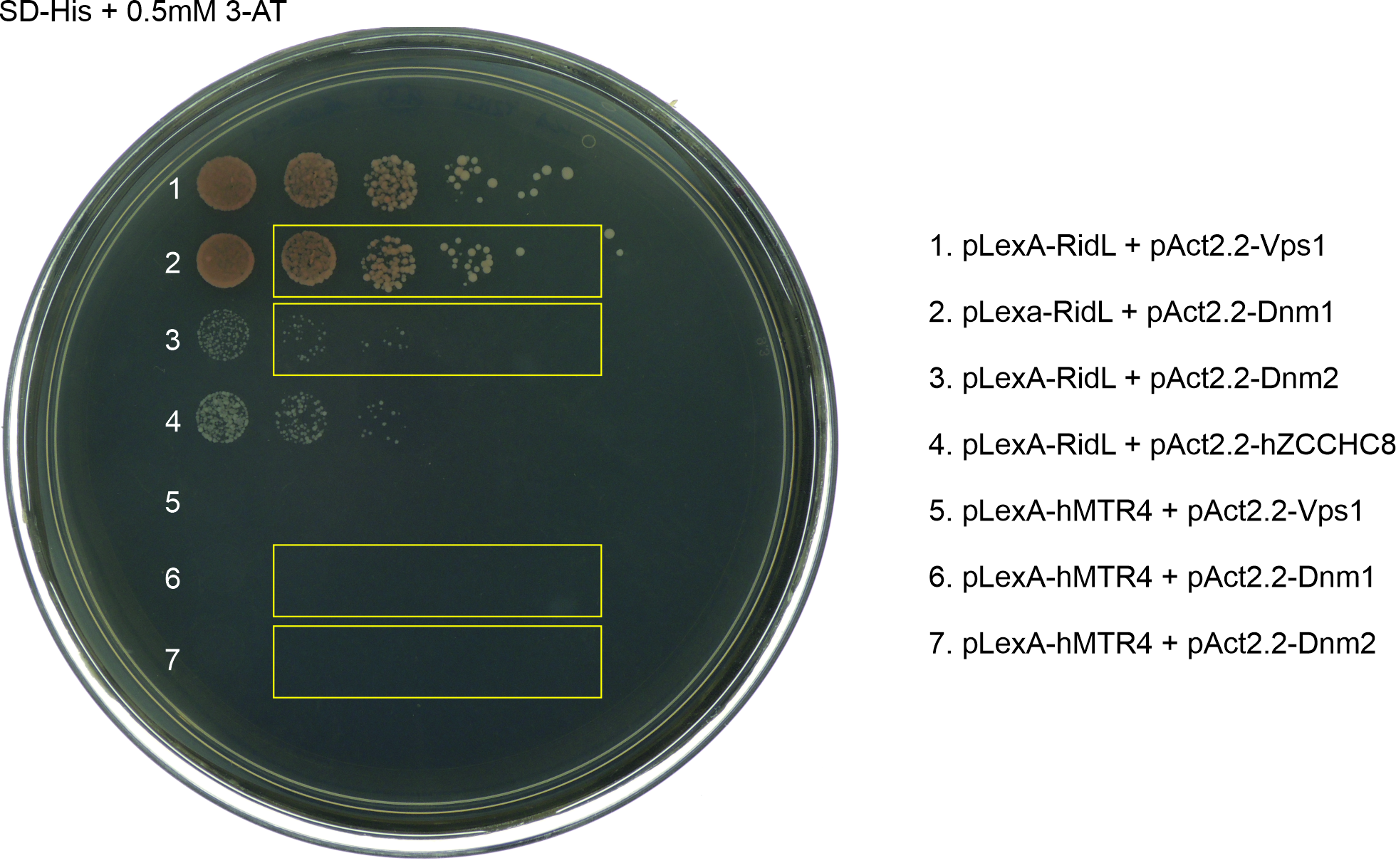

Supplement: Supplementary file 5 — Source data Fig. 3 [file 44319_2026_823_MOESM5_ESM.zip › Fig. 3/3A/SD-His + 0.5mM 3-AT (Dnm1, Dnm2).tif]

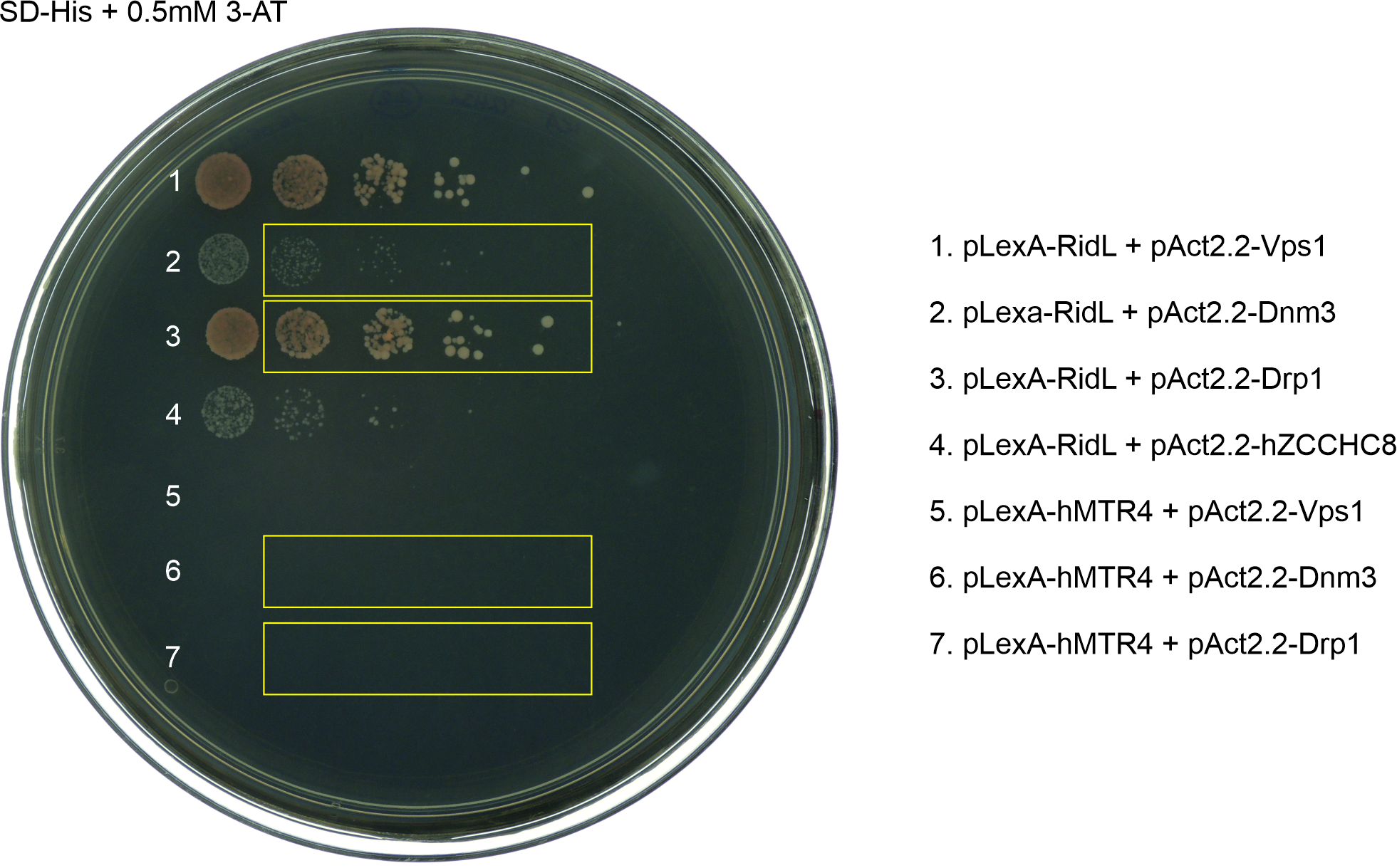

Supplement: Supplementary file 5 — Source data Fig. 3 [file 44319_2026_823_MOESM5_ESM.zip › Fig. 3/3A/SD-His + 0.5mM 3-AT (Drp1, Dnm3).tif]

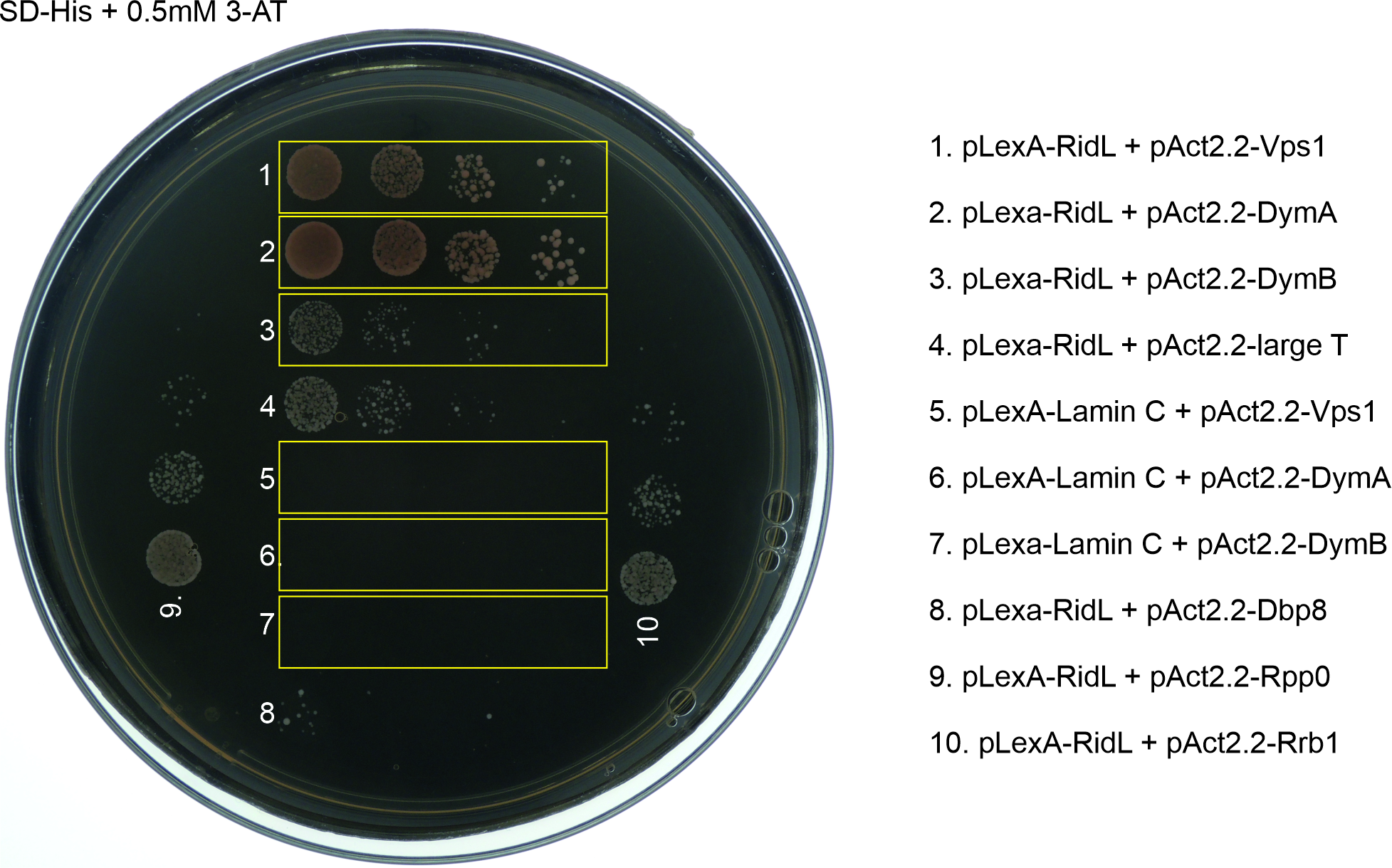

Supplement: Supplementary file 5 — Source data Fig. 3 [file 44319_2026_823_MOESM5_ESM.zip › Fig. 3/3A/SD-His + 0.5mM 3-AT (Vps1, DymA, DymB).tif]

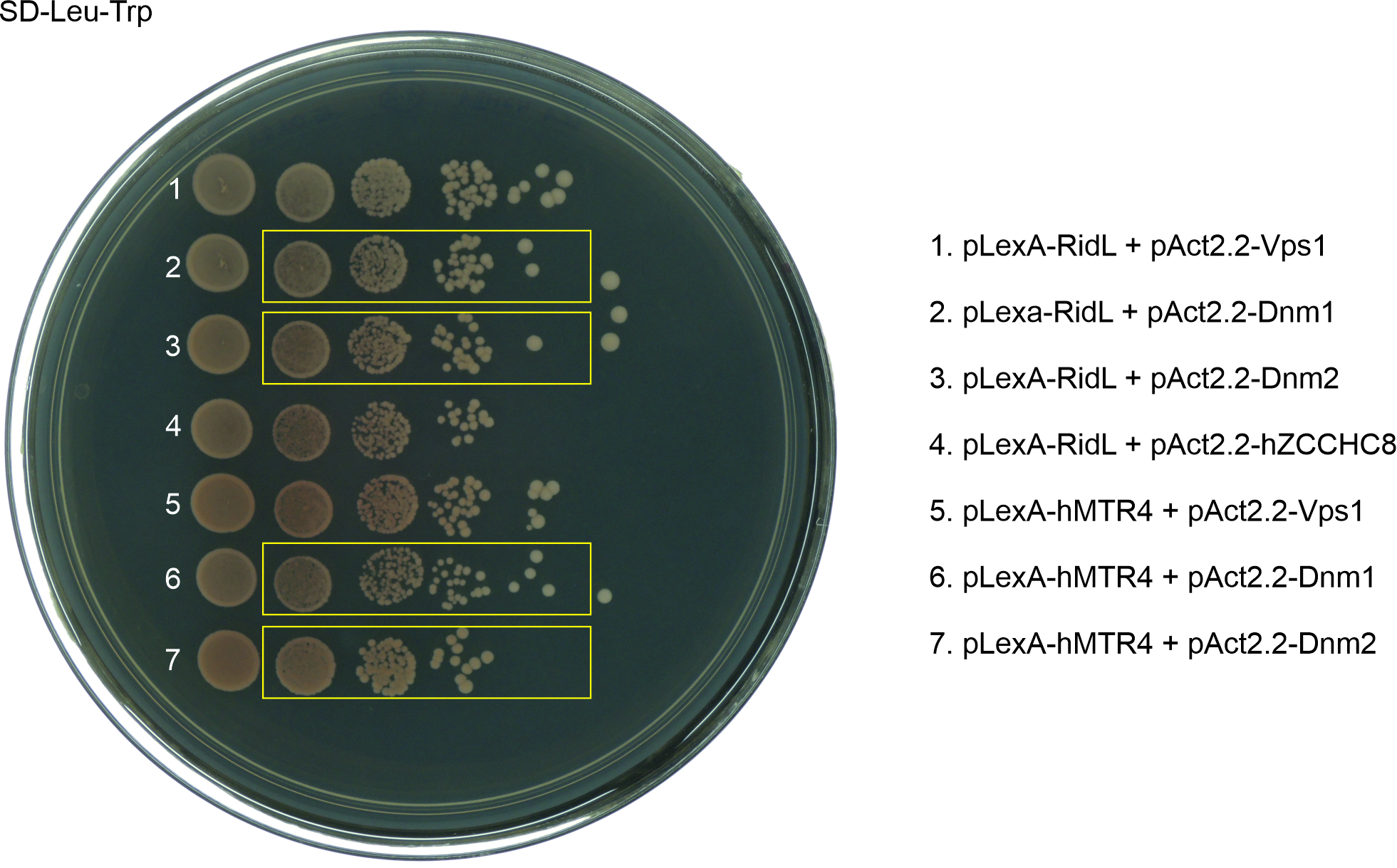

Supplement: Supplementary file 5 — Source data Fig. 3 [file 44319_2026_823_MOESM5_ESM.zip › Fig. 3/3A/SD-Leu-Trp (Dnm1, Dnm2).tif]

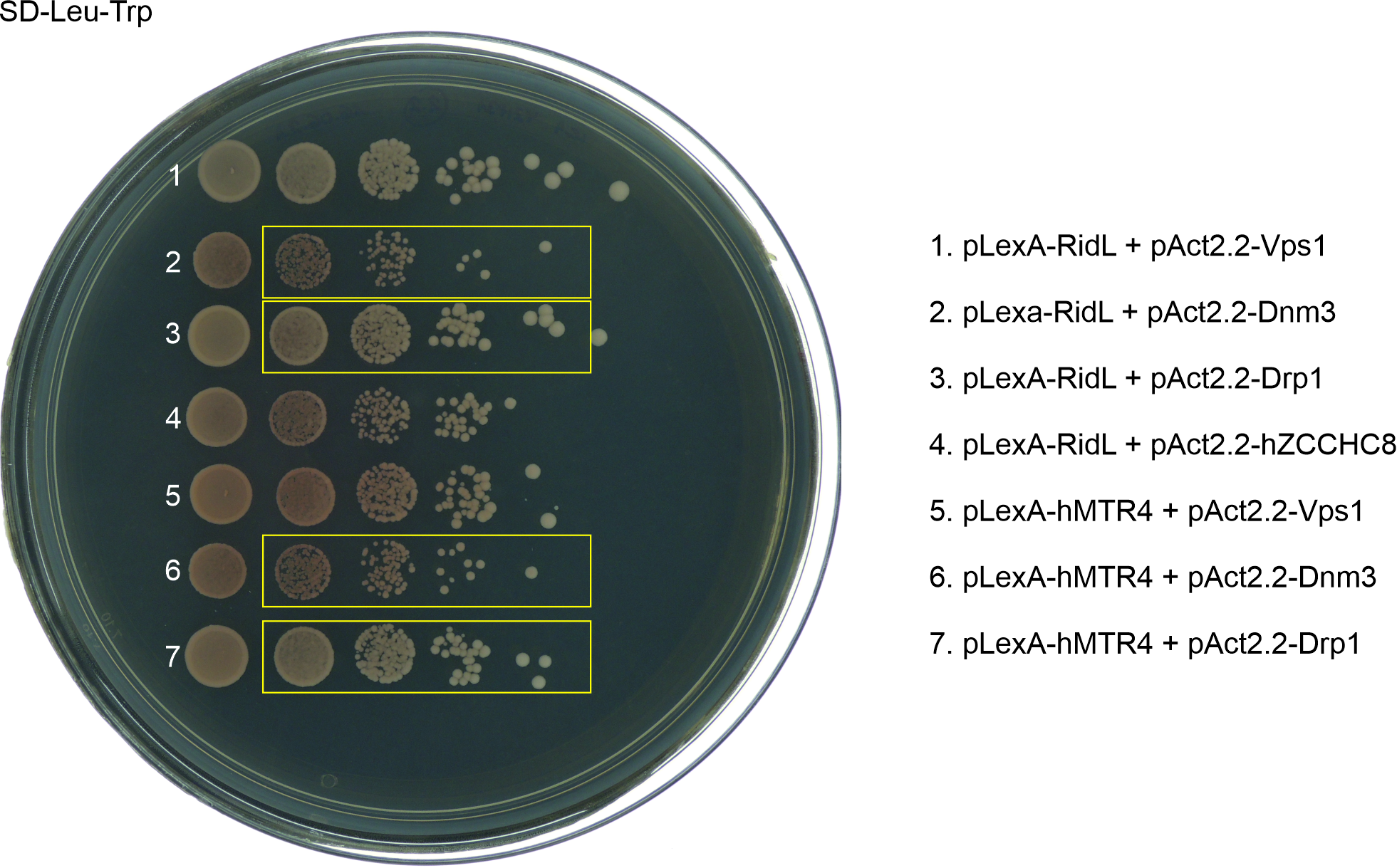

Supplement: Supplementary file 5 — Source data Fig. 3 [file 44319_2026_823_MOESM5_ESM.zip › Fig. 3/3A/SD-Leu-Trp (Drp1, Dnm3).tif]

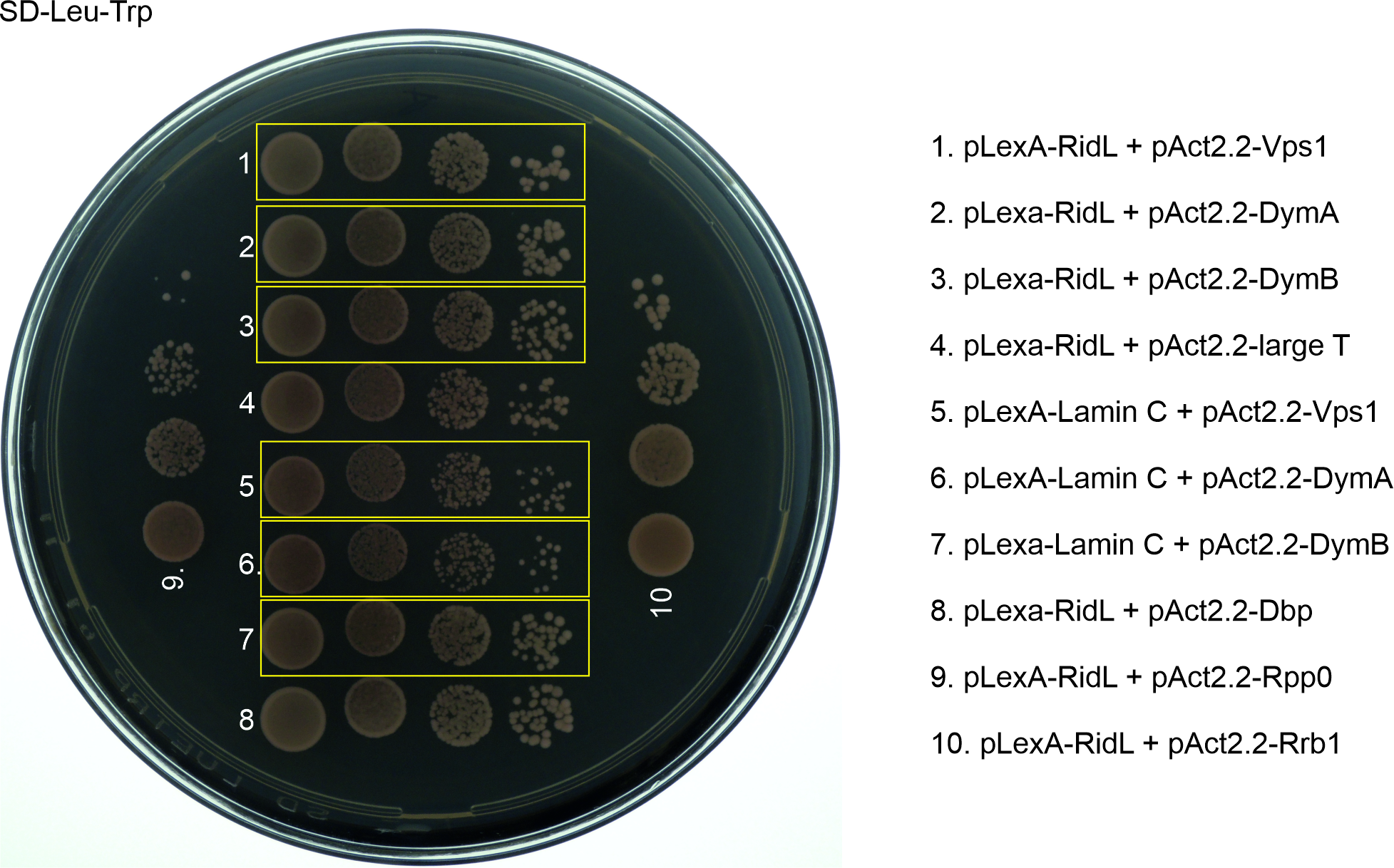

Supplement: Supplementary file 5 — Source data Fig. 3 [file 44319_2026_823_MOESM5_ESM.zip › Fig. 3/3A/SD-Leu-Trp (Vps1, DymA, DymB).tif]

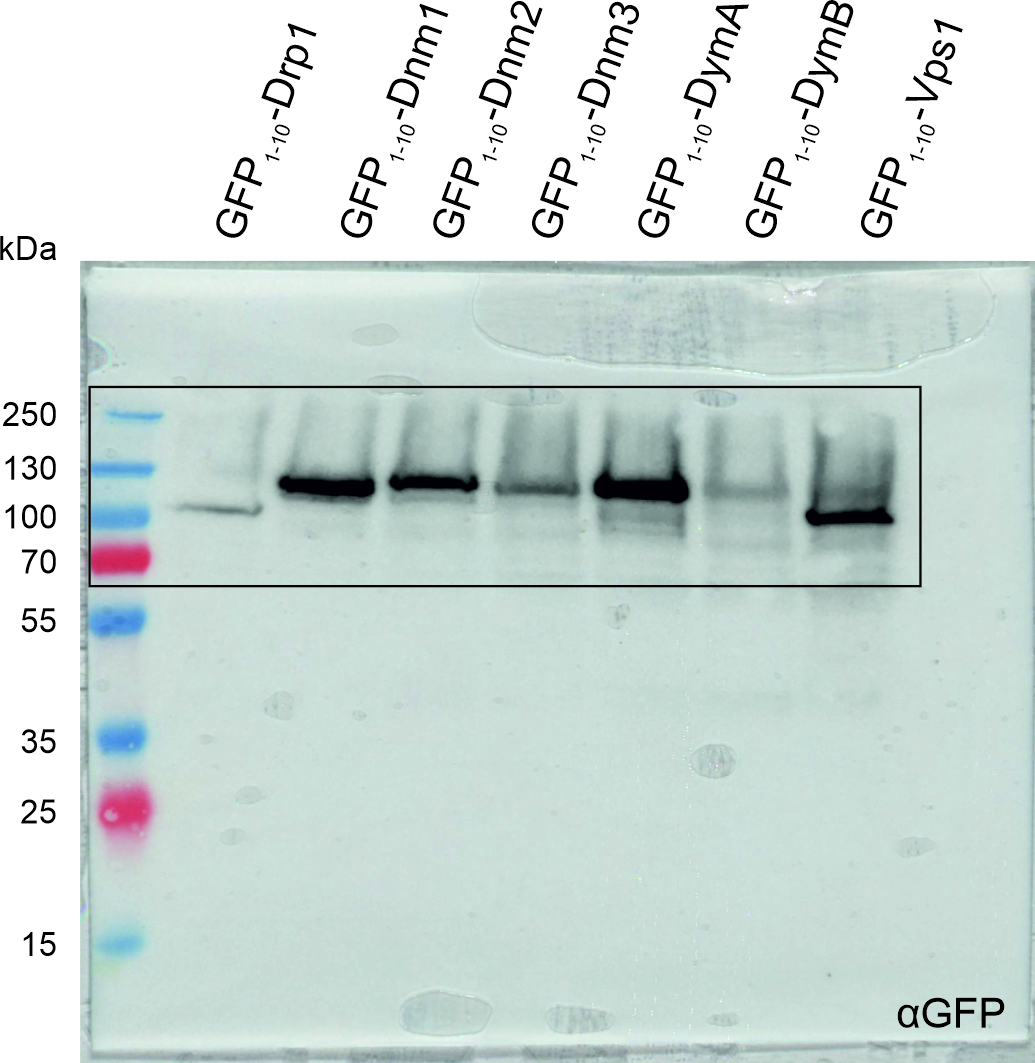

Supplement: Supplementary file 5 — Source data Fig. 3 [file 44319_2026_823_MOESM5_ESM.zip › Fig. 3/3D/anti-GFP western blot.tif]

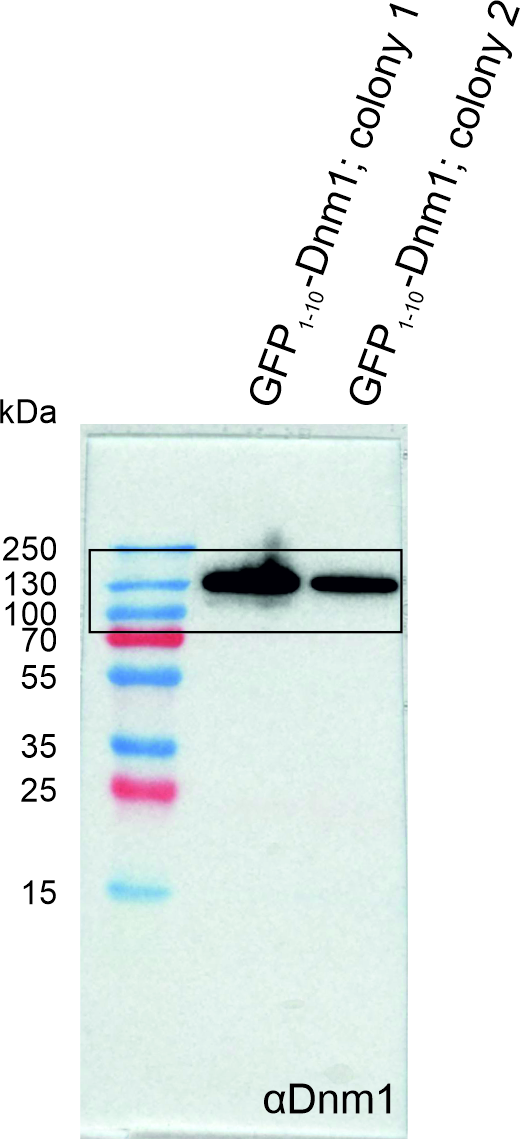

Supplement: Supplementary file 5 — Source data Fig. 3 [file 44319_2026_823_MOESM5_ESM.zip › Fig. 3/3E/anti-Dnm1 western blot.tif]

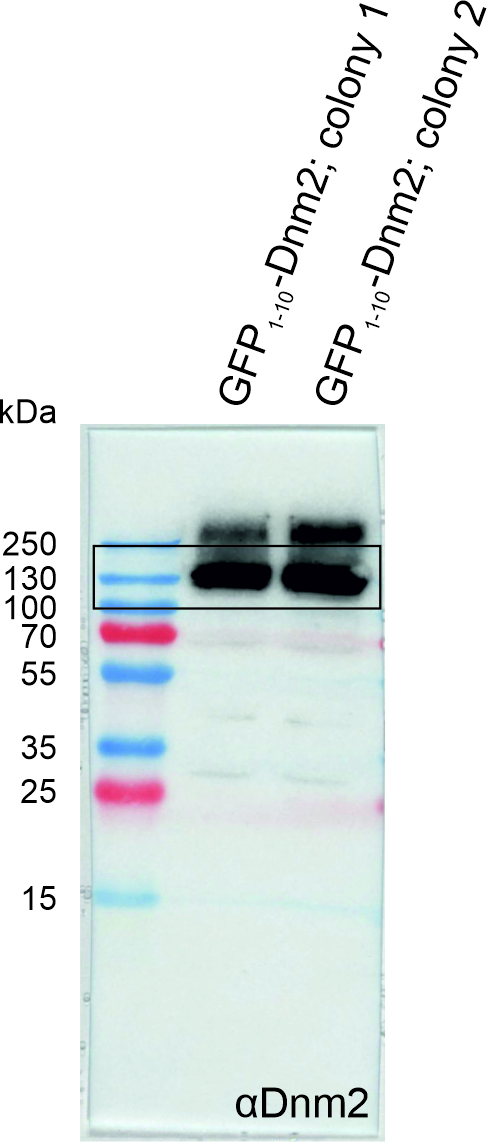

Supplement: Supplementary file 5 — Source data Fig. 3 [file 44319_2026_823_MOESM5_ESM.zip › Fig. 3/3E/anti-Dnm2 western blot.tif]

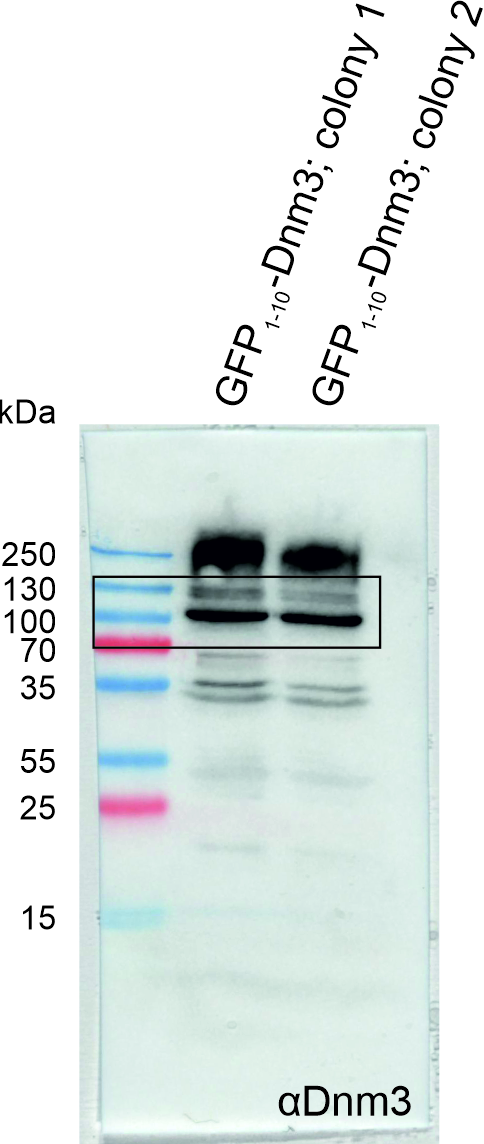

Supplement: Supplementary file 5 — Source data Fig. 3 [file 44319_2026_823_MOESM5_ESM.zip › Fig. 3/3E/anti-Dnm3 western blot.tif]

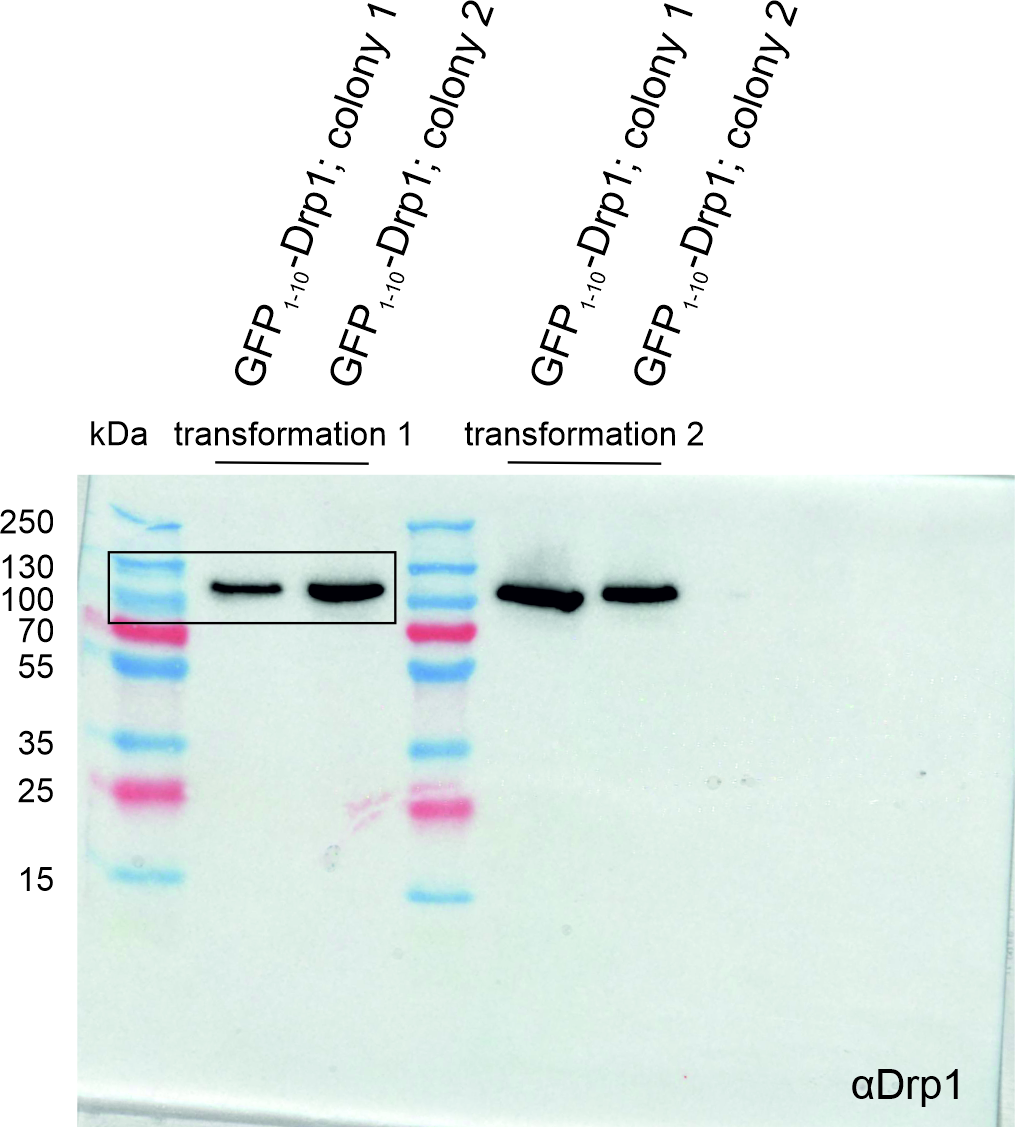

Supplement: Supplementary file 5 — Source data Fig. 3 [file 44319_2026_823_MOESM5_ESM.zip › Fig. 3/3E/anti-Drp1 western blot.tif]

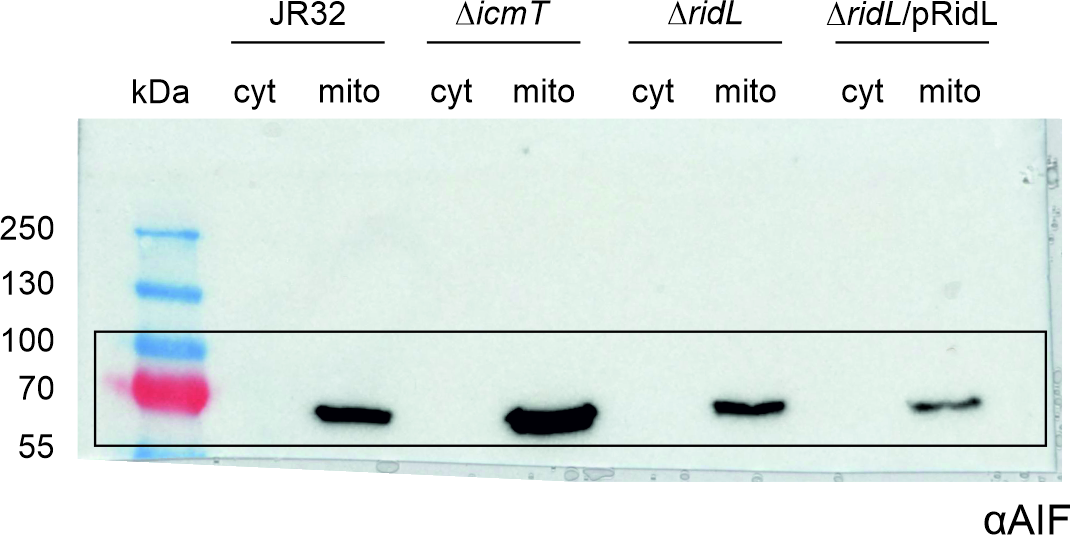

Supplement: Supplementary file 6 — Source data Fig. 4 [file 44319_2026_823_MOESM6_ESM.zip › Fig. 4/4B/antiAIF western blot.tif]

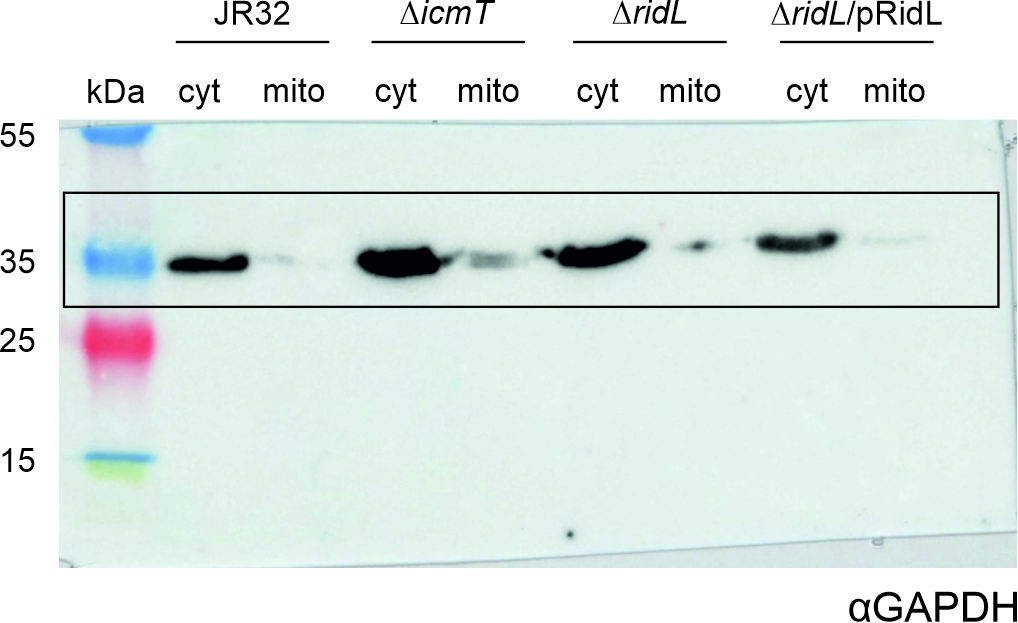

Supplement: Supplementary file 6 — Source data Fig. 4 [file 44319_2026_823_MOESM6_ESM.zip › Fig. 4/4B/antiGAPDH western blot.tif]

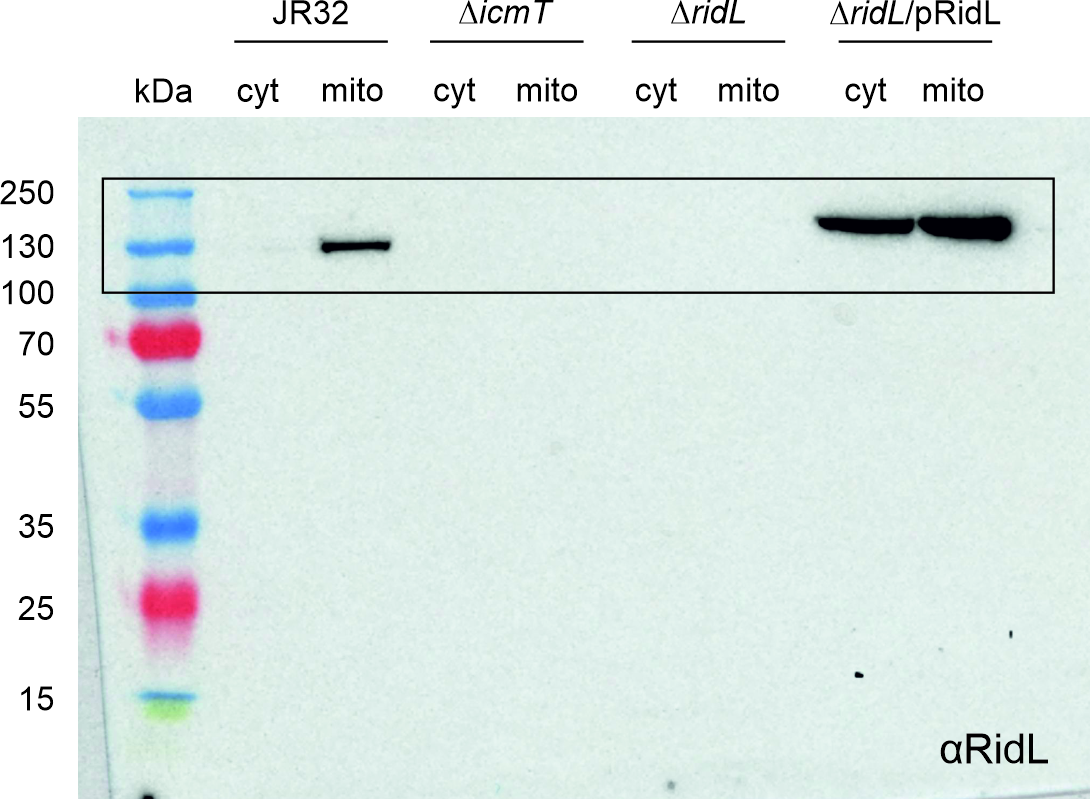

Supplement: Supplementary file 6 — Source data Fig. 4 [file 44319_2026_823_MOESM6_ESM.zip › Fig. 4/4B/antiRidL western blot.tif]

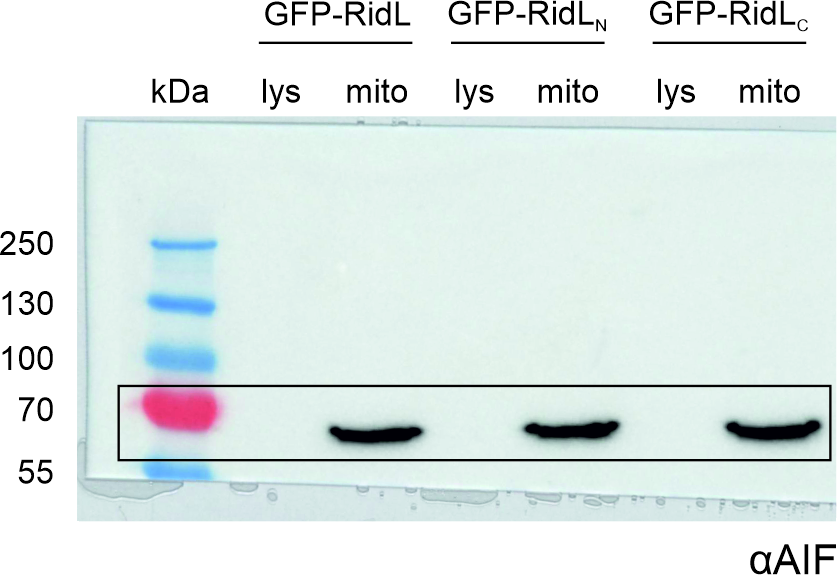

Supplement: Supplementary file 6 — Source data Fig. 4 [file 44319_2026_823_MOESM6_ESM.zip › Fig. 4/4C/antiAIF western blot.tif]

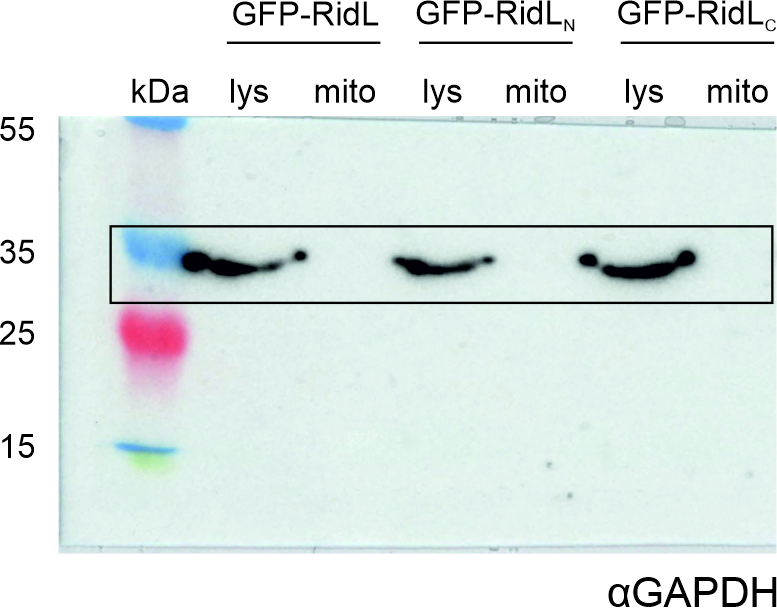

Supplement: Supplementary file 6 — Source data Fig. 4 [file 44319_2026_823_MOESM6_ESM.zip › Fig. 4/4C/antiGAPDH western blot.tif]

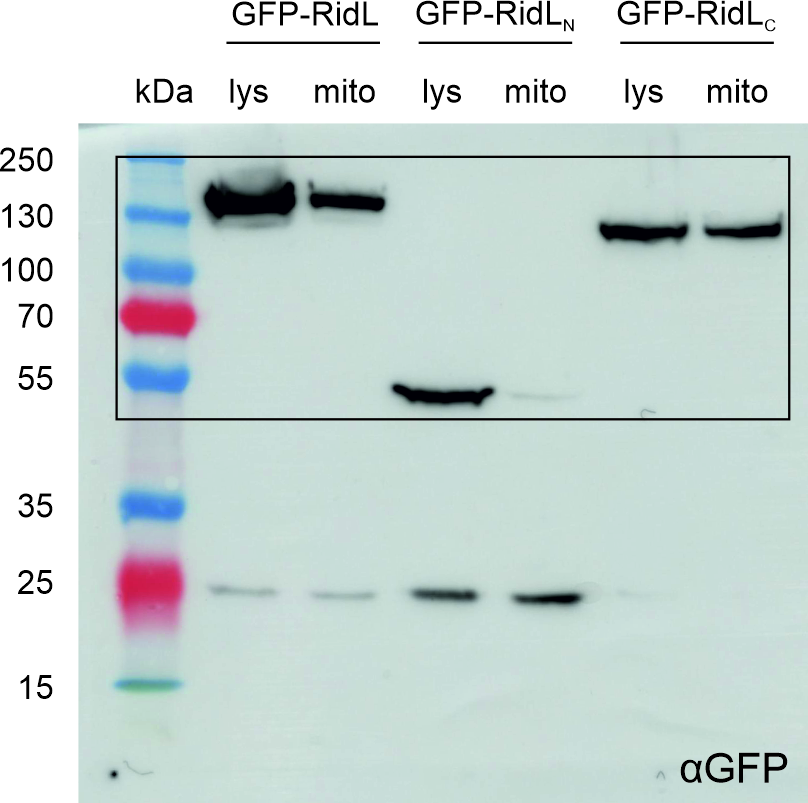

Supplement: Supplementary file 6 — Source data Fig. 4 [file 44319_2026_823_MOESM6_ESM.zip › Fig. 4/4C/antiGFP western blot.tif]

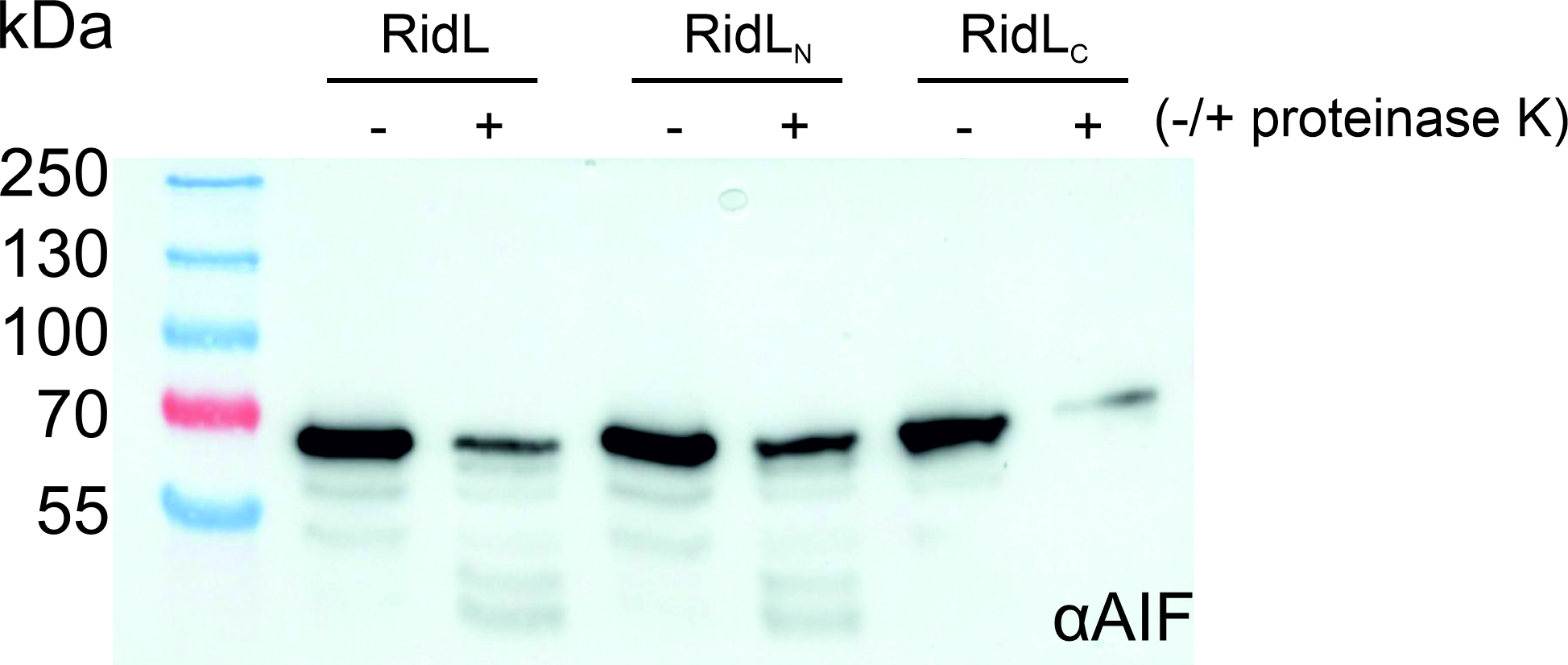

Supplement: Supplementary file 6 — Source data Fig. 4 [file 44319_2026_823_MOESM6_ESM.zip › Fig. 4/4D/western blot AIF.tif]

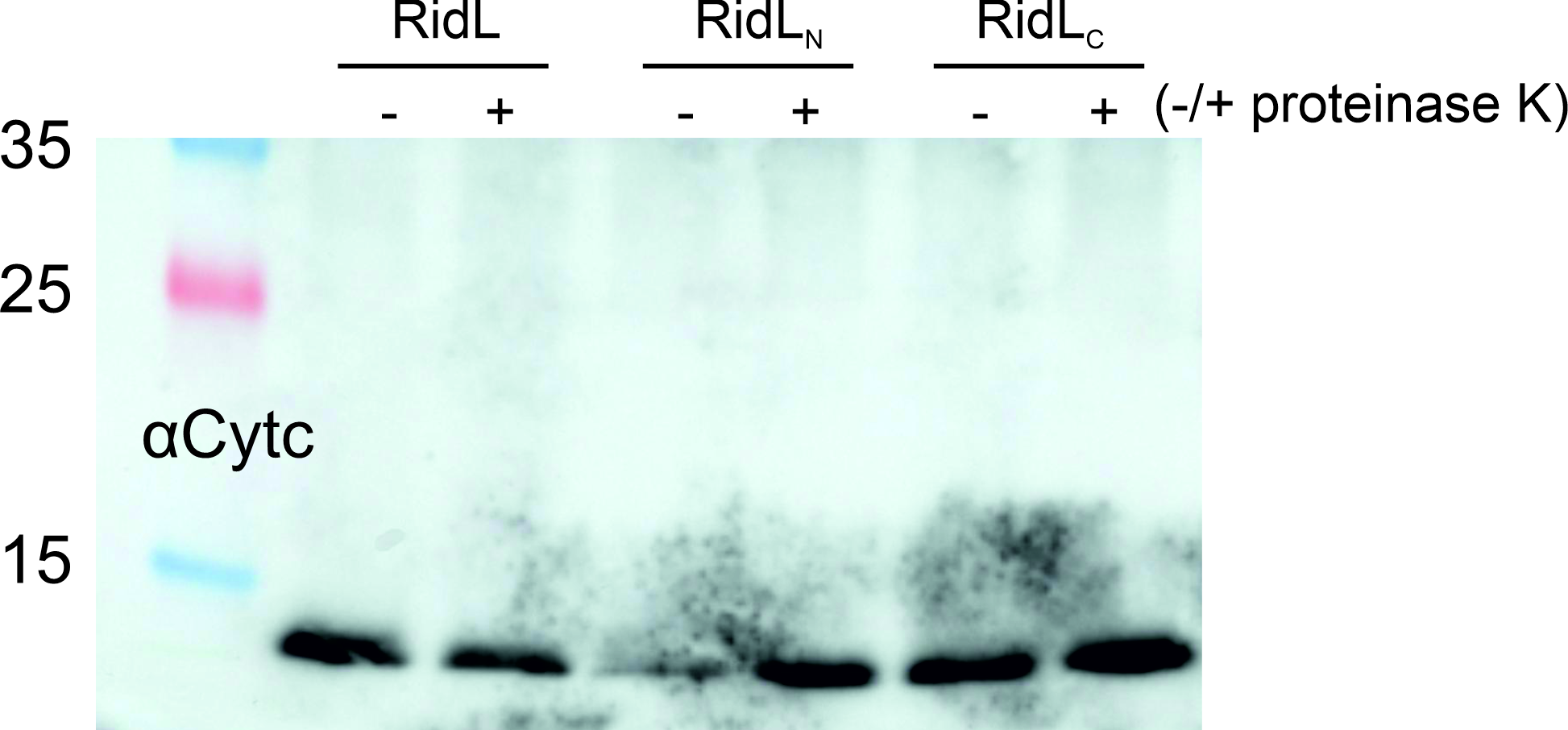

Supplement: Supplementary file 6 — Source data Fig. 4 [file 44319_2026_823_MOESM6_ESM.zip › Fig. 4/4D/western blot Cyt c.tif]

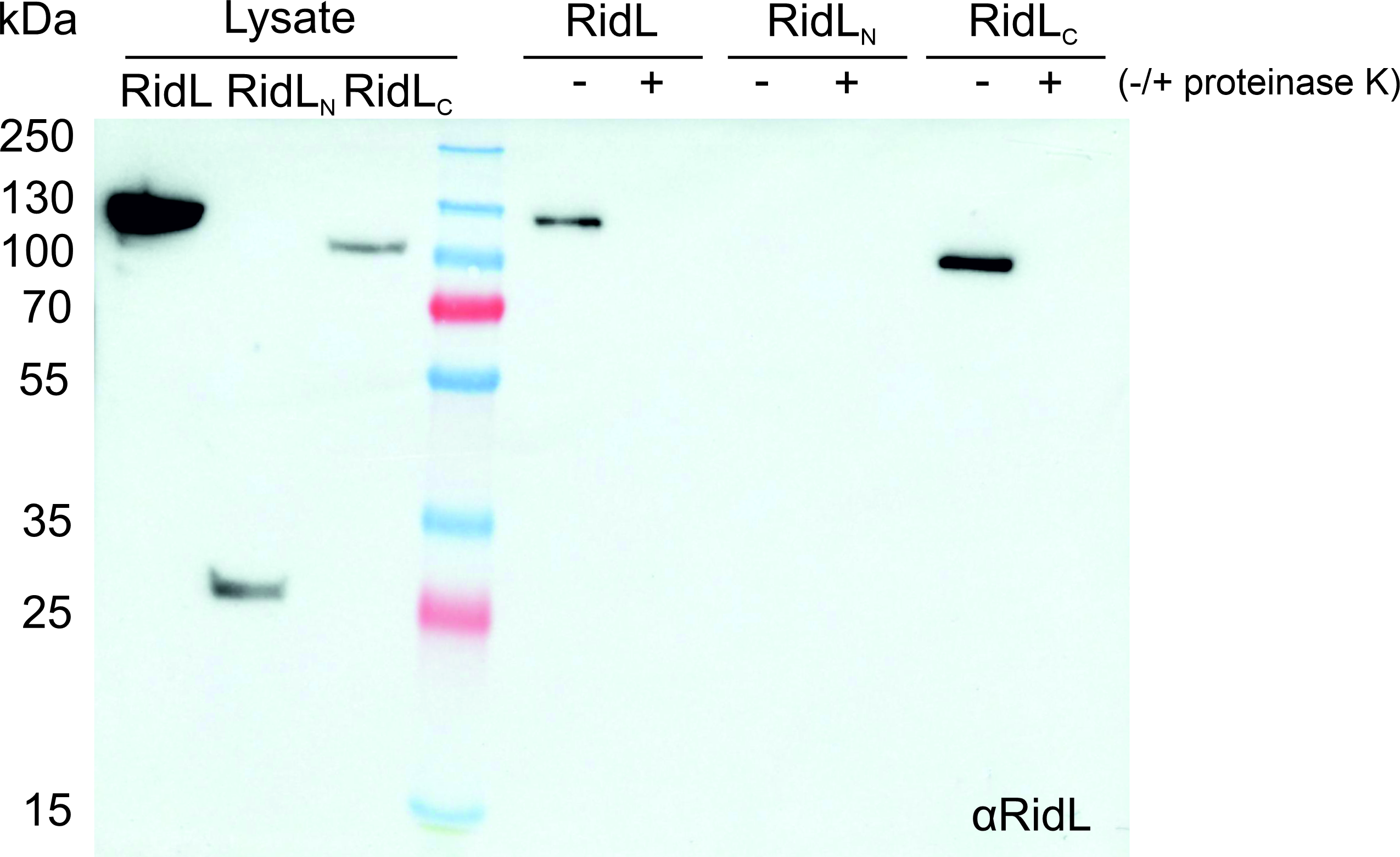

Supplement: Supplementary file 6 — Source data Fig. 4 [file 44319_2026_823_MOESM6_ESM.zip › Fig. 4/4D/western blot RidL.tif]

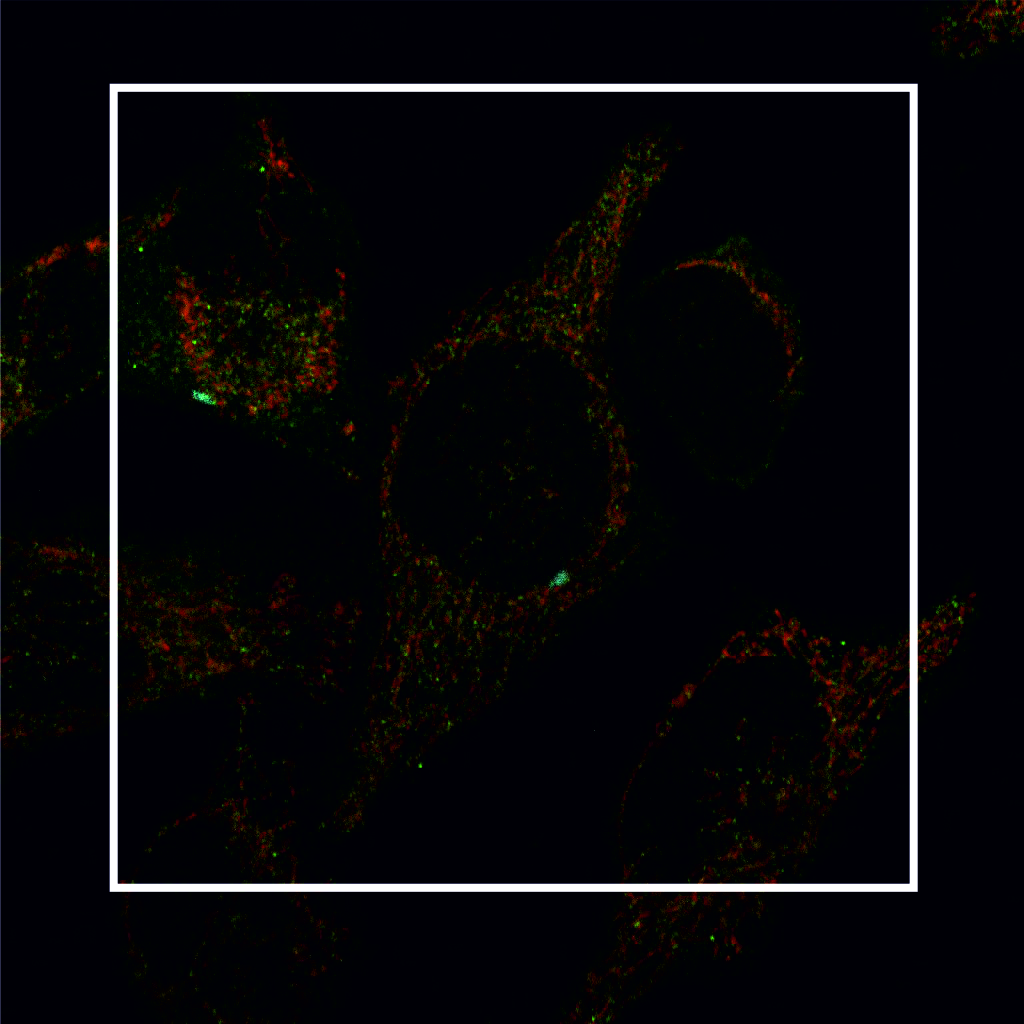

Supplement: Supplementary file 6 — Source data Fig. 4 [file 44319_2026_823_MOESM6_ESM.zip › Fig. 4/4E/dicmT, Drp1 (Alexa Fluor 488), MitoTracker DeepRed merged.tif]

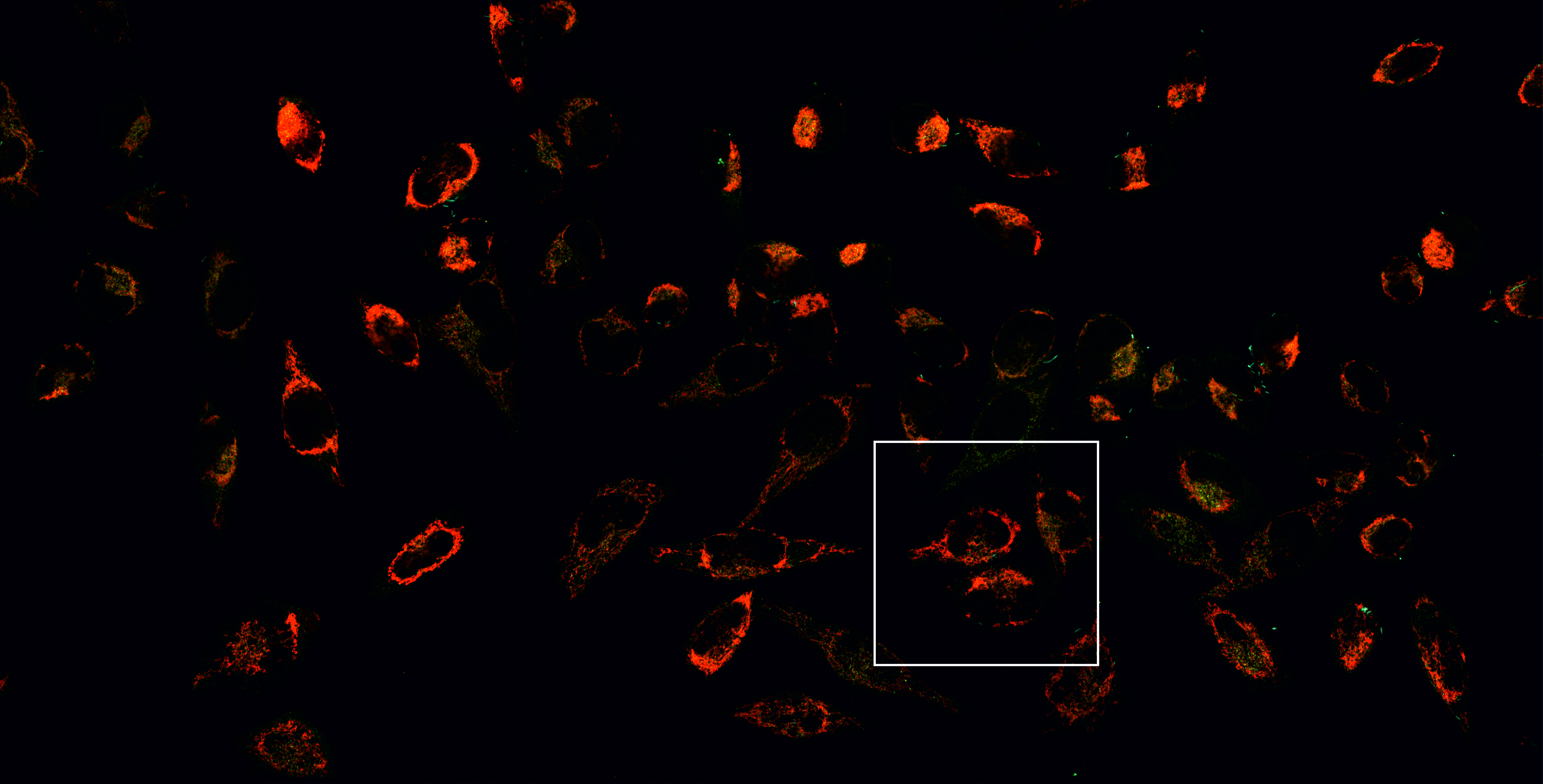

Supplement: Supplementary file 6 — Source data Fig. 4 [file 44319_2026_823_MOESM6_ESM.zip › Fig. 4/4E/dridL+pRidL, Drp1 (Alexa Fluor 488), MitoTracker DeepRed merged.tif]

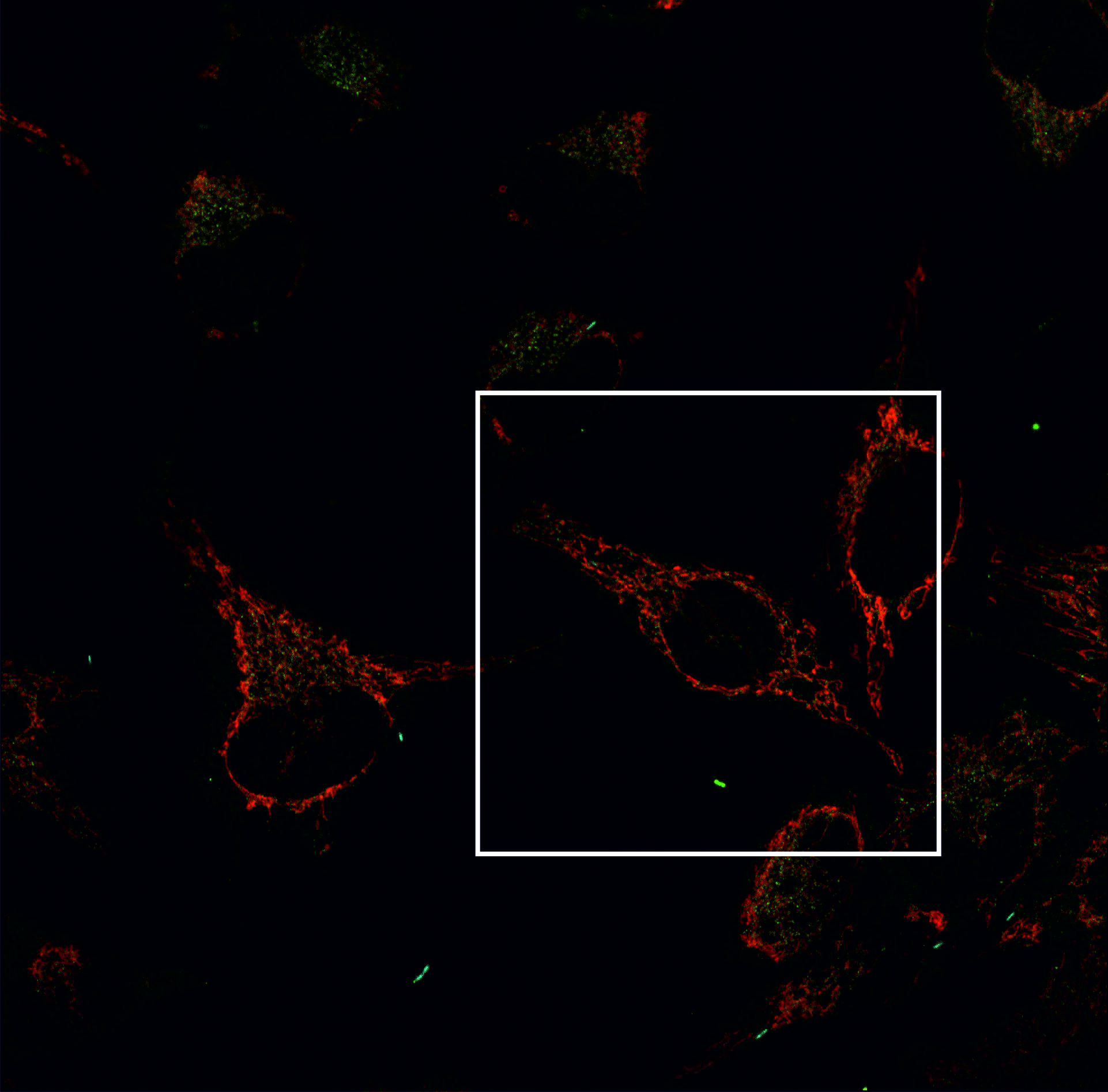

Supplement: Supplementary file 6 — Source data Fig. 4 [file 44319_2026_823_MOESM6_ESM.zip › Fig. 4/4E/dridL, Drp1 (Alexa Fluor 488), MitoTracker DeepRed merged.tif]

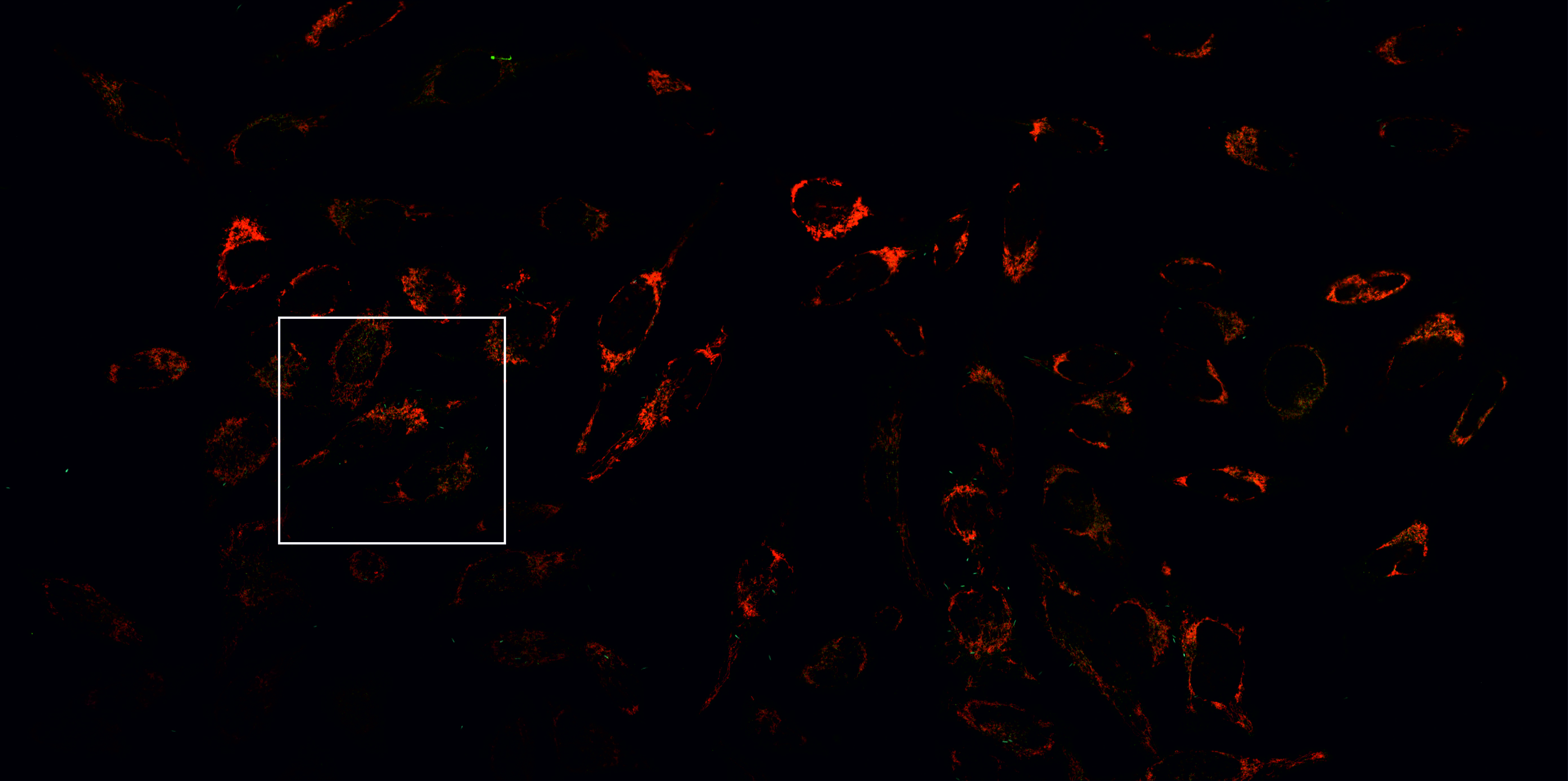

Supplement: Supplementary file 6 — Source data Fig. 4 [file 44319_2026_823_MOESM6_ESM.zip › Fig. 4/4E/JR32, Drp1 (Alexa Fluor 488), MitoTracker DeepRed merged.tif]

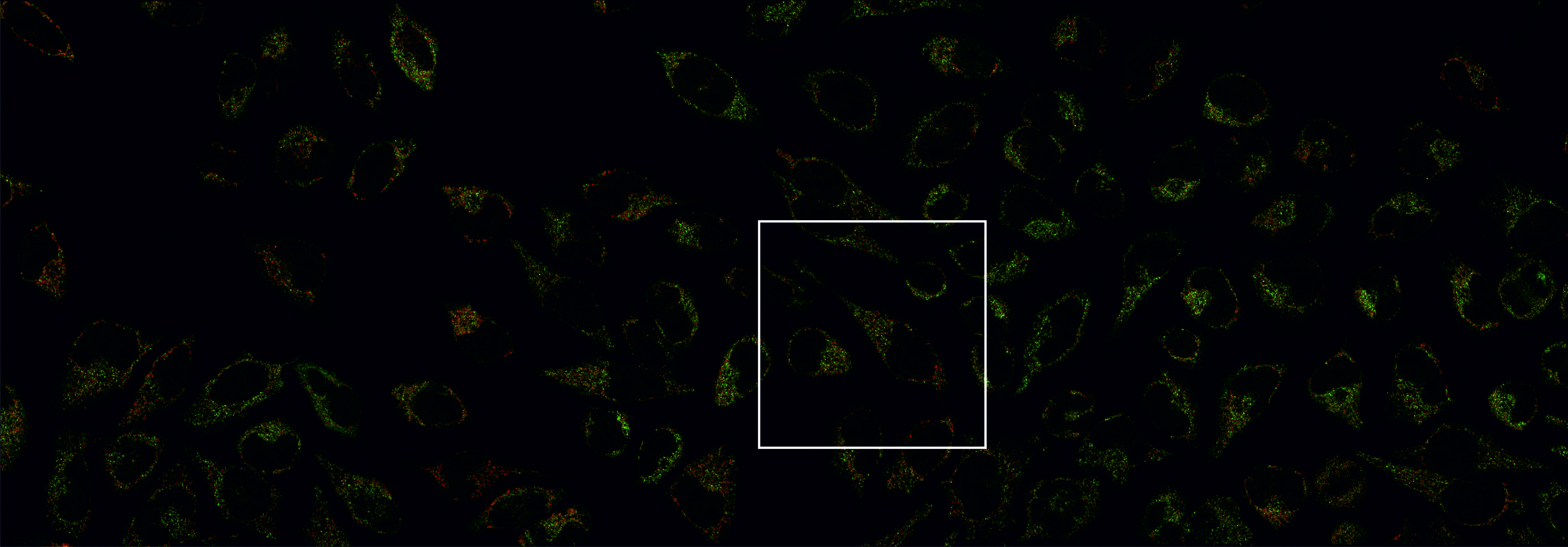

Supplement: Supplementary file 6 — Source data Fig. 4 [file 44319_2026_823_MOESM6_ESM.zip › Fig. 4/4E/uninfected, Drp1 (Alexa Fluor 488), MitoTracker DeepRed merged.tif]

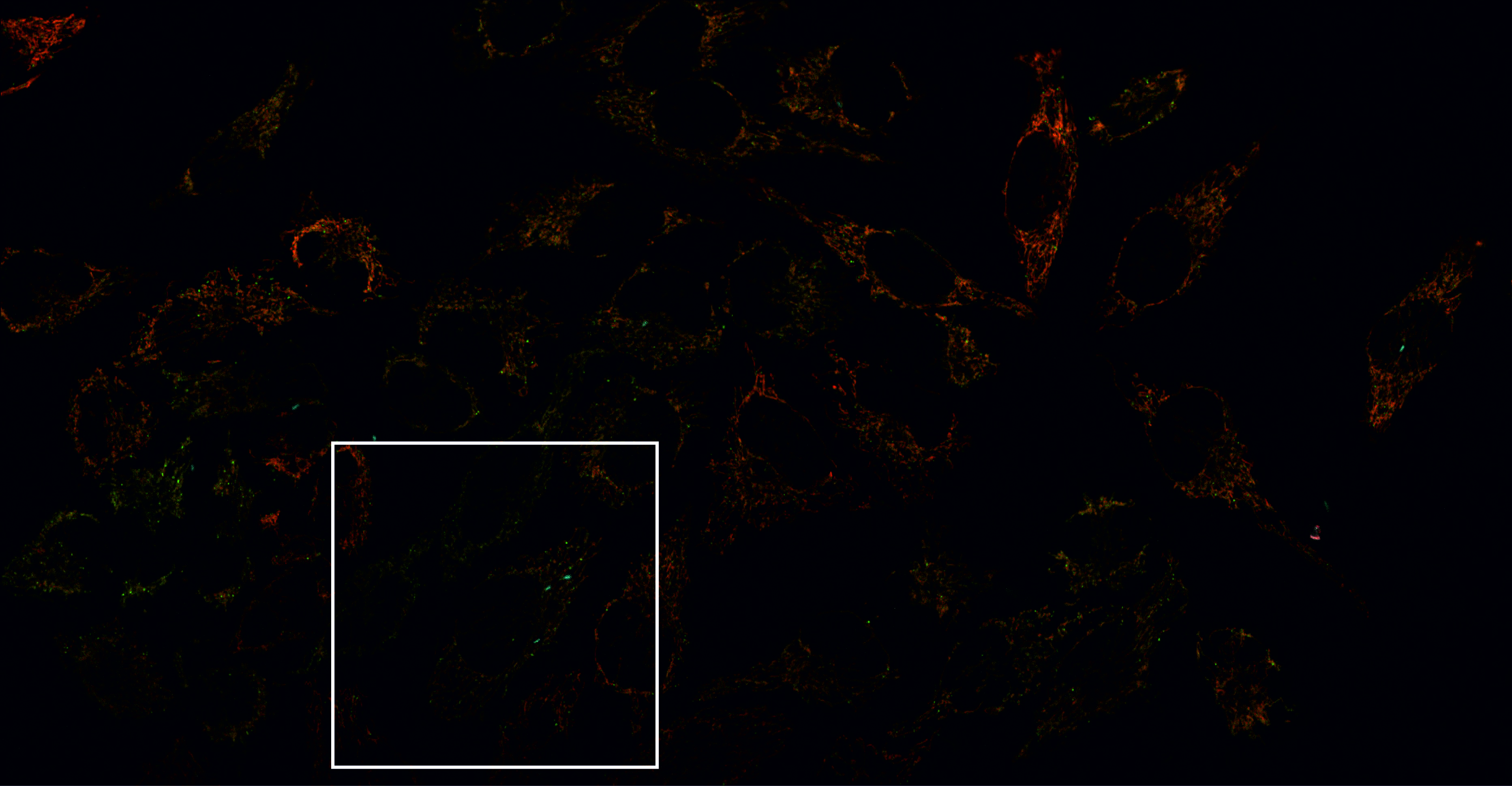

Supplement: Supplementary file 6 — Source data Fig. 4 [file 44319_2026_823_MOESM6_ESM.zip › Fig. 4/4F/dicmT, Tom20 (Alexa Fluor 488), MitoTracker DeepRed merged.tif]

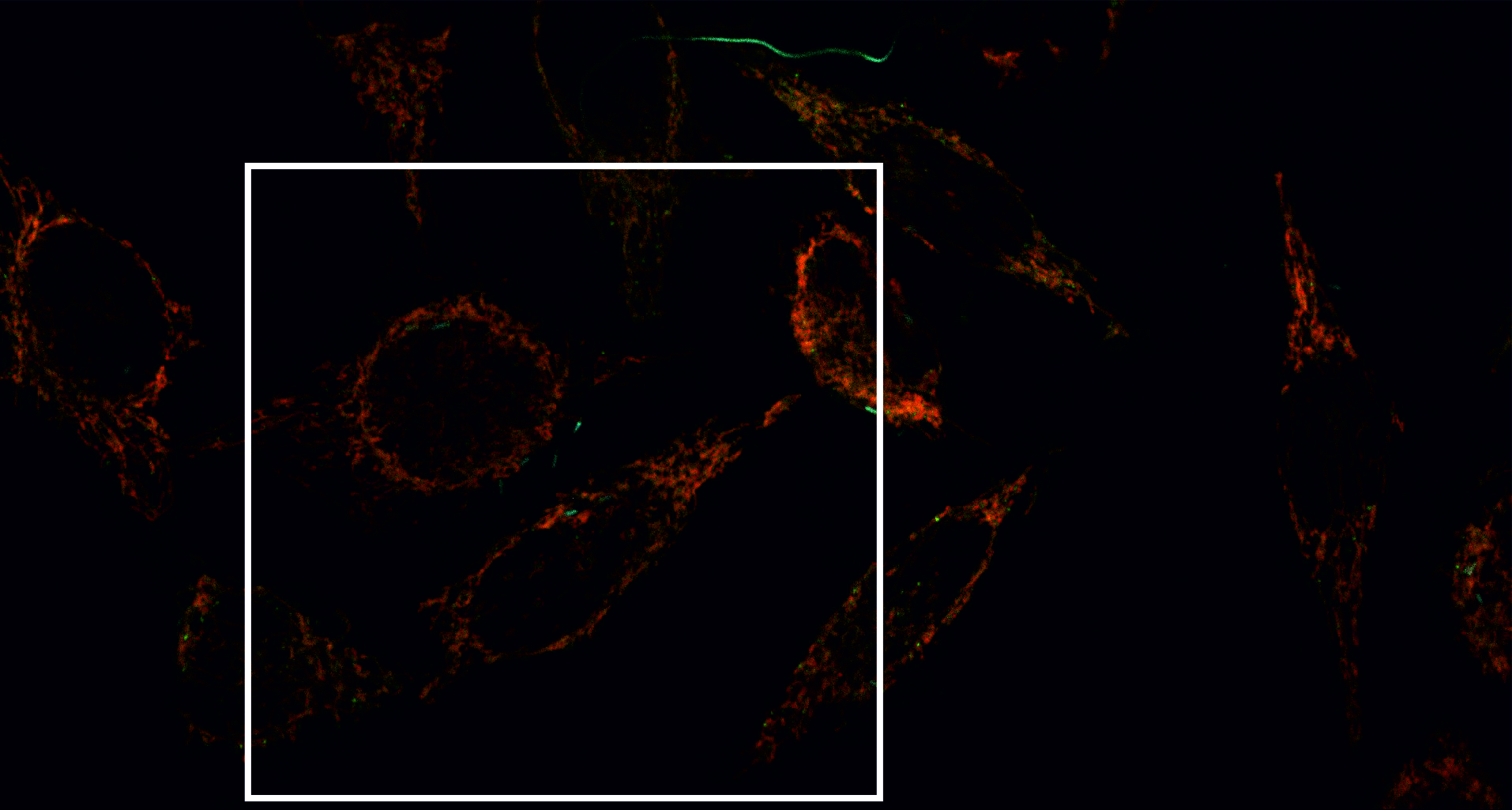

Supplement: Supplementary file 6 — Source data Fig. 4 [file 44319_2026_823_MOESM6_ESM.zip › Fig. 4/4F/dRidL+pRidL, Tom20 (Alexa Fluor 488), MitoTracker DeepRed merged.tif]

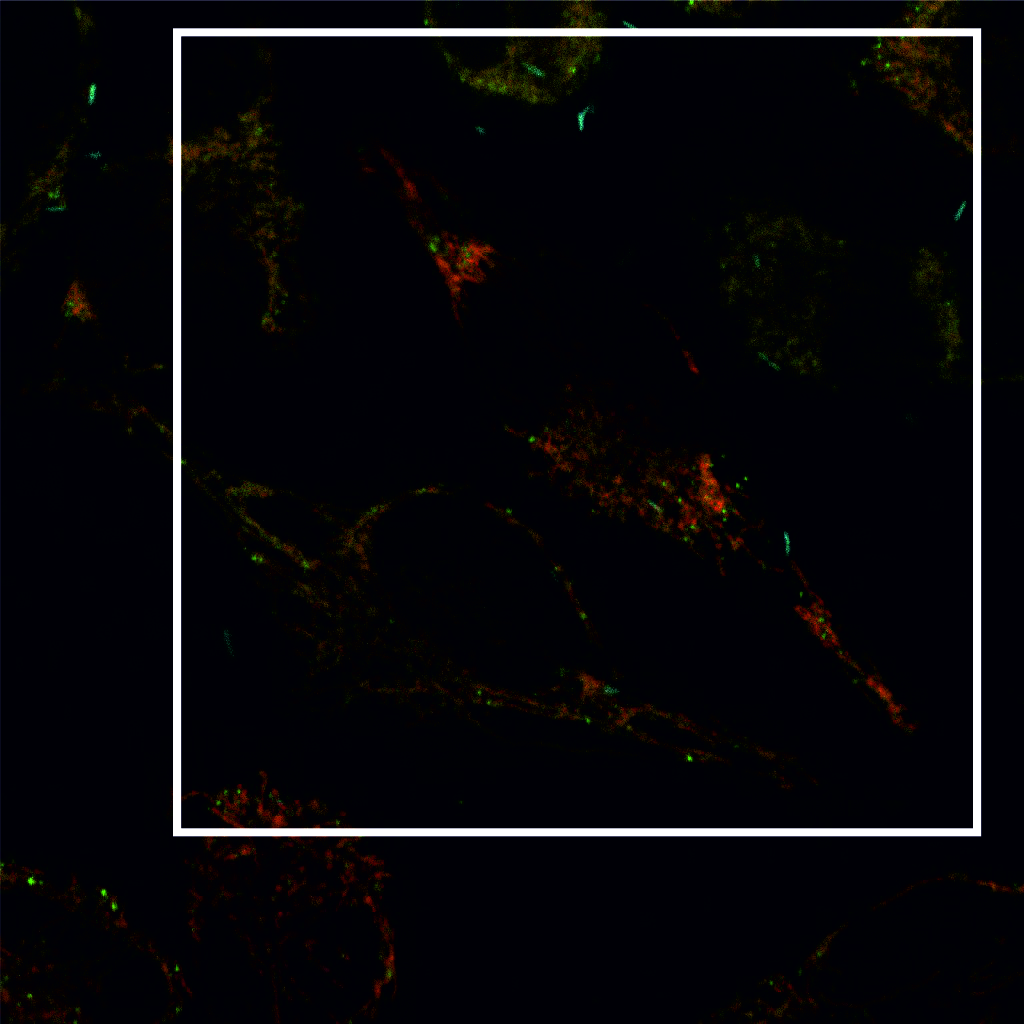

Supplement: Supplementary file 6 — Source data Fig. 4 [file 44319_2026_823_MOESM6_ESM.zip › Fig. 4/4F/dridL, Tom20 (Alexa Fluor 488), MitoTracker DeepRed merged.tif]

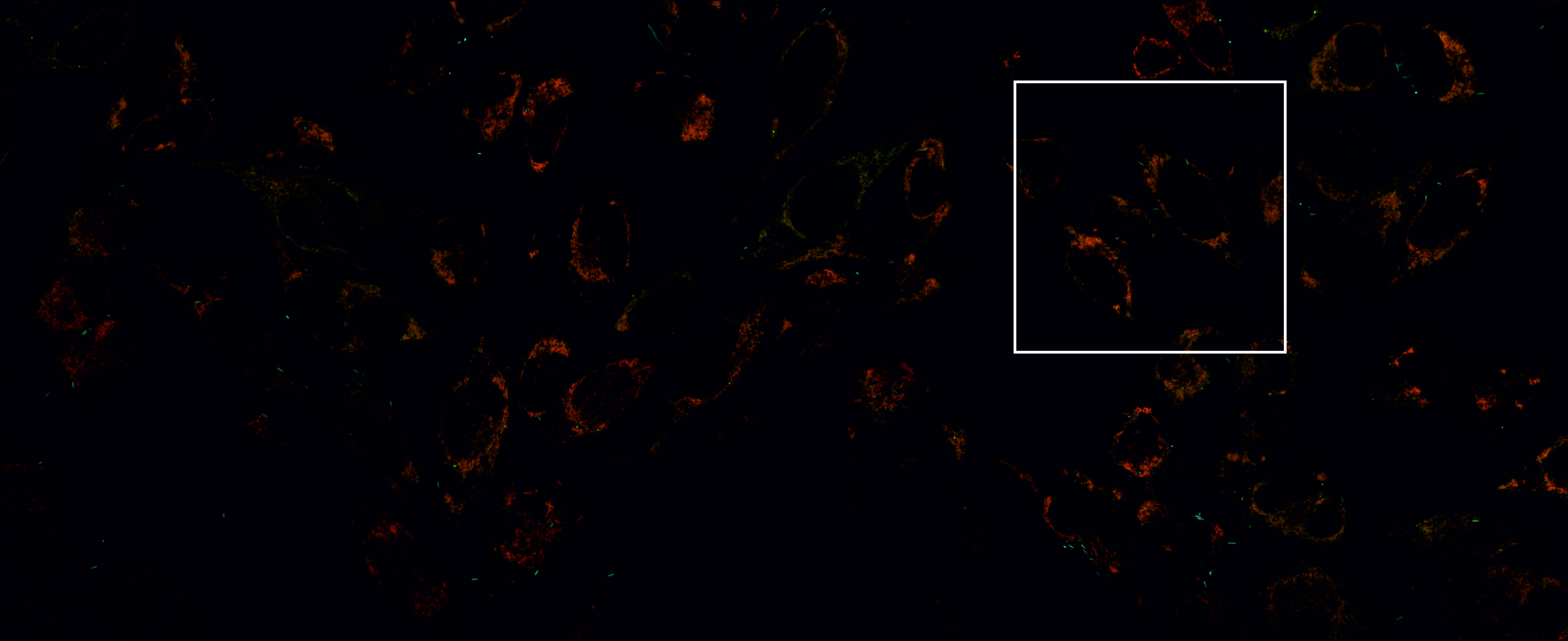

Supplement: Supplementary file 6 — Source data Fig. 4 [file 44319_2026_823_MOESM6_ESM.zip › Fig. 4/4F/JR32, Tom20 (Alexa Fluor 488), MitoTracker DeepRed merged.tif]

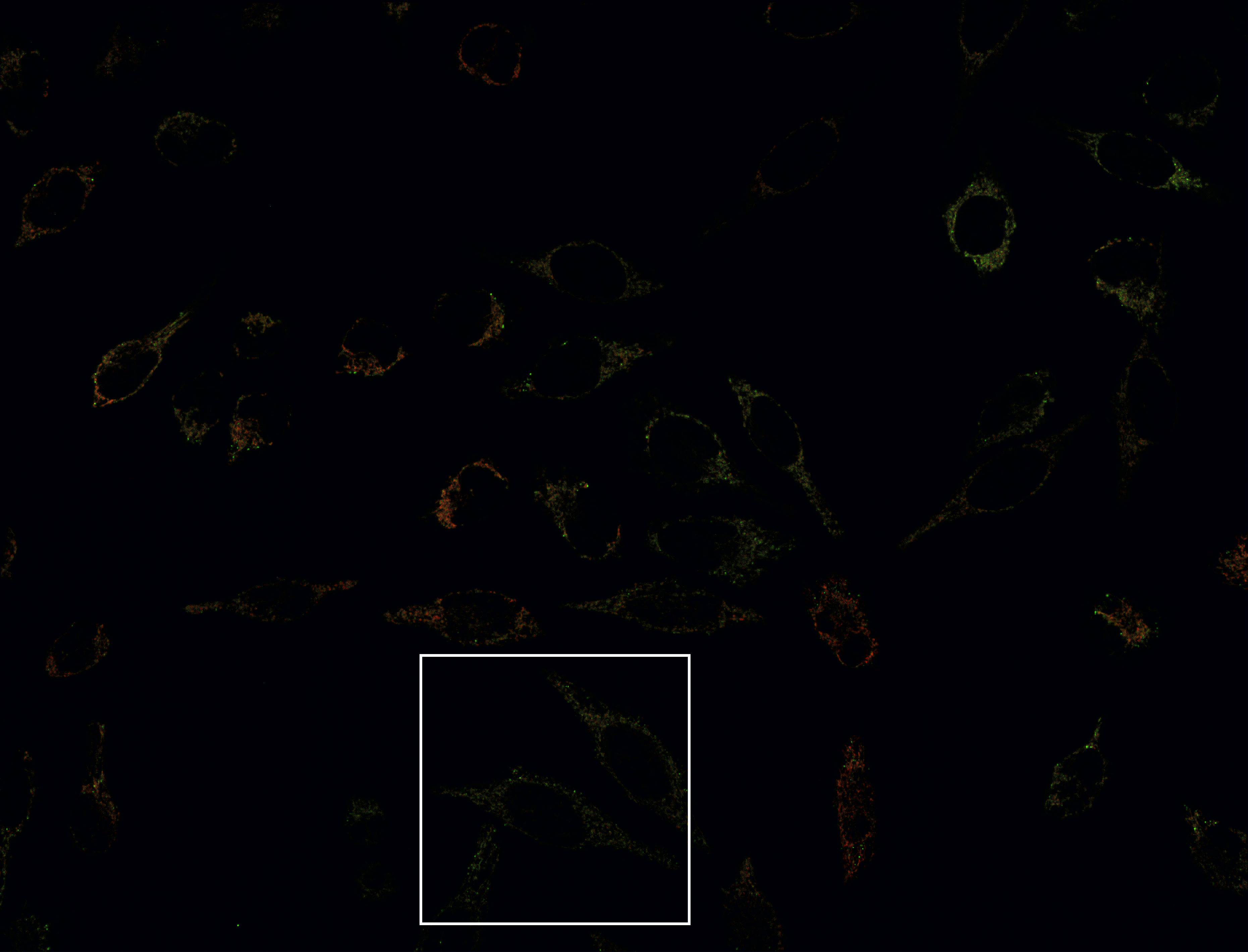

Supplement: Supplementary file 6 — Source data Fig. 4 [file 44319_2026_823_MOESM6_ESM.zip › Fig. 4/4F/uninfected, Tom20 (Alexa Fluor 488), MitoTracker DeepRed merged.tif]

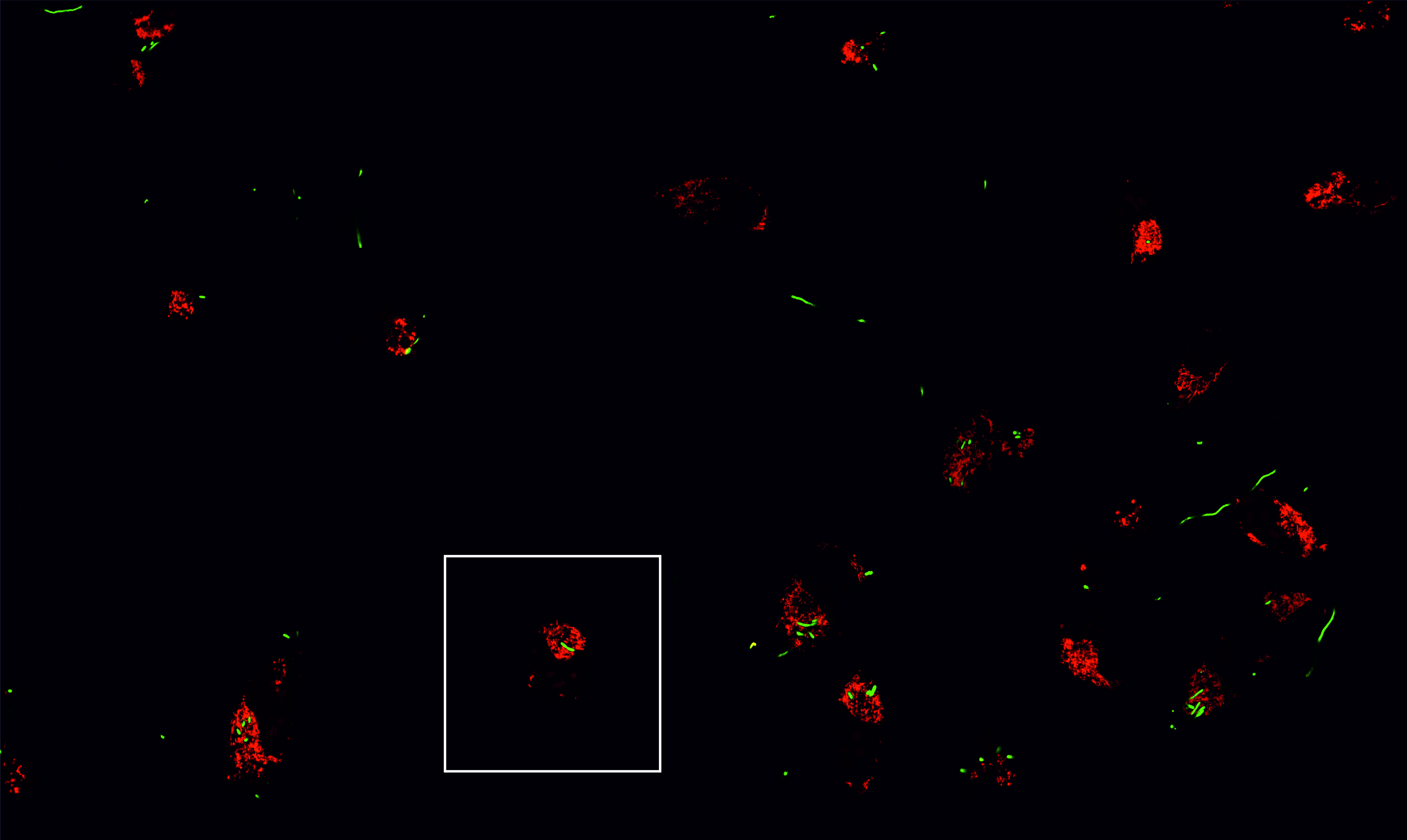

Supplement: Supplementary file 7 — Source data Fig. 5 [file 44319_2026_823_MOESM7_ESM.zip › Fig. 5/5A/dridL+pRidL, 6 h p.i..tif]

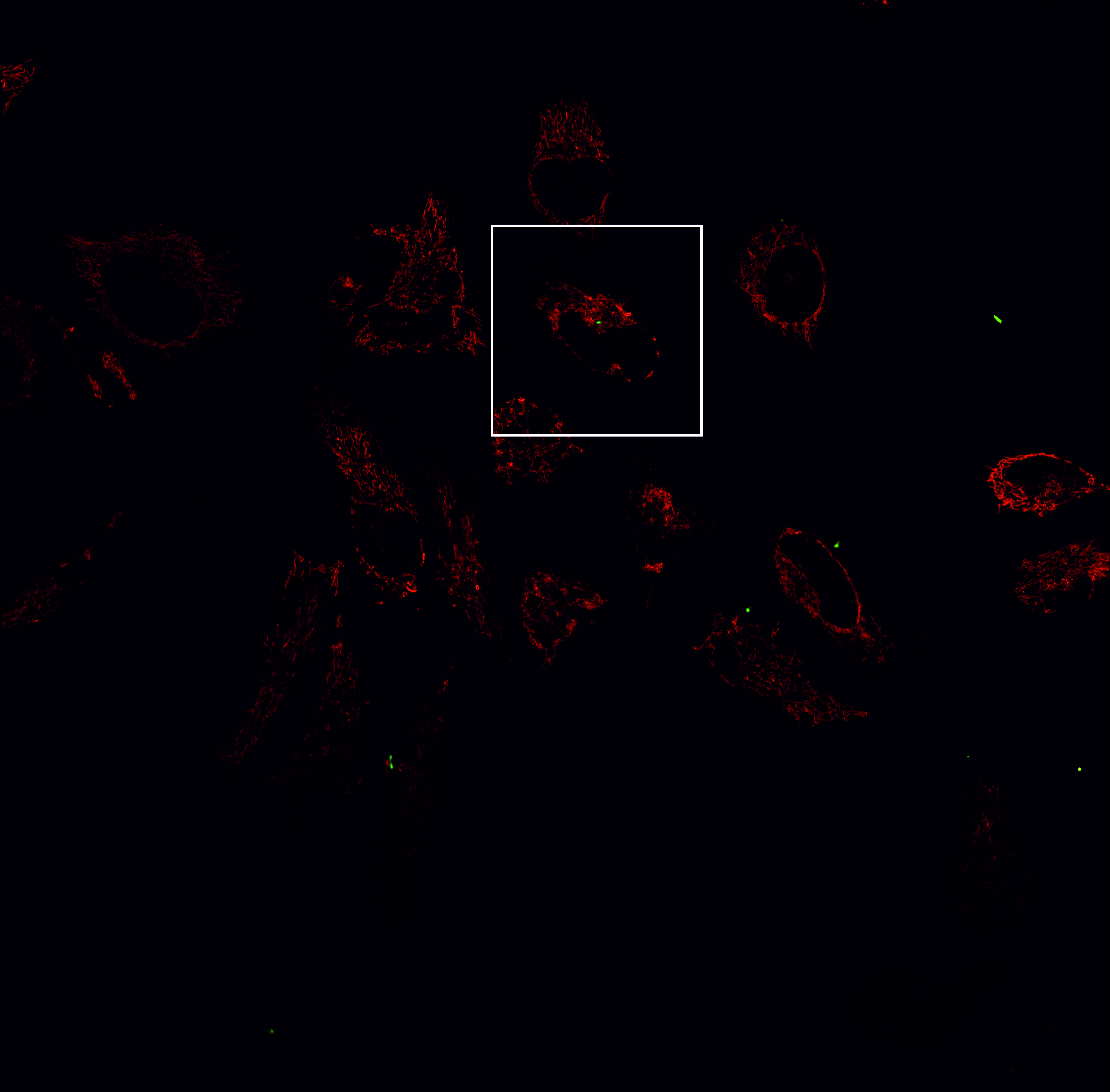

Supplement: Supplementary file 7 — Source data Fig. 5 [file 44319_2026_823_MOESM7_ESM.zip › Fig. 5/5A/dridl, 6 h p.i..tif]

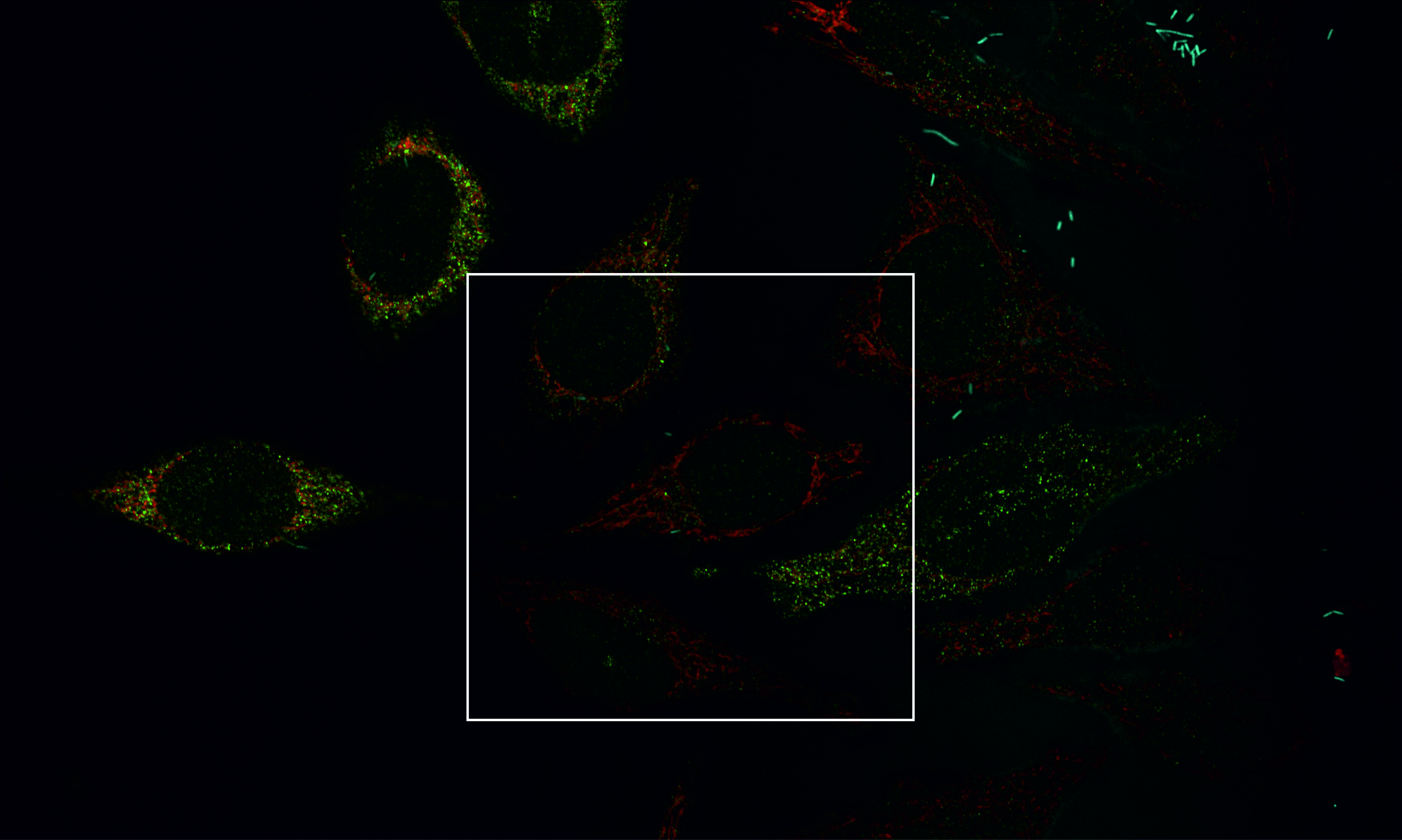

Supplement: Supplementary file 8 — Source data Fig. 6 [file 44319_2026_823_MOESM8_ESM.zip › Fig. 6/6A/dridL pDrp1.tif]

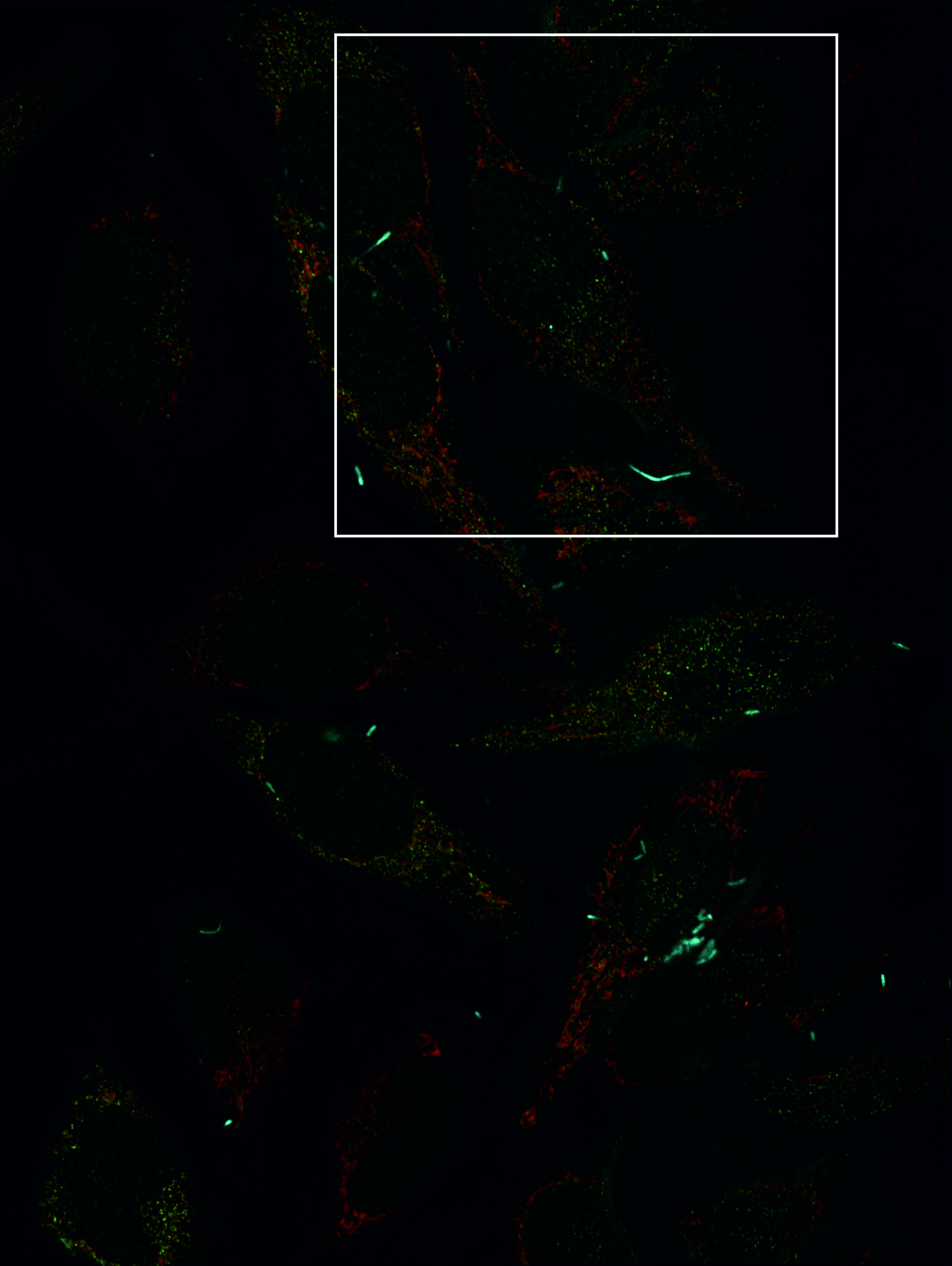

Supplement: Supplementary file 8 — Source data Fig. 6 [file 44319_2026_823_MOESM8_ESM.zip › Fig. 6/6A/dridL+pRidL pDrp1.tif]

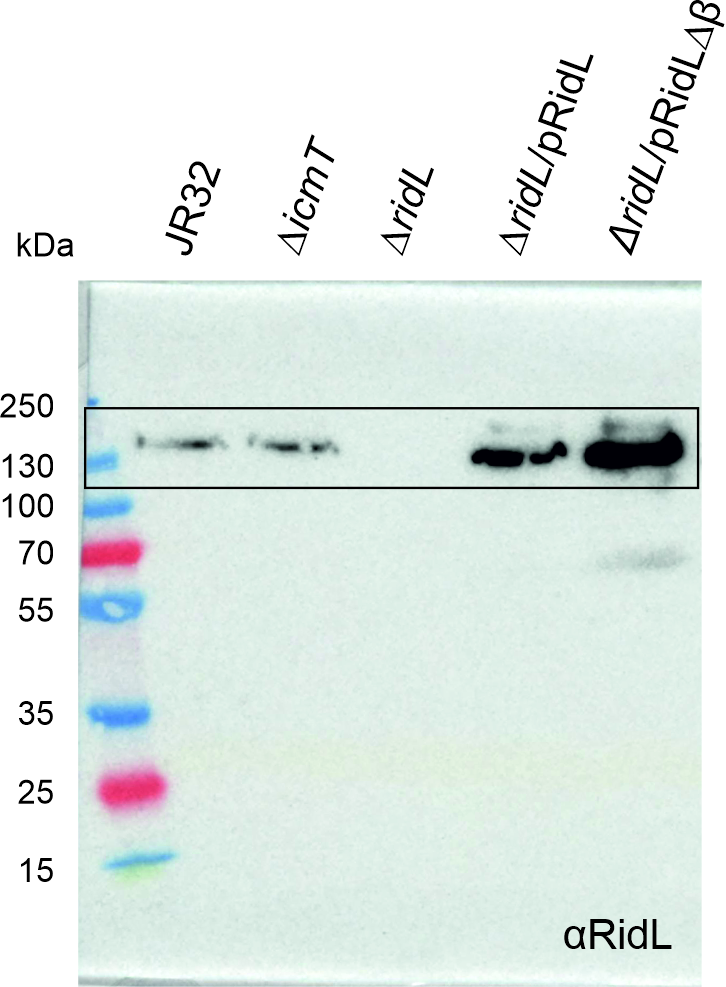

Supplement: Supplementary file 8 — Source data Fig. 6 [file 44319_2026_823_MOESM8_ESM.zip › Fig. 6/6D/anti-ridL western blot-1.tif]

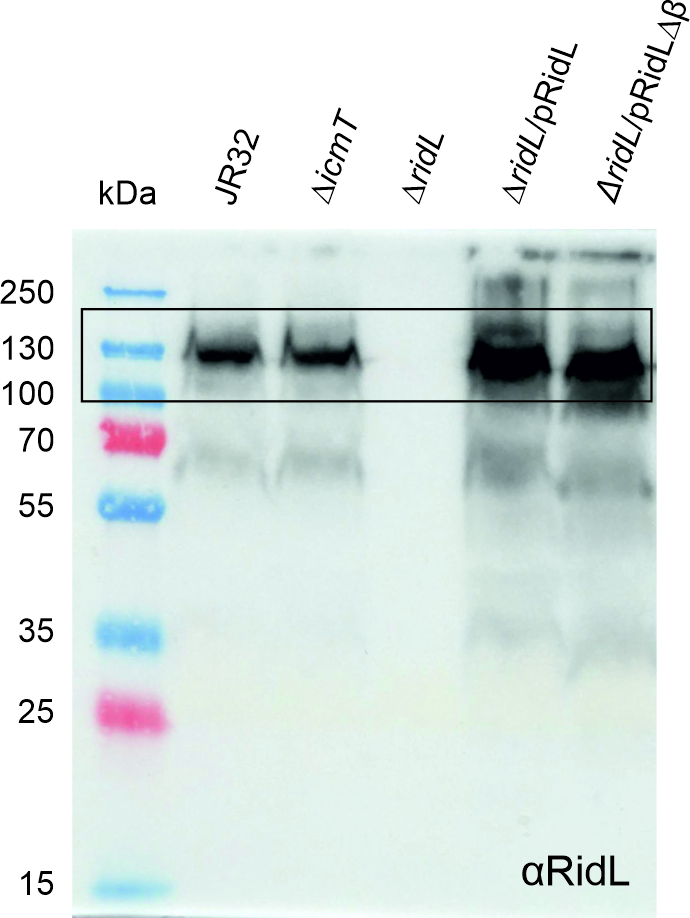

Supplement: Supplementary file 8 — Source data Fig. 6 [file 44319_2026_823_MOESM8_ESM.zip › Fig. 6/6D/anti-ridL western blot-2.tif]
