# Supplementary material for: The Legionella effector RidL binds the large fission GTPase Drp1 to promote mitochondrial fragmentation
Source: EMBO Rep. 2026 Jun 6;27(13):3759–87. doi: 10.1038/s44319-026-00823-3 (PMC13354802; doi:10.1038/s44319-026-00823-3)
Supplement: Supplementary file 9 — Expanded View Figures [file 44319_2026_823_MOESM9_ESM.pdf]

Expanded View Figures

Figure EV1. Binding of RidL to Drp1 and effect on Drp1 GTPase activity.

(**A**) Comparison of the structure of the N-terminal fragment (213 amino acids) of RidL (PDB code: 5OH5, red) with the corresponding AlphaFold model (blue); ribbon overlay (left image) and cartoon overlay (right image). (**B**) Proteomics data identifying Vps35 and Drp1 as binding partners of RidL or $RidL_{\Delta\beta}$, respectively (screenshot). (**C**) Purified RidL (131 kDa), $RidL_N$ ($RidL_{1-258}$, 30 kDa), or $RidL_C$ ($RidL_{437-1100}$, 75 kDa) were incubated with purified Drp1 (78 kDa) coupled to UltraLink Biosupport beads. The beads were washed once, and bound proteins were separated by SDS–PAGE and visualized by anti-RidL Western blot. Regular (left panel, Fig. 1G) and prolonged exposure (right panel) is shown. (**D**) Densitometrical quantification of RidL bound to Drp1. Purified Drp1 was immobilized onto polyacrylamide beads and incubated with purified RidL or $RidL_{\Delta\beta}$ preincubated or not with purified Vps29. After washing, bound proteins were eluted, separated by SDS–PAGE, and visualized by silver staining. Band intensities corresponding to RidL were quantified by densitometry and normalized for each replicate to Drp1-bound RidL in the absence of Vps29. Bars indicate mean ± SEM from three biological replicates ($n = 3$). (**E**) Scheme of coupled enzyme assay for Drp1 GTPase activity, adapted from (Ingerman and Nunnari, 2005). (**F**) Graphical depiction of continuous NADH depletion over time reflecting Drp1 GTPase activity ($n = 3$) (means of two technical duplicates each from three biological replicates).

 © The Author(s)

A

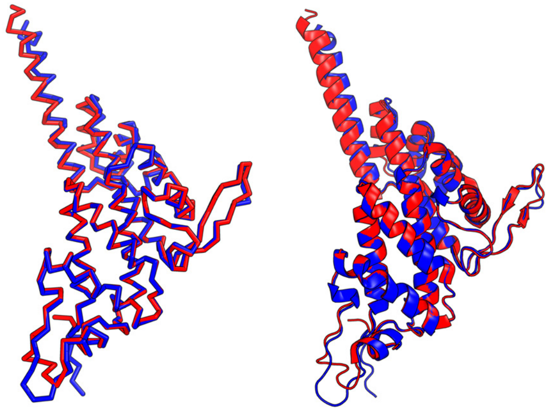

C

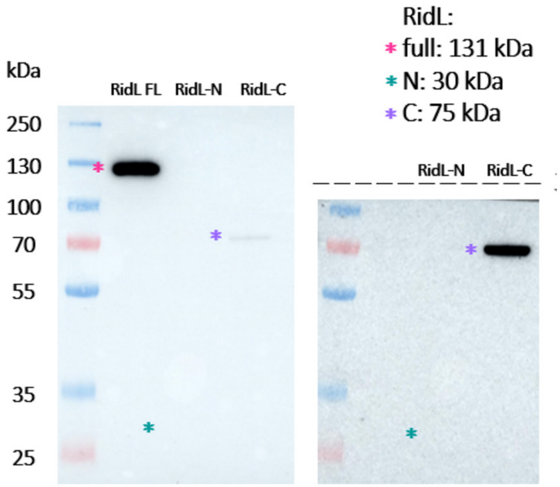

B

Sequence coverage	Protein	Accession	Bio Sample	Prob	%Spec	#Pep	#Unique	#Spec	%Cov	m.w.
	Vacuolar protein sorting-associated protein 35 OS=Homo sapiens OX=9606 GN=VPS35 PE=1 SV=2	sp|Q96QK1|VPS35_HUMAN	*	100%	0.033835	4	4	9	0.06281407	91710 Da
	Dynamin-1-like protein OS=Homo sapiens OX=9606 GN=DNM1L PE=1 SV=2	sp|O00429|DNM1L_HUMAN	>	100%	0.157895	15	16	36	0.16168478	81879 Da

D

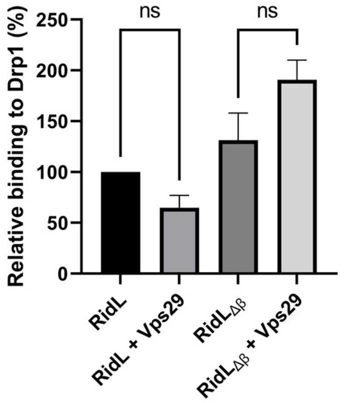

F

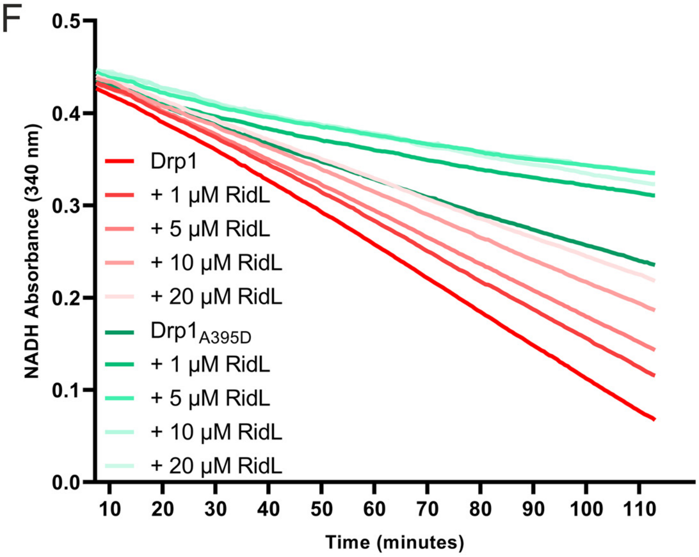

E

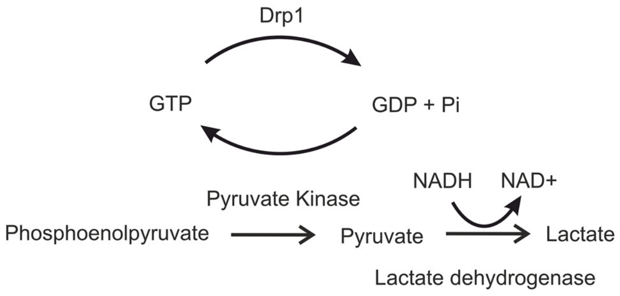

A

		Drp1			Drp1 + RidL			Drp1 + SidC	
	Z-average (nm)	PDI	1st peak (nm)	Z-average (nm)	PDI	1st peak (nm)	Z-average	PDI	1st peak (nm)
0 minutes	28	0.48	24	34	0.15	16	21	0.25	16
40 minutes	40	0.38	49	31	0.43	58	35	0.45	62

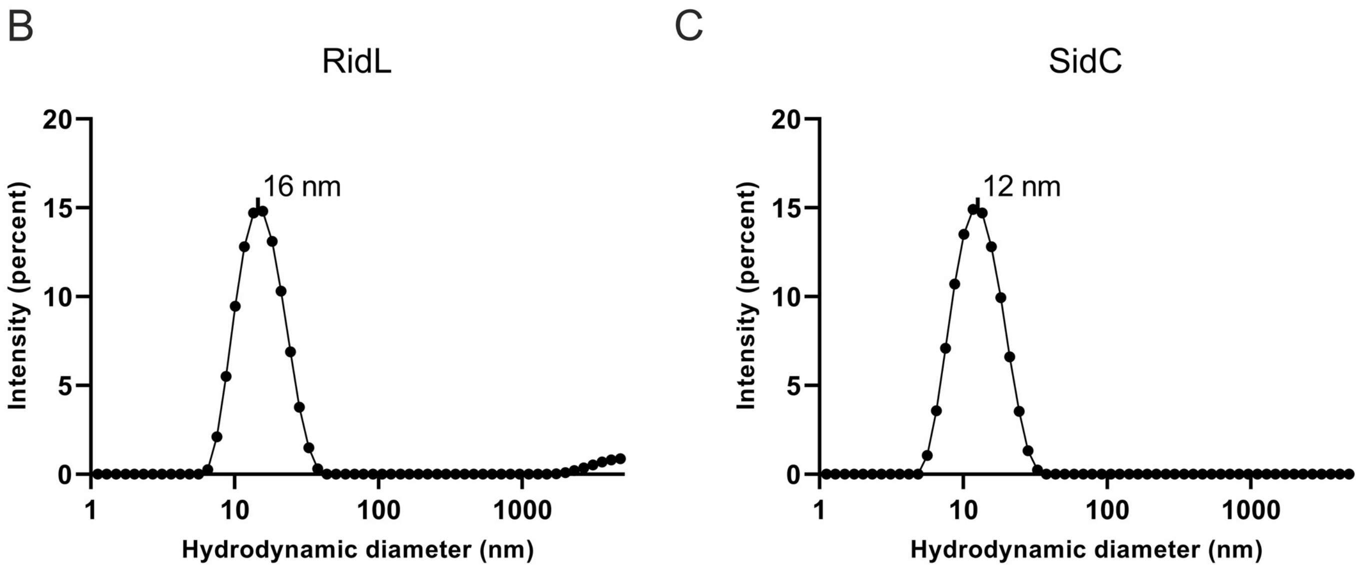

Figure EV2. Dynamic light scattering values and profiles.

(**A**) Hydrodynamic diameter (in terms of Z-average) and polydispersity index (PDI) from CUMULANT analysis, and mean value of the first distribution peak from CONTIN analysis of 1 µM Drp1 in the presence of 0.5 mM GMPPCP, in the absence ("Drp1") or presence of 3 µM RidL ("Drp1 + RidL") or 3 µM SidC ("Drp1 + SidC"). PDI, polydispersity index. Representative dynamic light scattering (DLS) profiles of (**B**) RidL (10 µM) or (**C**) SidC (10 µM) are shown (30 min).

 © The Author(s)

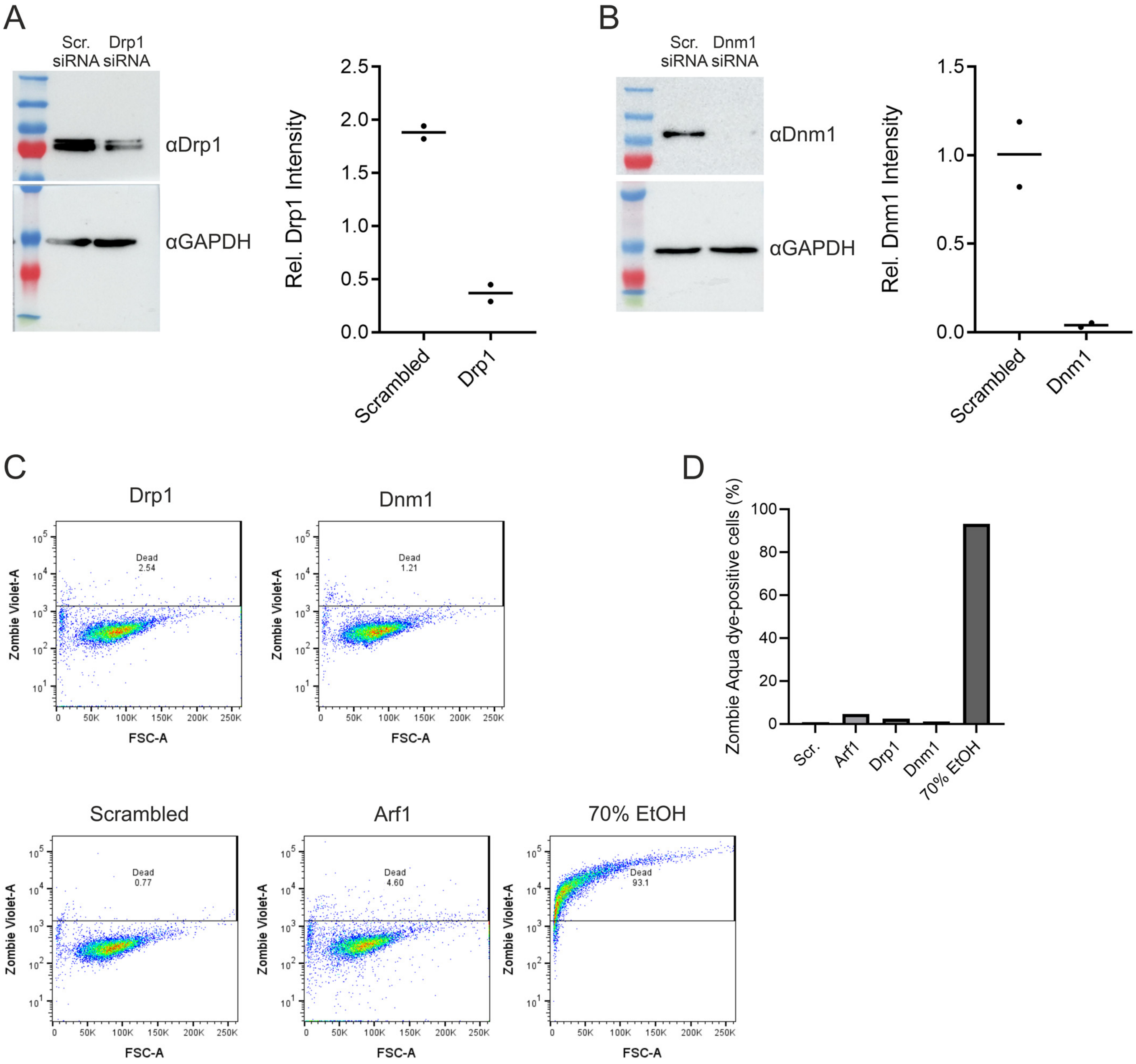

Figure EV3. Drp1 but not Dnm1 affects intracellular replication of *L. pneumophila*.

Efficiency of siRNA-mediated depletion of (**A**) Drp1 or (**B**) Dnm1 was assessed by Western blot (left panels). Quantification of the Drp1 or Dnm1 and GAPDH signal intensities of cells treated with Drp1- or Dnm1-specific or scrambled siRNA were calculated in ImageJ (right panels). Graphs show the Drp1 or Dnm1 signal intensities normalized to GAPDH from two biological replicates ($n = 2$). (**C, D**) Cytotoxicity was assessed in cells transfected with siRNA or treated with 70% EtOH (30 min, 37 °C) by staining with Zombie Aqua dye (1:500 dilution, 30 min). After staining, cells were fixed with 4% PFA and processed by flow cytometry with forward scatter (FSC) voltage 400, sideward scatter (SSC) voltage 250 and laser 525/50 (Vio510) voltage 380 (>10,000 events per sample). Data show one biological replicate ($n = 1$). (**C**) Scatter plots (indicated line shows the set threshold for live/dead cells) of FSC vs. Zombie Aqua dye fluorescence and (**D**) bar graph of Zombie Aqua dye-positive cells are shown.

© The Author(s)

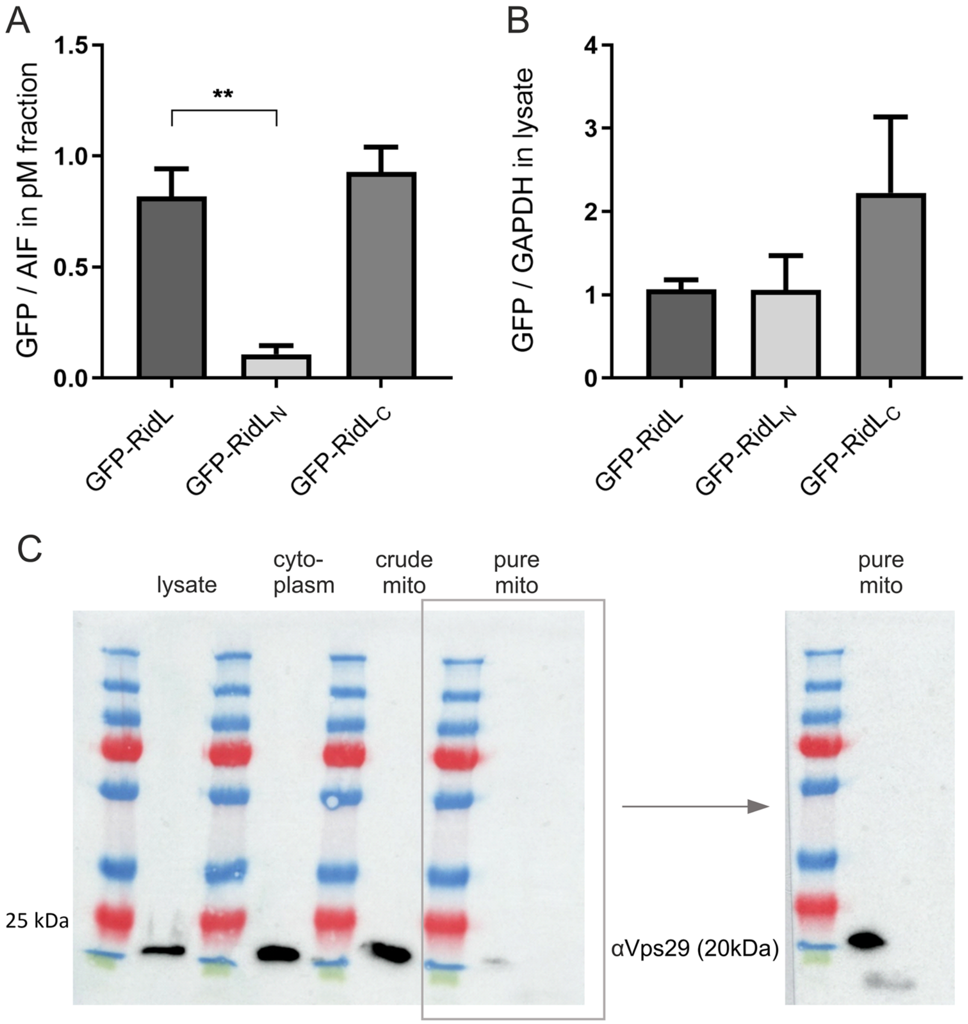

Figure EV4. Localization of RidL and retromer.

(**A**) Quantification of (Fig. 4C). Signal intensities of GFP and AIF were calculated by ImageJ. Bars show the relative GFP signal in pure mitochondria (pM) fractions. Means + SEM of three biological replicates are shown (one-way ANOVA, ** $p < 0.01$). (**B**) Detection of GFP-RidL, GFP-RidL_N, and GFP-RidL_C in HEK293 cell lysate (additional information for Fig. 4C). Signal intensities of GFP-RidL, GFP-RidL_N, GFP-RidL_C and GAPDH in HeLa cell lysate were assessed by ImageJ. Graphs show the means + SEM of relative GFP intensities (GFP/GAPDH) of three biological replicates. If not indicated otherwise, the data were not significantly different. (**C**) Localization of the Vps29 subunit of the retromer cargo recognition subcomplex in HeLa cell lysates (lysate), cytoplasmic (cytoplasm), and crude/purified mitochondrial (crude/pure mito) fractions. Western blot with anti-VPS29 Antibody (sc-398874, Santa Cruz Biotechnology, 1:100) and secondary antibody goat anti-mouse IgG [H + L] HRP-linked secondary antibody (Invitrogen; 1:5000). Regular (left panel) and prolonged exposure (right panel) is shown.

 © The Author(s)

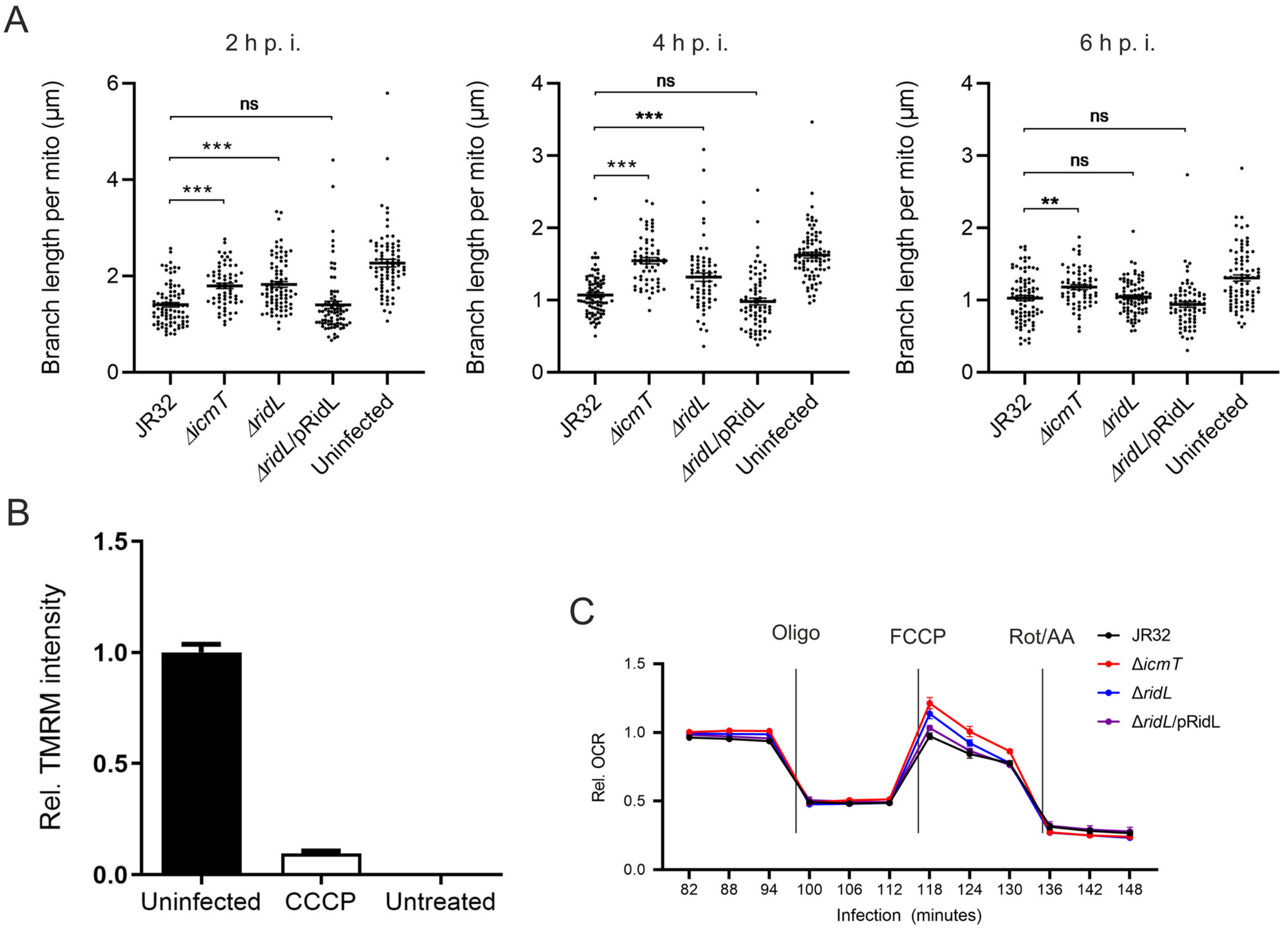

Figure EV5. RidL modulates mitochondrial fragmentation and function in *L. pneumophila*-infected cells.

(**A**) Mitochondrial branch lengths are quantified in infected cells (Fig. 5A). In infected cells, the average branch lengths per mitochondrion were assessed using the Mitochondria Analyzer plugin for ImageJ. Dots represent all analyzed cells from three biological replicates ($n = 3$) (20–30 cells each; means + SEM; one-way ANOVA, **$p < 0.01$; **$p < 0.001$; ****$p < 0.0001$). (**B**) Controls for TMRM assays shown in Fig. 5C. HeLa cells were treated with 50 µM CCCP (5 min, 37 °C). Uninfected and CCCP-treated cells were stained with tetramethyl rhodamine methyl ester (TMRM; 20 nM, 30 min, 37 °C), untreated cells were neither infected nor stained. TMRM intensities, reflecting the membrane potential, were assessed by flow cytometry with FSC voltage 420, SSC voltage 280 and YG610/20 voltage 400 (TMRM) (>10.000 events per sample). Data was processed in FlowJo and normalized to the average TMRM signal intensity of uninfected cells. Graphs show means + SEM of three biological replicates ($n = 3$; untreated mean: 0.000493). (**C**) Oxygen consumption of infected HeLa cells, as shown in Fig. 5D. Oxygen consumption rates (OCR) of uninfected HeLa cells were measured 2×. HeLa cells were infected (MOI 10) with *L. pneumophila* strains JR32, Δ*icmT*, Δ*ridL*, or Δ*ridL*/pRidL (pNT28 or pIF009), subjected to a mitochondrial stress test, and the OCR was measured every 6 min in 15 cycles (3:00 mix, 0:00 wait, 3:00 measure). Oligomycin (final concentration 1 µM), FCCP (0.5 µM) and rotenone/antimycin A (0.5 µM) were injected at the indicated time points. OCR values were normalized to the average OCR of the last two time points measured before the stress test. Graphs show the OCR during infection, normalized to OCR values before infection. Means + SEM of three biological replicates ($n = 3$).

© The Author(s)
