# Supplementary material for: The Legionella effector RidL binds the large fission GTPase Drp1 to promote mitochondrial fragmentation
Source: EMBO Rep. 2026 Jun 6;27(13):3759–87. doi: 10.1038/s44319-026-00823-3 (PMC13354802; doi:10.1038/s44319-026-00823-3)
Supplement: Supplementary file 2 — Peer Review File [file 44319_2026_823_MOESM2_ESM.pdf]

EMBO reports

The Legionella effector RidL binds the large GTPase Drp1 to promote mitochondrial fragmentation

Ana Katic, Elizabeth Vittori, Stefanie Halter, Kevin Steiner, A. Swart, Milad Radiom, Francisco-Javier Garcia-Rodriguez, Tobias Jäggi, Xiaodan Li, Jason Mears, Carmen Buchrieser, Pedro Escoll, Vikram Panse, and Hubert Hilbi

Corresponding author(s): Hubert Hilbi (hilbi@imm.uzh.ch)

Review Timeline:

Transfer Date:	5th Dec 25
Editorial Decision:	15th Dec 25
Revision Received:	12th Apr 26
Editorial Decision:	11th May 26
Revision Received:	21st May 26
Accepted:	28th May 26

Editor: Achim Breiling

Transaction Report: This manuscript was transferred to EMBO Reports following peer review at Review Commons.

Review COMMONS

(Note: With the exception of the correction of typographical or spelling errors that could be a source of ambiguity, letters and reports are not edited. Depending on transfer agreements, referee reports obtained elsewhere may or may not be included in this compilation. Referee reports are anonymous unless the Referee chooses to sign their reports.)

Revision Plan

Manuscript number: RC-2025-03255
Corresponding author(s): Hubert Hilbi

1. General Statements

In our study "The *Legionella* effector RidL binds the large fission GTPase Drp1 to promote mitochondrial fragmentation" we identify the mitochondrial large fission GTPase Drp1 and closely related GTPases as novel interactors of the *L. pneumophila* effector protein RidL. Drp1 is bound by a C-terminal fragment of the effector, the function of which was previously unknown. Moreover, we document that RidL promotes mitochondrial fission, reduction of mitochondrial membrane potential, and phosphorylation activation of Drp1. Our study reveals the first bacterial effector protein targeting eukaryotic large fission GTPases.

We would like to thank the three reviewers for their thorough, constructive and overall positive evaluation of our work. We appreciate the general assessment of the reviewers stating that "This study convincingly demonstrates an interaction between RidL and Drp1. Additionally, it shows a role for RidL in mitochondrial fragmentation, reduced mitochondrial membrane potential and oxygen consumption during *Legionella* infection, and is of interest to a specialized audience working on intracellular microorganisms." (**reviewer #1**), "Readers in basic research of bacterial pathogenesis and mitochondria biology would be interested" (**reviewer #2**), and "This is an interesting study that adds to the growing understanding of how *L. pneumophila* effectors target mitochondria. This is a growing and significant area of interest in the host-pathogen space." (**reviewer #3**).

We believe that the valid and important points raised by the three reviewers can be experimentally addressed in a major revision of the manuscript. To further strengthen our manuscript, we propose to perform the following experiments:

1. Determine the role of the retromer for binding of Drp1 to RidL. We currently assess binding of RidL and RidLΔβ to Drp1 in absence or presence of purified Vps29. This experiment is ongoing and provided the intriguing result that the presence of Vps29 decreased binding of RidL to Drp1 but not binding of RidLΔβ to Drp1.

2. Determine binding of Drp1 by RidL in infected cells. We currently perform co-IPs using anti-RidL antibody, protein A/G beads and lysates of HeLa cells infected with *L. pneumophila* JR32 wild-type, ΔicmT, ΔridL, and complemented ΔridL mutant strains. We will include anti-Vps29 (or anti-Vps35) as controls in the pulldown / co-IP experiments.

3. Localization of RidL to the mitochondrial outer membrane. We currently perform protease protection assays on mitochondria isolated HeLa cells infected with *L. pneumophila* JR32 wild-type, ΔicmT, ΔridL, and complemented ΔridL mutant strains and assess proteinase K susceptibility of mitochondria-localizing RidL.

4. Control for the production of GFP-fusion proteins in the bimolecular fluorescence complementation experiment.

5. Control for the production of RidL and mutant proteins in *L. pneumophila* strains.

2. Description of the planned revisions

Reviewer #1:
None

Reviewer #2:

2. Fig. 1:

a. Fig. 1B-F: The pulldown assays using recombinant proteins and HeLa cell lysate produced different results. It seems that full-length RidL interacts when using only recombinant proteins (in vitro pulldown) but the interaction was much more reduced in the presence of other cellular proteins (HeLa lysate). This raises a question whether this RidL/Drp1 interaction actually occurs within host cells. Although the Y2H experiment showed that RidL interacts with Drp1, the result still does not directly support the interaction in the natural host amoeba or mammalian cells. The authors should perform the pulldown assays in Lp-infected cells. Vps29 can be included as positive and negative controls for the experiments in 1B to 1F.

Response: Thank you for this suggestion. In ongoing experiments, we perform co-IPs using anti-RidL antibody, protein A/G beads and lysates of HeLa cells infected with *L. pneumophila* JR32 wild-type, Δ*icmT*, Δ*ridL*, and complemented Δ*ridL* mutant strains. We will include anti-Vps29 (or anti-Vps35) as controls in the pulldown / co-IP experiments. The results will be shown in **Fig. 1** (see also **reviewer #3, point 2**).

4b. Fig. 3B: Expression of the GFP fusion proteins should be determined.

Response: As suggested by the reviewer, we will determine the production of the GFP fusion proteins by anti-GFP Western blot (**Fig. 3B**).

5a. Expression of RidL in the Lp strains should be determined.

Response: The production of RidL in the *L. pneumophila* strains has been determined by anti-RidL Western blot (Finsel et al., 2013). While *L. pneumophila* JR32 wild-type, and Δ*icmT* produce similar amounts of RidL, the Δ*ridL* mutant produced none (as expected), and the complemented Δ*ridL* mutant strain produced ca. 3x more RidL than the parental strain. In the

revised version of the manuscript, we will include a WB showing the production of RidL, in particular of the strains Δ*ridL* (negative control), Δ*ridL*/pRidL and Δ*ridL*/pRidL$_{\Delta\beta}$.

6b. Fig. 6C: This result indicates that binding to Vps29 might interfere with phosphorylation of Drp1. This further encourages the authors to determine if the interactions with Drp1 and Vps29 can occur simultaneously and whether loss of Vps29 binding increases Drp1 interaction (and/or phosphorylation).

Response: We now assess binding of RidL and RidL$_{\Delta\beta}$ to Drp1 in absence or presence of purified Vps29 (**new Fig. 1G**). Intriguingly, the presence of Vps29 decreased binding of RidL to Drp1 but not binding of RidL$_{\Delta\beta}$ to Drp1.

Reviewer #3:

[5] Figure 4E and F need to be quantified or shown with western blot. The western would be more convincing.

Response: The microscopy data shown in **Fig. 4EF** is quantified in **Fig. GH**. As suggested by the reviewer, we will isolate mitochondria and perform Western blots anti-Drp1 and anti-Tom20. The results will be shown in **Fig. 4**.

[8] Localization data need stronger validation. Evidence for mitochondrial localisation of RidL is strong but would be good to show it is on the outer membrane to support an interaction with Drp1. Additional controls, such as protease protection assays or carbonate extraction, could clarify whether RidL is peripherally associated with the outer membrane or internalised.

Response: Thank you for this suggestion. We will perform protease protection assays on mitochondria isolated from *L. pneumophila*-infected cells. To this end, mitochondria from HeLa cells infected with *L. pneumophila* JR32 wild-type, Δ*icmT*, Δ*ridL*, and complemented Δ*ridL* mutant strains, will be isolated, treated with proteinase K or not, and the amount of RidL will be assessed by anti-RidL Western blot. The inner membrane protein Opa1 will be used as a protease-inaccessible negative control in anti-Opa1 Western blots.

3. Description of the revisions that have already been incorporated in the transferred manuscript

Reviewer #1:

- 1. Line 94: "The experimental results closely match the Alphafold2 structure prediction (Fig. 1A)..." How is closely matching defined? The authors should include a structural alignment between the alphafold structure and experimental structures and report a RMSD value or a figure of the structural alignment.

Response: As suggested by the reviewer, we included a structural alignment (**new Fig. S1A**) and report a RMSD value for the overlay (**l. 94**).

- 2. When describing the dynamic light scattering data in Fig. 2B the text is not clear about how the data is interpreted as supporting a role of RidL in inhibiting Drp1 oligomerization. Additionally, the conclusion that the peak of the distribution increasing from 44 nm in the Drp1 only sample to 51 nm in the Drp1+RidL sample suggests the presence of a Drp1-RidL complex isn't supported when the Drp1+SidC sample has a further shifted peak of 59 nm despite there being no known interaction between Drp1 and SidC.

Response: We clarified this point by adding explanatory sentences in the Results section (**l. 172-174**) and the Discussion section (**l. 366-374**). The addition of RidL, but not SidC, affects the symmetrical distribution of complexes resulting in a broad and rather irregular distribution of hydrodynamic radii. Moreover, in presence of Drp1, SidC but not RidL shows a peak at the hydrodynamic radius of the effector alone. See also **reviewer #2, point 3**.

- 3. Line 276: The authors state: "Co-localization of Drp1 with MitoTracker was restored by supplying plasmid-produced RidL or RidLΔβ-loop." However, figure 6B shows complementation with wild type RidL has no impact on p-Drp1-mitochondrial co-localization, while the RidLΔβ-loop complementation only partially restores the phenotype. Clarify this in the text.

Response: Thank you for pointing out this inaccuracy. We corrected the text accordingly (**l. 293-294**, **l. 301-302**). See also **reviewer #2**, minor points.

- 4. Quantification of microscopy data in Figures 3C, 4GH, and 6B are not adequately described. There is not enough information in either the figure legends or methods section to know how the quantification was performed.

Response: We outlined the quantification of the microscopy data in the Methods section (**l. 739-748**). Moreover, we added information on the construction of five plasmids, which were missing previously (**l. 490-495**).

- 5. Line 327: "Our study provides novel links between the retromer and Drp1..." I'm not sure what data in the paper provides any link between retromer and Drp1. In separate papers, the authors have shown that RidL interacts with Drp1 and the retromer, however, they have not performed experiments to determine how/if RidL interacts with both concurrently or if they are separate events.

Response: We assessed binding of RidL and $RidL_{\Delta\beta}$ to Drp1 in absence or presence of purified Vps29 (**new Fig. 1G**). Intriguingly, the presence of Vps29 decreased binding of RidL to Drp1 but not binding of $RidL_{\Delta\beta}$ to Drp1 (see also below, **reviewer #2, points 2b and 6b**). Accordingly, we indeed show that "Our study provides novel links between the retromer and Drp1 …" (**l. 345-346**).

- 6. The description of statistics states that two-tailed T tests were used. There should also be additional multiple hypotheses correction performed for the analysis of data within each experiment (or ANOVA with posthoc testing).

Response: We performed and documented ANOVA statistics throughout the manuscript.

Reviewer #2:
Major comments:

1. Suggest changing the title: The results did not directly demonstrate phosphorylation of Drp1 caused by RidL interaction promotes mitochondrial fragmentation. The presenting results show RidL interacts with Drp1 and RidL increases Drp1 phosphorylation. If the authors want to make the claim of "through phosphorylation

activation of Drp1", they should use a phospho-resistant Drp1 mutant in the infection experiment with wildtype Lp.

Response: We would like to thank the reviewer for pointing out this inaccuracy, and we changed the title as suggested: “The *Legionella* effector RidL binds the large fission GTPase Drp1 to promote mitochondrial fragmentation”.

2. According to the figure legend, the asterisks indicated the protein bands analyzed by mass spectrometry. However, there was no mass spec. data provided. Are both bands identified as Drp1? The two bands showed different Mw sizes, suggesting they are either different proteins or post-translationally modified differently. However, according to Fig. 6, both full length RidL and the Δbeta loop mutant increase Drp1 phosphorylation. Please clarify.

Response: We included the MS data (**new Fig. S1B**). Moreover, in order to unequivocally indicate the different protein bands retained by RidL (Vps35) or $RidL_{\Delta\beta}$ (Drp1), we labelled the two band with different symbols (**Fig. 1B**). We clarify in the Discussion section (**l. l. 361-365**) the observation that RidL and – even more efficiently – $RidL_{\Delta\beta}$ complement the Δ*ridL* mutant phenotype (**Fig. 6C**).

2b. Fig. 2B: I am not an expert in dynamic light scattering; however, the results in Fig. 2B were difficult to understand. Adding RidL or SidC caused shifts of the peak to 51 nm or 59 nm. However, the data did not help with the authors' claim that RidL disrupts Drp1 oligomerization. Although the authors speculated that SidC caused bigger Drp1 oligomers and RidL form complexes with Drp1, no direct evidence was shown.

Response: We clarified this point by adding explanatory sentences in the Results section (**l. 172-174**) and the Discussion section (**l. 366-374**). The addition of RidL, but not SidC, affects the symmetrical distribution of complexes resulting in a broad and rather irregular distribution of hydrodynamic radii. Moreover, in presence of Drp1, SidC but not RidL shows a peak at the hydrodynamic radius of the effector alone. See also **reviewer #1, point 2**.

5b. Fig. S5: The GFP images for Tom20 and Lp fluorescence images were very dim, making them difficult to see.

Response: We enhanced the brightness of the images (**Fig. S5, S7**), and in Fig. S7 we used fluorescence colors more easily recognized by color-blind people (see **reviewer #3, point 14**).

c. Fig. 4G, 4H: Did the ΔridL_ridL strain significantly restore co-localization of mitochondria with Drp1 or Tom20?

Response: The complemented Δ*ridL* mutant restored co-localization of mitochondria with Tom20 but not with Drp1 in a statistically significant manner. A tendency was also leaning toward restoring the co-localization of mitochondria with Drp1. The statistics are now indicated in **Fig. 4GH**.

6. Fig. 6:

a. Fig.6A, S7: The Fluor-488 image of Δ*icmT* showed strong signals for phospho-Ser616 Drp, even stronger than Δ*ridL-ridL* and Δ*ridL*_ridLΔbeta-loop. These observations contradicted Fig. 6C. The author should use a different method to measure phosphorylation of Ser616 Drp1 (ex: immunoblotting since the phospho-antibody is available and applicable based on the vendor's website).

Response: We repeated the experiments shown in **Fig. 6** and now show that upon infection with *L. pneumophila* wild-type, approximately 50% of the detected phospho-Drp1 localized with the mitochondrial stain MitoTracker, while this was the case for less than 10% or 20% of the detected phospho-Drp1 upon infection with *L. pneumophila* Δ*icmT* or Δ*ridL*, respectively. Plasmid-produced RidL or $RidL_{\Delta\beta}$ did not restore the colocalization of phospho-Drp1 and MitoTracker (**Fig. 6B**). Moreover, we unsuccessfully tried to quantify pDrp1 by Western blot. While we followed the manufacturer's instruction, not even the positive control provided by the company worked. Finally, we enhanced the brightness of the images (**Fig. S7**), and we used fluorescence colors more easily recognized by color-blind people (see **reviewer #3, point 14**).

Minor comments:

1. Fig. 1E: I assumed the bands slightly above 70 kD were recombinant Drp1. Please mark it in the figure.

Response: We marked the band of recombinant Drp1 in **Fig. 1E** as suggested.

2. Line 125-127 and Fig. 1H: The authors described "...the activity was reduced less than two-fold..." and "reduced the GTPase activity of Drp1A395D by approximately two-fold". Could the authors convert the estimated fold change to % of no RidL control? Ex: in the presence of 20x excess RidL, the GTPase activity of wildtype Drp1 is X % of Drp1 without RidL.

Response: We now quantify in percentage of control (**l. 135-136**).

3. Fig. 6B, Line 276-277: ΔridL_ridL did not seem to restore co-localization of phosphor-Drp1 with mitochondria.

Response: Thank you for pointing out this inaccuracy. We corrected the text accordingly (**l. 293-294**, **l. 301-302**). See also **reviewer #1, point 3**.

4. Line 317: not "hrough". Do you mean "through"?

Response: We corrected the typo (**l 335**).

Reviewer #3:

[1] Amount of RidL_C that binds to purified Drp1 is only a fraction of that of WT RidL (Figure 1F). If the C-terminal alone was facilitating the interaction with Drp1, significantly more RidL_C should be observed. Authors should repeat and compare unbound vs bound fractions to determine the proportion of RidL_C that is actually interacting with Drp1.

Response: Thank you for raising this point. We agree that compared to full length RidL only a fraction of RidL_C binds to Drp1 (**Fig. 1F**). However, even upon over-exposure of the Western blot, RidL_N does not bind at all to Drp1 (**Fig. S1C**), while the input amount of RidL, RidL_N, and RidL_C is the same (**Fig. 1D**). We now state this in the text (**l. 118-119**).

[3] The authors demonstrate that RidL promotes mitochondrial fragmentation in vivo by showing that cells infected with WT and ΔridL_ridL *L. pneumophila* have lower

mitochondrial aspect ratios and reduced oxygen consumption rates compared to uninfected or ΔridL-infected cells (Figure 5). This contradicts earlier findings that RidL prevents Drp1 oligomerisation and tubulation in vitro (Fig 2), which is required for mitochondrial fission.

Response: In the biochemical reconstitution and oligomerization experiments (**Figs. 1**, **2**), the unidentified host kinase activating Drp1 is missing (**Fig. 6**). We have now outlined this point in the Discussion section (**l. 366-369**).

[4] Figure 4B should be probed with a *Legionella* antibody to ensure this signal is not coming from bacteria. The amount of *Legionella* secreted RidL looks excellent by western blot, complement this via IFA to ensure specificity.

Response: The RidL signal cannot come from (lysed) bacteria, since there is no signal detected (in the cytoplasm or mitochondrial fraction) of cells infected with Δ*icmT* mutant bacteria, which do not secrete any effector proteins (**Fig. 4B**). Thus, the detection of RidL by Western blot in the cytoplasm or in the mitochondrial fraction of host cells requires a functional T4SS and (obviously) the presence of RidL.

[7] Physiological relevance of the in vitro Drp1 inhibition is uncertain. The inhibition of Drp1 GTPase activity by RidL requires a 20-fold molar excess and results in less than a twofold reduction in activity (Figure 1H-I). This weak inhibition raises doubts about its significance in cells. Authors need to comment on this and provide evidence that the effect is specific rather than due to protein crowding or aggregation.

Response: In the biochemical reconstitution and oligomerization experiments (**Figs. 1**, **2**), the unidentified host kinase activating Drp1 is missing (**Fig. 6**). We have now outlined this point in the Discussion section (**l. 366-374**). Moreover, we have relegated some of the GTPase activity data (graphical depiction of continuous NADH depletion; former **Fig. 1I**) to the Supplementary Information section (**Fig. S1D**).

Minor points:

[9] Why is there a considerable difference in GTPase activity of the same conditions (0 µM and 20 µM RidL) between Fig 1G and Fig 1H, especially since the small error bars suggest that there is otherwise minor variability between independent replicates?

Response: Due to availability and technical reasons, the experiments had to be done with two different plate readers.

[10] On graphs that depict the mean of replicate experiments, display the individual data points (e.g. Fig 1G, Fig 1H, Fig 4A, Fig 6).

Response: The individual data points have been added to the **Figs. 1IJ**, **Fig. 3C**, and **Fig. 6C**.

[11] Legend details of Fig 6C do not match those in the method. Can the authors clarify if (and if so, why) the cells were treated with MTDR for flow cytometry?

Response: Thank you for pointing out this oversight. The legend of **Fig. 6C** has been corrected.

[12] Line 207-213. Keep nomenclature consistent with previous text & figures, i.e. RidL_N and not RidL9-258.

Response: $RidL_N$ ($RidL_{1-258}$) is not identical to $RidL_{9-258}$ and $Ridl_C$ ($RidL_{437-1100}$) is not identical to GFP-$RidL_{259-1167}$.

[13] Line 317. Fix spelling, 'hrough'.

Response: Corrected (**l. 335**).

[14] Consider using colourblind-friendly palettes for immunofluorescence microscopy images.

Response: We enhanced the brightness of **Fig. S7** and used fluorescence colors more easily recognized by color-blind people.

[15] Increase the size of microscopy scale bar text or state the scale bar size in the figure legends.

Response: The length of all microscopy scale bars in now indicated in the figure legends.

4. Description of analyses that authors prefer not to carry out

Reviewer #1:
None.

Reviewer #2:
2b. The authors may also consider using the RidLΔbeta variant in the GTPase activity experiment, which may inform whether a stronger RidL interaction leads to more inhibition of the GTPase activity.

Response: In the *in vitro* GTPase assays shown in **Figs. 1IJ, S1E**, Vps29 as well as the unidentified Drp1 kinase are missing. Accordingly, we believe that adding $RidL_{\Delta\beta}$ to Drp1 in these GTPase assays might not be informative.

3a. Fig. 2A: Although SidC was used as a negative control, have the authors tried RidL-N, RidL-C, and RidL-Δbeta in the EM experiments? Results from these variants would establish a correlation of the Drp1 affinity with formation of the Drp1 oligomers.

Response: In the in vitro filamentation assays shown in **Fig. 2A** the unidentified Drp1 kinase is missing. Accordingly, we believe that adding RidL fragments or $RidL_{\Delta\beta}$ to Drp1 in the filamentation assays might not be informative.

4. Fig. 3:

a. Fig. 3A, Line 170-172:

- Yeast colonies were formed by cells co-expressing RidL with Dnm2, Dnm3 or DymB on -His plates. The negative controls (no RidL) were clearly no colony formation. Therefore, stating no interactions with these proteins was not appropriate. While the size and number colonies might correlate with the binding affinity, the fusions might interfere with the interactions. The authors should cautiously interpret this result.

- Given that Dnm2, Dnm3, and DymB still formed colonies, the authors should consider a more distally related GTPase as a negative control, such as DlpA.

Response: As suggested by the reviewer, we down-tone the statements regarding the interactions (**l. 185**). YTH screens are semiquantitative, false positives are frequently a problem (hence the addition of AT), and mutants might arise. Therefore, some background colonies grow even under conditions where there no or little bait-prey interactions occur.

We performed the YTH screens using RidL as bait and large GTPases as prey to possibly identify large GTPases that bind RidL with higher affinity than Drp1 (**Fig. 3A**). Among the mammalian large GTPases Drp1 bound strongest to RidL, while the *Dictyostelium* large GTPase DymA (but not DymB) and the yeast GTPase Vps1 bound RidL even stronger. Given this aim and results, we believe it is more productive to focus on the validation of positive hits (Drp1) than testing a large GTPase that likely will bind to RidL with lower affinity.

Reviewer #3:

[6] Mechanistic link between RidL and Drp1 phosphorylation is incomplete. While RidL expression increases Ser616 phosphorylation of Drp1, it remains unclear how this occurs. The co-immunoprecipitation experiments suggested above might help to identify the host signalling components responsible for this modification.

Response: We would like to thank the reviewer for pointing out this knowledge gap. While the identification of the host kinase responsible for Drp1 phosphorylation in the context of an *L. pneumophila* infection is obviously very important and intriguing, we believe that experiments along this line are beyond the scope of the current study. We now refer to the unknown Drp1 kinase in the Discussion section (**l. 366-374**).

Dear Prof. Hilbi,

Thank you for transferring your manuscript from Review Commons to EMBO reports. I now went through the manuscript, the referee reports from Review Commons (attached again below) and your revision plan.

Judging from your revision plan, it seems that you will be able to adequately address the concerns of the referees during a major revision. I thus invite you to revise your manuscript accordingly with the understanding that all referee concerns must be addressed in the revised manuscript and/or in a final detailed point-by-point response, as indicated in your revision plan.

Revised manuscripts should be submitted within three months of a request for revision. Please contact me to discuss the revision (also by video chat) if you have further questions or comments regarding the revision, or should you need additional time.

It is EMBO reports policy to allow a single round of revision only and acceptance of the manuscript will therefore depend on the completeness of your responses included in the next, final version of the manuscript, and of another round of review at EMBO reports, using the same referees.

When submitting your revised manuscript, please also carefully review the instructions that follow below.

PLEASE NOTE THAT upon resubmission revised manuscripts are subjected to an initial quality control prior to exposition to re-review. Upon failure in the initial quality control, the manuscripts are sent back to the authors, which may lead to delays. Frequent reasons for such a failure are the lack of the data availability section (please see below) and the presence of statistics based on n=2 (the authors are then asked to present scatter plots or provide more data points).

When submitting your revised manuscript, we will require:

1) a .docx formatted version of the final manuscript text (including legends for main figures, EV figures and tables), but without the figures included. Figure legends should be compiled at the end of the manuscript text.

2) individual production quality figure files as .eps, .tif, .jpg (one file per figure), of main figures and EV figures. Please upload these as separate, individual files upon re-submission.

The Expanded View format, which will be displayed in the main HTML of the paper in a collapsible format, has replaced the Supplementary information. You can submit up to 6 images as Expanded View. Please follow the nomenclature Figure EV1, Figure EV2 etc. The figure legend for these should be included in the main manuscript document file in a section called Expanded View Figure Legends after the main Figure Legends section. Additional Supplementary material should be supplied as a single pdf file labeled Appendix. The Appendix should have page numbers and needs to include a table of content on the first page (with page numbers) and legends for all content. Please follow the nomenclature Appendix Figure Sx, Appendix Table Sx etc. throughout the text, and also label the figures and tables according to this nomenclature.

For more details, please refer to our guide to authors:
https://link.springer.com/journal/44319/submission-guidelines#cms-Revised-submissions

Please consult our guide for figure preparation:
https://media.springernature.com/original/springer-cms/rest/v1/content/27825798/data/v1

3) a .docx formatted letter INCLUDING the reviewers' reports and your detailed point-by-point responses to their comments. As part of the EMBO Press transparent editorial process, the point-by-point response is part of the Review Process File (RPF), which will be published alongside your paper.

4) a complete author checklist, which you can download from our author guidelines (https://link.springer.com/journal/44319/submission-guidelines#cms-Revised-submissions). Please insert page numbers in the checklist to indicate where the requested information can be found in the manuscript. The completed author checklist will also be part of the RPF.

Please also follow our guidelines for the use of living organisms, and the respective reporting guidelines:
https://link.springer.com/partners/embo-press/editorial-policies#Animal%20and%20human%20studies

5) that primary datasets produced in this study (e.g. RNA-seq, ChIP-seq, structural and array data) are deposited in an appropriate public database. If no primary datasets have been deposited, please also state this in a dedicated section (e.g. 'No primary datasets have been generated and deposited'), see below.

See also: https://link.springer.com/partners/embo-press/editorial-policies#Data%20deposition

Please remember to provide a reviewer password if the datasets are not yet public.

The accession numbers and database should be listed in a formal "Data Availability" section that follows the model below. This is now mandatory (like the COI statement). Please note that the Data Availability Section is restricted to new primary data that are part of this study. This section is mandatory. As indicated above, if no primary datasets have been deposited, please state this in this section

Data availability

The datasets produced in this study are available in the following databases:

- RNA-Seq data: Gene Expression Omnibus GSE46843 (https://www.ncbi.nlm.nih.gov/geo/query/acc.cgi?acc=GSE46843)
- [data type]: [name of the resource] [accession number/identifier/doi] ([URL or identifiers.org/DATABASE:ACCESSION])

*** Note - All links should resolve to a page where the data can be accessed. ***

See also: https://link.springer.com/partners/embo-press/editorial-policies#Data%20availability%20statement

Moreover, I have these editorial requests:

6) We now request the publication of original source data with the aim of making primary data more accessible and transparent to the reader. You will receive a separate email with instructions for providing source data with your revised manuscript, including information how to upload and organize the files.

7) Our journal encourages inclusion of *data citations in the reference list* to directly cite datasets that were re-used and obtained from public databases. Data citations in the article text are distinct from normal bibliographical citations and should directly link to the database records from which the data can be accessed. In the main text, data citations are formatted as follows: "Data ref: Smith et al, 2001" or "Data ref: NCBI Sequence Read Archive PRJNA342805, 2017". In the Reference list, data citations must be labeled with "[DATASET]". A data reference must provide the database name, accession number/identifiers and a resolvable link to the landing page from which the data can be accessed at the end of the reference. Further instructions are available at: https://link.springer.com/journal/44319/submission-guidelines#cms-Reference-guidelines

8) Regarding data quantification and statistics, please make sure that the number "n" for how many independent experiments were performed, their nature (biological versus technical replicates), the bars and error bars (e.g. SEM, SD) and the test used to calculate p-values is indicated in the respective figure legends (also for EV and Appendix figures). Please also check that all the p-values are explained in the legend, and that these fit to those shown in the figure. Please provide statistical testing where applicable. Please avoid the phrase 'independent experiment' but clearly state if these were biological or technical replicates. Please also indicate (e.g. with n.s.) if testing was performed, but the differences are not significant. In case n=2, please show the data as separate datapoints without error bars and statistics. See also: https://link.springer.com/journal/44319/submission-guidelines#cms-Figure-and-data-presentation

If n<5, please show single datapoints for diagrams.

9) Please add scale bars of similar style and thickness to all microscopic images, using clearly visible black or white bars (depending on the background). Please place these in the lower right corner of the images themselves. Please do not write on or near the bars in the image but define the size in the respective figure legend.

10) Please also note our reference format:
https://link.springer.com/journal/44319/submission-guidelines#cms-Reference-guidelines

11) We updated our journal's competing interests policy in January 2022 and request authors to consider both actual and perceived competing interests. Please review the policy https://www.embopress.org/competing-interests and update your competing interests if necessary. Please name this section 'Disclosure and Competing Interests Statement' and put it after the Acknowledgements section.
See: https://link.springer.com/partners/embo-press/editorial-policies#Competing%20interest%20disclosures

12) We now use CRediT to specify the contributions of each author in the journal submission system. CRediT replaces the author contribution section. Please use the free text box to provide more detailed descriptions and do NOT provide your final manuscript text file with an author contributions section. See also our guide to authors (section 'Author contributions'):
https://link.springer.com/journal/44319/submission-guidelines#cms-Revised-submissions

13) All Materials and Methods need to be described in the main text using our 'Structured Methods' format, which is required for

all research articles. According to this format, the Methods section should include a Reagents and Tools Table (listing key reagents, experimental models, software, and relevant equipment and including their sources and relevant identifiers), uploaded as separate file, and a Methods section in which we encourage the authors to describe their methods using a step-by-step protocol format with bullet points, to facilitate the adoption of the methodologies across labs. More information on how to adhere to this format as well as downloadable templates (.doc) for the Reagents and Tools Table can be found in our author guidelines (section 'Structured Methods'):

https://link.springer.com/journal/44319/submission-guidelines#cms-Manuscript-organisation-and-formatting

14) Please order the manuscript sections like this, using only these names:
Title page - Abstract (max. 175 words) - Keywords - Introduction - Results - Discussion - Methods - Data availability section - Acknowledgements (please include here all the funding information) - Disclosure and Competing Interests Statement - References - Figure legends - Expanded View Figure legends

15) Please make sure that all the funding information is also entered into the online submission system and that it is complete and similar to the one in the acknowledgement section of the manuscript text file.

We would also welcome the submission of cover suggestions, or motifs to be used by our Graphics Illustrator in designing a cover.

As part of the EMBO publication's Transparent Editorial Process, EMBO reports publishes online a Review Process File (RPF) to accompany accepted manuscripts. This File will be published in conjunction with your paper and will include the referee reports, your point-by-point response and all pertinent correspondence relating to the manuscript.

You are able to opt out of this by letting the editorial office know (emboreports@embo.org). If you do opt out, the Review Process File link will point to the following statement: "No Review Process File is available with this article, as the authors have chosen not to make the review process public in this case."

I look forward to seeing a revised form of your manuscript when it is ready.

Yours sincerely,

Achim Breiling
Senior Editor
EMBO Reports

Referee #1:

In this paper, the authors identify Drp1 as an interaction partner of the L. pneumophila type 4 secretion system effector RidL both in vitro with co-immunoprecipitation and in vivo with yeast two hybrid assays. With in vitro assays they show RidL interferes with Drp1 GTPase activity and oligomerization. During Legionella infections, they show RidL localizes to mitochondria. Consistent with previous studies, Legionella infection leads to mitochondrial fission and reduced mitochondrial membrane potential and oxygen consumption, phenotypes reduced during infection with a ridL-deficient strain of Legionella. Additionally, the authors show that Tom20, Drp1 and Drp1(p-616) accumulate on MitoTracker stained mitochondria during Legionella infected cells in a partially RidL dependent process.

- Line 94: "The experimental results closely match the Alphafold2 structure prediction (Fig. 1A)..." How is closely matching defined? The authors should include a structural alignment between the alphafold structure and experimental structures and report a RMSD value or a figure of the structural alignment.
- When describing the dynamic light scattering data in Fig. 2b the text is not clear about how the data is interpreted as supporting a role of RidL in inhibiting Drp1 oligomerization. Additionally, the conclusion that the peak of the distribution increasing from 44nm in the Drp1 only sample to 51nm in the Drp1+RidL sample suggests the presence of a Drp1-RidL complex isn't supported when the Drp1+SidC sample has a further shifted peak of 59nm despite there being no known interaction between Drp1 and SidC
- Line 276: The authors state: "Co-localization of Drp1 with MitoTracker was restored by supplying plasmid-produced RidL or RidLΔβ-loop." However, figure 6B shows complementation with wild type RidL has no impact on p-Drp1-mitochondrial co-localization, while the RidLΔβ-loop complementation only partially restores the phenotype. Clarify this in the text.
- Quantification of microscopy data in Figures 3C, 4G,H, and 6B are not adequately described. There is not enough information in either the figure legends or methods section to know how the quantification was performed.
- Line 327: "Our study provides novel links between the retromer and Drp1..." I'm not sure what data in the paper provides any link between retromer and Drp1. In separate papers, the authors have shown that RidL interacts with Drp1 and the retromer,

however, they have not performed experiments to determine how/if RidL interacts with both concurrently or if they are separate events.
- The description of statistics states the two tailed T tests were used. There should also be additional multiple hypotheses correction performed for the analysis of data within each experiment (or ANOVA with posthoc testing)

Significance:

This study reveals a role for the Legionella type 4 secretion system effector RidL in interfering with mitochondrial function. It has previously been shown that Legionella infection disrupts mitochondrial function, and that other translocated effectors were involved, but this is the first time RidL has been implicated in this function. The authors have also previously demonstrated that RidL inhibits the retromer complex during Legionella infection but introduce this as a new role.

This study convincingly demonstrates an interaction between RidL and Drp1. Additionally, it shows a role for RidL in mitochondrial fragmentation, reduced mitochondrial membrane potential and oxygen consumption during Legionella infection, and is of interest to a specialized audience working on intracellular microorganisms. A weakness of the manuscript, is that except for the increase in Drp1 phosphorylation at Ser 616, it is unclear whether the interaction of RidL with Drp1 or the retromer is important for the observed phenotypes. The paper would be strengthened by addressing this concern, and including complementation of ΔridL mutants with RidL-Nterm (only interacts with retromer) and RidLΔβ-loop. For example, one interpretation of the results in Fig 4e-h would be that Legionella is blocking mitochondrial derived vesicle formation (Tom20 is a mitochondrial outer membrane marker and has been used to study MDVs) leading to the accumulation of Tom20 and Drp1 on MitoTracker staining mitochondria. However, the authors state that both the retromer and Drp1 are involved in the formation of MDVs.

Referee #2:

In the manuscript by Katic et al., the authors reported that the Legionella effector RidL promotes mitochondria fragmentation in host cells and identified a potential interacting protein Drp1 by microscopy and biochemical assays. In addition, they found that RidL also promotes phosphorylation of Drp1. These discoveries indicate a new activity of the bacterial effector to manipulate host mitochondria. My specific comments are below:

Major comments:

1. Suggest changing the title: The results did not directly demonstrate phosphorylation of Drp1 caused by RidL interaction promotes mitochondrial fragmentation. The presenting results show RidL interacts with Drp1 and RidL increases Drp1 phosphorylation. If the authors want to make the claim of "through phosphorylation activation of Drp1", they should use a phospho-resistant Drp1 mutant in the infection experiment with wildtype Lp.

2. Fig. 1:

a. Fig. 1B-F: The pulldown assays using recombinant proteins and HeLa cell lysate produced different results. It seems that full-length RidL interacts when using only recombinant proteins (in vitro pulldown) but the interaction was much more reduced in the presence of other cellular proteins (HeLa lysate). This raises a question whether this RidL/Drp1 interaction actually occurs within host cells. Although the Y2H experiment showed that RidL interacts with Drp1, the result still does not directly support the interaction in the natural host amoeba or mammalian cells. The authors should perform the pulldown assays in Lp-infected cells. Vps29 can be included as positive and negative controls for the experiments in 1B to 1F.

According to the figure legend, the asterisks indicated the protein bands analyzed by mass spectrometry. However, there was no mass spec. data provided. Are both bands identified as Drp1? The two bands showed different Mw sizes, suggesting they are either different proteins or post-translationally modified differently. However, according to Fig. 6, both full length RidL and the Δbeta loop mutant increase Drp1 phosphorylation. Please clarify.

b. Have the authors compared the affinities of full length RidL, RidL-N, RidL-C, and RidL-Δbeta toward Drp1 in the presence or absence of Vps29? This would provide information if the Vps29 interaction competes or interferes with the Drp1 binding affinity.

The authors may also consider using the RidLΔbeta variant in the GTPase activity experiment, which may inform whether a stronger RidL interaction leads to more inhibition of the GTPase activity.

3. Fig. 2:

a. Fig. 2A: Although SidC was used as a negative control, have the authors tried RidL-N, RidL-C, and RidL-Δbeta in the EM experiments? Results from these variants would establish a correlation of the Drp1 affinity with formation of the Drp1 oligomers.

b. Fig. 2B: I am not an expert in dynamic light scattering; however, the results in Fig. 2B were difficult to understand.

- Adding RidL or SidC caused shifts of the peak to 51 nm or 59 nm. However, the data did not help with the authors' claim that RidL disrupts Drp1 oligomerization. Although the authors speculated that SidC caused bigger Drp1 oligomers and RidL form complexes with Drp1, no direct evidence was shown.

4. Fig. 3:

a. Fig. 3A, Line 170-172:
- Yeast colonies were formed by cells co-expressing RidL with Dnm2, Dnm3 or DymB on -His plates. The negative controls (no RidL) were clearly no colony formation. Therefore, stating no interactions with these proteins was not appropriate. While the size and number colonies might correlate with the binding affinity, the fusions might interfere with the interactions. The authors should cautiously interpret this result.
- Given that Dnm2, Dnm3, and DymB still formed colonies, the authors should consider a more distally related GTPase as a negative control, such as DlpA.
- Expression of the fusion proteins in Y2H should be evaluated.

b. Fig. 3B: Expression of the GFP fusion proteins should be determined.

5. Fig. 4:

a. Expression of RidL in the Lp strains should be determined.

b. Fig. S5: The GFP images for Tom20 and Lp fluorescence images were very dim, making them difficult to see.

c. Fig. 4G, 4H: Did the ΔridL_ridL strain significantly restore co-localization of mitochondria with Drp1 or Tom20?

6. Fig. 6:

a. Fig.6A, S7: The Fluor-488 image of ΔicmT showed strong signals for phosphor-Ser616 Drp, even stronger than ΔridL-ridL and ΔridL_ridLΔbeta-loop. These observations contradicted Fig. 6C. The author should use a different method to measure phosphorylation of Ser616 Drp1 (ex: immunoblotting since the phospho-antibody is available and applicable based on the vendor's website).

b. Fig. 6C: This result indicates that binding to Vps29 might interfere with phosphorylation of Drp1. This further encourages the authors to determine if the interactions with Drp1 and Vps29 can occur simultaneously and whether loss of Vps29 binding increases Drp1 interaction (and/or phosphorylation).

7. Line 354-358: The authors mentioned RidL also binds to PI3Ps. This was good to see. In this study, there was no direct evidence showing that full-length RidL interacts with Drp1 in host cells. The only straightforward experiment was the in vitro pulldown assay when no other host proteins or factors were present. The rest of the results were about the effects of RidL on Drp1 or mitochondria. Taken together, it is still unclear if full-length RidL interacts with Drp1 in host cells infected with Lp. Note: Y2H is not in the host cells.

Minor comments:

1. Fig. 1E: I assumed the bands slightly above 70 kD were recombinant Drp1. Please mark it in the figure.
2. Line 125-127 and Fig. 1H: The authors described "...the activity was reduced less than two-fold..." and "reduced the GTPase activity of Drp1A395D by approximately two-fold". Could the authors convert the estimated fold change to % of no RidL control? Ex: in the presence of 20x excess RidL, the GTPase activity of wildtype Drp1 is X % of Drp1 without RidL.
3. Fig. 6B, Line 276-277: ΔridL_ridL did not seem to restore co-localization of phosphor-Drp1 with mitochondria.
4. Line 317: not "hrough". Do you mean "through"?

Significance:

The strength of this manuscript includes biochemical assays and advanced microscopy. The quantitative analyses of microscopy were rigorous. Identifying a new host target of a bacterial effector is always exciting in the field of host-pathogen interactions. The results in this manuscript suggest that Drp1 might be a new target of Legionella RidL.

Although the in vitro assays and cell lysate pulldown showed the interaction between RidL and Drp1, the inconsistency between the full-length RidL and variants raises a question whether the RidL-Drp1 interaction is true in infected cells. Additionally, the complementation experiments using ΔridL mutant Legionella showed the effect of RidL on mitochondria fragmentation and Drp1 localization, but there was no direct evidence provided to demonstrate the phenomenon is caused by the RidL-Drp1 interaction. In summary, the main questions are: 1. whether the RidL-Drp1 interaction can occur in Lp-infected cells, particularly in the presence of Vps29, 2. direct evidence to demonstrate this interaction causes mitochondria fragmentation and Drp1 phosphorylation, and 3. additional experiments are needed to confirm that RidL promote Drp1 phosphorylation and this

phosphorylation contributes to mitochondria fragmentation.

Readers in basic research of bacterial pathogenesis and mitochondria biology would be interested.

Referee #3:

This study reports a new function for the Legionella pneumophila effector RidL, expanding its role beyond retromer inhibition to direct modulation of mitochondrial dynamics. The authors suggest that the C-terminal domain of RidL binds to the mitochondrial fission GTPase Drp1, impairs its GTPase activity and oligomerization in vitro, and promotes phosphorylation of Drp1 at Ser616 during infection, leading to mitochondrial fragmentation and loss of mitochondrial function.

Several aspects could be strengthened to clarify the mechanism and physiological relevance.

Specific queries:

[1] Amount of RidLC that binds to purified Drp1 is only a fraction of that of WT RidL (Figure 1F). If the C-terminal alone was facilitating the interaction with Drp1, significantly more RidLC should be observed. Authors should repeat and compare unbound vs bound fractions to determine the proportion of RidLc that is actually interacting with Drp1.

[2] Can appreciate the pull down using recombinant proteins but this approach really limits biological interpretations. Given the tools available the authors should express RidL (exogenously) in cells and assess protein interactions using the available RiDL antibodies via IP. Given the authors also see such good expression of RidL from Legionella and association with mitochondria they can also assess this in the infection context.

[3] The authors demonstrate that RidL promotes mitochondrial fragmentation in vivo by showing that cells infected with WT and ΔridL_RidL L. pneumophila have lower mitochondrial aspect ratios and reduced oxygen consumption rates compared to uninfected or ΔridL-infected cells (Figure 5). This contradicts earlier findings that RidL prevents Drp1 oligomerisation and tubulation in vitro (Fig 2), which is required for mitochondrial fission.

[4] Figure 4B should be probed with a legionella antibody to ensure this signal is not coming from bacteria. The amount of Legionella secreted RidL looks excellent by western blot, complement this via IFA to ensure specificity.

[5] Figure 4E and F need to be quantified or shown with western blot. The western would be more convincing.

[6] Mechanistic link between RidL and Drp1 phosphorylation is incomplete. While RidL expression increases Ser616 phosphorylation of Drp1, it remains unclear how this occurs. The co-immunoprecipitation experiments suggested above might help to identify the host signalling components responsible for this modification.

[7] Physiological relevance of the in vitro Drp1 inhibition is uncertain. The inhibition of Drp1 GTPase activity by RidL requires a 20-fold molar excess and results in less than a twofold reduction in activity (Figure 1H-I). This weak inhibition raises doubts about its significance in cells. Authors need to comment on this and provide evidence that the effect is specific rather than due to protein crowding or aggregation.

[8] Localization data need stronger validation. Evidence for mitochondrial localisation of RidL is strong but would be good to show it is on the outer membrane to support an interaction with Drp1. Additional controls, such as protease protection assays or carbonate extraction, could clarify whether RidL is peripherally associated with the outer membrane or internalised.

Minor points:

[9] Why is there a considerable difference in GTPase activity of the same conditions (0 µM and 20 µM RidL) between Fig 1G and Fig 1H, especially since the small error bars suggest that there is otherwise minor variability between independent replicates?

[10] On graphs that depict the mean of replicate experiments, display the individual data points (e.g. Fig 1G, Fig 1H, Fig 4A, Fig 6).

[11] Legend details of Fig 6C do not match those in the method. Can the authors clarify if (and if so, why) the cells were treated with MTDR for flow cytometry?

[12] Line 207-213. Keep nomenclature consistent with previous text & figures, i.e. RidLN and not RidL9-258.

[13] Line 317. Fix spelling, 'hrough'.

[14] Consider using colourblind-friendly palettes for immunofluorescence microscopy images.

[15] Increase the size of microscopy scale bar text or state the scale bar size in the figure legends.

Significance:

This is an interesting study that adds to the growing understanding of how L. pneumophila effectors target mitochondria. This is a growing and significant area of interest in the host pathogen space. The data is clear but there are some contradictions that we have raised in our assessment. Undertaking the suggested experiments will strengthen the paper and overall conclusions.

General statements

In our study "The *Legionella* effector RidL binds the large fission GTPase Drp1 to promote mitochondrial fragmentation" we identify the mitochondrial large fission GTPase Drp1 and closely related GTPases as novel interactors of the *L. pneumophila* effector protein RidL. Drp1 is bound by a C-terminal fragment of the effector, the function of which was previously unknown. Moreover, we document that RidL promotes mitochondrial fission, reduction of mitochondrial membrane potential, and phosphorylation activation of Drp1. Our study reveals the first bacterial effector protein targeting eukaryotic large fission GTPases.

We would like to thank the three reviewers for their thorough, constructive and overall positive evaluation of our work. We appreciate the general assessment of the reviewers stating that "This study convincingly demonstrates an interaction between RidL and Drp1. Additionally, it shows a role for RidL in mitochondrial fragmentation, reduced mitochondrial membrane potential and oxygen consumption during *Legionella* infection, and is of interest to a specialized audience working on intracellular microorganisms." (**reviewer #1**), "Readers in basic research of bacterial pathogenesis and mitochondria biology would be interested" (**reviewer #2**), and "This is an interesting study that adds to the growing understanding of how *L. pneumophila* effectors target mitochondria. This is a growing and significant area of interest in the host-pathogen space." (**reviewer #3**).

To further strengthen our manuscript, we performed the following experiments:

1. Assess binding of Drp1 by RidL in infected cells. We performed co-IPs using anti-RidL antibody, protein A/G beads and lysates of HeLa cells ectopically producing RidL (new **Fig. 1D**). We also performed co-IPs using anti-RidL antibody, protein A/G beads and lysates of HeLa cells infected with *L. pneumophila* JR32 wild-type, Δ*icmT*, Δ*ridL*, and complemented Δ*ridL* mutant strains. However, this approach did not yield eukaryotic interaction partners of RidL.
2. Determine the role of the retromer for binding of Drp1 to RidL. We assessed binding of RidL and $RidL_{\Delta\beta}$ to Drp1 in absence or presence of purified Vps29 (new **Fig. 1H** and **EV1D**).
3. Control production of GFP-fusion proteins in the yeast bimolecular fluorescence complementation experiment. Using antibodies against GFP or specific large fission GTPases, we assessed the production of GFP_{1-10} fusion proteins and select large GTPases in yeast (new **Fig. 3D** and **3E**).

4. Localization of RidL to the mitochondrial outer membrane. We performed proteinase K protection assays on mitochondria isolated from HeLa cells ectopically producing RidL or N- or C-terminal fragments of RidL (new **Fig. 4D**).
5. Control production of RidL and mutant proteins in *L. pneumophila* strains. We assessed the production of RidL by Western blot in equal numbers of stationary phase *L. pneumophila* JR32, Δ*icmT*, Δ*ridL*, Δ*ridL*/pRidL, or Δ*ridL*/pRidL$_{\Delta\beta}$ (new **Fig. 6D**).

The line numbers in the below point-by-point response correspond to the "changes highlighted" version of the manuscript.

Point-by-point response to the reviewers' comments

Reviewer #1 (Evidence, reproducibility and clarity (Required)):

In this paper, the authors identify Drp1 as an interaction partner of the *L. pneumophila* type 4 secretion system effector RidL both in vitro with co-immunoprecipitation and in vivo with yeast two hybrid assays. With in vitro assays they show RidL interferes with Drp1 GTPase activity and oligomerization. During *Legionella* infections, they show RidL localizes to mitochondria. Consistent with previous studies, *Legionella* infection leads to mitochondrial fission and reduced mitochondrial membrane potential and oxygen consumption, phenotypes reduced during infection with a *ridL*-deficient strain of *Legionella*. Additionally, the authors show that Tom20, Drp1 and Drp1(p-616) accumulate on MitoTracker stained mitochondria during *Legionella* infected cells in a partially RidL dependent process.

Response: We would like to thank the reviewer for this thorough and positive evaluation of our work.

- 1. Line 94: "The experimental results closely match the Alphafold2 structure prediction (Fig. 1A)..." How is closely matching defined? The authors should include a structural alignment between the alphafold structure and experimental structures and report a RMSD value or a figure of the structural alignment.

Response: As suggested, we included a structural alignment (new **Fig. EV1A**), and we report a RMSD value of 0.445 Å for the 213 N-terminal amino acids for the overlay (**l. 110**).

- 2. When describing the dynamic light scattering data in Fig. 2B the text is not clear about how the data is interpreted as supporting a role of RidL in inhibiting Drp1 oligomerization. Additionally, the conclusion that the peak of the distribution increasing from 44 nm in the Drp1 only sample to 51 nm in the Drp1+RidL sample suggests the presence of a Drp1-RidL complex isn't supported when the Drp1+SidC sample has a further shifted peak of 59 nm despite there being no known interaction between Drp1 and SidC.

Response: We sought to clarify this point by adding explanatory sentences in the Results section (**l. 192-194, 201-202**) and the Discussion section (**l. 423-428**). The addition of RidL, but not SidC, affects the symmetrical distribution of complexes resulting in a broad and rather irregular distribution of hydrodynamic radii. Importantly, in presence of Drp1, SidC (12 nm) but not RidL (16 nm) shows a symmetrical peak at the hydrodynamic radius of the effector alone, indicating that unbound SidC but not RidL is present in the sample. Moreover, we tone down the statement about the symmetrical peak observed a 59 nm: "…, the symmetrical peak around 59 nm might correspond to oligomerized Drp1, which seems to form even larger aggregates than Drp1 alone in absence of SidC (44 nm, **Fig EV2C**)". See also **reviewer #2, point 3**.

- 3. Line 276: The authors state: "Co-localization of Drp1 with MitoTracker was restored by supplying plasmid-produced RidL or RidLΔβ-loop." However, figure 6B shows complementation with wild type RidL has no impact on p-Drp1-mitochondrial co-localization, while the RidLΔβ-loop complementation only partially restores the phenotype. Clarify this in the text.

Response: Thank you for pointing out this inaccuracy. In the immuno-fluorescence microscopy approach (**Fig. 6AB**), the phenotype of the Δ*ridL* mutant strain was indeed not complemented. However, in the FACS approach (**Fig. 6C**), the phenotype was complemented by $\text{RidL}_{\Delta\beta}$ and – less efficiently – by wild-type RidL. We corrected the text accordingly (**l. 340-341, l. 348-349**). As a control, we assessed the production of RidL by different *L. pneumophila* strains and found that the complemented Δ*ridL* mutant strains produced the effector protein at even higher levels than the wild-type strain JR32 (**Fig 6D**, **l. 349-351**). See also **reviewer #2**, minor points.

- 4. Quantification of microscopy data in Figures 3C, 4GH, and 6B are not adequately described. There is not enough information in either the figure legends or methods section to know how the quantification was performed.

Response: We outlined the quantification of the microscopy data in more detail in the Methods section (**l. 729-733, l. 876-885**). Moreover, we changed the Y-axes of Fig. 4GH, which now reads "% of Drp1/Tom20 colocalizing with mitotracker".

- 5. Line 327: "Our study provides novel links between the retromer and Drp1..." I'm not sure what data in the paper provides any link between retromer and Drp1. In separate papers, the authors have shown that RidL interacts with Drp1 and the retromer, however, they have not performed experiments to determine how/if RidL interacts with both concurrently or if they are separate events.

Response: To address this point, we assessed binding of RidL and $RidL_{\Delta\beta}$ to Drp1 in absence or presence of purified Vps29 (new **Fig. 1H**). However, while $RidL_{\Delta\beta}$ bound Drp1 more strongly than RidL, Vps29 did not significantly interfere with the interaction. Accordingly, we tone down the statement, which now reads "Our study addresses the complex interplay between …" (**l. 397-398**). See also below, **reviewer #2, points 2b and 6b**.

- 6. The description of statistics states that two-tailed T tests were used. There should also be additional multiple hypotheses correction performed for the analysis of data within each experiment (or ANOVA with posthoc testing).

Response: We performed and documented ANOVA statistics throughout the manuscript.

Reviewer #1 (Significance (Required)):

This study reveals a role for the *Legionella* type 4 secretion system effector RidL in interfering with mitochondrial function. It has previously been shown that *Legionella* infection disrupts mitochondrial function, and that other translocated effectors were involved, but this is the first time RidL has been implicated in this function. The authors have also previously demonstrated that RidL inhibits the retromer complex during *Legionella* infection but introduce this as a new role.

This study convincingly demonstrates an interaction between RidL and Drp1. Additionally, it shows a role for RidL in mitochondrial fragmentation, reduced mitochondrial membrane potential and oxygen consumption during *Legionella* infection, and is of interest to a specialized audience working on intracellular microorganisms. A weakness of the manuscript is that except for the increase in Drp1 phosphorylation at Ser 616, it is unclear whether the interaction of RidL with Drp1 or the retromer is important for the observed phenotypes. The paper would be strengthened by addressing this concern and including complementation of ΔridL mutants with RidL-Nterm (only interacts with retromer) and RidLΔβ-loop. For example, one interpretation of the results in Fig 4e-h would be that *Legionella* is blocking mitochondrial derived vesicle formation (Tom20 is a mitochondrial outer membrane marker and has been used to study MDVs) leading to the accumulation of Tom20 and Drp1 on MitoTracker staining mitochondria. However, the authors state that both the retromer and Drp1 are involved in the formation of MDVs.

Response: We would like to thank the reviewer for the constructive and positive evaluation of our work. Using FACS, we were indeed able to complement a phenotype of the *L. pneumophila* Δ*ridL* mutant strain (less pDrp1 co-localizing with mitochondria) with the $RidL_{\Delta\beta}$ construct more efficiently than with the RidL construct (**Fig. 6C**). This result suggests that the RidL-dependent co-localization of pDrp1 with mitochondria does not require an interaction of RidL with the retromer. The Δ*ridL* mutant can unfortunately not be complemented by a plasmid producing the RidL-N fragment, since this fragment is lacking the C-terminal type IV secretion signal, and therefore, will not be translocated by the bacteria. In agreement with the notion that the retromer is not implicated in localizing RidL to mitochondria, RidLC but not RidLN was found to localize to mitochondria (**Fig. 4CD**). Finally, as suggested by the reviewer, we discuss the data shown in **Fig. 4E-H** and in **Fig. 6A-C** in the context of mitochondrial dynamics, MDV formation, and localization of pDrp1, as well as the role of Drp1 and the retromer in these processes (**l. 383-403**).

Reviewer Expertise: *Legionella* infection biology.

Reviewer #2 (Evidence, reproducibility and clarity (Required)):

Summary:

In the manuscript by Katic et al., the authors reported that the *Legionella* effector RidL promotes mitochondria fragmentation in host cells and identified a potential interacting protein Drp1 by microscopy and biochemical assays. In addition, they found that RidL also promotes phosphorylation of Drp1. These discoveries indicate a new activity of the bacterial effector to manipulate host mitochondria. My specific comments are below:

Major comments:

1. Suggest changing the title: The results did not directly demonstrate phosphorylation of Drp1 caused by RidL interaction promotes mitochondrial fragmentation. The presenting results show RidL interacts with Drp1 and RidL increases Drp1 phosphorylation. If the authors want to make the claim of "through phosphorylation activation of Drp1", they should use a phospho-resistant Drp1 mutant in the infection experiment with wildtype Lp.

Response: We would like to thank the reviewer for pointing out this inaccuracy, and we changed the title as suggested: "The *Legionella* effector RidL binds the large fission GTPase Drp1 to promote mitochondrial fragmentation".

2. Fig. 1:

a. Fig. 1B-F: The pulldown assays using recombinant proteins and HeLa cell lysate produced different results. It seems that full-length RidL interacts when using only recombinant proteins (in vitro pulldown) but the interaction was much more reduced in the presence of other cellular proteins (HeLa lysate). This raises a question whether this RidL/Drp1 interaction actually occurs within host cells. Although the Y2H experiment showed that RidL interacts with Drp1, the result still does not directly support the interaction in the natural host amoeba or mammalian cells. The authors should perform the pulldown assays in Lp-infected cells. Vps29 can be included as positive and negative controls for the experiments in 1B to 1F.

Response: Thank you for this valid suggestion. We performed co-IPs using anti-RidL antibody, protein A/G beads and lysates of HeLa cells ectopically producing RidL (new **Fig. 1D**). These experiments revealed that ectopically produced RidL binds Drp1 but not the closely related

large fission GTPase Dnm1. We also performed co-IPs using anti-RidL antibody, protein A/G beads and lysates of HeLa cells infected with *L. pneumophila* JR32 wild-type, Δ*icmT*, Δ*ridL*, and complemented Δ*ridL* mutant strains. However, this approach did not yield eukaryotic interaction partners of RidL – likely because the amount of RidL delivered to the host cells was too little. Moreover, we also assessed binding of RidL and RidL$_{\Delta\beta}$ to Drp1 in absence or presence of purified Vps29 (new **Fig. 1H** and **EV1D**). See also below, **reviewer #2, point 6 and 7**, and **reviewer #3, point 2**.

According to the figure legend, the asterisks indicated the protein bands analyzed by mass spectrometry. However, there was no mass spec. data provided. Are both bands identified as Drp1? The two bands showed different Mw sizes, suggesting they are either different proteins or post-translationally modified differently. However, according to Fig. 6, both full length RidL and the Δbeta loop mutant increase Drp1 phosphorylation. Please clarify.

Response: As suggested, we included the MS data (new **Fig. S1B**) for **Fig. 1B**. Moreover, in order to unequivocally identify the different proteins retained by RidL (Vps35) or RidL$_{\Delta\beta}$ (Drp1), we labelled the two protein bands with different symbols (**Fig. 1B**), which are defined in the figure legend: "… analyzed by (**B**) silver stain and mass spectrometry: protein bands labelled by "<" (Vps35) or "*" (Drp1)". Finally, we outline in the Discussion section (**l. l. 417-420**) the observation that RidL and – even more efficiently – RidL$_{\Delta\beta}$ complement the Δ*ridL* mutant phenotype (**Fig. 6C**).

b. Have the authors compared the affinities of full length RidL, RidL-N, RidL-C, and RidL-Δbeta toward Drp1 in the presence or absence of Vps29? This would provide information if the Vps29 interaction competes or interferes with the Drp1 binding affinity. The authors may also consider using the RidLΔbeta variant in the GTPase activity experiment, which may inform whether a stronger RidL interaction leads to more inhibition of the GTPase activity.

Response: Thank you for this suggestion. We now assess binding of RidL and RidL$_{\Delta\beta}$ to Drp1 in absence or presence of purified Vps29 (new **Fig. 1H**). Under the conditions used, Vps29 slightly but non-significantly reduced the binding of RidL to Drp1, while RidL$_{\Delta\beta}$ bound Drp1

more strongly than RidL in absence as well as in presence of up to four times excess Vps29 (**Fig. 1H and EV1D**). Accordingly, while $RidL_{\Delta\beta}$ binds Drp1 more strongly than RidL, Vps29 does not significantly interfere with the interaction (**l. 144-151**). See also below, **point 6b**.

The effect of RidL on Drp1 implicates an unidentified kinase (**Fig. 6**). In the *in vitro* GTPase assays shown in **Fig. 1IJ and EV1D**, the unidentified Drp1 kinase is missing. Accordingly, we believe that adding $RidL_{\Delta\beta}$ to Drp1 in these GTPase assays might not reflect the in vivo conditions.

3. Fig. 2:

a. Fig. 2A: Although SidC was used as a negative control, have the authors tried RidL-N, RidL-C, and RidL-Δbeta in the EM experiments? Results from these variants would establish a correlation of the Drp1 affinity with formation of the Drp1 oligomers.

Response: The effect of RidL on Drp1 implicates an unidentified kinase (**Fig. 6**). In the in vitro filamentation assays shown in **Fig. 2A** the unidentified Drp1 kinase is missing. Accordingly, we believe that adding RidL fragments or $RidL_{\Delta\beta}$ to Drp1 in the filamentation assays might not reflect the in vivo conditions.

b. Fig. 2B: I am not an expert in dynamic light scattering; however, the results in Fig. 2B were difficult to understand. Adding RidL or SidC caused shifts of the peak to 51 nm or 59 nm. However, the data did not help with the authors' claim that RidL disrupts Drp1 oligomerization. Although the authors speculated that SidC caused bigger Drp1 oligomers and RidL form complexes with Drp1, no direct evidence was shown.

Response: We sought to clarify this point by adding explanatory sentences in the Results section (**l. 192-194, 201-202**) and the Discussion section (**l. 423-428**). The addition of RidL, but not SidC, affects the symmetrical distribution of complexes resulting in a broad and rather irregular distribution of hydrodynamic radii. Importantly, in presence of Drp1, SidC (12 nm) but not RidL (16 nm) shows a symmetrical peak at the hydrodynamic radius of the effector alone, indicating that unbound SidC but not RidL is present in the sample. Moreover, we tone down the statement about the symmetrical peak observed a 59 nm: "…, the symmetrical peak around 59 nm might correspond to oligomerized Drp1, which seems to form even larger

aggregates than Drp1 alone in absence of SidC (44 nm, **Fig EV2C**)". See also **reviewer #1, point 2**.

4. Fig. 3:

a. Fig. 3A, Line 170-172:

- Yeast colonies were formed by cells co-expressing RidL with Dnm2, Dnm3 or DymB on -His plates. The negative controls (no RidL) were clearly no colony formation. Therefore, stating no interactions with these proteins was not appropriate. While the size and number colonies might correlate with the binding affinity, the fusions might interfere with the interactions. The authors should cautiously interpret this result.
- Given that Dnm2, Dnm3, and DymB still formed colonies, the authors should consider a more distally related GTPase as a negative control, such as DlpA.
- Expression of the fusion proteins in Y2H should be evaluated.

Response: We agree with the reviewer and tone down the statements regarding the interactions of RidL with different large fission GTPases (**l. 213-214**). YTH screens are semiquantitative, false positives are frequently a problem (hence the addition of AT), and mutants might arise. Therefore, some background colonies grow even under conditions where there no or little bait-prey interactions occur.

We performed the YTH screens using RidL as bait and large GTPases as prey to possibly identify large GTPases that bind RidL with higher affinity than Drp1 (**Fig. 3A**). Among the mammalian large GTPases Drp1 seemed to bind strongest to RidL, while the *Dictyostelium* large GTPase DymA (but not DymB) and the yeast GTPase Vps1 also strongly bound RidL. Given this aim and results, we believe it is more straightforward to focus on the validation and functional characterization of positive hits (Drp1) (**Fig. 4-6**) than testing another, more distantly related large fission GTPase. The production of the large fission GTPases in yeast was assessed by Western blot (new **Fig 3E**).

b. Fig. 3B: Expression of the GFP fusion proteins should be determined.

Response: As suggested, we evaluated the production of GFP-fusion proteins in the yeast bimolecular fluorescence complementation experiments. Using antibodies against GFP or specific large fission GTPases, we assessed the production of GFP_{1-10} fusion proteins (new **Fig.**

3D) and select large GTPases (new **Fig. 3E**). The Western blots indicated that all large GTPases are produced in yeast.

5. Fig. 4:

a. Expression of RidL in the Lp strains should be determined.

Response: As suggested, we assessed the production of RidL by Western blot in equal numbers of stationary phase *L. pneumophila* JR32, Δ*icmT*, Δ*ridL*, Δ*ridL*/pRidL, or Δ*ridL*/pRidL$_{\Delta\beta}$ (new **Fig. 6D**). The complemented Δ*ridL* mutant strains produced the effector protein at even higher levels than the wild-type strain JR32.

b. Fig. S5: The GFP images for Tom20 and Lp fluorescence images were very dim, making them difficult to see.

Response: We enhanced the brightness of the images (**Fig. S1 and S2**), and we now use fluorescence colors (cyan, magenta, yellow) more easily recognized by color-blind people (see also **reviewer #3, point 14**).

c. Fig. 4G, 4H: Did the ΔridL_ridL strain significantly restore co-localization of mitochondria with Drp1 or Tom20?

Response: The complemented Δ*ridL* mutant, Δ*ridL*/pRidL restored co-localization of mitochondria with Tom20 but not with Drp1 in a statistically significant manner (**Fig. 4GH**). A (statistically not significant) tendency was also observed toward restoring the co-localization of mitochondria with Drp1 (**Fig. 4G**). These statistics are now indicated.

6. Fig. 6:

a. Fig.6A, S7: The Fluor-488 image of Δ*icmT* showed strong signals for phospho-Ser616 Drp, even stronger than Δ*ridL-ridL* and Δ*ridL*_ridLΔbeta-loop. These observations contradicted Fig. 6C. The author should use a different method to measure phosphorylation of Ser616 Drp1 (ex: immunoblotting since the phospho-antibody is available and applicable based on the vendor's website).

Response: We repeated the experiments shown in **Fig. 6** and now demonstrate that upon infection with *L. pneumophila* wild-type, approximately 50% of the detected phospho-Drp1

localized with the mitochondrial stain MitoTracker, while this was the case for ca. 20% or 30% of the detected phospho-Drp1 upon infection with *L. pneumophila* Δ*icmT* or Δ*ridL*, respectively. Plasmid-produced RidL or $RidL_{\Delta\beta}$ did not restore the colocalization of phospho-Drp1 and MitoTracker in a statistically significant manner, but a tendency was seen for $RidL_{\Delta\beta}$ (**Fig. 6B**). Moreover, we unsuccessfully tried to quantify pDrp1 by Western blot. While we followed the manufacturer's instruction, not even the positive control provided by the company worked. Finally, we enhanced the brightness of the images (**Fig. S1 and S2**), and we now use fluorescence colors (cyan, magenta, yellow) more easily recognized by color-blind people (see **reviewer #3, point 14**).

b. Fig. 6C: This result indicates that binding to Vps29 might interfere with phosphorylation of Drp1. This further encourages the authors to determine if the interactions with Drp1 and Vps29 can occur simultaneously and whether loss of Vps29 binding increases Drp1 interaction (and/or phosphorylation).

Response: To address this point, we assessed binding of RidL and $RidL_{\Delta\beta}$ to Drp1 in absence or presence of purified Vps29 (new **Fig. 1H**). However, while $RidL_{\Delta\beta}$ bound Drp1 more strongly than RidL, Vps29 did not significantly interfere with the interaction. See also above, response to **point 2b** and **reviewer #1, point 5**.

7. Line 354-358: The authors mentioned RidL also binds to PI3Ps. This was good to see. In this study, there was no direct evidence showing that full-length RidL interacts with Drp1 in host cells. The only straightforward experiment was the in vitro pulldown assay when no other host proteins or factors were present. The rest of the results were about the effects of RidL on Drp1 or mitochondria. Taken together, it is still unclear if full-length RidL interacts with Drp1 in host cells infected with Lp. Note: Y2H is not in the host cells.

Response: To assess whether RidL binds Drp1 in mammalian host cells, we performed co-IPs using anti-RidL antibody, protein A/G beads and lysates of HeLa cells ectopically producing RidL (new **Fig. 1D**). These experiments revealed that ectopically produced RidL binds Drp1 but not the closely related large fission GTPase Dnm1 (see also above, **point 2a**, and **reviewer #3, point 2**).

Minor comments:

1. Fig. 1E: I assumed the bands slightly above 70 kD were recombinant Drp1. Please mark it in the figure.

Response: As suggested, we marked the band representing recombinant Drp1 in **Fig. 1F**, and we state in the legend to the figure: "… visualized by (**F**) silver stain ("*" (RidL), "<" (Drp1)".

2. Line 125-127 and Fig. 1H: The authors described "...the activity was reduced less than two-fold..." and "reduced the GTPase activity of Drp1A395D by approximately two-fold". Could the authors convert the estimated fold change to % of no RidL control? Ex: in the presence of 20x excess RidL, the GTPase activity of wildtype Drp1 is X % of Drp1 without RidL.

Response: We now quantify in percentage of control (**l. 159-162**).

3. Fig. 6B, Line 276-277: ∆ridL_ridL did not seem to restore co-localization of phosphor-Drp1 with mitochondria.

Response: Thank you for pointing out this inaccuracy. In the immuno-fluorescence microscopy approach (**Fig. 6AB**), the phenotype of the Δ*ridL* mutant strain was indeed not complemented. However, in the FACS approach (**Fig. 6C**), the phenotype was complemented by $\text{RidL}_{\Delta\beta}$ and – less efficiently – by wild-type RidL. We corrected the text accordingly (**l. 340-341, l. 348-349**). See also **reviewer #1, point 3**.

4. Line 317: not "hrough". Do you mean "through"?

Response: We corrected the typo (**l 386**).

Reviewer #2 (Significance (Required)):

The strength of this manuscript includes biochemical assays and advanced microscopy. The quantitative analyses of microscopy were rigorous. Identifying a new host target of a bacterial effector is always exciting in the field of host-pathogen interactions. The results in this manuscript suggest that Drp1 might be a new target of *Legionella* RidL.

Although the in vitro assays and cell lysate pulldown showed the interaction between RidL and Drp1, the inconsistency between the full-length RidL and variants raises a

question whether the RidL-Drp1 interaction is true in infected cells. Additionally, the complementation experiments using ∆ridL mutant *Legionella* showed the effect of RidL on mitochondria fragmentation and Drp1 localization, but there was no direct evidence provided to demonstrate the phenomenon is caused by the RidL-Drp1 interaction. In summary, the main questions are: 1. whether the RidL-Drp1 interaction can occur in Lp-infected cells, particularly in the presence of Vps29, 2. direct evidence to demonstrate this interaction causes mitochondria fragmentation and Drp1 phosphorylation, and 3. additional experiments are needed to confirm that RidL promote Drp1 phosphorylation and this phosphorylation contributes to mitochondria fragmentation.

Readers in basic research of bacterial pathogenesis and mitochondria biology would be interested.

Response: Thank you for this thorough and overall positive evaluation. We sought to answer the main questions by addressing the above specific points. We agree that the further analysis of Drp1 phosphorylation by RidL and its effects on mitochondrial fragmentation are very important and intriguing aspects of *L. pneumophila* infection. We believe that the further analysis of the kinase catalyzing RidL-dependent Drp1 phosphorylation is beyond the scope of the current study.

My expertise: host-pathogen interactions. post-translational modification. bacterial virulence.

Reviewer #3 (Evidence, reproducibility and clarity (Required)):

This study reports a new function for the *Legionella pneumophila* effector RidL, expanding its role beyond retromer inhibition to direct modulation of mitochondrial dynamics. The authors suggest that the C-terminal domain of RidL binds to the mitochondrial fission GTPase Drp1, impairs its GTPase activity and oligomerization in vitro, and promotes phosphorylation of Drp1 at Ser616 during infection, leading to mitochondrial fragmentation and loss of mitochondrial function.

Several aspects could be strengthened to clarify the mechanism and physiological relevance.

Specific queries:

[1] Amount of RidL_C that binds to purified Drp1 is only a fraction of that of WT RidL (Figure 1F). If the C-terminal alone was facilitating the interaction with Drp1, significantly more RidL_C should be observed. Authors should repeat and compare unbound vs bound fractions to determine the proportion of RidL_C that is actually interacting with Drp1.

Response: Thank you for raising this point. We agree that compared to full length RidL only a fraction of RidL_C binds to Drp1 (**Fig. 1F**). To estimate the amount of bound vs. unbound RidL and fragments thereof, equal amounts of the proteins were used for the binding assays, as shown in **Fig. 1E**. Even upon over-exposure of the Western blot, RidL_N does not bind at all to Drp1 (**Fig. S1C**), while the input amount of RidL, RidL_N, and RidL_C is the same (**Fig. 1E**). We now state this in the text (**l. 141-142**).

[2] Can appreciate the pull down using recombinant proteins but this approach really limits biological interpretations. Given the tools available the authors should express RidL (exogenously) in cells and assess protein interactions using the available RidL antibodies via IP. Given the authors also see such good expression of RidL from *Legionella* and association with mitochondria they can also assess this in the infection context.

Response: Thank you for raising this important point. As suggested, we performed co-IPs using anti-RidL antibody, protein A/G beads and lysates of HeLa cells ectopically producing RidL to assess whether RidL binds Drp1 in mammalian host cells (new **Fig. 1D**). These experiments revealed that ectopically produced RidL binds Drp1 but not the closely related large fission GTPase Dnm1. See also above, **reviewer #2**, **point 2 and 7**.

[3] The authors demonstrate that RidL promotes mitochondrial fragmentation in vivo by showing that cells infected with WT and ΔridL_ridL *L. pneumophila* have lower mitochondrial aspect ratios and reduced oxygen consumption rates compared to uninfected or ΔridL-infected cells (Figure 5). This contradicts earlier findings that RidL prevents Drp1 oligomerisation and tubulation in vitro (Fig 2), which is required for mitochondrial fission.

Response: Thank you for raising this point. We show that RidL promotes the phosphorylation (and presumably activation) of Drp1 (**Fig. 6**) through an (unidentified) kinase. In the biochemical reconstitution and oligomerization experiments (**Figs. 1**, **2**), the unidentified host kinase activating Drp1 is missing, providing a possible explanation for the apparent discrepancy between the in vitro and the in vivo experiments. We have now further outlined this point in the Discussion section (**l. 421-429**). See also below, **point 7**.

[4] Figure 4B should be probed with a *Legionella* antibody to ensure this signal is not coming from bacteria. The amount of *Legionella* secreted RidL looks excellent by western blot, complement this via IFA to ensure specificity.

Response: We use the *L. pneumophila* Δ*icmT* mutant strain – lacking any Icm/Dot-dependent effector protein translocation – as a control for bacterial proteins. Since there is no signal detected (in the cytoplasm or mitochondrial fraction) of cells infected with Δ*icmT* mutant bacteria (**Fig. 4B**), the RidL signal cannot come from (lysed) bacteria. Thus, the detection of RidL by Western blot in the cytoplasm or in the mitochondrial fraction of host cells requires a functional T4SS and obviously the presence of RidL, as evidenced by the Δ*ridL* mutant strain (**Fig. 4B**).

[5] Figure 4E and F need to be quantified or shown with western blot. The western would be more convincing.

Response: The immuno-fluorescence microscopy data shown in **Fig. 4EF** is indeed quantified in **Fig. GH**, revealing that in *L. pneumophila*-infected HeLa cells, RidL and a functional Icm/Dot T4SS increase the ratios of mitochondrial Drp1 and Tom20. As suggested by the reviewer, we also sought to quantify Drp1 and Tom20 by Western blot on mitochondria isolated from *L. pneumophila*-infected cells (**Fig. R1**). Upon isolation of mitochondria, Drp1 was found to mainly localize to the cytoplasmic fraction (**Fig. R1A**). Thus, the amount of Drp1 on mitochondria was prone to high standard deviations but was not significantly different among cells infected with different *L. pneumophila* strains (**Fig. R1B**). Tom20 robustly and exclusively localized to the mitochondrial fraction of *L. pneumophila*-infected cells (**Fig. R1C**) but its amounts did also not differ significantly among the different samples (**Fig. R1D**). Given that

the Western blot approach remained inconclusive, we prefer not to show the corresponding data in the manuscript.

Figure R1

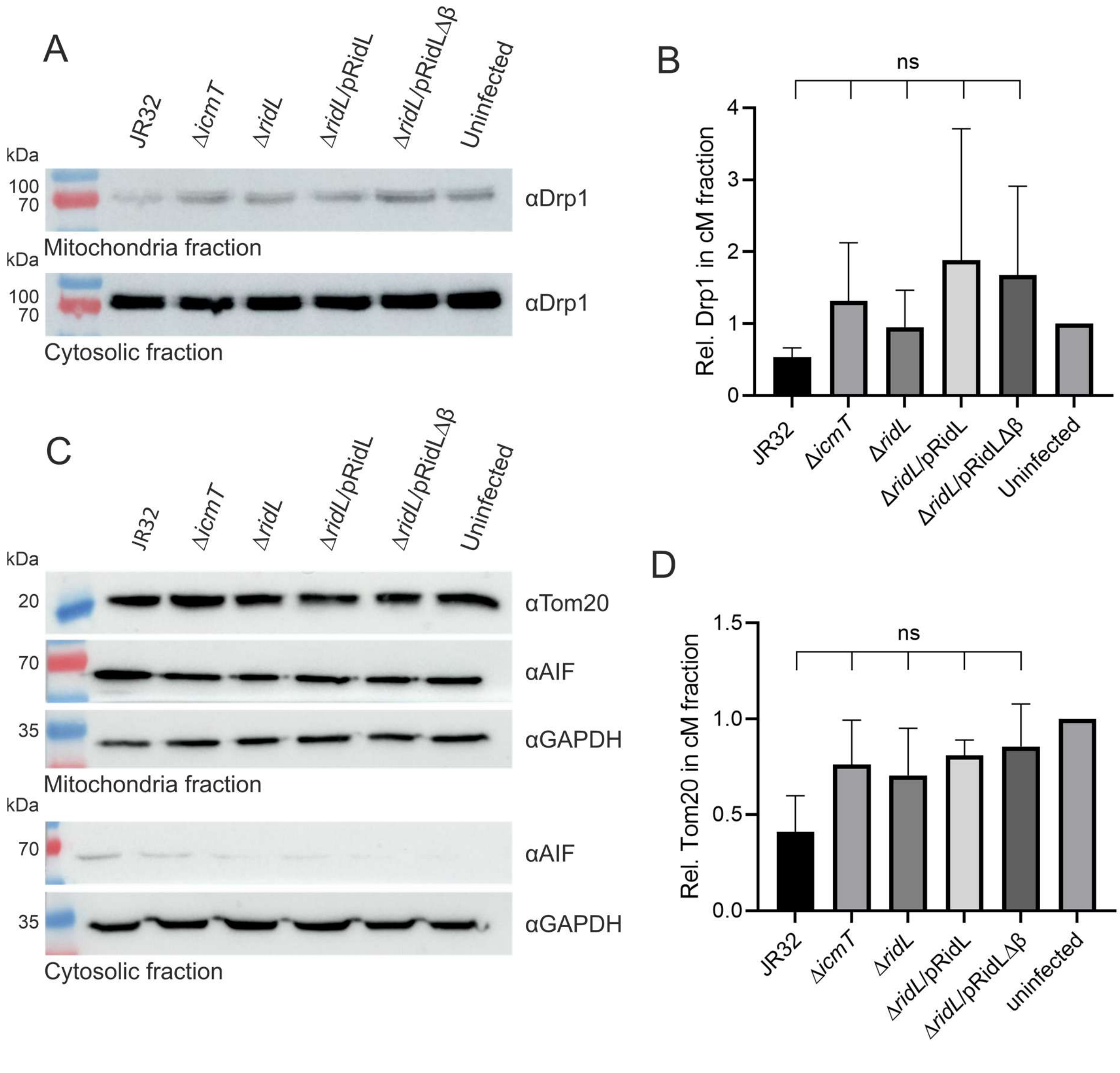

Fig. R1. Localization of Drp1 and Tom20 on isolated mitochondria of infected HeLa cells. (**A**) Western blot analysis of (**A**) Drp1 or (**C**) Tom20 on mitochondrial and cytoplasmic fractions from HeLa cells infected (MOI 100, 2 h) with *L. pneumophila* JR32, Δ*icmT*, Δ*ridL*, Δ*ridL*/pRidL, or Δ*ridL*/pRidL$_{\Delta\beta}$ (pNT28, pIF009, or pKB198). Mitochondria were separated from the cytosol by differential centrifugation. Drp1 localized predominantly in the cytoplasm. AIF served as mitochondria marker, and GAPDH as loading control. (**B**, **D**) Quantification of (A, C). Drp1 and Tom20 intensities were normalized to the corresponding AIF signal and are shown relative to Tom20 signals in uninfected samples. Signal quantification was done in ImageJ. Means and SD of three independent experiments are shown (statistical analysis: one-way ANOVA; ns, not significant).

[6] Mechanistic link between RidL and Drp1 phosphorylation is incomplete. While RidL expression increases Ser616 phosphorylation of Drp1, it remains unclear how this occurs. The co-immunoprecipitation experiments suggested above might help to identify the host signalling components responsible for this modification.

Response: We would like to thank the reviewer for pointing out this knowledge gap. We agree that further analysis of Drp1 phosphorylation by RidL and its effects on mitochondrial fragmentation are important and intriguing aspects of *L. pneumophila* infection. However, we believe that the identification of the kinase catalyzing RidL-dependent Drp1 phosphorylation is beyond the scope of the current study. Some reflections on RidL-promoted phosphorylation of Drp1 by an (unknown) kinase are outlined in the Discussion section (**l. 371-382**).

[7] Physiological relevance of the in vitro Drp1 inhibition is uncertain. The inhibition of Drp1 GTPase activity by RidL requires a 20-fold molar excess and results in less than a twofold reduction in activity (Figure 1H-I). This weak inhibition raises doubts about its significance in cells. Authors need to comment on this and provide evidence that the effect is specific rather than due to protein crowding or aggregation.

Response: We show that RidL promotes the phosphorylation (and presumably activation) of Drp1 (**Fig. 6**) through an (unidentified) kinase. In the biochemical reconstitution and oligomerization experiments (**Figs. 1**, **2**), the unidentified host kinase activating Drp1 is missing, providing a possible explanation for the apparent discrepancy between the in vitro and the in vivo experiments. We have now further outlined this point in the Discussion section (**l. 421-429**). See also above, **point 3**.

[8] Localization data need stronger validation. Evidence for mitochondrial localisation of RidL is strong but would be good to show it is on the outer membrane to support an interaction with Drp1. Additional controls, such as protease protection assays or carbonate extraction, could clarify whether RidL is peripherally associated with the outer membrane or internalised.

Response: Thank you for this suggestion. We performed proteinase K protection assays on mitochondria isolated from HeLa cells ectopically producing RidL or N- or C-terminal fragments of RidL (new **Fig. 4D**). Under the conditions used, RidL and $RidL_C$ were completely

digested while the mitochondria-associated protein AIF displayed partial sensitivity to protease treatment, and the inner mitochondrial membrane protein cytochrome c was protease resistant (**Fig 4D**). These findings indicate that RidL localizes to the outer mitochondrial membrane.

Minor points:

[9] Why is there a considerable difference in GTPase activity of the same conditions (0 μM and 20 μM RidL) between Fig 1G and Fig 1H, especially since the small error bars suggest that there is otherwise minor variability between independent replicates?

Response: Due to availability and technical reasons, the experiments had to be done with two different plate readers, accounting for the apparent differences in GTPase activity.

[10] On graphs that depict the mean of replicate experiments, display the individual data points (e.g. Fig 1G, Fig 1H, Fig 4A, Fig 6).

Response: The individual data points have been added to the **Figs. 1IJ**, **Fig. 3C**, and **Fig. 6C**.

[11] Legend details of Fig 6C do not match those in the method. Can the authors clarify if (and if so, why) the cells were treated with MTDR for flow cytometry?

Response: Thank you for pointing out this oversight. The reference to MitoTracker Deep Red has been deleted from the legend of **Fig. 6C**.

[12] Line 207-213. Keep nomenclature consistent with previous text & figures, i.e. RidL_N and not RidL9-258.

Response: This has been corrected as suggested.

[13] Line 317. Fix spelling, 'hrough'.

Response: Corrected (**l. 386**).

[14] Consider using colourblind-friendly palettes for immunofluorescence microscopy images.

Response: We enhanced the brightness of the images (**Fig. S1, S2**), and we now use fluorescence colors (cyan, magenta, yellow) more easily recognized by color-blind people.

[15] Increase the size of microscopy scale bar text or state the scale bar size in the figure legends.

Response: The length of all microscopy scale bars in now indicated in the figure legends.

Reviewer #3 (Significance (Required)):

This is an interesting study that adds to the growing understanding of how *L. pneumophila* effectors target mitochondria. This is a growing and significant area of interest in the host pathogen space. The data is clear but there are some contradictions that we have raised in our assessment. Undertaking the suggested experiments will strengthen the paper and overall conclusions.

Response: We would like to thank the reviewer for a thorough, constructive, and positive evaluation of our study.

Dear Prof. Hilbi,

Thank you for the submission of your revised manuscript to our editorial offices. I have now received the reports from the three referees that were asked to re-evaluate the study, you will find below. As you will see, the referees now support the publication of the study in EMBO reports. However, referees #1 and #2 have remaining concerns and suggestions to improve the manuscript, I ask you to address in a final revised version. Please also provide a final p-b-p-response to these points and the editorial requests below.

Editorial requests:

- There is a name discrepancy. It is Elizabeth Teresa Vittori in the manuscript vs. Elizabeth Vittori in the submission system. Please check and make sure the name is complete and similar in the submission system and the manuscript title page.

- Please remove the running title from the manuscript title page.

- Please remove the list of abbreviations. Please define each abbreviation the first time it is mentioned in the text.

- Please reduce the number of keywords to five.

- The data availability section is restricted to information on externally deposited datasets. Please remove any other text from this section. If no datasets have been deposited, just state this here.

- The text defining the size of the scale bars in the microscopic images is mostly illegible. Please add scale bars of similar style and thickness to all microscopic images (main, EV and Appendix figures), using clearly visible black or white bars (depending on the background). Please place these in the lower right corner of the images themselves. Please do not write on or near the bars in the image but define the size in the respective figure legend.

- Please check again that the number "n" for how many independent experiments were performed, their nature (biological versus technical replicates), the bars and error bars (e.g. SEM, SD) and the test used to calculate p-values is indicated in the respective figure legends (main and EV figures). Please also check that all the p-values are explained in the legend, and that these fit to those shown in the figure. Please provide statistical testing where applicable. Please avoid the phrase 'independent experiment' but clearly state if these were biological or technical replicates. Please also indicate (e.g. with n.s.) if testing was performed, but the differences are not significant. In case n=2, please show the data as separate datapoints without error bars and statistics. See also:

https://link.springer.com/journal/44319/submission-guidelines#cms-Figure-and-data-presentation

If n<5, please show single datapoints for diagrams (presently, this seems not done!). Some diagrams lack statistics or show only partial statistics (e.g. 4A, 5C/D, EV4A/B and EV5B). For EV3A/B you indicate that data from 2 experiments are shown (n=2). Please show these datasets separated and remove the error par for both panels. Moreover:
- Please note that information related to n is missing in the legend of figure 6B.
- Please note that the error bars are not defined in the legends of figures 5B, 6B, EV5 A
- Please note that the exact p values are not provided in the legends of figures 1I, J; 3C, 4A, G, H; 5B-D; 6B, C; EV4 A, EV5 A
- Please indicate the statistical test used for data analysis in the legend of figure EV1 D

- Please make sure that each figure panel is called out separately and that the panels are called out sequentially. Presently, it seems callouts for Appendix Figs.S1 and S2 seem missing. Please check.

- It seems that the strain and primer information shown in Appendix Tables S1 and S2 are already present in the Reagents & Tools Table. Please check again that this is the case (such information should only be presented in the Reagents & Tools Table) and then remove the tables from the Appendix file. Please update any callouts or add callouts to the Reagents & Tools Table where appropriate.

- Please remove the author names and affiliations from the Appendix Title page. It is sufficient to state "Appendix for ..." followed by the title and then the table of contents. Do not call this 'Supplementary Information'.

- It seems the same Western blot is shown in Fig. 1G and EV1C (left panel). Please check. If the re-use is intentional (and necessary), please clearly indicate this in the respective figure legends.

In addition, I would need from you uploaded separately:
- a short, two-sentence summary of the manuscript (not more than 35 words).
- two to four short (!) bullet points highlighting the key findings of your study (two lines each).

- a schematic summary figure as separate file that provides a sketch of the major findings (not a data image) in jpeg or tiff format (with the exact width of 550 pixels and the exact height of 300 pixels) that can be used as a visual synopsis on our website.

I look forward to seeing the further revised version of your manuscript when it is ready. Please let me know if you have questions regarding the revision.

I look forward to seeing a new revised version of your manuscript as soon as possible.

Best,

Achim Breiling
Senior Editor
EMBO Reports

Referee #1:

Overall the author's responses to reviewer comments have improved the manuscript. There is still, however, one issue related to comment 4 in my initial review. While they have now sufficiently described their quantification of Drp1, Tom20 and pDrp1 colocalization, there is a problem with their approach. Currently, they are thresholding to find ROIs associated with their antibody-labeled signal (ROIaf) and with mitotracker (ROImt). They then find the intersection of ROIaf with ROImt and compare the area of this intersection to the area of all ROIaf. A more appropriate approach is to measure the integrated pixel density in the intersection of the ROIaf with ROImt and compare this with the integrated pixel density of ROIaf. This method incorporates the pixel intensity values which provide information on the relative amount of protein present within the ROIs/ROI intersections.

A couple of small comments:
When describing the flow cytometry assay in Fig6c, (lines 341-343) describe that the cells are being stained for pDrp1.
In the discussion in the sentence discussing interpretation of DLS results (lines 423-426), it is not clear what the interaction of RidL with Drp1 is interfering with.

Referee #2:

The authors have significantly improved the manuscript and addressed most of the questions raised in the first review. The main concern for the revised manuscript is the phospho-S616 Drp1 results in Fig. 6. The evidence of promoting phosphorylation of Drp1 was weak and the specificity of the phospho-antibody could be an issue. The authors should address this antibody issue or remove Fig. 6 and the claim of Drp1 phosphorylation.

Specific points:

Line 122-123, Line 127: odd uses of " - ". For example: " and - intriguingly - the large ".

Line 143: Revise the wording "robust". As the authors stated, Fig EV1C was a result of over-exposure, which demonstrated RidLc can specifically bind to Drp1. However, the interaction is actually much weaker than full-length RidL.

Fig. 2B, Line 192-204: "including the region around 16 nm associated with RidL and showing a peak at 51 nm". Based on this description, it seems like the 16 nm peak is Drp1 associated with RidL. If so, please clarify why the 16 nm peak in Drp1+RidL represented the Drp1+RidL complex but the 12 nm peak in Drp1+SidC represented SidC alone. If the 12 nm peak was the RidL alone, then line 192 should be revised.

Fig. 4A: The figure legend stated the normalization was done by comparing GFP signals of 24-hr to 1-hr. Was this done by comparing each bacterial strain and the knockdown condition? For example, the 100% bar (first black bar) was normalized by scrambled:JR32, 24-hr vs. scrambled:JR32, 1-hr, and the third black bar was normalized by Drp1:JR32, 24-hr vs. Drp1:JR32, 1-hr. If so, please clarify why the first black has no error bars.

Line 334: "Drp1 antibody (Fig 6A and S2)"- Did not see any pS616 data in Fig. S2.

Fig. 6: I appreciated the authors' effort on doing the Western blotting experiments for pS616 Drp1 as they described in the point-by-point letter. However, if even the positive control of pS616 Drp1 was not detected, this raised a concern that the pS616 Drp1 antibody used in the confocal microscopy or flow cytometry may not be specific. This might explain the inconsistency among the results from the microscopy and flow cytometry.

Line 391-392 and Line 414-418: Figure 6 did not provide direct evidence supporting the claim the RidL promotes the accumulation of phosphor-Drp1 on mitochondria. The flow cytometry result was not specific for mitochondria accumulation. The specificity of pS616 Drp1 is questionable as described above.

Referee #3:

I have evaluated the revisions and all of my concerns have been addressed. The authors have undertaken a considerable amount of revisions which has improved the paper significantly. I thank the authors for addressing all of my concerns.

***
Rev_Com_number: RC-2025-03255
New_manu_number: EMBOR-2025-63360V2
Corr_author: Hilbi
Title: The Legionella effector RidL binds the large GTPase Drp1 to promote mitochondrial fragmentation

EMBOR-2025-63360V2 – final revision

Editorial requests:

- There is a name discrepancy. It is Elizabeth Teresa Vittori in the manuscript vs. Elizabeth Vittori in the submission system. Please check and make sure the name is complete and similar in the submission system and the manuscript title page.

Response: The name discrepancy has been resolved.

- Please remove the running title from the manuscript title page.

Response: Done as requested.

- Please remove the list of abbreviations. Please define each abbreviation the first time it is mentioned in the text.

Response: Done as requested.

- Please reduce the number of keywords to five.

Response: Done as requested.

- The data availability section is restricted to information on externally deposited datasets. Please remove any other text from this section. If no datasets have been deposited, just state this here.

Response: Done as requested.

- The text defining the size of the scale bars in the microscopic images is mostly illegible. Please add scale bars of similar style and thickness to all microscopic images (main, EV and Appendix figures), using clearly visible black or white bars (depending on the background). Please place these in the lower right corner of the images themselves. Please do not write on or near the bars in the image but define the size in the respective figure legend.

Response: Done as requested (**Fig. 2A**, **Fig. 3B**, **Fig. 4EF**, **Fig. 5A**, **Fig. 6A**, **Fig. S1**, and **Fig. S2**).

- Please check again that the number "n" for how many independent experiments were performed, their nature (biological versus technical replicates), the bars and error bars (e.g. SEM, SD) and the test used to calculate p-values is indicated in the respective figure legends (main and EV figures). Please also check that all the p-values are explained in the legend, and that these fit to those shown in the figure. Please provide statistical testing where applicable. Please avoid the phrase 'independent experiment' but clearly state if these were biological or technical replicates. Please also indicate (e.g. with n.s.) if testing was performed, but the differences are not significant. In case n=2, please show the data as separate datapoints without error bars and statistics.

Response: We checked all the figure legends again and amended the description of the statistics if necessary. As requested, we show the n = 2 data points separately without error bars (**Fig. EV3AB**).

If n<5, please show single datapoints for diagrams (presently, this seems not done!). Some diagrams lack statistics or show only partial statistics (e.g. 4A, 5C/D, EV4A/B and EV5B). For EV3A/B you indicate that data from 2 experiments are shown (n=2). Please show these datasets separated and remove the error bar for both panels. Moreover:

- Please note that information related to n is missing in the legend of figure 6B.
- Please note that the error bars are not defined in the legends of figures 5B, 6B, EV5A.
- Please note that the exact p values are not provided in the legends of figures 1I, J; 3C, 4A, G, H; 5B-D; 6B, C; EV4A, EV5A.
- Please indicate the statistical test used for data analysis in the legend of figure EV1D.

Response: We checked all the figure legends again and amended the description of the statistics if necessary. As requested, we show the n = 2 data points separately without error bars (**Fig. EV3AB**)

- Please make sure that each figure panel is called out separately and that the panels are called out sequentially. Presently, it seems callouts for Appendix Figs.S1 and S2 seem missing. Please check.

Response: **Fig. S1** and **Fig. S2** are called out (**l. 286**, **l. 337**).

- It seems that the strain and primer information shown in Appendix Tables S1 and S2 are already present in the Reagents & Tools Table. Please check again that this is the case (such information should only be presented in the Reagents & Tools Table) and then remove the tables from the Appendix file. Please update any callouts or add callouts to the Reagents & Tools Table where appropriate.

Response: Done as requested: Appendix **Table S1** and **Table S2** were removed.

- Please remove the author names and affiliations from the Appendix Title page. It is sufficient to state "Appendix for ..." followed by the title and then the table of contents. Do not call this 'Supplementary Information'.

Response: Corrected as requested.

- It seems the same Western blot is shown in Fig. 1G and EV1C (left panel). Please check. If the re-use is intentional (and necessary), please clearly indicate this in the respective figure legends.

Response: The re-use is intentional: for direct comparison, we opt to show **Fig. 1G** again in **Fig. EV1C** (left panel). This is now explicitly stated in the legend to Fig. EV1C.

In addition, I would need from you uploaded separately:
- a short, two-sentence summary of the manuscript (not more than 35 words).
- two to four short (!) bullet points highlighting the key findings of your study (two lines each).
- a schematic summary figure as separate file that provides a sketch of the major findings (not a data image) in jpeg or tiff format (with the exact width of 550 pixels and the exact height of 300 pixels) that can be used as a visual synopsis on our website.

Response: Done as requested.

I look forward to seeing the further revised version of your manuscript when it is ready. Please let me know if you have questions regarding the revision. I look forward to seeing a new revised version of your manuscript as soon as possible.

Best,

Achim Breiling, Senior Editor, EMBO Reports

Referee #1:

Overall the author's responses to reviewer comments have improved the manuscript. There is still, however, one issue related to comment 4 in my initial review. While they have now sufficiently described their quantification of Drp1, Tom20 and pDrp1 colocalization, there is a problem with their approach. Currently, they are thresholding to find ROIs associated with their antibody-labeled signal (ROIaf) and with mitotracker (ROImt). They then find the intersection of ROIaf with ROImt and compare the area of this intersection to the area of all ROIaf. A more appropriate approach is to measure the integrated pixel density in the intersection of the ROIaf with ROImt and compare this with the integrated pixel density of ROIaf. This method incorporates the pixel intensity values which provide information on the relative amount of protein present within the ROIs/ROI intersections.

Response: We would like to thank the reviewer for this suggestion. Accordingly, we have re-analyzed the data in **Fig. 4GH** and **Fig. 6B**. The re-analysis did not fundamentally change the experiments' outcome; however, based on the new quantification approach, the percentage of Drp1 co-localizing with MitoTracker is now significantly higher upon infection with the complemented strain Δ*ridL*/pRidL compared to Δ*ridL* (**Fig. 4G**, **l. 287-292**). The new analysis is now shown in **Fig. 4GH** and **Fig. 6B** and outlined in the Methods section (**l. 892-899**). We also replaced the source data to match the new analysis.

A couple of small comments:

When describing the flow cytometry assay in Fig6c, (lines 341-343) describe that the cells are being stained for pDrp1.

Response: Description added as requested (**l. 343/344**).

In the discussion in the sentence discussing interpretation of DLS results (lines 423-426), it is not clear what the interaction of RidL with Drp1 is interfering with.

Response: We added the word "oligomerization" and apologize for the confusion this omission caused (**l. 428**).

Referee #2:

The authors have significantly improved the manuscript and addressed most of the questions raised in the first review. The main concern for the revised manuscript is the phospho-S616 Drp1 results in Fig. 6. The evidence of promoting phosphorylation of Drp1 was weak and the specificity of the phospho-antibody could be an issue. The authors should address this antibody issue or remove Fig. 6 and the claim of Drp1 phosphorylation.

Response: Using fluorescence microscopy as well as flow cytometry, we provide evidence that in *L. pneumophila*-infected cells, Drp1 is phosphorylated at the activation site in a RidL-dependent manner (**Fig. 6**). For both approaches, we used PFA-fixed cells, and under these conditions, the phospho-Drp1 epitope was recognized by the anti-phospho-Drp1 antibody used. In the flow cytometry approach, control experiments without the primary antibody yielded only background signal. On the other hand, quantification of Drp1 phosphorylation in *L. pneumophila*-infected cells by Western blot yielded inconsistent results. Since gel electrophoresis was performed under denaturing conditions, the phospho-Drp1 epitope is likely altered, possibly compromising the recognition by the anti-phospho-Drp1 antibody. Therefore, different phospho-Drp1 epitope conformations, rather than the specificity of the antibody, might account for the different outcomes of the fluorescence microscopy, flow cytometry, and Western blot approaches. Accordingly, while – if successful – the Western blot approach would have strengthened our hypothesis, failure of the approach does not discredit the results of the independent fluorescence microscopy and flow cytometry approaches. The data shown in Fig. 6 are of great importance for our study, since they provide a putative mechanism for RidL-dependent mitochondria fragmentation. Based on these reflections, we propose keeping **Fig. 6** in the manuscript.

Specific points:

Line 122-123, Line 127: odd uses of " - ". For example: " and - intriguingly - the large ".

Response: We replaced the hyphens with commas (**l. 123/124, l. 128**).

Line 143: Revise the wording "robust". As the authors stated, Fig EV1C was a result of over-exposure, which demonstrated RidLc can specifically bind to Drp1. However, the interaction is actually much weaker than full-length RidL.

Response: We deleted the word "robust" (**l. 144**).

Fig. 2B, Line 192-204: "including the region around 16 nm associated with RidL and showing a peak at 51 nm". Based on this description, it seems like the 16 nm peak is Drp1 associated with RidL. If so, please clarify why the 16 nm peak in Drp1+RidL represented the Drp1+RidL complex but the 12 nm peak in Drp1+SidC represented SidC alone. If the 16 nm peak was the RidL alone, then line 192 should be revised.

Response: We clarify now (**l. 191-204**) that the peaks around 16 nm and 12 nm represent RidL and SidC only, as documented in Fig. EV2BC. Moreover, we further outline our interpretation of the DLS results in the Discussion section (**l. 427-435**).

Fig. 4A: The figure legend stated the normalization was done by comparing GFP signals of 24-hr to 1-hr. Was this done by comparing each bacterial strain and the knockdown condition? For example, the 100% bar (first black bar) was normalized by scrambled:JR32, 24-hr vs. scrambled:JR32, 1-hr, and the third black bar was normalized by Drp1:JR32, 24-hr vs. Drp1:JR32, 1-hr. If so, please clarify why the first black has no error bars.

Response: We now outline in the Methods section the detailed RNAi procedure (**l. 857-863**): For RNAi analysis, the background signal measured for untreated A549 cells was subtracted from the measured values of infected cells. The means of each three technical replicates measured 24 h post infection were divided by the means measured 1 h p.i to show relative intracellular replication. For normalization to treatment with Scrambled siRNA, relative means were normalized to the relative mean of the Scrambled sample of each biological replicate. To compensate for day-to-day variations, the data for Scrambled was set to 100 for each of the different biological replicates.

Line 334: "Drp1 antibody (Fig 6A and S2)"- Did not see any pS616 data in Fig. S2.

Response: pDrp1 (Alexa Fluor 488) is indicated in **Fig. S2**.

Fig. 6: I appreciated the authors' effort on doing the Western blotting experiments for pS616 Drp1 as they described in the point-by-point letter. However, if even the positive control of pS616 Drp1 was not detected, this raised a concern that the pS616 Drp1 antibody used in the confocal microscopy or flow cytometry may not be specific. This might explain the inconsistency among the results from the microscopy and flow cytometry.

Response: The results from fluorescence microscopy (**Fig. 6AB**) and flow cytometry (**Fig. 6C**) are entirely consistent and coherent. However, the Western blot results using the same anti-phospho-Drp1 antibody are inconclusive. As outlined above, the discrepancy among the three approaches might be due to different phospho-Drp1 epitope conformations, rather than due to the lack of specificity of the antibody.

Line 391-392 and Line 414-418: Figure 6 did not provide direct evidence supporting the claim the RidL promotes the accumulation of phospho-Drp1 on mitochondria. The flow cytometry result was not specific for mitochondria accumulation. The specificity of pS616 Drp1 is questionable as described above.

Response: While the flow cytometry approach does not provide subcellular spatial information about phospho-Drp1, the fluorescence microscopy approach indeed does so. We now word the corresponding statements more carefully (**l. 395-398**) and indicate the specific panel of the figure (**Fig. 6AB**, **l. 419**).

Referee #3:

I have evaluated the revisions and all of my concerns have been addressed. The authors have undertaken a considerable amount of revisions which has improved the paper significantly. I thank the authors for addressing all of my concerns.

Response: We would like to thank the referee once again for a thorough and constructive review.

2nd Revision - Editorial Decision 28th May 2026

Prof. Hubert Hilbi
University of Zürich
Institute of Medical Microbiology
Gloriastrasse 30
Zürich 8006
Switzerland

Dear Prof. Hilbi,

Thank you for the submission of your final revised manuscript to our editorial offices. I now looked through this and your final p-b-p-response and consider the remaining points of the referees and the editorial requests as adequately addressed.

I thus am very pleased to accept your manuscript for publication in the next available issue of EMBO reports. Thank you for your contribution to our journal.

Your manuscript will be processed for publication by EMBO Press. It will be copy edited and you will receive page proofs prior to publication. Please note that you will be contacted by Springer Nature Author Services to complete licensing and payment information.

You may qualify for financial assistance for your publication charges - either via a Springer Nature fully open access agreement or an EMBO initiative. Check your eligibility: https://link.springer.com/journal/44319/how-to-publish-with-us

Should you be planning a Press Release on your article, please notify the production team via this form in order to coordinate publication and release dates: https://forms.cloud.microsoft/e/eH4A8JVP6g

If you have any questions, please do not hesitate to contact the Editorial Office. Thank you for your contribution to EMBO Reports.

Yours sincerely,

Achim Breiling
Senior Editor
EMBO Reports

>>> Please note that it is EMBO Reports policy for the transcript of the editorial process (containing referee reports and your response letter) to be published as an online supplement to each paper. If you do NOT want this, you will need to inform the Editorial Office via email immediately. More information is available here: https://link.springer.com/partners/embo-press/editorial-policies#Peer%20review

EMBO Press Author Checklist

Corresponding Author Name: Hubert Hilbi
Journal Submitted to: EMBO Reports
Manuscript Number: EMBOR-2025-63360

USEFUL LINKS FOR COMPLETING THIS FORM
The EMBO Journal - Author Guidelines
EMBO Reports - Author Guidelines
Molecular Systems Biology - Author Guidelines
EMBO Molecular Medicine - Author Guidelines

Reporting Checklist for Life Science Articles (updated January
This checklist is adapted from Materials Design Analysis Reporting (MDAR) Checklist for Authors. MDAR establishes a minimum set of requirements in transparent reporting in the life sciences (see Statement of Task: 10.31222/osf.io/9sm4x). Please follow the journal's guidelines in preparing your
Please note that a copy of this checklist will be published alongside your article.

Abridged guidelines for figures
1. Data
The data shown in figures should satisfy the following conditions:
- ➞ the data were obtained and processed according to the field's best practice and are presented to reflect the results of the experiments in an accurate and unbiased manner.
- ➞ ideally, figure panels should include only measurements that are directly comparable to each other and obtained with the same assay.
- ➞ plots include clearly labeled error bars for independent experiments and sample sizes. Unless justified, error bars should not be shown for technical
- ➞ if n<5, the individual data points from each experiment should be plotted. Any statistical test employed should be justified.
- ➞ Source Data should be included to report the data underlying figures according to the guidelines set out in the authorship guidelines on Data

2. Captions
Each figure caption should contain the following information, for each panel where they are relevant:
- ➞ a specification of the experimental system investigated (eg cell line, species name).
- ➞ the assay(s) and method(s) used to carry out the reported observations and measurements.
- ➞ an explicit mention of the biological and chemical entity(ies) that are being measured.
- ➞ an explicit mention of the biological and chemical entity(ies) that are altered/varied/perturbed in a controlled manner.
- ➞ the exact sample size (n) for each experimental group/condition, given as a number, not a range;
- ➞ a description of the sample collection allowing the reader to understand whether the samples represent technical or biological replicates (including how many animals, litters, cultures, etc.).
- ➞ a statement of how many times the experiment shown was independently replicated in the laboratory.
- ➞ definitions of statistical methods and measures:
  - common tests, such as t-test (please specify whether paired vs. unpaired), simple χ^2 tests, Wilcoxon and Mann-Whitney tests, can be unambiguously identified by name only, but more complex techniques should be described in the methods section;
  - are tests one-sided or two-sided?
  - are there adjustments for multiple comparisons?
  - exact statistical test results, e.g., P values = x but not P values < x;
  - definition of 'center values' as median or average;
  - definition of error bars as s.d. or s.e.m.

Please complete ALL of the questions below.
Select "Not Applicable" only when the requested information is not relevant for your study.

Materials

Newly Created Materials	Information included in the manuscript?	In which section is the information available? (Reagents and Tools Table, Materials and Methods, Figures, Data Availability Section)
New materials and reagents need to be available; do any restrictions apply?	Yes	Reagents and Tools Table, Materials and Methods, Data Availability Section, Table S1, Table S2

Antibodies	Information included in the manuscript?	In which section is the information available? (Reagents and Tools Table, Materials and Methods, Figures, Data Availability Section)
For **antibodies** provide the following information:<br>- Commercial antibodies: RRID (if possible) or supplier name, catalogue number and or/clone number<br>- Non-commercial: RRID or citation	Yes	Reagents and Tools Table, Materials and Methods,

DNA and RNA sequences	Information included in the manuscript?	In which section is the information available? (Reagents and Tools Table, Materials and Methods, Figures, Data Availability Section)
**Short novel DNA or RNA including primers, probes**: provide the sequences.	Yes	Reagents and Tools Table, Materials and Methods, Table S1, Table S2

Cell materials	Information included in the manuscript?	In which section is the information available? (Reagents and Tools Table, Materials and Methods, Figures, Data Availability Section)
**Cell lines:** Provide species information, strain. Provide accession number in repository **OR** supplier name, catalog number, clone number, and/**OR** RRID.	Yes	Reagents and Tools Table, Materials and Methods
**Primary cultures:** Provide species, strain, sex of origin, genetic modification status.	Not Applicable	
Report if the cell lines were recently **authenticated** (e.g., by STR profiling) and tested for mycoplasma contamination.	Not Applicable	

Experimental animals	Information included in the manuscript?	In which section is the information available? (Reagents and Tools Table, Materials and Methods, Figures, Data Availability Section)
**Laboratory animals or Model organisms:** Provide species, strain, sex, age, genetic modification status. Provide accession number in repository **OR** supplier name, catalog number, clone number, **OR** RRID.	Not Applicable	
**Animal observed in or captured from the field:** Provide species, sex, and age where possible.	Not Applicable	
Please detail **housing and husbandry conditions**.	Not Applicable	

Plants and microbes	Information included in the manuscript?	In which section is the information available? (Reagents and Tools Table, Materials and Methods, Figures, Data Availability Section)
**Plants:** provide species and strain, ecotype and cultivar where relevant, unique accession number if available, and source (including location for collected wild specimens).	Not Applicable	
**Microbes:** provide species and strain, unique accession number if available, and source.	Yes	Reagents and Tools Table, Materials and Methods, Table S1

Human research participants	Information included in the manuscript?	In which section is the information available? (Reagents and Tools Table, Materials and Methods, Figures, Data Availability Section)
If collected and within the bounds of privacy constraints report on age, sex and gender or ethnicity for all study participants.	Not Applicable	

Core facilities	Information included in the manuscript?	In which section is the information available? (Reagents and Tools Table, Materials and Methods, Figures, Data Availability Section)
If your work benefited from core facilities, was their service mentioned in the acknowledgments section?	Yes	Acknowledgments

Design

Study protocol	Information included in the manuscript?	In which section is the information available? (Reagents and Tools Table, Materials and Methods, Figures, Data Availability Section)
If study protocol has been **pre-registered, provide DOI in the manuscript**. For clinical trials, provide the trial registration number **OR** cite DOI.	Not Applicable	
Report the **clinical trial registration number** (at ClinicalTrials.gov or equivalent), where applicable.	Not Applicable	

Laboratory protocol	Information included in the manuscript?	In which section is the information available? (Reagents and Tools Table, Materials and Methods, Figures, Data Availability Section)
Provide DOI OR other citation details if **external detailed step-by-step protocols** are available.	Not Applicable	

Experimental study design and statistics	Information included in the manuscript?	In which section is the information available? (Reagents and Tools Table, Materials and Methods, Figures, Data Availability Section)
Include a statement about **sample size** estimate even if no statistical methods were used.	Yes	Bioinformatics and Statistics Section, Figure Legends
Were any steps taken to minimize the effects of subjective bias when allocating animals/samples to treatment (e.g. **randomization procedure**)? If yes, have they been described?	Not Applicable	
Include a statement about **blinding** even if no blinding was done.	Not Applicable	
Describe **inclusion/exclusion criteria** if samples or animals were excluded from the analysis. Were the criteria pre-established?<br><br>If sample or data points were omitted from analysis, report if this was due to attrition or intentional exclusion and provide justification.	Not Applicable	
For every figure, are **statistical tests** justified as appropriate? Do the data meet the assumptions of the tests (e.g., normal distribution)? Describe any methods used to assess it. Is there an estimate of variation within each group of data? Is the variance similar between the groups that are being statistically compared?	Yes	Figure Legends

Sample definition and in-laboratory replication	Information included in the manuscript?	In which section is the information available? (Reagents and Tools Table, Materials and Methods, Figures, Data Availability Section)
In the figure legends: state number of times the experiment was **replicated** in laboratory.	Yes	Figure Legends
In the figure legends: define whether data describe **technical or biological replicates**.	Yes	Figure Legends

Ethics

Ethics	Information included in the manuscript?	In which section is the information available? (Reagents and Tools Table, Materials and Methods, Figures, Data Availability Section)
Studies involving **human participants**: State details of **authority granting ethics approval** (IRB or equivalent committee(s), provide reference number for approval.	Not Applicable	
Studies involving **human participants**: Include a statement confirming that **informed consent** was obtained from all subjects and that the experiments conformed to the principles set out in the WMA Declaration of Helsinki and the Department of Health and Human Services Belmont Report.	Not Applicable	
Studies involving **human participants:** For publication of **patient photos**, include a statement confirming that consent to publish was obtained.	Not Applicable	
Studies involving experimental **animals**: State details of **authority granting ethics approval** (IRB or equivalent committee(s), provide reference number for approval. Include a statement of compliance with ethical regulations.	Not Applicable	
Studies involving **specimen and field samples:** State if relevant **permits** obtained, provide details of authority approving study; if none were required, explain why.	Not Applicable	

Dual Use Research of Concern (DURC)	Information included in the manuscript?	In which section is the information available? (Reagents and Tools Table, Materials and Methods, Figures, Data Availability Section)
Could your study fall under dual use research restrictions? Please check biosecurity documents and list of **select agents and toxins** (CDC): https://www.selectagents.gov/sat/list.htm	Not Applicable	
If you used a select agent, is the security level of the lab appropriate and reported in the manuscript?	Not Applicable	
If a study is subject to dual use research of concern regulations, is the name of the **authority granting approval and reference number** for the regulatory approval provided in the manuscript?	Not Applicable	

Reporting

The MDAR framework recommends adoption of discipline-specific guidelines, established and endorsed through community initiatives. Journals have their own policy about requiring specific guidelines and recommendations to complement MDAR.

Adherence to community standards	Information included in the manuscript?	In which section is the information available? (Reagents and Tools Table, Materials and Methods, Figures, Data Availability Section)
State if relevant guidelines or checklists (e.g., **ICMJE, MIBBI, ARRIVE, PRISMA**) have been followed or provided.	Not Applicable	
For **tumor marker prognostic studies,** we recommend that you follow the **REMARK** reporting guidelines (see link list at top right). See author guidelines, under 'Reporting Guidelines'. Please confirm you have followed these guidelines.	Not Applicable	
For **phase II and III randomized controlled trials**, please refer to the **CONSORT** flow diagram (see link list at top right) and submit the CONSORT checklist (see link list at top right) with your submission. See author guidelines, under 'Reporting Guidelines'. Please confirm you have submitted this list.	Not Applicable	

Data Availability

Data availability	Information included in the manuscript?	In which section is the information available? (Reagents and Tools Table, Materials and Methods, Figures, Data Availability Section)
Have **primary datasets** been deposited according to the journal's guidelines (see 'Data Deposition' section) and the respective accession numbers provided in the Data Availability Section?	Yes	Data Availability Section
Were **human clinical and genomic datasets** deposited in a public access-controlled repository in accordance to ethical obligations to the patients and to the applicable consent agreement?	Not Applicable	
Are **computational models** that are central and integral to a study available without restrictions in a machine-readable form? Were the relevant accession numbers or links provided?	Not Applicable	
If publicly available data were reused, provide the respective **data citations in the reference list**.	Not Applicable	
